# Activity-based proteomics discovery of deubiquitinating enzyme inhibitors with immunomodulatory activity

**DOI:** 10.1038/s44386-026-00092-z

**Published:** 2026-10-26

**Authors:** Hannah B. L. Jones, Simeon D. Draganov, Sofia Schönbauer Huamán, Peter A. C. Wing, Chuong Nguyen, Zhu Liang, Julia Dörner, Jasper Lithgow, Emma Murphy, Alice Beard, Angela Chen, Andrea Pierangelini, Jack W. Houghton, Mackenzie E. Robert, Sarah Flannery, Cara J. Ellison, Sakshi Sharma, Anthony Tumber, Lynn Wambua, Iolanda Vendrell, Edward W. Tate, Paul R. Elliott, Graham M. West, Darragh P. O’Brien, Eidarus Salah, Andrew P. Turnbull, Christopher J. Schofield, Benedikt M. Kessler, Lennart Brewitz, Adán Pinto-Fernández

**Affiliations:** 1 Nuffield Department of Medicine, Centre for Medicines Discovery, Target Discovery Institute, University of Oxford, Oxford, UK; 2 Nuffield Department of Medicine, CAMS Oxford Institute and Centre for Medicines Discovery, University of Oxford, Oxford, UK; 3 Chemistry Research Laboratory, Department of Chemistry and the Ineos Oxford Institute for Antimicrobial Research, University of Oxford, Oxford, UK; 4 Hit Discovery and Optimization (HDO) Pfizer Inc, Groton, CT USA; 5 Department of Chemistry, Molecular Sciences Research Hub, Imperial College London, London, UK; 6 Discovery Biology and Pharmacology, Pfizer Inc, Groton, CT USA; 7 Department of Biochemistry, University of Oxford, Oxford, UK; 8 Cancer Research Horizons, Francis Crick Institute, London, UK; 9 Centre for Chemical Biology, Institute of Physical Chemistry, Polish Academy of Sciences, Warsaw, Poland; 10 Present Address: Department of Microbiology, Harvard Medical School, Boston, MA USA

**Keywords:** Biochemistry, Computational biology and bioinformatics, Drug discovery

## Abstract

Deubiquitinases (DUBs) play key roles in human pathologies, including cancer, infectious, autoimmune, and neurodegenerative diseases. The progress of potent and selective active site-targeting DUB inhibitors into clinical trials has demonstrated the therapeutic opportunities of such molecules; however, the shared catalytic geometry among DUB family members poses a challenge for achieving inhibitor selectivity. We describe a high-throughput, DUB-focused, activity-based proteomics workflow to identify new types of cysteine-reactive DUB inhibitors. We demonstrate the potential of αβ,α′β′-diepoxyketones (DEKs) and ubiquitin-derived covalently reacting alkynes as the first selective, cell-active compounds targeting USP47, OTUD7B (Cezanne), and USP5. These DUBs are reported to have critical roles in inflammasome formation, hypoxia, and viral replication, respectively. The identified compounds phenocopy these findings, demonstrating the tractability of these DUBs as immunotherapeutic targets.

## Introduction

Post-translational (poly)-ubiquitination regulates key cellular processes, including protein and organelle homeostasis, through the interplay of ubiquitinating and deubiquitinating molecular machineries^1–4^. Deubiquitinases (DUBs) are mostly nucleophilic cysteine enzymes that cleave ubiquitin (Ub) from biomolecule substrates, mainly proteins, modulating their fate^5,6^. Consequently, dysregulated DUB functions have been linked to diseases including cancer, neurodegeneration, inflammation, infectious disease, or immune-mediated disease^1^.

DUBs cleave amide bonds after the conserved C-terminal LRGG motif of ubiquitin. Despite sharing a broadly conserved active-site architecture, individual DUBs discriminate among distinct polyubiquitin chain linkages and ubiquitinated substrates through differences in ubiquitin-binding interfaces (including at the active site)^7^ and allosteric regulatory elements. This combination of conserved catalytic geometry and context-dependent substrate recognition complicates the development of selective active-site-directed DUB inhibitors^8^. Nonetheless, both active-site targeting and allosteric small-molecule inhibitors of USP1, USP28, and USP30 have been developed, and progressed into clinical trials for treatment of advanced solid tumors^9,10^, relapsed/refractory acute myeloid leukemia (R/R AML)^11^, chronic kidney disease^12^, and Parkinson’s disease^3,13^.

DUB activity-based protein profiling (ABPP) was established to profile DUB activities in complex systems. DUB ABPP employs activity-based probes (ABPs) that react with the nucleophilic cysteine of DUBs^14–18^, including haemagglutinin (HA)-tagged ubiquitin-propargylamide (HA-Ub-PA), which bears an alkyne warhead for irreversible covalent reaction with active-site cysteines. The alkyne group acts as a latent electrophile that generally requires an appropriately activated and positioned nucleophilic cysteine, often within a catalytic triad or dyad, to undergo covalent reaction. This requirement for productive active-site templating helps explain the relatively high target selectivity observed for ubiquitin-derived alkyne probes and inhibitors (13–16), which is a key criterion for ABPs^14–18^. Coupled with affinity purification and liquid chromatography-tandem mass spectrometry (LC-MS/MS), ABPP enables the analysis of endogenous DUB-ABP complexes. Unlike recombinant enzyme assays, ABPP supports the identification of potent and selective inhibitors in a cellular environment, including those for challenging targets^19,20^, whilst also providing information on cell permeability and stability^21–23^.

Historically, the scope of ABPP has been limited by sensitivity and throughput because of labor-intensive workflows involving ABP labeling, purification, sample preparation, MS analysis, and data processing. To enhance its utility for DUB inhibitor discovery, we previously increased throughput by adapting the probe purification step to a 96-well plate format, integrating an automated liquid handling system (AssayMAP Bravo) with ultra-fast, sensitive LC-MS/MS instrumentation (Evosep-timsTOF). This method (ABPP-HT) facilitated analysis of ~100 samples daily^24^. We subsequently enhanced the sensitivity of ABPP-HT by incorporating data-independent acquisition LC-MS/MS (DIA), enabling deeper and more comprehensive DUBome profiling (ABPP-HT*)^25^. Additional improvements aimed at enhancing sensitivity were tested and include using diverse Ub probe approaches, high-pH pre-fractionation^26^, and isobaric labeling (Figure S1A-C)^19^.

Here, we advance the ABPP-HT* platform into a broadly accessible and scalable workflow, enhancing its sensitivity, flexibility, and ease of use while establishing a fully functional on-bench (non-automated) format. As a proof of concept, we applied the platform to the discovery of unconventional cysteine-reactive and selective DUB-targeting αβ,α′β′-diepoxyketones (DEKs) that selectively engage the active sites of USP47/USP7 and OTUD7B. The platform also enabled the identification and optimization of a ubiquitin-derived inhibitor class that uses an alkyne as a latent electrophile for covalent engagement, yielding probes with selective cellular inhibition of USP5. These compounds performed robustly in functional assays and are useful tools for assigning DUB-dependent phenotypes in cells. Consistent with reported biology, the USP47/USP7, OTUD7B, and USP5 inhibitors modulated pathways linked to innate immunity, hypoxic signaling, and viral infection by suppressing inflammasome activation, stabilizing HIF-1α, and relieving inhibition of type I interferon signaling, respectively.

## Results

**Fig. 1:**
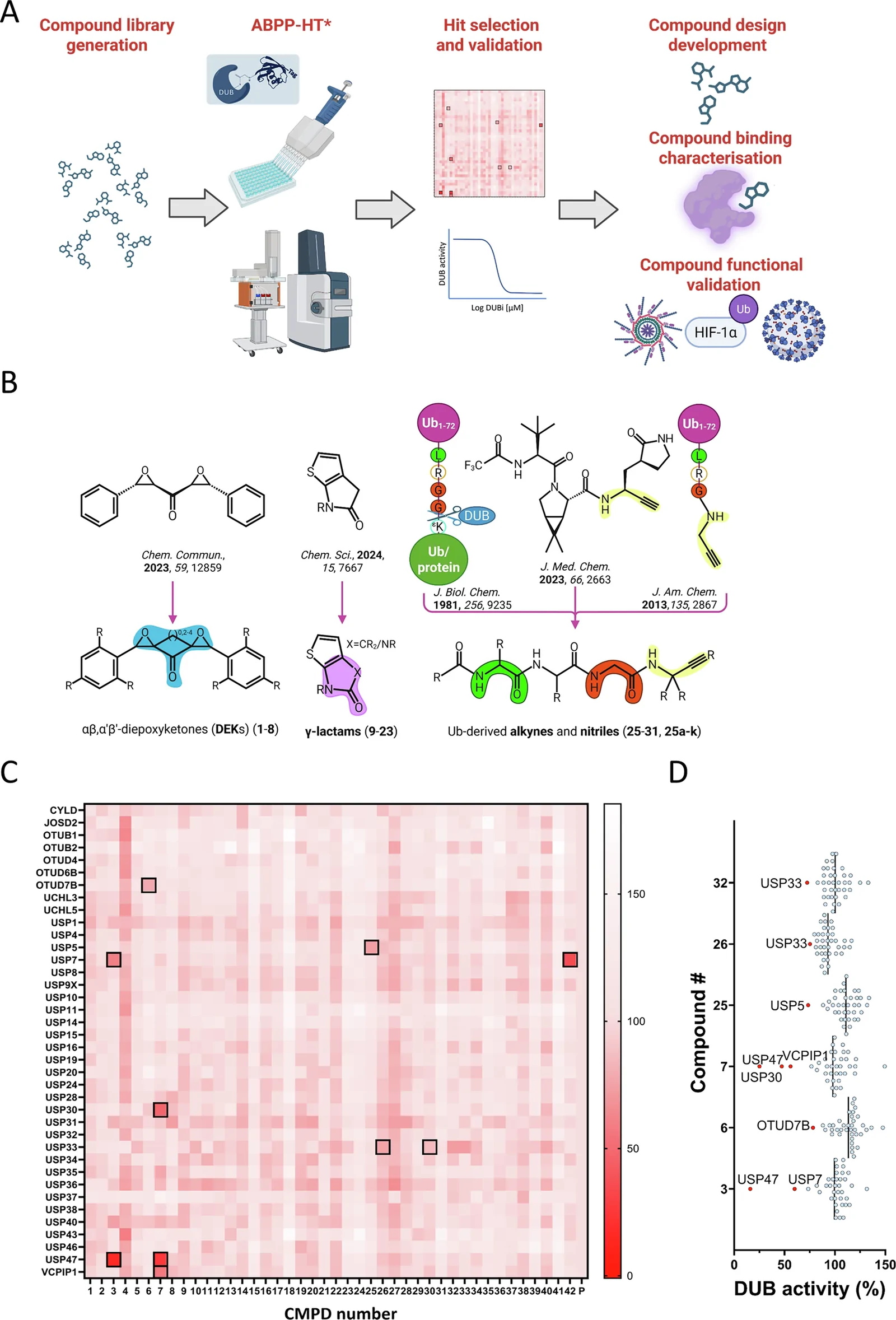
DUB-targeting compound screen using activity proteomics and a cysteine-centric compound library. **A** ABPP-HT* DUB-targeting compound screen workflow. Created in BioRender. Jones, H. (2025) (License link to be inserted upon publication). **B** Design rationale for DUB inhibitor chemotypes. **C** Heatmap of DUBs identified from ABPP-HT* screen, performed using MCF-7 lysates. DUB activity in the presence of 25 µM of **Compounds 1-41** (**Compound 42** = established USP7 inhibitor FT827) as a % of positive control activity (*n* = 2, controls *n* = 4). Cells outlined with black squares indicate >20% probe labeling reduction (*p* < 0.05—two tailed unpaired t-test, (**Compound 6** OTUD7B *p* = 0.066)), with significant probe labeling reduction of **Compounds 4** and **27** not highlighted due to lack of selectivity. **D** Significant hits from ABPP-HT* screen taken forward for validations, showing reduction in activity relative to other DUBs.

We modified our reported ABPP-HT* methodology^24,25^ by adapting the immunoprecipitation step to either a multi-well magnetic rack for on-bench processing or a KingFisher Apex purification system, demonstrating the workflow’s flexibility (Fig. S1D). Compared to using an automated liquid handling platform (AssayMAP Bravo), the new method manifested comparable or improved enrichment of cysteine-reactive DUBs (Fig. S1E–I). The improved and user-friendly “on-bench” ABPP-HT* set-up was then used to identify novel types of DUB-targeting small-molecules from a set of unconventional cysteine-reactive small-molecules (Fig. 1A).

Four complementary classes of small-molecules were investigated in the screen: (i) αβ,α′β′-diepoxyketones (DEKs), which are reported to covalently react with the nucleophilic cysteine residues of bacterial and viral enzymes^27^, (ii) electronically activated γ-lactams bearing N-electron-withdrawing substituents, which are substantially less reactive than DEKs but can covalently react with Cys145 of the SARS-CoV-2 main protease when appropriately positioned at the active site^28^, (iii) Michael acceptors, which are a common motif in covalently reacting small-molecule drugs, and (iv) Ub-derived alkyne and nitrile inhibitors that may bind at the active site (Supplementary Data S1-3) (Fig. 1B); note that classes (i), (ii), and (iv) are relatively uncommon in established DUB inhibitor libraries. The latter class of inhibitors was designed based on the Ub C-terminal LRGG motif^29^, replacing the C-terminal glycine by 2-aminoacetonitrile or propargyl amide (PA) to enable reversible or irreversible covalent reaction with the DUB active site cysteine thiol, respectively; note that the arginine residue at the P3-equivalent position was substituted for a lysine group to facilitate chemical modification. This design strategy was informed by the observations that Ub-PA^17,30–32^ and nitrile, as well as alkyne-containing substrate-derived small-molecules are potent and selective active site-targeting inhibitors of nucleophilic cysteine enzymes^33–38^. Compounds from classes (i) and (ii) were synthesized in our group for prior studies on unconventional cysteine-reactive chemotypes^27,28^, whereas compounds in class (iv) were designed specifically for this study. Of note, the ABPP-HT* setup can identify small molecules that engage DUBs through irreversible or reversible covalent mechanisms, provided that probe-labeling parameters, including probe stoichiometry, labeling time, and temperature, are optimized to account for compound residence time and competition with the ABP, as described previously^24^.

A total of 41 small-molecules were thus incubated with MCF-7 breast cancer cell lysates in a 96 well plate in technical duplicates. FT827, a well-characterized USP7 inhibitor^39^, was included as a reference inhibitor; negative and positive controls (absence or presence of HA-Ub-PA) were included on the plate. The results reveal that 38 DUBs were consistently enriched >5-fold across replicates using the ABPP-HT* workflow, with an average coefficient of variation (CV) of 16%, confirming robustness (Fig. S2A). Importantly, we confirmed potent and selective reduction of USP7 probe labeling by FT827^39^, further validating this assay.

Eight compounds reduced probe labeling of at least one DUB by >20% in the primary screen. Most met the initial statistical threshold (*p* < 0.05), whereas **Compound 6** showed borderline OTUD7B engagement (*p* = 0.066), but was retained because of the magnitude and selectivity of the effect, and was subsequently validated by orthogonal assays (Fig. 1C). Of these, **Compounds 4** and **27** were excluded due to a lack of selectivity. Selective, or partially selective, hits included: **Compound 3** (USP47 > USP7), **Compound 6** (OTUD7B), **Compound 7** (USP30/USP47/VCPIP1), **Compound 25** (USP5), and **Compounds 26** and **32** (USP33) (Fig. 1D). Notably, USP5-selective **Compound 25** is a diastereomer of the inactive **Compound 27**, highlighting the workflow’s ability to resolve isomer-specific effects (Fig. S2B, C).

### Hit validation and dose-response analysis

**Fig. 2:**
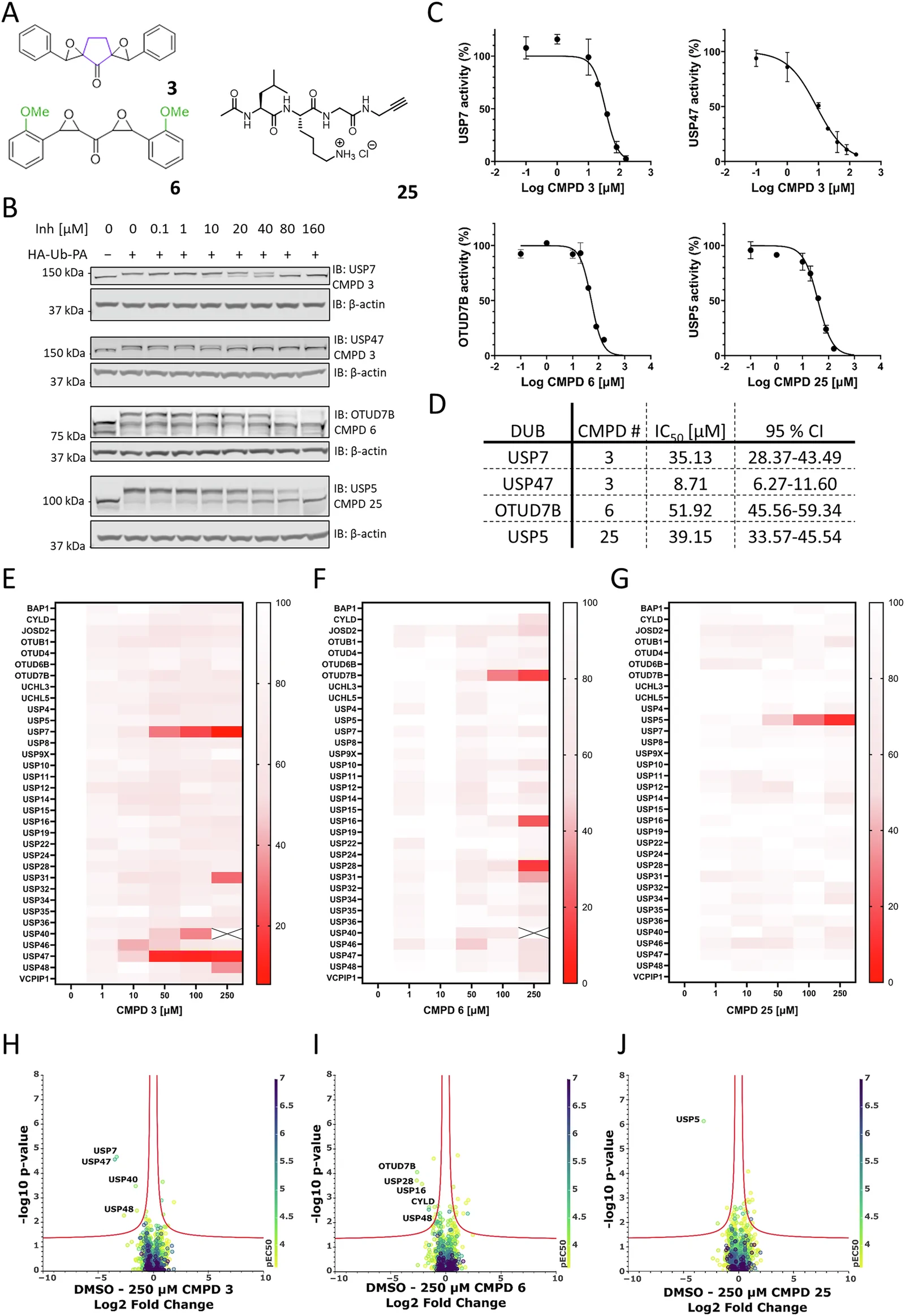
Hit validation and dose-response analysis. **A** Compound structures of positive hits. **B** HA-Ub-PA ABP validations in MCF-7 cell lysates showing concentration-dependent probe labeling reduction inhibition of DUBs by positive hits identified from ABPP-HT* screen. **C** Densitometric quantitation from western blots of positive hits (*n* = 2) showing log compound concentration vs. normalized DUB activity, fit to *Y* = 100/(1 + 10^((LogIC_50_-X)*HillSlope)) (error bars = SD). **D** IC_50_ values extracted from fits in (**C**), (CI = Confidence interval). ABPP-HT* DUB activity with concentration-dependence of **Compounds 3** (**E**), **6** (**F**), and **25** (**G**) (*n* = 2, controls *n* = 3). Curve curator volcano plots^87^, showing Log_2_ Fold change decrease in DUB intensity with 250 µM of **Compound 3** (**H**), **6** (**I**), and **25** (**J**). Color is reflective of pEC50 values across concentration-dependence shown in (**E**–**G**).

Western blot analysis supported engagement of USP47/USP7 by **Compound 3**, OTUD7B by **Compound 6**, and USP5 by **Compound 25** (Fig. 2A, B). DUB probe-labeling reduction calculated from western blot densitometric analysis was concentration-dependent (Fig. 2C) and IC₅₀ values revealed the preference of **Compound 3** for USP47 (~8.8 μM) over USP7 (~36 μM) (Fig. 2D), which was consistent with screening data. The apparent preference of **Compound 3** for USP47 over USP7 was remarkable given that reported USP47 inhibitors typically also inhibit USP7 with similar or lower potency^40,41^, likely reflecting the close evolutionary relationship between human USP47 and USP7 (their catalytic domains share ~40%–45% sequence identity, depending on the alignment boundaries used)^42,43^.

**Compounds 26** and **32** appeared to be false positive hits and could not be validated (Fig. S2D), while **Compound 7** induced protein cross-linking, likely due to its bifunctional epoxide structure (Fig. S2E)^27^. To assess binding stability, **Compounds 3,** **6**, and **25** were pre-incubated with MCF-7 lysates for 30 min before the addition of HA-Ub-PA and incubation for 5-, 15-, or 45-min. Efficient prevention of HA-Ub-PA binding was observed across all timepoints (Fig. S2F–H), suggesting irreversible binding of **Compounds 3,** **6**, and **25** at the DUB active sites.

The increased throughput of the ABPP-HT* workflow enabled systematic evaluation of the selectivity of **Compounds 3,** **6**, and **25** across multiple concentrations and replicates (Fig. 2E–G). The dose-dependent inhibition curves and IC_50_ values for the three compounds were comparable to those generated by immunoblotting (Fig. S3A–D)^24^. The results reveal that **Compound 3** selectively reduced USP47 and USP7 probe labeling up to 50 µM, with reduced USP40 and USP48 probe labeling observed at higher concentrations (Fig. 2E, H). **Compound 6** was selective for OTUD7B up to 100 µM but also reduced probe labeling of USP16 and USP28 at the highest concentration (250 µM) (Fig. 2F, I). Note that **Compounds 3** and **6** are reported to inhibit the mycobacterial L,D-transpeptidase Ldt_Mt2_ and the SARS-CoV-2 main protease^27^, indicating that DUBome selectivity does not exclude additional cysteine-dependent targets outside the DUB family. By contrast to **Compounds 3** and **6,** **Compound 25** was highly selective, only reducing probe labeling of USP5 across the tested concentration range (Fig. 2G, J).

### Molecular basis for selective inhibition of USP47 by compound 3

**Fig. 3:**
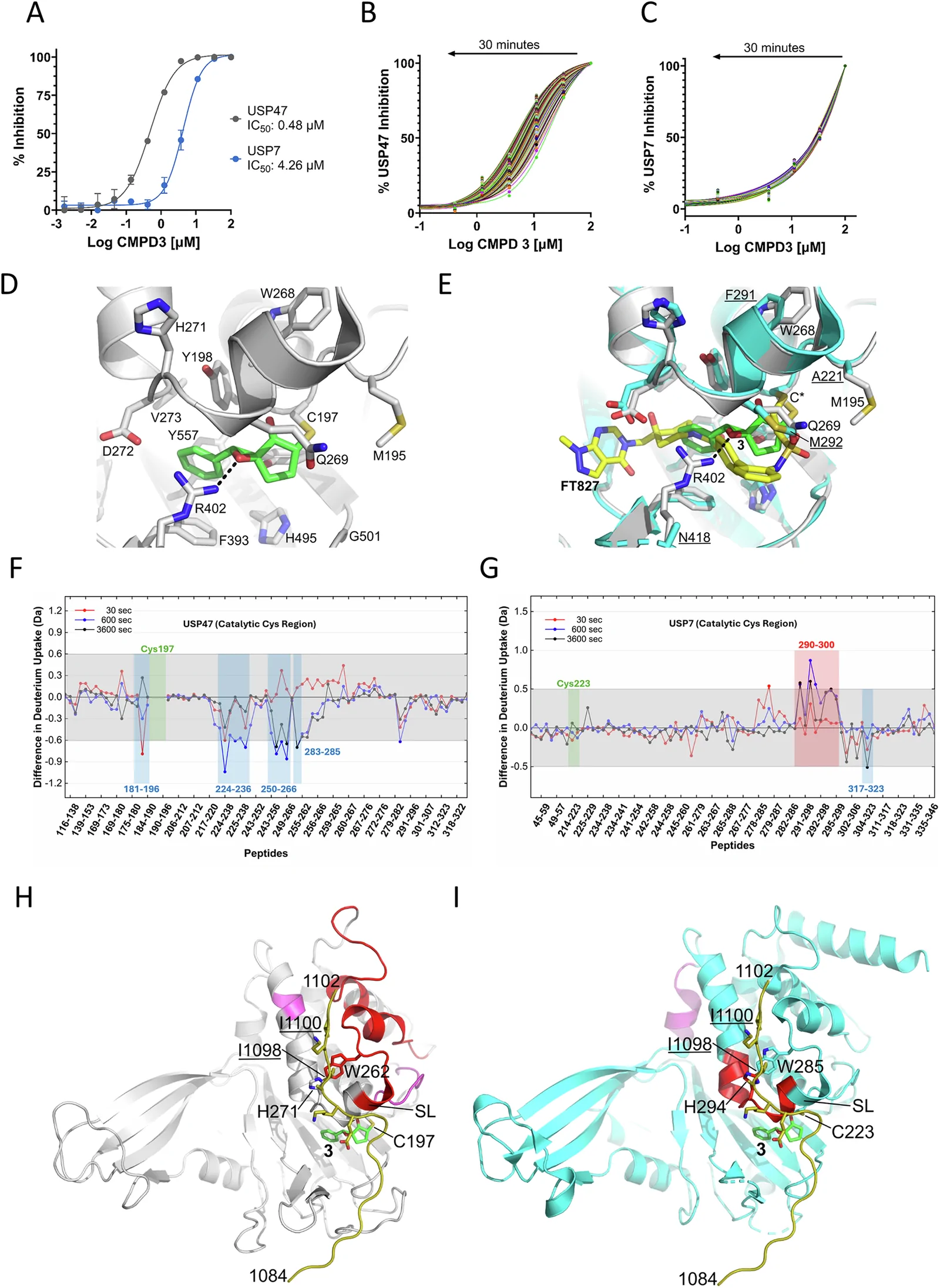
Molecular basis for selective inhibition of USP47 by Compound 3. **A** Inhibition of purified USP47 and USP7 determined from Ub-Rho cleavage after a 30 min pre-incubation with varying concentrations of **Compound 3**. IC_50_ curves fit to: *Y* = Bottom + (Top-Bottom)/(1 + 10^((LogIC_50_-X)*HillSlope)) (Error bars = SD). **B** Inhibition of USP47 with **Compound 3** without preincubation, determined from Ub-Rho cleavage. Each curve represents inhibition data at an individual incubation time from 18 to 1782 s. **C** Inhibition of USP7 with **Compound 3** without pre-incubation, determined from Ub-Rho cleavage. Each curve represents inhibition data at an individual incubation time from 18 to 1782 s. **D** Modeled structure of human USP47 in complex with **Compound 3**, shown as a stick representation with carbon atoms colored green. Residues within 5 Å of the **Compound 3**-C197 adduct are highlighted, and the key hydrogen-bonding interaction between the epoxide moiety of **Compound 3** and the side chain of R402 is represented as a dotted line. Figure prepared using PyMOL (The PyMOL Molecular Graphics System, version 2.5.8; Schrödinger, LLC). **E** Superposition of the modeled structure of human USP47 (gray) in complex with **Compound 3** (green carbon atoms) on the X-ray structure of human USP7 (cyan) in complex with the covalent inhibitor, FT827 (yellow carbon atoms; PDB: 5NGF). C* denotes the catalytic cysteine (corresponding to C197 in human USP47 and C223 in human USP7). **Compound 3** is predicted to bind within the thumb-palm cleft in a similar fashion to FT827, with FT827 extending out further towards the fingers subdomain. Sequence differences are labeled for human USP47 and the corresponding residues are labeled and underlined for human USP7. R402 in USP47 is predicted to hydrogen bond directly to **Compound 3** (represented as a dotted line). R402 in USP47 corresponds to N418 (disordered side chain in PDB: 5NGF) in USP7 with a shorter side chain that cannot interact with the compound, which may explain the higher potency of **Compound 3** for USP47. Residual ΔHDX plots showing differences in deuterium uptake between holo- and apo- states for USP47 (**F**) and USP7 (**G**), across all indicated peptide fragments and time points (30 s to 1 h). These zoomed-in views highlight the peptides surrounding only those regions of the full-length protein (residues 116-323 of USP47 (**F**) and 45–347 of USP7 (**G**)). These regions also encompass the catalytic cysteine residue of each DUB. Positive ΔHDX values indicate increased solvent exposure or flexibility in the presence of **Compound 3**, whilst negative values suggest protection or structural stabilization. A gray shaded area marks the significance threshold for ΔHDX applied in each study. The green boxes indicate those peptides which encompass the catalytic cysteine. Due to the disappearance of these peptides in the holo-state of USP47, these peptides could not be visualized on the plot. This is further evidence of the covalent attachment of **Compound 3** to the catalytic cysteine in USP47. **H** Modeled structure of human USP47 catalytic domain (gray) in complex with **Compound 3** (carbon atoms in green) highlighting regions identified in the HDX-MS analysis colored red (mean perturbation over 60 min of 5%–20%) and magenta (mean perturbation over 60 min of <60%). The 19-residue C-terminal activation peptide of human USP7 (PDB: 5JTV; corresponding to residues 1084 to 1102) is shown in gold with key residues labeled and underlined. The activation peptide of USP47 (corresponding to residues 1345 to 1362)^44^ is proposed to play an important role in activation and regulation analogous to the activation peptide of USP7, which maintains the switching loop (SL; especially W285 and H294) in the active “out” conformation^45^. W285 and H294 in human USP7 correspond to W262 and H271 in human USP47. Regions identified in the USP47 HDX-MS analysis correlate with a conformational shift in the SL and potential engagement of the activation peptide upon **Compound 3** binding. **I** Human USP7 catalytic domain (cyan; from PDB: 5NGF) with the superimposed modeled position of **Compound 3** in human USP47 (green carbons) highlighting regions identified in the HDX-MS analysis in red (mean perturbation over 60 min of 5–20%) and magenta (mean perturbation over 60 min of <60%). The 19-residue C-terminal activation peptide of human USP7 (PDB: 5JTV; corresponding to residues 1084 to 1102) is in gold with key residues labeled and underlined. Analysis of human USP7 HDX-MS data reveals that the region of highest confidence directly flanks the proposed compound binding site, and differences are not observed in structural elements implicated in activation peptide binding, suggesting that **Compound 3** does not trigger an equivalent shift in SL to the active “out” conformation and subsequent binding of the activation peptide as predicted for USP47.

Given the preferential activity of **Compound 3** for USP47 over USP7, we investigated its mode of action. The results of in vitro activity assays using recombinant DUBs and a fluorogenic ubiquitin-rhodamine (Ub-Rho) substrate revealed that **Compound 3** indeed inhibited DUB activity. Interestingly, **Compound 3** was ~10-fold more potent in inhibiting USP47 than USP7 after 30 min preincubation (IC₅₀: ~0.48 vs. ~4.3 μM; Figs. 3A and S4A–D). Time-dependency experiments indicated covalent or slow tight-binding inhibition for USP47 (Fig. 3B), while USP7 inhibition was weaker and time-independent (Fig. 3C). For both enzymes, progress curves in the presence of **Compound 3** were non-linear, indicating slow initial binding (Fig. S4E, F).

Molecular docking studies were initially performed to investigate the preferred reaction of **Compound 3** with USP47. In the absence of a human USP47 Protein Data Bank (PDB) structure, the structure of human USP7 in complex with the covalent inhibitor FT827 (PDB: 5NGF) was used as the template to generate a homology model of human USP47 with an appropriate conformational state for covalent inhibitor docking studies. **Compound 3** most likely forms a covalent adduct with the catalytic cysteine of human USP47 (C197) via 1,2-carbonyl attack and retro-aldol fragmentation based on previously reported work (Fig. S4G)^27^.

The docking studies also suggest that **Compound 3** covalently binds at the thumb-palm cleft (Fig. S4H) and sterically blocks Ub binding (Fig. S4I), analogous to the covalent USP7 inhibitor FT827 (PDB: 5NGF; Fig. S4J). The binding site is flanked by 14 residues including R402, the side chain of which is predicted to form a hydrogen bond with the epoxide moiety of **Compound 3** (Fig. 3D). In human USP7, the substitution of R402 with an asparagine residue (N418), which cannot interact with **Compound 3**, may rationalize the apparent selectivity of **Compound 3** for USP47 inhibition (Fig. 3E).

USP47 apo-form (PDB: 8ITN) and Ub-bound (PDB: 8ITP) structures are reported for the closely related catalytic domain of *C. elegans* USP47 (44% sequence identity). Comparison of the human USP47-**Compound 3** model with the apo *C. elegans* USP47 structure suggests that **Compound 3** binding disrupts the hydrogen bond between R402 and H271 that stabilizes the switching loop in its catalytically incompetent “in” conformation. Disruption of this interaction is predicted to favor the active “out” conformation of the switching loop, compatible with activation peptide binding, as described for USP7 (Fig. S4O, P)^44,45^.

Hydrogen-deuterium exchange mass spectrometry (HDX-MS) studies were performed to investigate the binding of **Compound 3** to USP47 and USP7 (Figs. 3F, G and S4K, L). The conformational shifts observed by HDX-MS indicate a switching loop rearrangement in USP47 (Fig. S4M) and potential engagement of the activation peptide (Fig. 3H)^45^. Notably, HDX-MS analysis with USP7 showed no such distal conformational changes, indicating that **Compound 3** does not promote an equivalent switching loop reorganization or activation peptide binding in USP7 (Figs. 3I and S4N), further rationalizing its selectivity for USP47.

### Inhibition of USP47 with compound 3 further supports its roles in inflammasome activation

**Fig. 4:**
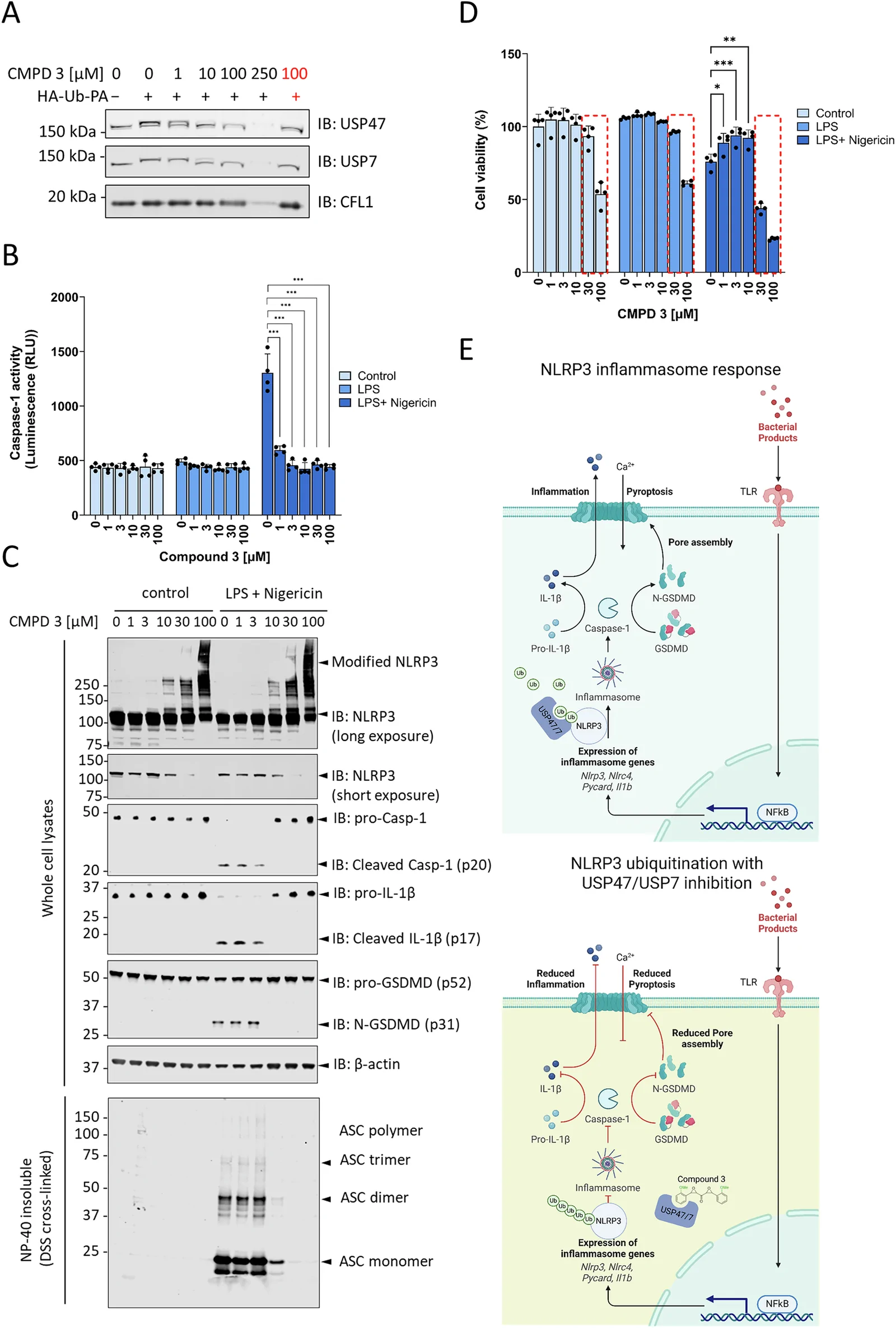
Inhibition of USP47 with Compound 3 further supports its roles in inflammasome activation. **A** Cell permeability concentration-dependence of **Compound 3** after incubation with MCF-7 cells for 4 h, lysis and subsequent labeling with HA-Ub-PA. Red = lysate control (**B**) Caspase-1 activity in the media (supernatant) of PMA-differentiated THP-1 cells treated with indicated concentrations of **Compound 3**, determined using Caspase-Glo 1 luminescence assay. **Compound 3** incubation for 4 h, ±1 µg/mL LPS incubation for 4 h and 10 µM Nigericin incubation for 45 min (error bars = SD). One-way ANOVA was used. ***P < 0.001. **C** Immunoblots of NLRP3, Caspase-1 (Casp-1), and gasdermin d (GSDMD) from whole cell lysates of PMA-differentiated THP-1 cells treated with indicated concentrations of **Compound 3**. Immunoblot of ASC oligomerisation of the NP40-insoluble fraction of PMA-differentiated THP-1 cells after treatment with the indicated concentrations of **Compound 3** and DSS cross-linking. **Compound 3** incubation for 4 h, ± 1 µg/mL LPS incubation for 4 h and 10 µM Nigericin incubation for 45 min. **D** MTS cell viability of THP-1 cells treated with indicated concentrations of **Compound 3** ± LPS or LPS/Nigericin. Compound treatment for 4 h, 1 µg/mL LPS treatment for 4 h, 10 µM Nigericin treatment for 2 h (error bars = SD). Red dashed boxes indicate cell death. Two-way ANOVA was used, discounting data in red dashed boxes, with Tukey multiple comparisons correction. *P < 0.0332, **P < 0.0021, ***P < 0.0002. **E** Formation of the NLRP3 inflammasome complex upon stimulation with bacterial products such as LPS and nigericin, with USP47 / USP7 deubiquitinating NLRP3, and the ubiquitination of NLRP3 upon USP47 / USP7 inhibition with **Compound 3** preventing the formation of the NLRP3 inflammasome. Created in BioRender. Jones, H. (2025) (License link to be inserted upon publication).

ABP assays in MCF-7 cells revealed that Compound 3 is cell-permeable and reduced USP47 probe labeling in a concentration-dependent manner (Fig. 4A); notably, **Compound 3** reduced USP47/USP7 probe labeling at concentrations comparable to those required to inhibit catalysis by purified USP47/USP7 (Fig. 3A). Cytotoxicity was observed at 250 µM, indicative of off-target effects, in line with the reported reactivity of DEKs with other enzymes. In addition, **Compound 3** exhibits limited stability in glutathione-containing buffer, potentially limiting its utility as a tool compound (Fig. S15). We next investigated the ability of **Compound 3** to replicate reports describing a role of both USP47 and USP7 in the activation of the NLRP3 inflammasome by regulating NLRP3 ubiquitination^42^.

Human monocyte (THP-1) cells were treated with lipopolysaccharide (LPS) followed by a 45 min or 2 h nigericin incubation to activate the NLRP3 inflammasome. Under these conditions, treatment with **Compound 3** inhibited inflammasome activation, as evidenced by reduced Caspase-1 activity (Figs. 4B and S5A–C). To ensure **Compound 3** did not reduce Caspase-1 activity via direct inhibition, the upstream formation of the NLRP3 inflammasome was also examined: THP-1 treatment with **Compound 3**, in combination with LPS and Nigericin, blocked the oligomerisation of apoptosis-associated speck-like protein containing a CARD (ASC), and prevented the cleavage of Caspase-1, IL-1β, and gasdermin D (GSDMD) (Fig. 4C). While a decrease in NLRP3 levels was observed, the treatment of cells with **Compound 3** resulted in appearance of a high molecular weight NLRP3 smear, consistent with ubiquitination (Fig. 4C). While further investigation is required to fully characterize this smear, it may be attributable to the ubiquitination of NLRP3, as previously proposed^42^.

No decrease in THP-1 cell viability was detected when incubating with up to 100 µM **Compound 3**, when the LPS treatment was followed by 45 min nigericin treatment (Fig. S5D). In contrast, 2 h of nigericin exposure induced toxicity at **Compound 3** concentrations of 30 µM and higher (Fig. 4D), consistent with additional targets beyond DUBs at these high concentrations^27^. In contrast, and consistent with an on-target reduction in inflammasome activation, lower concentrations of **Compound 3** increased THP-1 viability upon LPS and a 4 h nigericin treatment, indicating reduced inflammasome-mediated pyroptosis (Fig. 4D). Taken together, the data demonstrates that **Compound 3** can reproduce phenotypes previously seen by USP47/7 gene knock-out (KO) and inhibition, through the prevention of NLRP3 inflammasome formation (Fig. 4E).

### Inhibition of OTUD7B with compound 6 further supports its roles in hypoxic signaling and associated SARS-CoV-2 repression

**Fig. 5:**
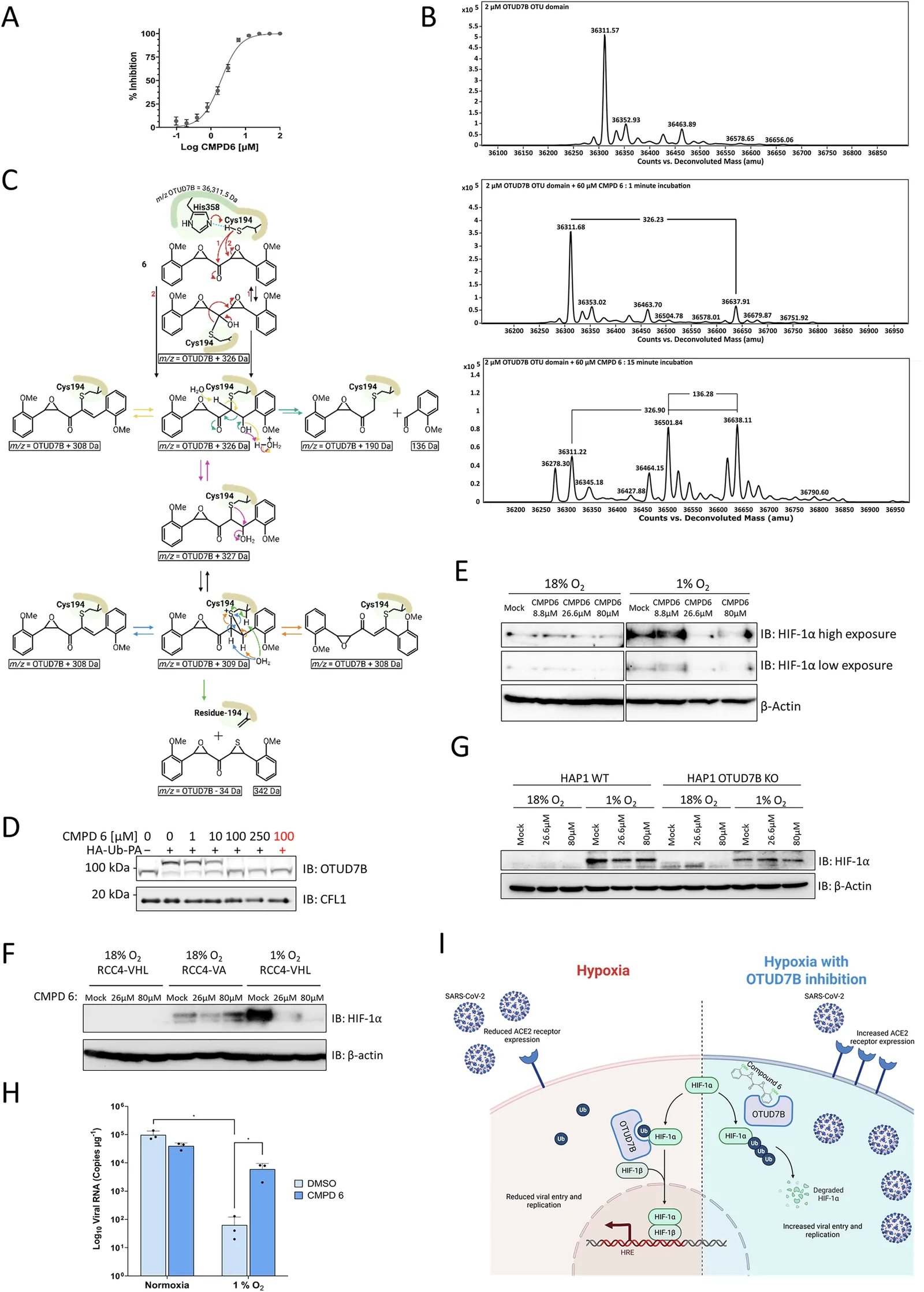
Inhibition of OTUD7B with Compound 6 further supports its roles in hypoxic signaling and associated SARS-CoV-2 repression. **A** Inhibition of purified OTU domain of OTUD7B determined from Ub-Rho cleavage after a 30 min pre-incubation with varying concentrations of **Compound 6**. IC_50_ curves fit to: *Y* = 100/(1 + 10^((LogIC50-X)*HillSlope)) (Error bars = SD, *N* = 3). **B** LC-MS (liquid chromatography-mass spectrometry) analysis of the reaction of OTUD7B (top spectrum) following 1 min (central spectrum) and 15 min (bottom spectrum) incubation with **Compound 6**. **C** Mechanism for the covalent reaction of OTUD7B with **Compound 6**. **D** Cell permeability concentration-dependence of **Compound 6** after incubation with MCF-7 cells for 4 h, lysis and subsequent labeling with HA-Ub-PA. Red = Lysate control (**E**) Immunoblot of HIF-1α in Calu-3 cells incubated in 18% or 1% O_2_ for 24 h followed by treatment with increasing doses of **Compound 6** for 1 h. **F** Expression of HIF-1α in RCC4-VA (Mutant VHL) or –VHL (WT VHL) cells treated with increasing doses of **Compound 6**. Lysates from hypoxic RCC4-VHL cells treated with **Compound 6** were also performed to induce HIF-1α expression. **G** HAP1-WT and OTUD7B KO cells were incubated in 18% or 1% O_2_ for 24 h followed by treatment with indicated doses of **Compound 6** for 1 h. HIF-1α expression was then determined by immunoblot in WT and KO cells. Representative immunoblots from *n* = 2 independent biological experiments. **H** Calu-3 cells were infected with SARS-CoV-2 (VIC02/20) at a multiplicity of infection (MOI) of 0.01 for 1 h. The inoculum was removed and cells incubated at 18% or 1% O_2_ for 24 h treated with or without 26.6 µM of **Compound 6**. Viral replication was quantified by qPCR of intracellular viral RNA and expressed as copies µg-1 of total cellular RNA. Data is representative of three biological replicates and data presented as mean −/+ S.D. Significance from Mann-Whitney test, * = *p* < 0.0332. **I** Under hypoxic conditions, HIF1α expression is increased, leading to reduced ACE2 receptor expression and subsequent reduced SARS-CoV-2 viral entry and replication^48^. Inhibition of OTUD7B by **Compound 6** leads to increased ubiquitination, and subsequently decreased levels, of HIF1α under hypoxic conditions. This results in increased ACE2 receptor expression, allowing for increased SARS-CoV-2 viral entry and replication. Created in BioRender. Jones, H. (2025) (License link to be inserted upon publication).

Orthogonal in vitro assays using the recombinant catalytic domain of OTUD7B and a ubiquitin–rhodamine substrate demonstrated that **Compound 6** inhibits recombinant OTUD7B with an IC₅₀ of ~1.9 µM (95% confidence interval, 1.8–2.1 µM) (Fig. 5A). Intact protein mass spectrometry revealed that incubation of the catalytic domain of OTUD7B with **Compound 6** (molecular weight: 326.35 Da) for 1 min results in a + 326 Da mass shift, consistent with covalent reaction of OTUD7B with **Compound 6** in a 1:1 stoichiometry. After 15 min, the formation of multiple species derived from the +326 Da adduct were observed with a +190 Da adduct dominating. The +190 Da adduct likely arises from elimination of *ortho*-methoxybenzaldehyde from the initially formed +326 Da intermediate, consistent with the crystallographically validated mechanism by which nucleophilic cysteine enzymes react with DEKs (Fig. 5B, C)^27^. Although these data clearly support covalent modification of OTUD7B by **Compound 6**, the precise modified residue was not assigned in this study.

**Compound 6** was active and cell-permeable in MCF-7 cells (Fig. 5D), enabling subsequent evaluation of the phenotypic consequences of OTUD7B inhibition. OTUD7B has been shown to stabilize hypoxia-inducible factor 1α (HIF-1α) under hypoxic conditions in a ubiquitin-dependent manner, counteracting the E3 ubiquitin ligase activity of the von Hippel–Lindau tumor suppressor (VHL)^46^. Consistent with these findings, treatment of Calu-3 cells with **Compound 6** under hypoxic conditions reduced HIF-1α levels in a dose-dependent manner (Fig. 5E). Furthermore, in renal carcinoma cells lacking active VHL (RCC4-VA)^47^, we observed that treatment with **Compound 6** had no impact on HIF-1α protein levels. When wild-type VHL (RCC4-VHL) expression was restored, however, a dose-dependent reduction in hypoxic HIF-1α protein levels was observed (Fig. 5F). In Chronic Myeloid Leukemia (CML)-derived HAP1 wild-type (WT) cells, treatment with **Compound 6** also resulted in a dose-dependent reduction in HIF-1α protein levels. Importantly, HIF-1α levels were markedly reduced in HAP1 OTUD7B KO cells, consistent with published findings^46^, and treatment of the KO cells with **Compound 6** did not further reduce HIF-1α, supporting an on-target mechanism at the tested concentrations (Fig. 5G).

Previous studies have shown that SARS-CoV-2 replication is reduced under hypoxic conditions, attributable to increased HIF-1α protein levels^48^. Consistent with these findings, we observed decreased viral replication in Calu-3 cells under hypoxia compared with normoxia. Notably, treatment with **Compound 6** significantly attenuated the antiviral effect of hypoxia (Fig. 5H). These results are consistent with reduced HIF-1α protein levels following OTUD7B inhibition. The ability of **Compound 6** to phenocopy OTUD7B knockout supports on-target engagement and provides further evidence that OTUD7B catalytic activity directly contributes to HIF-1α stability under hypoxic conditions. These data provide proof-of-concept pharmacological evidence that OTUD7B inhibition can modulate hypoxia-associated changes in SARS-CoV-2 replication, consistent with effects on HIF-1α protein levels (Fig. 5I).

### Inhibition of USP5 demonstrates its essential role in SARS-CoV-2 replication

**Fig. 6:**
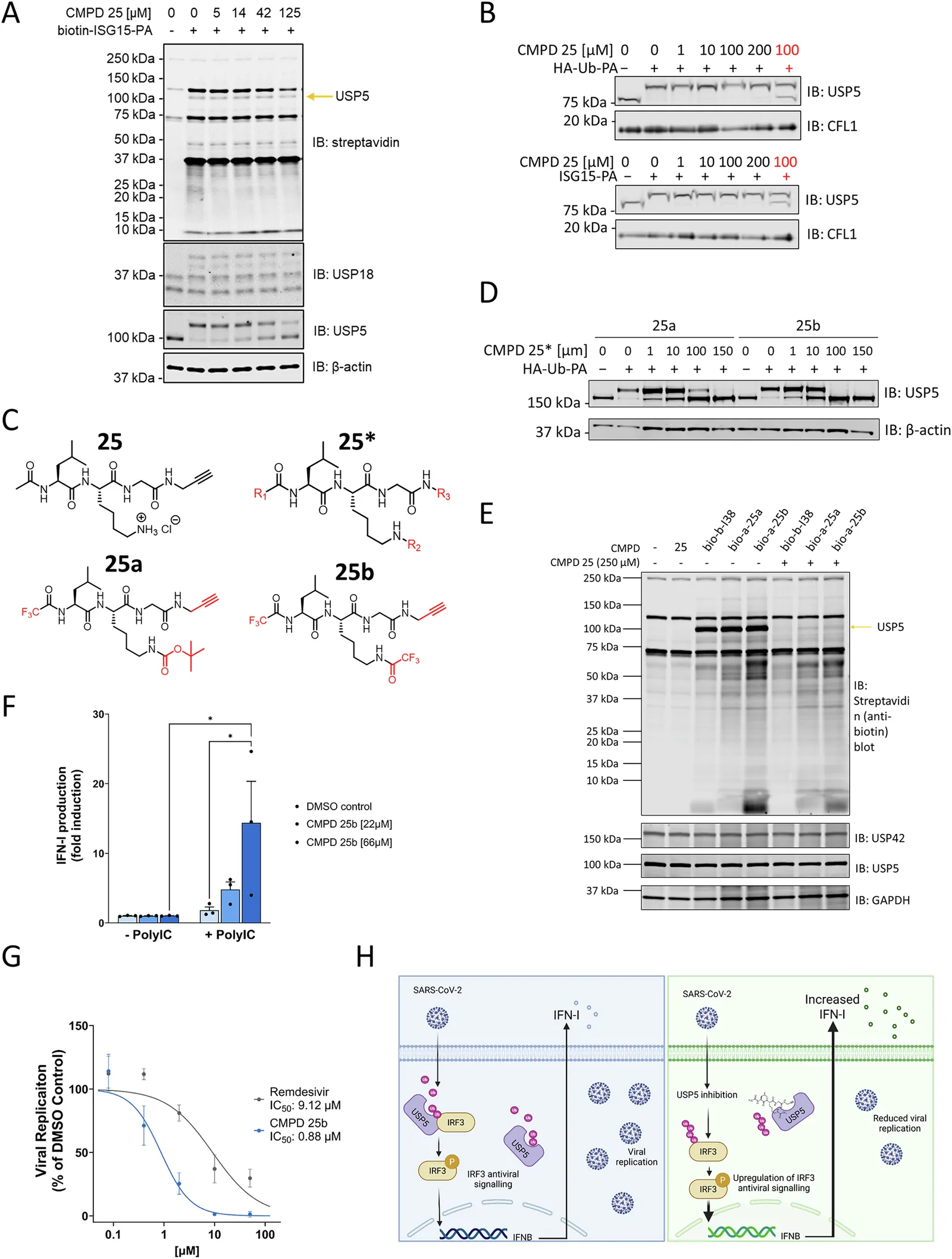
Inhibition of USP5 with Compound 25 demonstrates its essential role in SARS-CoV-2 replication. **A** **Compound 25** concentration dependence bio-ISG15-PA ABP with USP5 and USP18 in lysates from HAP1 cells treated with 1000 U/mL of human interferon α 2 (α2b) for 24 h. **B** Cell permeability concentration-dependence of **Compound 25** after incubation with MCF-7 cells for 4 h, lysis and subsequent labeling with HA-Ub-PA or ISG15-PA. Red = Lysate control. **C** Varying the R1 and R2 groups of **Compound 25** enhances cellular permeability. **D** Cellular permeability concentration-dependence of USP5 probe labeling reduction with **Compounds 25a** and **25b** after incubation with MCF-7 cells for 4 h, lysis and subsequent labeling with HA-Ub-PA. **E** MCF-7 lysates were incubated with DMSO or 250 µM of **Compound 25** for 1 h, followed by incubation with 150 µM of biotinylated derivatives of **Compound 25** for 1 h. **F** Interferon I (IFN-I) production by HAP1 cells treated with **Compound 25b**/DMSO for 1 h, followed by poly(I:C) (0.5 µg/mL) for 48 h. HEK293 IFN reporter cells were incubated with HAP1 media for 24 h, and luminescence determined with the One-Glo luciferase assay system. Data are representative of three replicates with data presented as mean −/+ SEM. Significance from 2-way ANOVA, with Tukey’s multiple comparisons test, * = *p* < 0.0332 (**G**) Calu-3 cells were infected with SARS-CoV-2-mNeonGreen reporter virus at a MOI of 0.1 and treated with a range of doses of **Compound 25b** or remdesivir^94^. Data is representative of three biological replicates and data presented as mean −/+ SEM. Dose response curves were determined using a non-linear regression analysis to calculate IC_50_ values for each compound. **H** Upon SARS-CoV-2 infection, ubiquitinated IRF3 is activated leading to antiviral signaling, thanks to upregulation of IFN-I production. USP5 deubiquitinates IRF3^54^, and upon SARS-CoV-2 infection, inhibition of USP5 with **Compound 25b** leads to an increase in IRF3 ubiquitination, resulting in an upregulation of IRF-3-mediated IFN-I production, and subsequent reduced viral replication. Created in BioRender. Jones, H. (2025) (License link to be inserted upon publication).

The active sites of DUBs and deISGylases, cellular enzymes catalyzing the cleavage of the ubiquitin-like protein ISG15 (interferon-stimulated gene 15) from modified proteins, are structurally very similar. Most deISGylating enzymes are cross-reactive DUBs, catalyzing the removal of both Ub and ISG15, with the notable exception of USP18^43,49–51^. Given USP5’s dual Ub/ISG15 specificity, we evaluated the effect of **Compound 25** on deISGylases by immunoblotting, using a bio-ISG15-PA ABP. The results reveal that the extent of USP5 probe labeling reduction by **Compound 25** is comparable using the the bio-ISG15-PA and Ub-PA ABPs, and that **Compound 25** did not cross-react with USP18 or any of the other detected deISGylases in the tested concentration range (up to 125 μM), demonstrating the high selectivity of **Compound 25** for targeting USP5 (Fig. 6A).

Interestingly, ABP assays in MCF-7 cells revealed that **Compound 25** was inactive in intact cells, limiting its applicability (Fig. 6B). To optimize **Compound 25** for cellular activity, we modified the alkyne and amine protecting groups. Substituting the terminal alkyne reduced potency (Fig. 6C; Supplementary Data S4; synthesis detailed in SI), supporting that **Compound 25** covalently reacts with the USP5 nucleophilic cysteine via its alkyne group, which serves as a latent electrophile. By contrast, altering amine-protecting groups at the N-terminus (R_1_) and/or the *N*^ε^-lysine amino group (R_2_) by incorporating fluorine atoms to likely increase cell permeability^52,53^, we generated the active and cell-permeable derivatives **Compounds 25a** and **25b** bearing trifluoromethyl groups at R1 and trifluoroacetyl (**25a**) or *tert*-butyloxycarbonyl (Boc; **25b**) at R2, respectively (Fig. 6C, D). Both **25a** and **25b** retained the potency and selectivity for USP5 probe labeling reduction observed in lysates (Fig. S6A) and, like **Compound 25**, were stable for >22 h in glutathione-containing buffer (Fig. S16–19), thus offering promising leads for functional studies and possibly therapeutic development.

To investigate the binding and selectivity of **Compound 25** and its derivatives to USP5, we generated five biotinylated derivatives with different linker strategies, including biotinylation via the N-terminal amino group or the *N*^ε^-lysine amino group (Supplementary Data S5). All five biotinylated versions of **Compound 25** bound to endogenous USP5 in MCF-7 lysates. Binding was confirmed by streptavidin western-blotting, with a strong band at the expected molecular weight of USP5 (~95 kDa; Figs. 6E and S7B, D). By contrast, no signal was observed for another USP, USP42, at ~150 kDa. Interestingly, despite comparable binding to USP5, derivatives **bio-b-25a** and **bio-a-25b** were less active than the other three biotinylated derivatives, as shown by Ub-PA probe competition assays, at two different concentrations (Fig. S7A, B), an observation likely reflecting the substantial changes in, e.g., structure and physicochemical properties associated with incorporation of biotin. Selective binding of these **Compound 25** derivatives to the catalytic cysteine of USP5 was supported by competition experiments using free **Compound 25** and *N*-ethylmaleimide (**NEM**), a broad-spectrum cysteine-reactive alkylating agent. In both cases, competition resulted in loss of the USP5 band at ~95 kDa, consistent with covalent engagement of the nucleophilic cysteine. (Figs. S7C, 6E, S7D). Together, these data are consistent with covalent engagement of the USP5 nucleophilic cysteine, although the precise site of modification was not assigned in this study.

To functionally validate **Compound 25b** as an on-target inhibitor of USP5, we investigated its effect on viral replication. USP5 has been linked to IFN-I production by regulating the ubiquitination of different innate immune factors such as RIG-I and IRF3^54,55^. In agreement with these observations, when IFN-I production was induced by synthetic RNA Poly I:C, there was a significant amplification of IFN-I production in HAP1 cells co-treated with **Compound 25b** (Fig. 6F). In line with the observed elevated IFN-I levels, **Compound 25b** exhibited a dose-dependent reduction in viral replication against SARS-CoV-2 in infected Calu-3 cells. Remdesivir, a guanosine nucleoside analog that targets the viral replicase complex and is in clinical use as an antiviral medication^56^, was included as a direct-acting antiviral benchmark. In this assay, the host-directed USP5 inhibitor **Compound 25b** reduced SARS-CoV-2 replication with an apparent IC_50_ ~ 10-fold lower than that of remdesivir (Fig. 6G), without significant cellular toxicity at concentrations as high as 200 µM in HAP1 cells (Fig. S8A). Furthermore, **Compound 25b** also showed antiviral activity against influenza A virus and encephalomyocarditis virus, with IC_50_ values of ~1.9 µM and ~5.1 µM, respectively (Fig. S14). The increased IFN-I production and inhibition of viral replication by Compound 25b validate it as a tool compound for investigating USP5 function in signaling and provide evidence for USP5 as a potential therapeutic target (Fig. 6H).

## Discussion

As interest in DUBs as drug targets grows^6,57,58^, so does the need for improved discovery strategies. High-throughput screening (HTS) has enabled rapid evaluation of large compound libraries^39,59^, yet it remains constrained by high cost, low hit rates, and reliance on recombinant proteins that may not fully capture native regulation and complex formation. Consequently, many HTS-derived hits require substantial optimization and secondary validation before demonstrating cellular activity.

To overcome these limitations, computational approaches and activity-based proteomics have emerged as complementary strategies. AI-enabled virtual screening platforms such as AtomNet and Boltz-2 prioritize compounds in silico, even in the absence of prior ligand data^60^. In parallel, chemoproteomic profiling of reactive cysteines has enabled more directed targeting of cysteine-containing proteins, including DUBs^61–64^. Recent advances in DUB-focused ABPP have produced high-throughput-ready platforms, including our ABPP-HT* workflow^19,24,65^. ABPP-HT* combines sensitivity, cost-efficiency, and flexibility, operating in both manual and automated formats while maintaining compatibility with diverse probe chemistries and enzyme classes.

Here, we demonstrate a proof-of-concept application of the ABPP-HT* platform for DUB binder and inhibitor discovery and characterization. Screening a curated electrophile-containing library, including αβ,α′β′-diepoxyketones (DEKs), electronically activated γ-lactams, and ubiquitin-derived peptides specifically designed for this study, yielded a hit rate ~10-fold higher than traditional DUB HTS approaches. Following validation, we identified selective binders/inhibitors of USP47, OTUD7B, and USP5 and further characterized their potency, permeability, selectivity, and functional consequences. Of note, the identification of false-positive and cross-linking compounds underscores the importance of secondary validation, including orthogonal ABP immunoblotting and concentration-dependent profiling, following primary ABPP-HT* screening. These findings highlight the efficiency of endogenous chemoproteomic screening for rapid identification of cell-active DUB inhibitors.

USP47 represents an emerging therapeutic target^66–68^, yet previously reported inhibitors display comparable potency toward its close homolog USP7^40,41^, consistent with their substantial sequence similarity^42,43^. We identified **Compound 3** as a first-in-class inhibitor exhibiting a biochemical preference for USP47 over USP7. Orthogonal kinetic analyses and HDX-MS-guided structural modeling provided insight into a possible structural basis for this preference (Figs. 3 and S4). Importantly, **Compound 3** recapitulated the reported effects of USP47/USP7 inhibition on inflammasome activation, supporting pharmacological modulation of the USP47/USP7 axis^42^.

Although the roles of OTUD7B in disease are still being defined^69^, accumulating evidence supports its therapeutic potential^46,70–72^, and recent reports describe emerging OTUD7B-directed ligands^65,73^. We identified **Compound 6** as a potent, selective, and cell-permeable OTUD7B inhibitor that reduced HIF-1α protein levels under hypoxia, phenocopying OTUD7B knockdown^46^. Given that hypoxia restricts SARS-CoV-2 infection in a HIF-1α-dependent manner^48,74^, **Compound 6** correspondingly rescued viral replication under hypoxic conditions^74^. These findings establish inhibition of OTUD7B as a tool to interrogate hypoxia-driven cellular responses.

**Compound 25** displayed high selectivity for USP5 over related DUBs such as USP16 and USP22^75^. USP5 hydrolyzes free polyubiquitin chains via a zinc finger ubiquitin-binding domain (ZnF UBP) that recognizes the C-terminal diglycine motif of ubiquitin^76^. Although USP5 has been implicated in cancer, pain, and inflammatory disorders^77^, existing inhibitors remain limited in selectivity, scope, and cellular validation (Fig. S10)^78,79^. In contrast to prior inhibitors designed to engage the ZnF UBP carboxylate-binding pocket^78,80^, our ubiquitin-derived molecules substitute the C-terminal glycine with a propargyl amide group, inspired by the covalent reactivity of Ub-PA probes. Remarkably, these relatively simple substrate-inspired molecules achieved high selectivity, highlighting subtle but functionally significant differences in active-site architecture across DUBs.

Mechanistically, **Compound 25** likely modifies the catalytic cysteine of USP5 via its alkyne warhead, supported by structural precedent (PDB: 3IHP)^81^, related USP-Ub-PA complexes, and reduced activity upon steric modification (Supplementary Data S4). USP5’s unique domain architecture, including a disulfide linkage between C793 and C195 in the USP5-Ub complex, may constrain blocking loop flexibility and promote favorable compound engagement (Fig. S8B). The use of a latent electrophile may further enhance selectivity through differential binding kinetics across DUBs^33,38,82,83^; alkynes also manifest high stability towards reaction with abundant nucleophiles such as glutathione (Fig. S16–S19).

Structure-activity optimization yielded the fluorinated, cell-permeable **Compounds 25a** and **25b** that retained cellular activity and were suitable for functional studies. Biotinylated derivatives confirmed covalent engagement and selective binding to USP5 in our chemoproteomic assays. Notably, **Compound 25b** enhanced type I interferon production and exhibited strong antiviral activity (Figs. 6 and S14), extending established USP5 functions.

The combined results manifest the potential of high-throughput ABPP workflows for identifying DUB inhibitors and streamlining the development of potent and, at least, partially selective compounds for use as biochemical tools in functional assignment and mechanistic studies. Importantly, **Compounds 3** and **6**, as well as **Compounds 25a** and **25b**, are novel classes of active site-targeting covalently reacting DUB inhibitors that show promise for the development of chemical probes and drugs. Further optimization of these scaffolds, including broader cysteine-proteome profiling, will be important to advance them toward high-quality chemical probes and drug-like inhibitors. By eliciting pathway-relevant phenotypes in innate immunity, hypoxic signaling, and viral infection models, these inhibitors further reinforce the therapeutic potential of DUBs.

## Methods

### Small-molecule synthesis

αβ,α′β′-Diepoxyketones (DEKs) (**Compounds 1-8**)^27^ and electronically activated γ-lactams (**Compounds 9–23**)^28^  were reported. The synthesis of Ub-derived small-molecules (**25**-**35 and 25a-k**) is described in the Supplementary Information (Figs. S11 and S12). The structures of **Compounds 1-31** are in the Supplementary Information (Supplementary Data S1-3), the structures of **Compounds 25a-k** are in the Supplementary Data S4. The synthesis and structures of biotinylated derivatives of **Compound 25** are described in the Supplementary Information (Fig. S13 and Supplementary Data S5).

### Cell culture

MCF-7 cells were cultured in high glucose DMEM supplemented with 10% (v/v) fetal bovine serum (FBS) and L-glutamine (2 mM). Cells were maintained at 37 °C, 5% CO_2_. MCF-7 cells were washed with phosphate buffered saline (PBS) and collected in PBS by scraping and centrifugation at 200 × *g* for 10 min. For data shown in Fig. S1A–C, MCF-7 cells were collected by the addition of TrypLE followed by media and centrifuged at 1600 rpm for 5 min with pellets being resuspended in PBS (3 cycles). HAP1 cells were cultured in IMDM media (#21980065) supplemented with 10% FBS (v/v) and maintained at 37 °C, 5% CO_2_. Approximately 3 × 10^6^ cells were seeded per 10 cm plate in 10 mL media overnight. After seeding, 4 mL of media were aspirated from each plate, and recombinant human interferon-alpha, IFN-α2 (α2b; PBL Assay Science; Cat. No. 11105-1), was added to the remaining media (1000 units/mL final concentration). IFN-α treatment was performed for 24 h (to induce USP18), media from each plate were then aspirated, and cells were washed once with PBS (5 mL per plate). Cells were subsequently stored at –80 °C until lysis. THP-1 cells were cultured in RPMI 1640 supplemented with 10% (v/v) FBS, 0.05 mM β-mercaptoethanol, GlutaMAX Supplement, and Penicillin-Streptomycin. Cells were maintained at 37 °C, 5% CO_2_. RCC4-VHL and VA cells were cultured in DMEM media supplemented with 10% fetal bovine serum (FBS), GlutaMAX, and Penicillin-Streptomycin^47^. Calu-3 cells were maintained in Advanced DMEM media, 10% FCS, L-glutamine, and Penicillin-Streptomycin.

### High-throughput ABPP workflow: sample preparation for Agilent Bravo ABPP-HT*

The data presented here on the Agilent Bravo ABPP immunoprecipitation is from published work^24^, and is included for comparison.

### High-throughput ABPP workflow: sample preparation for “on bench” ABPP-HT* high-throughput screen and compound concentration-dependence Validations

HA-Ub-PA was synthesized as reported^24,26^. MCF-7 cells were washed with PBS, scraped in PBS and centrifuged at 200 × *g* for 10 min. Pellets were then resuspended in lysis buffer (50 mM Tris, 5 mM MgCl_2_, 0.5 mM EDTA, 250 mM sucrose, 1 mM dithiothreitol (DTT), pH 7.5), and lysed using glass beads (2:1 lysate to beads ratio) with vortexing (10 cycles of 30 s vortexing, followed by 2 min incubation on ice). The MCF-7 lysate was then clarified at 600 × *g* for 10 min at 4 °C. Lysate protein concentration was determined by BCA, and 250 µg was aliquoted per well of a 96-well plate at a concentration of ~4.7 mg/mL.

The compound library was diluted to 1 mM DMSO stock solutions in a 96-well plate and multichannel-pipetted with mixing into the lysate plate to a final compound concentration of 25 µM in duplicate for the screen. For the concentration-dependence validations, the indicated molecule concentrations were added in duplicate. Additional control wells (4 × negative, 4 × positive for the screen, 3 × negative and 3 × positive for the concentration-dependence validations) were treated with DMSO. The plate was then incubated at 37 °C for 1 h on a thermomixer without shaking.

A total of 2 µg of HA-Ub-PA was multichannel-pipetted with mixing into the positive control wells and all molecule-treated wells, with an equivalent volume of buffer added to the negative control wells. The lysates were then incubated with the HA-Ub-PA at 37 °C for 45 min on a thermomixer without shaking. The final reaction volume with the MCF-7 lysate, inhibitor/DMSO and HA-Ub-PA/buffer was 60 µL. Reactions were quenched by the addition of SDS and NP-40 (IGEPAL CA-630) to final concentrations of 0.4% (v/v) and 0.5% (v/v), respectively. Reactions were then diluted by addition of 200 µL NP40 buffer (50 mM Tris, 0.5% NP-40 (IGEPAL CA-630), 150 mM NaCl, 20 mM MgCl_2_·6H_2_O, pH 7.4).

75 µL of anti-HA magnetic beads were aliquoted into a separate 96-well plate and washed 5x with 200 µL of NP40 buffer (50 mM Tris Base, 0.5% NP-40 (IGEPAL CA-630), 150 mM NaCl, 20 mM MgCl_2_·6H_2_O, pH 7.4), using a magnetic rack for bead separation and multichannel pipetting with cut tips for mixing. Reaction mixtures were then added to the washed magnetic beads, mixed by multichannel-pipetting, and incubated in a thermomixer overnight at 4 °C with gentle agitation (350 rpm). The beads were then washed 5x with 200 µL of NP40 buffer, as before.

Proteins were eluted from the beads and reduced by the addition of 100 µL of 2x Laemmli buffer supplemented with 20 mM DTT, and incubated for 10 min at 90 °C with agitation (350 rpm) on a thermomixer. Eluates were then removed from the beads using a magnetic rack and transferred to a fresh 96-well plate. Eluates were alkylated by the addition of iodoacetamide (IAA) to a final concentration of 40 mM. Proteins were then digested using trypsin and cleaned up using a 96-well S-trap plate according to the manufacturer’s instructions^84^, dried down in a speedvac and resuspended in 0.1% (v/v) aqueous formic acid.

### High-throughput ABPP workflow: sample preparation for the Thermo KingFisher apex ABPP-HT*

The bio-ISG15-PA probe was synthesized as previously described^85^. Cell lysis, subsequent labeling with HA-Ub-PA or bio-ISG15-PA probes, immunoprecipitation and protein enrichment, and processing of samples for analysis by western blot and LC-MS/MS were performed as described^86^. Briefly, HAP1 cells were treated and prepared as described in the subsection “*Cell culture*”. Cells were resuspended in lysis buffer (50 mM Tris, 5 mM MgCl_2_, 0.5 mM EDTA, 2.5% (v/v) glycerol, 1 mM dithiothreitol (DTT), pH 7.5), and lysed using glass beads (2:1 ratio lysate to beads) with vortexing (10 × 30 s vortexing, followed by 1 min incubation on ice). The HAP1 lysates were then centrifuged (600 × *g* for 5 min at 4 °C; ~80% of the supernatant was transferred into tubes, then the remaining ~20% of supernatant was centrifuged at 14,000 × *g* for 10 min at 4 °C, and then the two fractions from each spin were combined). The protein concentration in the lysates was estimated using the BCA assay kit (#23227), and 270 µg were aliquoted per well into a 96-deep well plate. The total volume in each well was made up to 143 μL for samples to be labeled with HA-Ub-PA, or 147 μL for samples to be labeled with bio-ISG15-PA, with lysis buffer.

Negative and probe-only positive controls were treated with 1.5 μL of DMSO in triplicate. The plate was then incubated at RT for 1 h without shaking.

5.4 µL of HA-Ub-PA or 2.35 µL of bio-ISG15-PA (volumes determined from probe titration assays for protein labeling) were transferred into the respective wells using a multichannel pipette, with an equivalent volume of buffer added to the corresponding negative control wells, and incubated at 37 °C for 45 min without shaking. The final reaction volume with the HAP1 lysate, inhibitor or DMSO, and HA-Ub-PA or bio-ISG15-PA or buffer was 150 µL. Reactions were quenched by addition of SDS and NP-40 (IGEPAL CA-630) to final concentrations of 0.4% (v/v) and 0.5% (v/v), respectively, from a master mixture. The master mixture was prepared immediately before quenching by mixing a 5% (v/v) SDS stock with a 10% (v/v) NP-40 stock in a ratio of 1.6:1 and adding 21.5 μL of the mixture to each well. 20 μL of each reaction mixture was kept as “input” samples (to evaluate efficiency of probe labeling), and the remaining ~150 μL of reaction volume was diluted by addition of 250 µL NP-40 buffer (50 mM Tris, 0.5% (v/v) NP-40 (IGEPAL CA-630), 150 mM NaCl, 20 mM MgCl_2_, pH 7.4) to a total volume of 400 μL.

For immunoprecipitation/pull-down steps, 50 µL of anti-HA magnetic beads or 37.5 µL of streptavidin magnetic Sepharose beads were used per sample. The appropriate total amount of anti-HA magnetic beads or streptavidin magnetic Sepharose beads was split into 450 µL aliquots, washed with 4 × 1.6 mL of NP-40 buffer (50 mM Tris, 0.5% NP-40 (IGEPAL CA-630), 150 mM NaCl, 20 mM MgCl_2_, pH 7.4) using a magnetic rack for bead separation, and transferred into the respective reaction wells using a multichannel pipette with cut tips. The 96-deep well plate was then incubated for 4 h at 4 °C on the Thermo KingFisher Apex system, with the mixing speed set to “medium”. The beads were then washed with 4 × 500 μL NP-40 buffer, as described above, and proteins were eluted from the beads twice with 2× Laemmli buffer supplemented with 50 mM DTT (2 × 55 μL, 2 × 5 min at 95 °C with the mixing speed set to “slow”). Beads were removed from the eluates by programming the system to capture and release the beads into an empty 96-deep well plate. Eluates were reduced with DTT to a final concentration of 10 mM (15 min, RT), then alkylated by the addition of iodoacetamide (IAA) to a final concentration of 20 mM (10 min, RT). Proteins were then digested using trypsin and cleaned up using a 96-well S-trap plate according to the manufacturer’s instructions^84^, dried down in a speedvac and resuspended in 0.1% (v/v) aqueous formic acid.

### High-throughput ABPP Workflow: LC-MS/MS timsTOF for “on bench” ABPP-HT* high-throughput screen and compound concentration-dependence validations

Peptide samples were loaded onto Evotips (Evosep) desalting columns according to the manufacturer’s instructions, then chromatographically separated on an 8 cm × 150 µm analytical column with bead size 1.5 µm (EV1109, Evosep), using an Evosep One liquid chromatography (LC) system with the 100 samples per day (spd) standard method. Peptides were eluted onto a TimsTOF Pro mass spectrometer (Bruker) operated in diaPASEF mode using 8 diaPASEF scans per TIMS-MS scan. The ion mobility range was set to 0.85–1.3 Vs/cm^2^. Each mass window isolated was 25 m/z wide, ranging from 475–1000 m/z with an ion mobility-dependent collision energy that increased linearly from 20 to 59 eV between 0.6 and 1.6 Vs/cm^2^.

### High-throughput ABPP workflow: LC-MS/MS for timsTOF Agilent Bravo ABPP-HT*

The previously reported timsTOF data collected after immunoprecipitation using the Agilent robot sample prep methodology is identical to that detailed above^25^, other than a different ion mobility range (0.6–1.6 Vs/cm^2^), and mass range (400–1000 m/z).

### High-throughput ABPP workflow: LC-MS/MS for timsTOF for thermo KingFisher Apex ABPP-HT*

Peptides were analyzed by nanoLC-MS/MS using an Evosep One LC system coupled with a timsTOF HT (Bruker) equipped with an 8 cm × 150 µm, 1.5 µm analytical column (Evosep). Two hundred nanograms of peptides were separated using the Evosep 60SPD workflow (Analytical solvents A: 0.1% FA and B: acetonitrile plus 0.1% FA). Column was held at 40 °C. Data were acquired in data-independent acquisition PASEF mode with the following settings: m/z range from 100 m/z to 1700 m/z, ion mobility range from 1/K0 = 1.30 to 0.85 Vs/cm^2^ using equal ion accumulation and ramp times in the dual TIMS analyzer of 100 ms each. Each cycle consisted of 8 PASEF ramps covering 21 mass steps each with 25 Da windows, each with 2/3 non-overlapping ion mobility windows covering the 475 to 1000 m/z range and 0.85 and 1.26 Vs/cm^2^ ion mobility range. The collision energy was lowered as a function of increasing ion mobility from 59 eV at 1/K0 = 1.6 Vs/cm^2^ to 20 eV at 1/K0 = 0.6 Vs/cm^2^.

### High-throughput ABPP workflow: Agilent Bravo vs. “on bench” ABPP-HT* comparison LC-MS/MS data analysis

Data was searched in DIA-NN 1.8.1 with search settings left as default and match between runs enabled. Data was searched using a Uniprot *Homo sapiens* database containing isoforms (42,469 entries, retrieved on 15/08/2023). *N* = 3 for HA-Ub-PA positive controls, *N* = 1 for negative controls with no HA-Ub-PA present. The “report.pg_matrix” output was used for analysis. The volcano plot at Fig. S1G (*N* = 3 HA-Ub-PA positive and negative controls) was generated from a two-sample student’s *t* test in Perseus (Version 1.6.15).

### High-throughput ABPP workflow: thermo KingFisher Apex ABPP-HT* workflow LC-MS/MS data analysis

Data was searched in DIA-NN 1.8.1 with search settings left as default and match between runs enabled. Data was searched using a Uniprot *Homo sapiens* database containing isoforms (20412 entries, retrieved 13/12/2023). The “report.pg_matrix” output was used (identical number of DUBs were identified in the “report.unique_genes_matrix” output). DUBs were filtered to select hits >1.5-fold enriched in the positive HA-Ub-PA control *vs* the negative control without HA-Ub-PA. Volcano plots were generated/calculated using GraphPad Prism (Version 10.2.3).

### High-throughput ABPP workflow: ABPP-HT* screen and concentration-dependence data analysis

Data was searched in DIA-NN 1.8.1 with search settings left as default and match between runs enabled. Data was searched using a Uniprot *Homo sapiens* database, not containing isoforms (20,416 entries, retrieved on 15/02/2023). The “report.unique_genes_matrix” output was used to ensure inhibitor identification unique to specific DUBs. DUBs were filtered to remove those that were not >5-fold enriched in the positive HA-Ub-PA control vs. the negative control without HA-Ub-PA. For the concentration-dependent data, the following DUBs were discounted due to their unreliable detection across replicates, likely attributable to their low intensities: OTUD1, USP42, OTUD5, USP1, USP30, USP33, USP37, USP43, and OTUB2. Intensities were normalized as a percentage of the positive HA-Ub-PA control and for the screen multiple two-tailed unpaired (homoscedastic) T-tests were carried out (Microsoft Excel) to identify DUBs with significantly different intensities relative to the positive control. The resultant data were then filtered to select hits with a >20% average reduction in DUB activity. The concentration-dependence ABPP outputs were processed using CurveCurator (Version 0.3.0)^87^, with the alpha asymptote set to 5%.

### In-depth ABPP workflows: sample preparation

Cell lysis and DUB Activity-Based Enrichment: Cells were lysed in lysis buffer (50 mM Tris, pH 7.5, 5 mM MgCl_2_, 0.5 mM EDTA, 1 mM DTT, 250 mM sucrose) containing acid-washed glass beads (Sigma Aldrich #G4649). Glass beads made up approximately one third of the total lysis buffer volume. Cell lysates were vortexed and centrifuged (14 K × *g*, 4 °C, 25 min) to separate the pellet, glass beads, nuclei, and membranes from the supernatant. The supernatant was transferred to Eppendorf tubes and the pellets were discarded.

Proteins extracted from ~1 × 10^7^ cells were used as starting materials. When profiling a DUB inhibitor, PR-619 (Sigma-Aldrich # 662141) was first incubated and mixed at 1000 rpm at 37 °C for 1 h. 25 μg of a DUB probe (HA-Ahx-Ahx-Ub-PA (UbiQ-078), HA-Ahx-Ahx-Ub-VME (UbiQ-035), HA-Ahx-Ahx-Ub-VS (UbiQ-178)) or probe mixtures were added to each of the samples and mixed at 1000 rpm at 37 °C for 1 h. The reaction was quenched by the addition of SDS to 0.4% (v/v) and NP-40 to 0.5% (v/v). Lysate mixtures were then diluted to 1 mL, 0.5 mg/mL with NP-40 lysis buffer (pH 7.4, 50 mM Tris, 0.5% (v/v) NP-40, 150 mM NaCl, 20 mM MgCl_2_). Two hundred microliters of anti-HA-Agarose (Pierce # 26182) slurry, previously washed three times with NP-40 lysis buffer, was added to the samples and incubated on a rotator overnight at 4 °C. After a first centrifugation step (2000 × *g*, 4 °C, 1 min), beads were washed four times with 500 μL of NP-40 lysis buffer. Protein complexes were eluted by boiling beads in 250 μL SDS Laemmli 2X sample buffer.

Methanol-chloroform precipitation was performed prior to protease digestion. For each sample, a 4x volume of methanol was added and vortexed for 5 s. A 1x volume of chloroform was added to the samples, which were then vortexed for 5 s. A 3x volume of water was added, and samples were vortexed for 5 s. Samples were then centrifuged for 10 min at 10,000 rpm. The aqueous and organic phases were removed to leave only the protein pellet. The pellets were subsequently washed with 4x volume of methanol and centrifuged for 10 min at 10,000 rpm. The supernatant was discarded, and pellets were re-dissolved in 100 μL of water. Protein precipitation was repeated for a second time to ensure sufficient removal of detergent.

Sample digestion and TMT labeling were performed using the IST-NHS 96x kit (PreOmics # P.O.00030) according to the manufacturer’s instructions. Briefly, 100 µL lysis buffer was added to each sample and heated to 95 °C while mixing at 1000 rpm for 10 min. Lyophilized digestion enzymes were dissolved in 100 µL water before being added to the samples. The samples were digested at 37 °C for 2 h. For samples to be analyzed by a Label-Free quantitation MS method, digestion was stopped, and the sample was transferred to the cartridge in a centrifuge at 2000 g for 1 min before proceeding the washing steps with wash solvent 1 and 2 provided with the kit. For the remaining samples, 200 µg TMTpro 18 Plex reagents (ThermoFischer Scientific # A52045) were added to each of the samples and incubated for 1 h while shaking at 1000 rpm. A total of 10 µL of 5% (v/v) aqueous hydroxylamine was added to each of the samples. A total of 100 µL of stop reagent was also added to the samples before transferring to cartridge for washing with washing solvent 1 and 2 provided with the kit. Samples were eluted with 250 µL of 50% (v/v) acetonitrile in 0.01% (v/v) aqueous formic acid. Samples labeled with TMT reagents were combined, dried and subjected to high pH fractionation using HPLC (Agilent). Peptides were loaded into a XBridge C18 column 4.6 × 250 mm (Waters # PN186003117) in buffer A (H_2_O, pH 10) and eluted with increasing buffer B (90% (v/v) aqueous acetonitrile, pH 10) at a 0.50 mL/min flow rate over a 96 min gradient. Eluted samples were collected every minute and concatenated to give a total of 24 fractions. Desalting was performed by solid phase extraction (SPE) employing reversed-phase C18 cartridges (Sep-Pak C18 light).

### In-depth ABPP workflows: LC-MS/MS methodology

For LFQ Data Dependent Acquisition (DDA) and Data independent Acquisition (DIA) workflows, peptides were analyzed by liquid chromatography tandem mass spectrometry (LC-MS/MS) using an Ultimate 3000 UHPLC coupled to an Orbitrap Fusion Lumos Mass Spectrometer (both from ThermoFisher). Tryptic peptides were separated on an EASY-spray PepMap column (2 µm, 75 µm × 50 cm; ThermoScientific) using a 60 min linear gradient from 2 to 35% buffer B (5% DMSO, 100% acetonitrile, 0.1% TFA) with a 250 nL/min flow and analyzed on the Orbitrap Fusion Lumos (Thermo Scientific). Data were acquired in DDA and DIA as^19^. In both cases the advance peak detection (ADP) was enabled. For DDA, survey scans were acquired in the Orbitrap at 120 k resolution over a m/z range 400–1500, AGC target of 4e5 and S-lens RF of 30. Fragment ion spectra (MS/MS) were obtained in the Ion trap (rapid scan mode) with a Quad isolation window of 1.6, 40% AGC target and a maximum injection time of 35 ms, with HCD activation and 28% collision energy. For DIA, MS1 scans were acquired in the Orbitrap with 120k resolution (m/z 350–1650), an AGC target of 5 × 10⁵ and a maximum injection time of 20 ms. The full MS events were followed by 40 DIA scan windows per cycle (with variable isolation width) covering the m/z range from 350–1650. The MS/MS scans were acquired in the orbitrap at 30k resolution and normalized HCD set up at 30%.

For TMT labeled samples, mass spectrometric analyses were carried out with a Proxeon Easy Nano-LC fitted with a PepMap C18 column 75 µm × 50 cm (Thermos Scientific #PN ES903) and connected to a Lumos Orbitrap mass spectrometer (Thermo Scientific). Peptides were eluted at 450 nL flowrate during 120 min gradient. MS data was acquired with SPS M3 approach. Each full orbitrap scan (*R* = 60 K, m/z scan range: 385–1350, AGC target: 100k, max. injection time: 100 ms) was acquired and followed with dependent IT MS/MS scans (isolation window: 0.5, CID collision energy (%): 35, AGC target: 30k ions, max. injection time: 175 ms) for a 2.7 s cycle time. SPS MS3 scans were acquired with the following parameters (R: 50k, isolation window = 1.5, AGC target: 120k, HCD collision energy (%): 55, max. injection time: 100 ms).

### In-depth ABPP workflows: LC-MS/MS data analysis single shot LFQ

Data was searched using a Uniprot *Homo sapiens* database containing isoforms (42,469 entries, retrieved on 15/08/2023). Software versions: DIA-NN 1.8.1, Fragpipe 22.0, MaxQuant/MaxDIA 2.6.1.0. All search settings were left as default, with match between runs enabled and cysteine carbamidomethylation included as a fixed modification. Each search was 3 × HA-Ub-* and 3 × HA-Ub-* + 50 µM PR619. The PA warhead alone was searched separately from the PA/VME/VS cocktail of warheads. The intensities output “report.pg_matrix” was used from DIA-NN, the LFQ intensities from “combined_protein” output was used from Fragpipe, and the LFQ intensities from the “protein groups” was used from Maxquant for both DDA and DIA searches.

### In-depth ABPP workflows: LC-MS/MS data analysis TMT fractions

MS data processing was performed with Proteome Discoverer Version 2.4.0.305 (Thermo Scientific) with UniProtKB Swiss-Prot (TaxID = 9606, *Homo sapiens*) fasta. Peptide identifications were carried out using the Sequest™ search engine with the following parameters: precursor tolerance: 10 ppm, fragment tolerance = 0.6 Da, max. missed cleavage sites: 3, dynamic modifications: M oxidation and N/Q deamination, fixed modifications: PreOMics cysteine modification (+113.084 Da) and TMTpro +304.207 N-terminus and K ABP western blot compound validations

### ABPP-HT* screen hit validations

For positive hit validations by western blot the sample preparation detailed for the “on bench” ABPP-HT* were used with the concentrations of compounds indicated in figures. Following compound incubation and reaction with HA-Ub-PA, the reaction was quenched directly with Laemmli buffer supplemented with 100 mM DTT, boiled, and analyzed by western blot. IC_50_ values were extracted by quantifying the HA-Ub-PA labeled band by densitometry (Image studio lite) normalized to the β-actin signal and subsequently normalizing the signal of the DUB activity to the negative and positive HA-Ub-PA controls. Concentration-dependencies were fit to *Y* = 100/(1 + 10^((LogIC_50_-X)*HillSlope)) in Graphpad Prism (Version 10.1.1).

### Compound vs. HA-Ub-PA time dependence competition studies

Samples were processed as for the ABP western blot compound concentration-dependence validations, with the HA-Ub-PA incubated for the indicated time.

### ABP compound cellular permeability studies

MCF-7 cells were cultured in 6-well plates to 90% confluence, with a media change to 1 mL prior to DMSO/compound treatment at the indicated concentration for 4 h. Cells were then washed 3 x with PBS, and collected by scraping in PBS and centrifuged (200 × *g* for 10 min). Pellets were then processed as outlined above in the ABPP screen sample preparation section, with 0.4 µg of HA-Ub-PA being used per 50 µg of lysate protein. The mixture was incubated for 45 min at 37 °C, before being quenched by addition of Laemmli buffer supplemented with 100 mM of DTT; the mixture was then analyzed by western blotting.

### Compound 25 selectivity with biotin-ISG15-PA ABP

For the selectivity of **Compound 25** with biotin-ISG15-PA, the sample preparation detailed for the Thermo KingFisher Apex ABPP-HT* was used with the concentrations of compounds indicated in figures. For each compound, stock solutions (100×) were prepared at a concentration of 0.46, 1.39, 4.16, or 12.5 mM in DMSO in a 96-well plate, and 1.5 μL of each stock were transferred into the respective wells using a multichannel pipette (resulting in a final concentration of 4.6, 13.9, 41.6, or 125 μM). Negative and probe-only positive controls were treated with 1.5 μL of DMSO. The plate was then incubated at RT for 1 h without shaking. Following compound incubation 2.35 µL of bio-ISG15-PA was transferred into the respective wells using a multichannel pipette, with an equivalent volume of buffer added to the corresponding negative control wells and incubated at 37 °C for 45 min without shaking. Following compound incubation and reaction with HA-Ub-PA, the reaction was quenched directly with Laemmli buffer supplemented with 50 mM DTT, boiled, and analyzed by western blot.

### Biotinylated compound 25 derivatives

For HA-Ub-PA ABP assays MCF-7 cell lysates were lysed as detailed for the Thermo KingFisher Apex ABPP-HT* methodology and incubated with biotinylated **Compound 25** derivatives at either 150 μM or 250 μM for 1 h at room temperature. Following the initial incubation, 0.5 µl of HA-Ub-PA was added and incubated at 37 °C for 45 min. Reactions were quenched with Laemmli buffer and DTT (50 mM), boiled and analyzed by western blot with immunoblotting for streptavidin, USP5, USP42, and GAPDH. DMSO-treated samples and reactions lacking HA-Ub-PA ABP were included as vehicle controls. Reactions with non-biotinylated USP5 compound (**Compound 25b**) at 150 μM were included as a negative control. Immunoblotting against USP5, USP42, and GAPDH were also included as controls, positive, negative, and loading, respectively.

### GSH stability assays

The stability of Compounds **25,** **25a** and **25b** was investigated in the presence of L-glutathione (GSH) according to a reported procedure^88^. Assays were performed at 37 °C in phosphate buffer (20 mM, pH = 7.4) containing 1mM L-glutathione (reduced form), 0.01 mM indoprofen as an internal standard, and 0.05 mM compound. 2.0 μL aliquots from an initial assay volume of 40 μL with 1% _v/v_ acetonitrile were sequentially analyzed. The results reveal that Compounds **25,** **25a** and **25b** are stable under the assay conditions for >22 h. By contrast, compound **3** was unstable.

Assays were performed in Agilent screw top vials with fixed insert using a Waters UltraPerformance Liquid Chromatography system fitted with an XBridge^TM^ Premier BEH C18 (2.1 × 50 mm, 2.5 µm, 40 °C) column. (Mobile phases A = H₂O + 0.1%_v/v_ formic acid and B = MeCN + 0.1% _v/v_ formic acid; flow rate 0.80 mL/min; gradient 5% _v/v_ B (0–0.1 min), 5–95% _v/v_ B (0.1–3.0 min), 95% _v/v_ B (3.0–3.5 min), 95–5% _v/v_ B (3.5–4.0 min), 5% _v/v_ B (4.0–4.2 min); injection 2.0 µL; detection at 254 nm; electrospray ionization (ESI) mass spectrometry (MS) in the positive and negative ion modes using an ACQUITY QDa mass spectrometer (Waters))

### Competition assay with a non-biotinylated inhibitor (Compound 25) and *N*-ethylmaleimide (NEM)

For non-biotinylated **Compound 25** and NEM competition assays MCF-7 cell lysates were lysed as detailed for the Thermo KingFisher Apex ABPP-HT* methodology and pre-incubated with either non-biotinylated inhibitor **Compound 25** at 250 μM or NEM at 30 mM at room temperature for 1 h. Following the initial incubation, biotinylated **Compound 25** derivatives at 150 μM or DMSO control were added and incubated at room temperature for 1 h. Reactions were quenched with Laemmli buffer and DTT (50 mM), boiled and analyzed by western blot with immunoblotting for streptavidin, USP5, USP42, and GAPDH. DMSO-treated samples lacking biotinylated USP5 derivatives and/or competitors were included as vehicle controls. Immunoblotting against USP5, USP42 and GAPDH was also included as controls, positive, negative, and loading, respectively.

### OTUD7B protein expression and purification

OTUD7B (129-438) cloned into pOPIN E vector was expressed in Rosetta2 (DE3) *E. coli* cells grown in 2xTY medium supplemented with 100 µg/mL carbenicillin and induced with 400 µM Isopropyl-β-D-thiogalactoside (IPTG) at 18 °C overnight. *E.coli* pellets were lysed in 20 mM Tris pH 8.5, 500 mM NaCl, 20 mM Imidazole, 2 mM β-mercaptoethanol supplemented with lysozyme, DNase, 2 µM pepstatin, 4 µM leupeptin and 1 mM PMSF. Clarified lysates were applied to a HisTrap HP column (Cytiva) and eluted in 20 mM Tris pH 8.5, 500 mM NaCl, 500 mM Imidazole, 2 mM β-mercaptoethanol. Further purification was achieved by anion exchange chromatography (Resource Q, Cytiva) followed by size exclusion chromatography (Superdex 75, Cytiva) in 20 mM HEPES pH 7.5, 200 mM NaCl, 4 mM DTT. Concentrated protein was flash frozen and stored in single-use aliquots at −75 °C.

### Enzyme kinetics

Fluorescence intensity measurements were used to monitor the cleavage of a ubiquitin-rhodamine substrate. All activity assays were performed in black 384-well plates in assay buffer (20 mM Tris, pH 8.0, 150 mM potassium glutamate, 0.1 mM TCEP, 0.03% Bovine Gamma Globulin) with a final assay volume of 20 μL. USP7, 1 nM (USP7 (1-1102), DU15644, MRC Protein Phosphorylation and Ubiquitylation Unit) or USP47, 10 nM (USP47 (N-terminal His), DU15682, MRC Protein Phosphorylation and Ubiquitylation Unit) was added and preincubated with **Compound 3** for 30 min. OTUD7B (OTU domain) was added and preincubated with **Compound 6** for 30 min. 180 nM ubiquitin-rhodamine 110 (Ubiquigent for USP47/7, Bio-Techne for OTUD7B) was added to initiate the reaction, and the fluorescence intensity was recorded for 30 min on a PherastarFSX (BMG Labtech) with an Ex_485_/Em_520_ optic module. Initial rates were plotted against compound concentration to determine IC_50_. To investigate the time-dependence of the potency of **Compound 3**, activity assays were repeated without a preincubation step.

### HDX-MS: sample preparation

Hydrogen Deuterium eXchange Mass Spectrometry (HDX-MS) experiments were performed on the same stock of recombinant USP7 and USP47 protein constructs as those used in the in vitro enzyme kinetics experiments. USP7 was provided at a concentration of 25.3 μM and USP47 at 16.5 μM, respectively. Both proteins were prepared in 50 mM HEPES, pH 7.5, 10% glycerol, 150 mM NaCl, 1 mM DTT. The initial stock concentration of **Compound 3** was 30 mM in DMSO. In solution HDX-MS was performed by preparing a volume-to-volume mixture at a molar ratio of 1:2 of protein:**Compound 3** (i.e., the holo-state). Samples were diluted to achieve a final concentration of 16 pmol on column per reaction for USP7 and 21 pmol on column per reaction for USP47. Equivalent control samples (i.e., the apo-state) were prepared where DMSO was supplemented in place of the compound.

### HDX-MS: data acquisition

HDX-MS was performed as^89^. Briefly, an identical labeling buffer was prepared as that of the protein stocks except in deuterium oxide D_2_O (99+ %D, Cambridge Isotope Laboratories, Tewksbury, MA) and with the 10% glycerol was removed (a final composition of 50 mM HEPES, pH 7.5, 150 mM NaCl, 1 mM DTT). The pH of the labeling buffer was measured and corrected to pD (pD=pH+0.4). The quenching buffer comprises 2 M Guanidine HCl, 100 mM citric acid, pH 2.3 in H_2_O. Proteins +/− **Compound 3** were preincubated for 30 min at room temperature to allow complexes to form. Samples were then diluted with the labeling buffer in a 1:20 ratio, achieving an excess D_2_O concentration of 95%. Several labeling time points were sampled at 20 ᵒC, more specifically, at 30, 600, and 3600 s. All analyses were acquired in triplicate. Non-deuterated controls were prepared in an identical fashion, with H_2_O in place of D_2_O. At the end of each labeling time point, samples were quenched upon addition of quench buffer (1:1 ratio), resulting in a final pH of 2.5. Samples were subsequently digested with a dual pepsin/proteaseXIII column (2.1 × 3.0 mm; NovaBioAssays, MA) at 8 ᵒC for 3 min. Peptides were trapped on a 1.0 mm × 5.0 mm, 5.0 µm trap cartridge (Thermo Scientific™ Acclaim PepMap100) for desalting. The flow rate was maintained at 150 µL/min. Peptides were then separated on a Thermo Scientific™ Hypersil Gold^™^, 50 × 1 mm, 1.9 µm, C18 column, increasing hydrophobicity achieved by a linear gradient of 10% to 40% Buffer B (A: water, 0.1% FA; B: ACN, 0.1% FA). The flow rate was maintained at 40 µL/min. A protease wash (2 M guanidine, 100 mM citric acid, pH 2.3 in H_2_O) was performed for each run to limit carry-over. To minimize back-exchange, the quenching, trapping, and separation steps were performed at 1.5 ᵒC. Labeling, quenching, and online digestion steps were performed with the aid of an automated HDX robot (Trajan Scientific and Medical). Sample preparation was managed in Chronos (version 5.4.1). All samples were acquired in MS1 mode on Thermo Scientific^™^ Orbitrap Exploris^™^ 480 Hybrid^™^ mass spectrometer.

### HDX-MS: data analysis

Before each HDX-MS experiment, an unspecific digested peptide database was created in BioPharma Finder (version 5.2) for non-deuterated USP7 and USP47 proteins through a data-dependent and targeted HCD-MS^2^ acquisition regime^89^. Labeling data were processed and manually curated in HDExaminer version 3.4.2 (Trajan Scientific and Medical). The charge state with the highest quality spectra for all replicates for each peptide across all HDX-MS labeling times was used in the final analysis. The significant differences observed at each residue were used to map HDX-MS consensus effects (based on overlapping peptides) onto the AlphaFold model.

### Caspase-1 assay

PMA-differentiated THP-1 cells were treated as indicated, and the caspase-1 activity in the culture medium (supernatant) was measured using the Caspase-Glo 1 Inflammasome Assay (Promega) according to the manufacturer’s instructions. Briefly, the Caspase-Glo 1 Reagent was prepared by resuspending the Z-WEHD substrate in Caspase-Glo 1 buffer and addition of MG132 inhibitor to a final concentration of 60 μM. Cell culture medium (50 μL) was then added to the Caspase-Glo 1 Reagent (50 μL) in a 96-well plate, and the resultant mixture was incubated at room temperature for 1 h. Luminescence was measured using a FLUOstar Omega multi-mode microplate reader (BMG LABTECH) according to the manufacturer’s instructions.

### MTS cell viability assay

Cell viability was assessed using the CellTiter 96 AQueous One Solution Cell Proliferation Assay (MTS, Promega). The One Solution Reagent (20 μL) was added to each well of a 96-well plate containing 100 μL of sample in culture medium. Following incubation at 37 °C for 1 h, absorbance was measured at the wavelength of 490 nm using a FLUOstar Omega multi-mode microplate reader (BMG LABTECH). Cell viability was calculated according to the manufacturer’s instructions.

### ASC speck oligomer cross-linking

ASC oligomer chemical cross-linking was performed as previously described^90^. Briefly, PMA-differentiated THP-1 cells were primed with LPS (1 μg/mL) for 4 h, followed by stimulation with nigericin (10 μM) for 45 min and indicated concentrations of **Compound 3**. Cells were lysed on ice in Buffer A (20 mM HEPES, pH 7.5, 10 mM KCl, 1.5 mM MgCl₂, 1 mM EDTA, 1 mM EGTA, 320 mM sucrose, and protease inhibitors), and homogenized using a 21-gauge needle. Lysates were centrifuged at 300 × *g* for 8 min at 4 °C. The supernatants were collected, mixed 1:1 with CHAPS buffer (20 mM HEPES, pH 7.5, 5 mM MgCl₂, 0.5 mM EGTA, 0.1% CHAPS, and protease inhibitors), and centrifuged at 2400 × *g* for 8 min to pellet crude inflammasome complexes. Pellets were washed twice with ice-cold PBS, resuspended in CHAPS buffer, and cross-linked with 2 mM DSS (prepared in DMSO) at 37 °C for 20 min. The reaction was quenched with NuPAGE™ LDS Sample Buffer (4X) and subjected to western blot analysis.

### Alignment and structure prediction of *homo sapiens* and Pan *troglodytes* USP47

For kinetic and HDX MS studies of **Compound 3**, full-length *Homo Sapiens* USP7 and full-length *Pan troglodytes* isoform 2 were used. Sequences of full-length *Homo sapiens* canonical USP47 and full-length USP47 from *Pan troglodytes* isoform 2 were aligned using T-coffee multiple sequence alignment with differences reported in Fig. S9A^91^. The sequences, including the His-tag of the recombinant *Pan troglodytes* isoform 2, were then used to predict the structures using AlphaFold 3^92^, with the highest-ranking structures being superposed using PyMOL (version 2.5.0) with an RMSD of 2.511 (Fig. S9B, C).

### OTUD7B intact MS

Binding of OTUD7B to **Compound 6** was detected by Liquid Chromatography Mass Spectrometry (LCMS) using an Agilent 1290 Infinity II LC system connected to an Agilent 6550 accurate mass iFunnel quadrupole time of flight (QTOF) mass spectrometer. OTUD7B was incubated with **Compound 6** in 50 mM HEPES pH 7.5 for 1.0 and 15.0 min and 1.0 µL of sample was injected and loaded onto a *ProSwift RP-4H monolithic phenyl reverse phase*, 1.0 × 50 mm, HPLC column (Thermo). Solvent A consisted of LCMS grade water containing formic acid (0.1% v/v), and solvent B consisted of acetonitrile containing formic acid (0.1% v/v). OTUD7B (OTU domain) and **Compound 6** were separated using a step-wise gradient (0 min–95.0% solvent A, 1.0 min–80% solvent A, 9.0 min–45% solvent A, 10 min–0% solvent A, 11 min–0% solvent A, 12.0 min–95.0% solvent A), followed by a 3.0-min post-run with solvent A. All flow rates were 0.2 mL/min. The mass spectrometer was operated in positive ion mode with a drying gas temperature (225 °C), drying gas flow rate (13 L/min), nebulizer pressure (20 psi), sheath gas temperature (350 °C), sheath gas flow rate (12 L/min), capillary voltage (4000 V), nozzle voltage (1500 V), fragmentor voltage (365 V). All acquired data were analyzed using Agilent MassHunter Qualitative Analysis (Version B.07.00) software.

### HIF-1α hypoxia conditions and Covid-19 infection assay

SARS-CoV-2 Australia/VIC01/2020 virus and cells were provided by the Peter Doherty Institute for Infection and Immunity, Melbourne, Australia at P1 and passaged twice in Vero/hSLAM cells (Cat#04091501) - obtained from the European Collection of Cell Cultures (ECACC), UK. IAV-PR8-eGFP was provided by Jack Tan, and EMCV stocks were provided by Jan Rehwinkel. Virus infectivity was determined by plaque assay on Vero-TMPRSS2 cells as previously reported^93^. Calu-3 cells were infected with the viruses listed above at an MOI of 0.01 for 2 h. Viral inocula were removed, cells washed three times in PBS and maintained in growth media until harvest. Cell lines were maintained at 37 °C and 5% CO_2_ in a standard culture incubator and exposed to hypoxia using an atmosphere-regulated workstation set to 37 °C, 5% CO_2_:1%–5% O_2_:balance N_2_ (InvivO_2_ 200, Baker-Ruskinn Technologies). For quantification of viral RNA, total cellular RNA was extracted using the RNeasy kit (Qiagen) according to the manufacturer’s instructions. Equal amounts of RNA, as determined by Nanodrop analysis, were used in a one-step RT-qPCR using the Takyon-One Step RT probe mastermix (Eurogentec) and run on a Roche Light Cycler 96. For quantification of viral copy numbers, qPCR runs contained serial dilutions of viral RNA standards. Total SARS-CoV-2 RNA was quantified using: 2019-nCoV_N1-F: 5’-GAC CCC AAA ATC AGC GAA AT-3’, 2019-nCoV_N1-R: 5’-TCT GGT TAC TGC CAG TTG AA TCT G-3’, 2019nCoV_N1-Probe: 5’-FAM-ACC CCG CAT TAC GTT TGG ACC-BHQ1-3’. For assaying the antiviral response against **Compound 25b**, Calu-3 cells were infected with a SARS-CoV-2 reporter virus containing a mNeonGreen fluorescent reporter as previously described at an MOI of 0.1^94^ or IAV-PR8-eGFP or EMCV. Infected cells were treated with serial dilutions of the compound for 24 h followed by fixation in 4% PFA. Cells were stained with DAPI and mNeonGreen fluorescence was quantified by Clariostar for SARS-CoV-2 and IAV infections. Fluorescence data were normalized to the DAPI signal and plotted as relative to the DMSO control. For EMCV, infectious virus from was quantified by plaque assay from drug-treated wells as described previously^95^ and expressed as relative to the DMSO control.

### Type I interferon production

Type I interferon production was performed as previously described^96^, using HEK293 cells transduced with a pGreenFire-ISRE reporter. In brief, HAP1 cells were seeded at 5,000 cells per well in a 96-well plate in DMEM containing 10% FBS and 2 mM glutamine. After attachment, cells were treated with **Compound 25b** for 1 h, followed by poly(I:C) (0.5 mg/mL) for 48 h. HAP1 media was then collected, centrifuged, and transferred to HEK293 IFN reporter cells, which were seeded at 25,000 cells per well in a 96-well plate and allowed to attach. Recombinant human IFNα2 (PBL assay science) was used for standards. After 24 h, the One-Glo Luciferase Assay System (Promega) was used to quantify luciferase expression following the manufacturer’s instructions.

### Cell growth and proliferation assays

A total of 5000 cells were seeded into either 6-well or 12-well plates and imaged using an IncuCyte Zoom Imager (Sartorius) for live-cell imaging. Imaging was performed at the specified time points using the phase-contrast channel, as previously described^85^. Cell growth was assessed by measuring the percentage confluence.

### >Molecular docking

A model for human USP47 catalytic domain (UniProt: Q96K76), which is representative of a covalently bound inhibitor conformational state, was generated using the structure of human USP7 in complex with the covalent inhibitor, FT827 (PDB: 5NGF; Turnbull et al.), as the template in SwissModel^97^. Coordinates for **Compound 3** were generated using ChemDraw Prime 23.0.1.10, and molecular docking was performed using CovDock^98^ implemented in Maestro version 13.8.135 (Schrödinger Release 2024-3: Maestro, Schrödinger, LLC, New York, NY, 2024). The best docking pose for **Compound 3** corresponded to a docking score of −5.704.

### Antibodies

The antibodies used for immunoblotting in this study are listed in Table 1.

**Table 1:** List of antibodies for immunoblotting used in this study

Reagent	Brand	Catalog Number	Notes
USP5	Proteintech	15158-1-AP	
USP7	ENZO	BML-PW0540-0100	
USP18 (D4E7)	Cell Signaling Technology	4813	
USP42	Santa Cruz	sc-390604	
USP30	Atlas antibodies	HPA016952	
USP33	Proteintech	20445-1-AP	
USP47	Abcam	ab72143	
OTUD7B	Cell Signaling Technology	14817	
VCPIP1	Cell Signaling Technology	88153	
HA (12CA5)	Roche	11666606001	
CFL1	Cell Signaling Technology	5175	
NLRP3(D4D8T)	Cell Signaling Technology	15101	
Caspase-1(D7F10)	Cell Signaling Technology	3866	
ASC(B-3)	Santa Cruz	sc-514414	
IL-1b(D3U3E)	Cell Signaling Technology	12703	
Cleaved-IL-1b (Asp116) (D3A3Z)	Cell Signaling Technology	83186	
GSDMD	Novus Biologicals	NBP2-33422	
HIF-1α	BD Biosciences	610959	
β-actin	Sigma Aldrich	A5441	Used in all figures except for Fig. 5A
β-actin (8H10D10)	Cell Signaling Technology	3700	Used in Fig. 5A
IRDye 680RD Streptavidin	Li-Cor	926-68079	
GAPDH	Cell Signaling Technology	97166	

## Supplementary information

Jones_Draganov_et_al_Supplementary_Information_Drug_Discovery

## Source data

Jones_Draganov_et_al_Source_Data_S1_Data_for_Figure_1C-D_Drug_Discovery_2026

Jones_Draganov_et_al_Source_Data_S2_Data_for_Figure_2E-J_and_S3_Drug_Discovery_2026

Jones_Draganov_et_al_Source_Data_S3_HDX_Summary_Table_for_Figure_4K-L_and_S3F-G_Drug_Discovery_2026

Jones_Draganov_et_al_Source_Data_S4_HDX_Uptake_Summary_Table_for_Figure_4K-L_and_SF-G_Drug_Discovery_2026

Jones_Draganov_et_al_Source_Data_S5_Data_for_Figure_S1A_S1B_S1C_Drug_Discovery_2026

Jones_Draganov_et_al_Source_Data_S6_Data_for_Figure_S1E_Drug_Discovery_2026

Jones_Draganov_et_al_Source_Data_S7_Data_for_Figure_S1F_S1H_S1I_Drug_Discovery_2026

Jones_Draganov_et_al_Source_Data_S8_Data_for_Figure_S1G_Drug_Discovery_2026

## Data Availability

The mass spectrometry proteomics data have been deposited to the ProteomeXchange Consortium via the PRIDE partner repository with the dataset identifier PXD064209. The mass spectrometry HDX data have been deposited to the ProteomeXchange Consortium via the PRIDE partner repository with the dataset identifier PXD064060.
