# Supplementary material for: Activity-based proteomics discovery of deubiquitinating enzyme inhibitors with immunomodulatory activity
Source: NPJ Drug Discov. 2026 Oct 26;3:52. doi: 10.1038/s44386-026-00092-z (PMC13710844; doi:10.1038/s44386-026-00092-z)
Supplement: Supplementary file 1 [file 44386_2026_92_MOESM1_ESM.pdf]

Supplementary Information

Activity-Based Proteomics Discovery of Deubiquitinating Enzyme Inhibitors with Immunomodulatory Activity

Hannah B. L. Jones[1*Δ], Simeon D. Draganov[1,2Δ], Sofia Schönbauer Huamán[3], Peter A. C. Wing[2], Chuong Nguyen[4], Zhu Liang[1,2,9], Julia Dörner[3], Jasper Lithgow[3], Emma Murphy[1], Alice Beard[1,2], Angela Chen[1,2], Andrea Pierangelini[1], Jack W. Houghton[5], Mackenzie E. Robert[6], Sarah Flannery[1], Cara J. Ellison[7], Sakshi Sharma[3], Anthony Tumber[3], Lynn Wambua[1], Iolanda Vendrell[1], Edward W. Tate[5], Paul R. Elliott[7], Graham M. West[4], Darragh P. O'Brien[1], Eidarus Salah[3], Andrew P. Turnbull[8], Christopher J. Schofield[3], Benedikt M. Kessler[1,2], Lennart Brewitz[3,10*], and Adán Pinto-Fernández[1,2*]

Affiliations:
[1]Target Discovery Institute, Centre for Medicines Discovery, Nuffield Department of Medicine, University of Oxford, Oxford, United Kingdom.
[2]CAMS Oxford Institute and Centre for Medicines Discovery, Nuffield Department of Medicine, University of Oxford, Oxford, United Kingdom.
[3]Chemistry Research Laboratory, Department of Chemistry and the Ineos Oxford Institute for Antimicrobial Research, University of Oxford, Oxford, United Kingdom.
[4]Hit Discovery and Optimization (HDO) Pfizer Inc. Groton, Connecticut, United States of America.
[5]Department of Chemistry, Molecular Sciences Research Hub, Imperial College London, London, United Kingdom.
[6]Discovery Biology and Pharmacology, Pfizer Inc. Groton, Connecticut, United States of America.
[7]Department of Biochemistry, University of Oxford, Oxford, United Kingdom.
[8]Cancer Research Horizons, Francis Crick Institute, London, United Kingdom
[9]Present address: Department of Microbiology, Harvard Medical School, Boston, Massachusetts, United States of America.
[10]Centre for Chemical Biology. Institute of Physical Chemistry, Polish Academy of Sciences, Kasprzaka 44/52, 01-224 Warsaw, Poland.

Equal contributions: Δ

*** To whom correspondence may be addressed. Email:** hannah.jones@ndm.ox.ac.uk, lennart.brewitz@ichf.edu.pl, or adan.pintofernandez@ndm.ox.ac.uk.

This PDF file includes:

- Supplementary Information Text
- Figures S1 to S20
- Supplementary Data S1 to S5
- SI References

Other supporting materials for this manuscript include the following:

- Source Data S1: Source data for Figure 1C-D.
- Source Data S2: Source data for Figure 2E-J and S3.
- Source Data S3: Source data for Figure 4K-L and S3F-G.
- Source Data S4: Source data for Figure 4K-L and S3F-G.
- Source Data S5: Source data for Figure S1A-C.
- Source Data S6: Source data for Figure S1E.
- Source Data S7: Source data for Figure S1F-I.
- Source Data S8: Source data for Figure S1G.

Supplementary Information Text

Synthesis

General information

All reagents were from commercial suppliers (Merck, Thermo Fisher Scientific, Enamine). Anhydrous solvents were under an atmosphere of N_2 or Ar gas. All solvents, liquids and solutions were transferred using stainless steel needles and syringes or gas-tight micro-syringes. All solvents used in extractions, work-up procedures, and purifications were of HPLC grade.

Column chromatography was performed using an automated Biotage® Selekt purification machine using Biotage® Sfär Silica D Duo chromatography cartridges. The cartridge size, solvent gradients, and column volumes (CV) used are specified in individual experimental procedures.

High-performance liquid chromatography (HPLC) purification was performed using a Shimadzu Nexera or a Teledyne ACCQPrep HP150 semi-preparative machine, equipped with an Avantor® ACE® 10 AQ preparative column (250 × 21.2 mm) at flow rate of 20 mL/min, using a linear gradient of 2% (v/v) to 98% (v/v) acetonitrile in water (each containing 0.1% (v/v) formic acid) over 45 or 30 min, unless specified otherwise.

Thin layer chromatography (TLC) analyses were performed on Merck silica gel 60 F_{254} TLC plates; compounds were visualized under UV light followed by ninhydrin stain.

High resolution mass spectrometry (HRMS) data were recorded in the positive ionization mode on an ACQUITY I-Class PLUS UPLC System (Waters, Milford, MA, USA) coupled to an ACQUITY RDa mass spectrometer (Waters, Milford, MA, USA) equipped with an electrospray ionization (ESI) probe.

NMR spectra were recorded using Bruker AVIII HD 400, AVIII HD 600, or AVIII 700 instruments. For ^{1}H NMR, chemical shifts are reported in ppm downfield from tetramethylsilane, referenced to residual protium in the NMR solvent (DMSO-d_6: δ = 2.50 ppm). For ^{13}C NMR, chemical shifts are reported in the scale relative to the NMR solvent (DMSO-d_6: δ = 39.52 ppm). Coupling constants are accurate to 0.1 Hz. ^{19}F NMR chemical shifts are reported in ppm and referenced to an external standard.

IR spectroscopy was performed on purified samples using a Bruker Tensor-27 Fourier transform infrared (FT-IR) instrument.

Optical rotations of purified samples were determined using a Unipol (Schmidt Haensch) polarimeter at the sample concentrations specified in the individual experimental procedures (in g·100 mL^{-1}). Results are reported as $[\alpha]_D^T$ values in deg·cm^2·g^{-1} at a water bath temperature of T = 25 °C and at the wavelength of the D sodium line (λ = 589 nm).

General Synthetic Procedures

General Procedure A

A solution of *N,N'*-dicyclohexylcarbodiimide (DCC) (1)(1.08 equiv.) in anhydrous tetrahydrofuran (0.68 M) was added dropwise to a solution of an *N*-Fmoc-protected α-amino acid (1.0 equiv.) and *N*-hydroxysuccinimide (NHS; 1.0 equiv.) in anhydrous tetrahydrofuran (0.19 M) at 0 °C under an atmosphere of N_2 gas. The reaction mixture was stirred while being allowed to warm to ambient temperature overnight (14-17 h). The resultant suspension was then cooled to –20 °C for 1.5 h and filtered through celite®; the filtrate was concentrated under reduced pressure. The crude residue was redissolved in a minimal amount of acetone, cooled to –20 °C for 3 h, and filtered through celite®. The filtrate was concentrated under reduced pressure to afford the corresponding purified NHS-ester, which was used in the following step without further purification.

General Procedure B

A solution of a commercially sourced L-Lys(Boc)-OH amine (1.5 equiv.) and anhydrous Na_2CO_3 (1.5 equiv.) in a tetrahydrofuran:water mixture ($2{:}1_{v/v}$, 0.10 M) was added dropwise to a solution of an NHS-ester (1.0 equiv.) in tetrahydrofuran (0.17 M) at 0 °C. The reaction mixture was stirred until TLC analysis indicated complete conversion (~1-6 h), before being concentrated under reduced pressure. The crude residue was suspended in ethyl acetate, then washed with aqueous $NaHSO_4$ ($10\%_{w/v}$; 3 times) and brine. The organic layer was dried over anhydrous Na_2SO_4, filtered and concentrated under reduced pressure to afford an amide product which was used in the following step without further purification.

General Procedure C

An amine hydrochloride salt (1.0 equiv.) was added to a solution of *N*-Boc-glycine (1.1 equiv.) in anhydrous dichloromethane (0.24 M) at ambient temperature. *N*-Methylmorpholine (NMM) (1.1 equiv.), 1-hydroxybenzotriazole hydrate (HOBt) (1.1 equiv.) and *N*-(3-dimethylaminopropyl)-*N*'-ethylcarbodiimide (EDCI) (2) (1.1 equiv.) were then sequentially added to the reaction mixture, which was then stirred until TLC analysis indicated complete conversion (~3-6 h). The reaction mixture was concentrated under reduced pressure; the crude residue was suspended in ethyl acetate and was sequentially washed with aqueous $NaHSO_4$ ($10\%_{w/v}$; 4 times), saturated aqueous $NaHCO_3$ (3 times), and brine (3 times). The organic layer was dried over anhydrous Na_2SO_4, filtered and concentrated under reduced pressure to give the amide product, which was used in the following step without further purification.

General Procedure D

HCl in dioxane (4 M; 4.0 equiv.) was added to a solution of an *N*-Boc-protected amine (1.0 equiv.) in anhydrous dichloromethane (0.2 M) at ambient temperature under an atmosphere of N_2. The reaction mixture was stirred overnight (15-19 h), then concentrated under reduced pressure. The crude amine product was used immediately in the following step, without further purification.

General Procedure E

N-Methylla (NMM; 1.5 equiv.) and isobutyl chloroformate (IBCF) (1.0 equiv.) were sequentially added to a solution of an *N*-Fmoc-protected α-amino acid (1.0 equiv.) in tetrahydrofuran (0.06 M) at –15 °C under an atmosphere of N_2 gas. The reaction mixture was stirred vigorously for 10 min, then a solution of an amine hydrochloride (1.0 equiv.) in tetrahydrofuran:water ($3{:}1_{v/v}$, 0.13 M) was added. Additional NMM (1.5 equiv.) was added to the reaction mixture, which was then stirred while warming to ambient temperature over 2 h, before being concentrated under reduced pressure. The crude residue was suspended in water and the suspension was acidified by addition of aqueous H_2SO_4 (2 N) to pH 1, then extracted with ethyl acetate (3 times); the organic extracts were combined and sequentially washed with saturated aqueous $NaHCO_3$ and brine, dried over anhydrous Na_2SO_4, filtered and concentrated under reduced pressure. The crude amide product was used in the following step without purification.

General Procedure F

An excess of *N*,*N*-diethylamine (~72 equiv.) was added to a suspension of an *N*-Fmoc-protected amine (1.0 equiv.) in acetonitrile (0.13 M). The reaction mixture was stirred until TLC analysis indicated complete conversion (~1-1.5 h), before being concentrated under reduced pressure and being azeotroped with acetonitrile to remove residual *N*,*N*-diethylamine (3 times). Then, 4-(*N,N*-dimethylamino)pyridine (DMAP) (1.05 equiv.), anhydrous tetrahydrofuran (0.09 M) and acetic anhydride (1.05 equiv.) or trifluoroacetic anhydride (1.05 equiv.) were added sequentially to the crude residue. The resultant mixture was stirred under reflux for 1 h, then cooled to ambient temperature. The reaction mixture was concentrated under reduced pressure, and the crude residue was redissolved in ethyl acetate before being sequentially washed with aqueous HCl (1 M), saturated aqueous $NaHCO_3$ and brine, dried over anhydrous Na_2SO_4, filtered, and concentrated under reduced pressure. The residue was purified via flash column chromatography and/or reversed-phase high-performance liquid chromatography (HPLC) to give the corresponding amine.

General Procedure G

An *N*-Boc-protected amine (1.0 equiv.) was diluted in formic acid (0.04 M); the resultant mixture was stirred at ambient temperature until TLC analysis indicated complete conversion (~2.5-3 h). The reaction mixture was then concentrated under reduced pressure; the crude residue was suspended in acetonitrile (0.03 M). HCl in dioxane (4 M; 2.0 equiv.) was added to the mixture, which was then sonicated and allowed to sediment. The supernatant was removed before the residue was sonicated in acetonitrile (0.03 M) and allowed to sediment twice more. The supernatant was removed both times, and the resultant solid was dried under reduced pressure and used without further purification as the free deprotected amine.

General Procedure H

To a solution or suspension of a N^{α}-acetylated, N^{ε}-Boc-protected tetrapeptide alkyne (1.0 equiv.) in 2-propanol (0.05 M) was added a solution of HCl in dioxane (4M; ~ 80 equiv.), at ambient temperature under an atmosphere of nitrogen. The reaction mixture was stirred at ambient temperature for 1 h before being concentrated under reduced pressure. The solid residue was suspended in acetonitrile (~ 0.03 M) and sonicated. The dispersed solid was allowed to sediment before the supernatant was removed. The precipitate was dried *in vacuo* to afford the corresponding N^{ε}-deprotected tetrapeptide alkyne as hydrochloride adduct, which was >95% pure as judged by ^{1}H and ^{13}C NMR analysis.

General Procedure I

A solution of an NHS-ester (1.0 equiv.) in DMF (0.06 M) was added dropwise to a solution of an amine (1.0 equiv.) in saturated aqueous $NaHCO_3$ (0.28 M) at ambient temperature. The reaction mixture was stirred until TLC analysis indicated complete conversion (~2 h), before being concentrated under reduced pressure. The residue was purified via high-performance liquid chromatography to give the corresponding amide.

General Procedure J

An excess of *N*,*N*-diethylamine (72 equiv.) was added to a suspension of an *N*-Fmoc-protected amine (1.0 equiv.) in acetonitrile (0.13 M). The reaction mixture was stirred until TLC analysis indicated complete conversion (~1-1.5 h), before being concentrated under reduced pressure and being azeotroped with acetonitrile to remove residual *N*,*N*-diethylamine (3 times). The crude amine product was used immediately, without further purification, in the following step.

Synthetic Procedures and Characterization

2,5-Dioxopyrrolidin-1-yl (((9*H*-fluoren-9-yl)methoxy)carbonyl)-*L*-leucinate (I1)

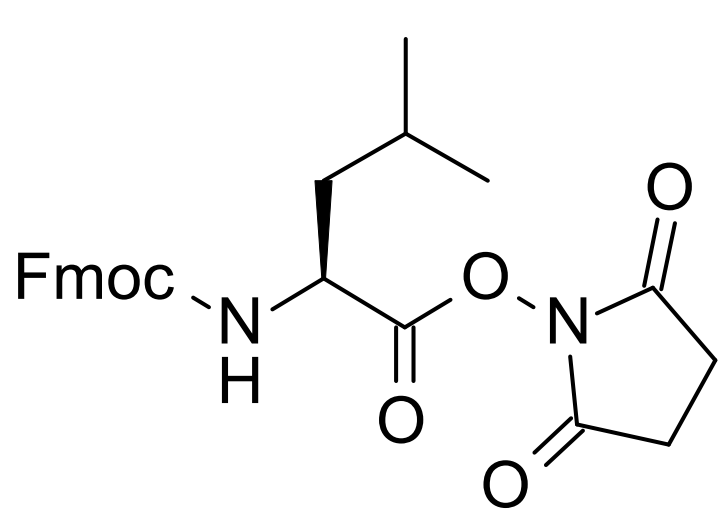

According to General Procedure A, NHS-ester **I1** (3.22 g, apparent quant.) was obtained from Fmoc-L-Leu-OH (2.50 g, 7.1 mmol). Note that ^{1}H NMR spectroscopy revealed the presence of minor amounts of 2,6-di-*tert*-butyl-4-methylphenol (BHT) (<1%), resulting from the use of THF as solvent that contained BHT as a stabilizer; the product was used without further purification.

White foam; **^{1}H NMR** (500 MHz, DMSO-d_6): δ = 8.12 (d, J = 8.1 Hz, 1H), 7.89 (d, J = 7.5 Hz, 2H), 7.72-7.69 (m, 2H), 7.42 (t, J = 7.4 Hz, 2H), 7.32 (tt, J = 7.4, 1.5 Hz, 2H), 4.42 (ddd, J = 10.4, 8.0, 4.5 Hz, 1H), 4.36 (d, J = 7.0 Hz, 2H), 4.24 (t, J = 6.9 Hz, 1H), 2.80 (s, 4H), 1.81-1.69 (m, 2H), 1.68-1.59 (m, 1H), 0.93 (d, J = 6.3 Hz, 3H), 0.88 ppm (d, J = 6.3 Hz, 3H); **^{13}C NMR** (125 MHz, DMSO-d_6): δ = 170.0 (2C), 169.1, 156.0, 143.8, 143.6, 140.8

(2C), 127.7 (2C), 127.0(7), 127.0(6), 125.2 (2C), 120.2, 120.1, 65.8, 50.5, 46.6, 39.3 (determined by HSQC), 25.5 (2C), 24.1, 22.7, 21.0 ppm; **IR** (film): ṽ = 2957, 2926, 2870, 2853, 1816, 1786, 1740, 1522, 1449, 1362, 1325, 1257, 1206, 1066, 1046 cm^{-1}; **HRMS** (ESI): *m/z* calculated for $C_{25}H_{26}O_6N_2Na$ [*M*+Na]$^+$: 473.1683, found: 473.1681; $[\alpha]_D^{25}$ = –31.5 (c = 0.50, $CHCl_3$).

2,5-Dioxopyrrolidin-1-yl (((9*H*-fluoren-9-yl)methoxy)carbonyl)-*D*-leucinate (I2)

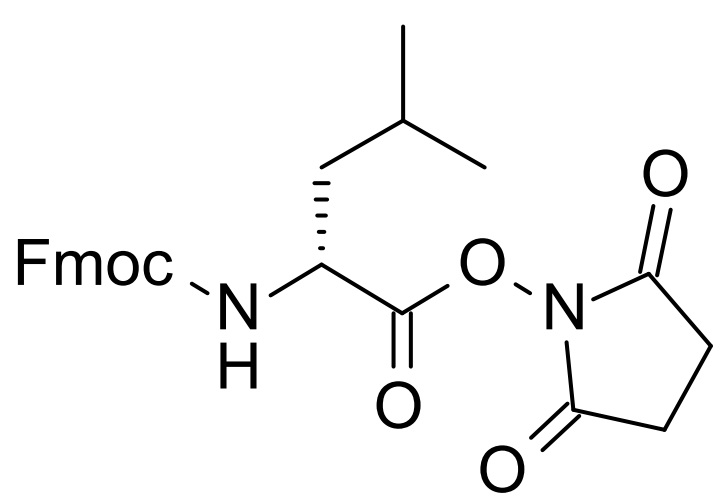

According to General Procedure A, NHS-ester **I2** (1.70 g, 95%) was obtained from Fmoc-D-Leu-OH (1.41 g, 4.0 mmol). Note that ^{1}H NMR spectroscopy revealed the presence of minor amounts of BHT (<2%), resulting from the use of THF as solvent contained BHT as a stabilizer; the product was used without further purification.

White foam; **^{1}H NMR** (500 MHz, DMSO-*d$_6$*): *δ* = 8.12 (d, *J* = 8.1 Hz, 1H), 7.89 (d, *J* = 7.6 Hz, 2H), 7.72-7.69 (m, 2H), 7.42 (t, *J* = 7.5 Hz, 2H), 7.32 (tt, *J* = 7.4, 1.5 Hz, 2H), 4.43 (ddd, *J* = 10.4, 8.0, 4.5 Hz, 1H), 4.36 (d, *J* = 7.0 Hz, 2H), 4.24 (t, *J* = 6.9 Hz, 1H), 2.80 (s, 4H), 1.79-1.71 (m, 2H), 1.65-1.60 (m, 1H), 0.93 (d, *J* = 6.3 Hz, 3H), 0.88 ppm (d, *J* = 6.3 Hz, 3H); **^{13}C NMR** (125 MHz, DMSO-*d$_6$*): *δ* = 170.0 (2C), 169.1, 156.0, 143.8, 143.6, 140.8 (2C), 127.7 (2C), 127.0(7), 127.0(6), 125.2 (2C), 120.2, 120.1, 65.8, 50.5, 46.6, 39.3 (determined by HSQC), 25.5 (2C), 24.1, 22.7, 21.0 ppm; **IR** (film): ṽ = 2956, 2925, 2870, 2852, 1816, 1786, 1739, 1524, 1450, 1361, 1327, 1258, 1206, 1065, 1046 cm^{-1}; **HRMS** (ESI): *m/z* calculated for $C_{25}H_{26}O_6N_2Na$ [*M*+Na]$^+$: 473.1683, found: 473.1681; $[\alpha]_D^{25}$ = +34.6 (c = 0.50, $CHCl_3$).

2,5-Dioxopyrrolidin-1-yl (*S*)-2-((((9*H*-fluoren-9-yl)methoxy)carbonyl)amino)-3-cyclohexylpropanoate (I3)

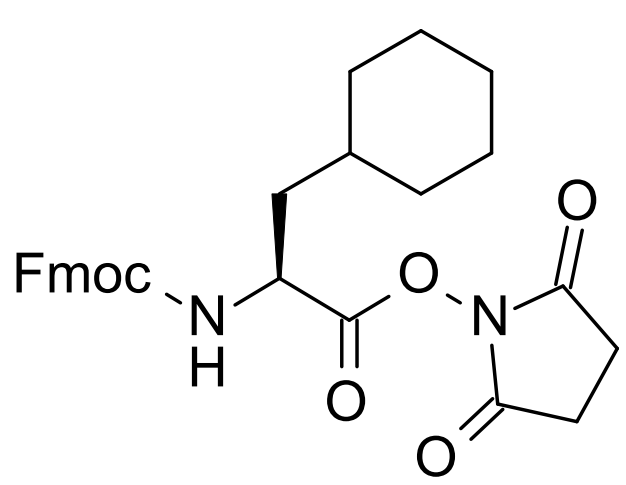

According to General Procedure A, NHS-ester **I3** (1.24 g, 99%) was obtained from Fmoc-L-Cha-OH (1.00 g, 2.6 mmol). Note that ^{1}H NMR spectroscopy revealed the presence of minor amounts of BHT (<2%), resulting from the use of THF as solvent that contained BHT as a stabilizer; the product was used without further purification.

White foam; **^{1}H NMR** (600 MHz, DMSO-*d$_6$*): *δ* = 8.11 (d, *J* = 8.0 Hz, 1H), 7.90 (d, *J* = 7.5 Hz, 2H), 7.71-7.70 (m, 2H), 7.42 (t, *J* = 7.4 Hz, 2H), 7.33 (t, J = 7.5, 2H), 4.45 (ddd, *J* = 10.3, 8.0, 4.6 Hz, 1H), 4.38-4.32 (m, 2H), 4.25 (t, *J* = 6.9 Hz, 1H), 2.80 (s, 4H), 1.76-1.61 (m, 7H), 1.49-1.42 (m, 1H), 1.24-1.09 (m, 3H), 1.00-0.93 (m, 1H), 0.90-0.84 ppm (m, 1H); **^{13}C NMR** (150 MHz, DMSO-*d$_6$*): *δ* = 170.0 (2C), 169.2, 155.9, 143.8, 143.6, 140.7 (2C), 127.7 (2C), 127.1, 127.0, 125.2 (2C), 120.2, 120.1, 65.9, 49.9, 46.6, 38.0, 33.3, 32.9, 31.2, 25.9, 25.7, 25.4(9), 25.4(6) (2C) ppm; **IR** (film): ṽ = 2923, 2851, 1816, 1786, 1740, 1522, 1449, 1361, 1329, 1259, 1205, 1065, 1045 cm^{-1}; **HRMS** (ESI): *m/z* calculated for $C_{28}H_{30}O_6N_2Na$ [*M*+Na]$^+$: 513.1996, found: 513.1994; $[\alpha]_D^{25}$ = –22.4 (c = 0.50, $CHCl_3$).

2,5-Dioxopyrrolidin-1-yl (*S*)-2-((((9*H*-fluoren-9-yl)methoxy)carbonyl)amino)-2-cyclohexylacetate (I4)

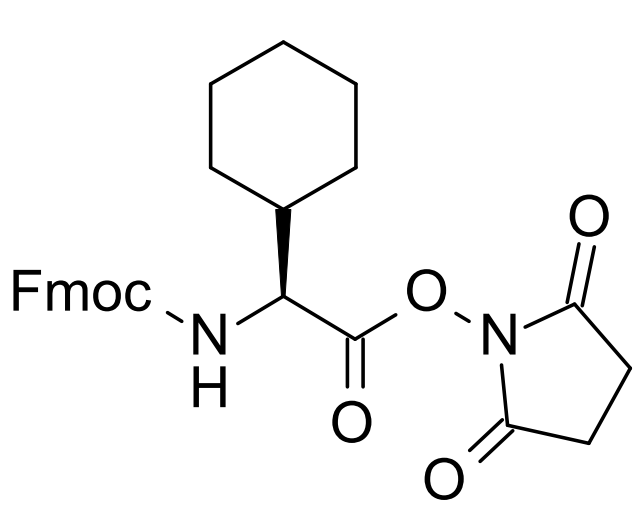

According to General Procedure A, NHS-ester **I4** (1.15 g, 89%) was obtained from Fmoc-L-Chg-OH (1.03 g, 2.7 mmol). Note that ^{1}H NMR spectroscopy revealed the presence of minor amounts of BHT (<2%), resulting from the use of THF as solvent that contained BHT as a stabilizer; the product was used without further purification.

White foam; **^{1}H NMR** (600 MHz, DMSO-*d$_6$*): *δ* = 8.12 (d, *J* = 8.4 Hz, 1H), 7.89 (d, *J* = 7.4 Hz, 2H), 7.75 (d, *J* = 7.5 Hz, 1H), 7.73 (d,

J = 7.5 Hz, 1H), 7.41 (t, *J* = 7.4 Hz, 2H), 7.36-7.29 (m, 2H), 4.36-4.30 (m, 3H), 4.24 (t, *J* = 7.2 Hz, 1H), 2.81 (s, 4H), 1.89-1.84 (m, 1H), 1.80-1.78 (m, 1H), 1.72-1.71 (m, 3H), 1.63-1.61 (m, 1H), 1.28-1.08 (m, 5H); **^{13}C NMR** (150 MHz, DMSO-*d6*): *δ* = 170.0 (2C), 167.8, 156.2, 143.8, 143.6, 140.7 (2C), 127.7 (2C), 127.1, 127.0, 125.3(2), 125.2(6), 120.1(1), 120.1(0), 66.0, 57.4, 46.6, 39.1 (determined by HSQC), 28.4, 27.7, 25.5 (2C), 25.4 (3C) ppm; **IR** (film): ṽ = 2925, 2853, 1815, 1785, 1740, 1527, 1515, 1450, 1358, 1331, 1297, 1247, 1205, 1064, 1033 cm^{-1}; **HRMS** (ESI): *m/z* calculated for $C_{27}H_{28}O_6N_2Na$ [*M*+Na]$^+$: 499.1840, found: 499.1840; $[\alpha]_D^{25}$ = –8.8 (c = 0.50, $CHCl_3$).

N^2-((((9*H*-Fluoren-9-yl)methoxy)carbonyl)-*L*-leucyl)-N^6-(*tert*-butoxycarbonyl)-*L*-lysine (I5)

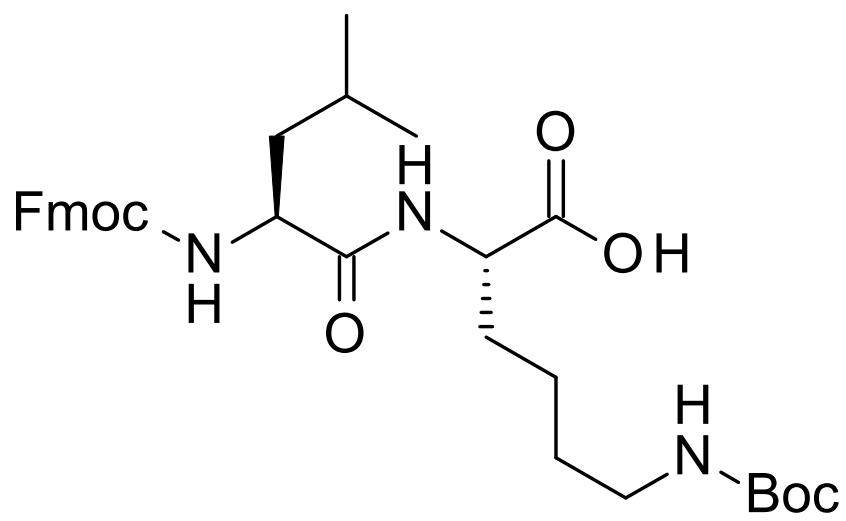

According to General Procedure B, dipeptide **I5** (1.54 g, 98%) was obtained from NHS-ester **I1** (1.22 g, 2.71 mmol). Note that evidence for partial loss (<2%) of the Fmoc group was observed in using ^{1}H NMR spectroscopy.

White foam; **^{1}H NMR** (500 MHz, DMSO-*d6*): *δ* = 12.50 (br, 1H), 8.04 (d, *J* = 7.6 Hz, 1H), 7.89 (d, *J* = 7.6 Hz, 2H), 7.72 (t, *J* = 7.4 Hz, 2H), 7.48 (d, *J* = 8.6 Hz, 1H), 7.41 (t, *J* = 7.4 Hz, 2H), 7.33-7.30 (m, 2H), 6.74 (t, *J* = 5.4 Hz, 1H), 4.31-4.19 (m, 3H), 4.15-4.06 (m, 2H), 2.89-2.85 (app. q, 2H), 1.72-1.53 (m, 3H), 1.50-1.39 (m, 2H), 1.38-1.21 (m, 13H), 0.89 (d, *J* = 6.6 Hz, 3H), 0.85 ppm (d, *J* = 6.6 Hz, 3H); **^{13}C NMR** (125 MHz, DMSO-*d6*): *δ* = 173.5, 172.4, 155.8, 155.5, 144.0, 143.7, 140.7 (2C), 127.6 (2C), 127.0 (2C), 125.3 (2C), 120.1(0), 120.0(7), 77.31, 65.52, 52.82, 51.78, 46.69, 40.73, 39.5 (determined by HSQC), 30.7, 29.1, 28.3 (3C), 24.1, 23.1, 22.8, 21.5 ppm; **IR** (film): ṽ = 3312, 3066, 3009, 2956, 2927, 2869, 1708, 1665, 1532, 1451, 1407, 1394, 1367, 1338, 1319, 1251, 1171, 1122, 1079, 1044 cm^{-1}; **HRMS** (ESI): *m/z* calculated for $C_{32}H_{44}O_7N_3$ [*M*+H]$^+$: 582.3174, found: 582.3171; $[\alpha]_D^{25}$ = –0.2 (c = 0.50, $CHCl_3$).

N^2-((((9*H*-Fluoren-9-yl)methoxy)carbonyl)-*D*-leucyl)-N^6-(*tert*-butoxycarbonyl)-*L*-lysine (I6)

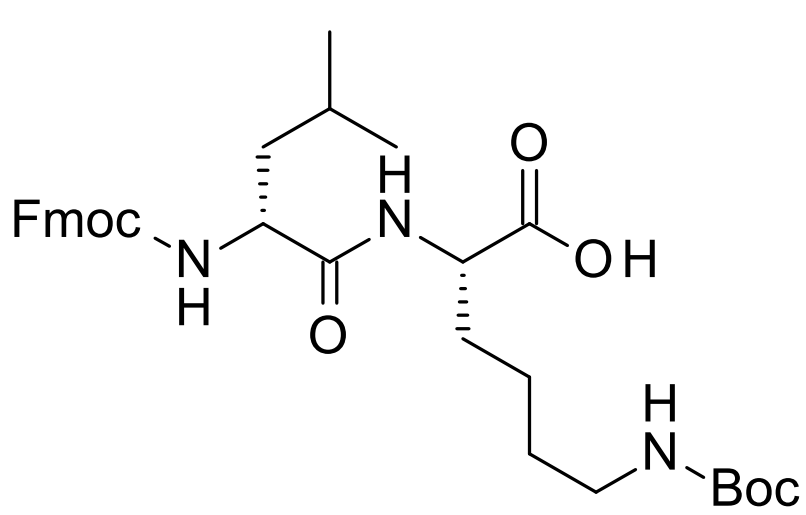

According to General Procedure B, dipeptide **I6** (605 mg, 94%) was obtained from NHS-ester **I2** (500 mg, 1.1 mmol).

White foam; **^{1}H NMR** (600 MHz, DMSO-*d6*): *δ* = 12.56 (br, 1H), 8.12 (d, *J* = 7.9 Hz, 1H), 7.89 (d, *J* = 7.5 Hz, 2H), 7.73 (t, *J* = 7.6 Hz, 2H), 7.45 (d, *J* = 8.8 Hz, 1H), 7.41 (t, *J* = 7.4 Hz, 2H), 7.33-7.30 (m, 2H), 6.73 (t, *J* = 5.5 Hz, 1H), 4.29-4.19 (m, 3H), 4.16-4.11 (m, 2H), 2.90-2.81 (m, 2H), 1.70-1.64 (m, 1H), 1.64-1.53 (m, 2H), 1.51-1.46 (m, 1H), 1.42-1.30 (m, 12H), 1.27-1.18 (m, 2H), 0.89 (d, *J* = 6.6 Hz, 3H), 0.86 ppm (d, *J* = 6.6 Hz, 3fH); **^{13}C NMR** (150 MHz, DMSO-*d6*): *δ* = 173.5, 172.3, 155.8, 155.5, 143.9, 143.7, 140.7 (2C), 127.6 (2C), 127.0(4), 127.0(1), 125.4, 125.3, 120.1 (2C), 77.3, 65.6, 52.9, 51.7, 46.7, 41.2, 39.4 (determined by HSQC), 30.8, 28.9, 28.3 (3C), 24.2, 23.0, 22.6, 21.5 ppm; **IR** (film): ṽ = 3311, 3069, 3019, 2954, 2927, 2864, 2854, 1712, 1662, 1529, 1479, 1451, 1412, 1367, 1319, 1252, 1172, 1122, 1079, 1044 cm^{-1}; **HRMS** (ESI): *m/z* calculated for $C_{32}H_{42}O_7N_3$ [*M*-H]$^-$: 580.3028, found: 580.3022; $[\alpha]_D^{25}$ = +26.2 (c = 0.50, $CHCl_3$).

N^2-((*S*)-2-((((9*H*-Fluoren-9-yl)methoxy)carbonyl)amino)-3-cyclohexylpropanoyl)-N^6-(*tert*-butoxycarbonyl)-*L*-lysine (I7)

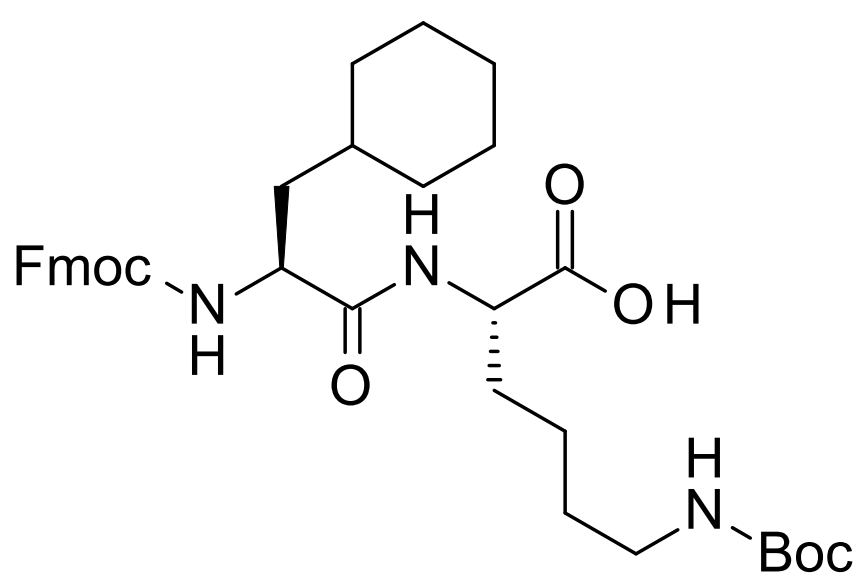

According to General Procedure B, dipeptide **I7** (823 mg, 92%) was obtained from NHS-ester **I3** (706 mg, 1.4 mmol).

White foam; **^{1}H NMR** (500 MHz, DMSO-*d$_6$*): δ = 12.50 (br, 1H), 8.03 (d, *J* = 7.7 Hz, 1H), 7.89 (d, *J* = 7.6 Hz, 2H), 7.73-7.71 (m, 2H), 7.48 (d, *J* = 8.5 Hz, 1H), 7.42 (t, *J* = 7.3 Hz, 2H), 7.33-7.30 (m, 2H), 6.73 (t, J = 5.4 Hz, 1H), 4.31-4.27 (m, 1H), 4.24-4.20 (m, 2H), 4.15-4.08 (m, 2H), 2.89-2.85 (app. q, 2H), 1.72-1.51 (m, 7H), 1.50-1.41 (m, 2H), 1.37-1.16 (m, 15H), 1.16-1.07 (m, 2H), 0.93-0.81 ppm (m, 2H); **^{13}C NMR** (125 MHz, DMSO-*d$_6$*): δ = 173.5, 172.5, 155.8, 155.5, 143.9, 143.8, 140.72 (2C), 127.6 (2C), 127.0 (2C), 125.3 (2C), 120.1 (2C), 77.3, 65.6, 52.2, 51.8, 46.7, 39.5 (determined by HSQC), 39.0 (determined by HSQC), 33.4, 33.2, 31.8, 30.7, 29.1, 28.3 (3C), 26.1, 25.9, 25.7, 22.7 ppm; **IR** (film): ṽ = 3309, 3063, 2925, 2852, 1701, 1669, 1533, 1524, 1478, 1450, 1407, 1395, 1367, 1277, 1249, 1171, 1134, 1107, 1082, 1043 cm^{-1}; **HRMS** (ESI): *m/z* calculated for $C_{35}H_{46}O_7N_3$ $[M\text{-H}]^-$: 620.3341, found: 620.3332; $[\alpha]_D^{25}$ = +1.7 (c = 0.50, $CHCl_3$).

N^2-((*S*)-2-((((9*H*-Fluoren-9-yl)methoxy)carbonyl)amino)-2-cyclohexylacetyl)-N^6-(*tert*-butoxycarbonyl)-*L*-lysine (I8)

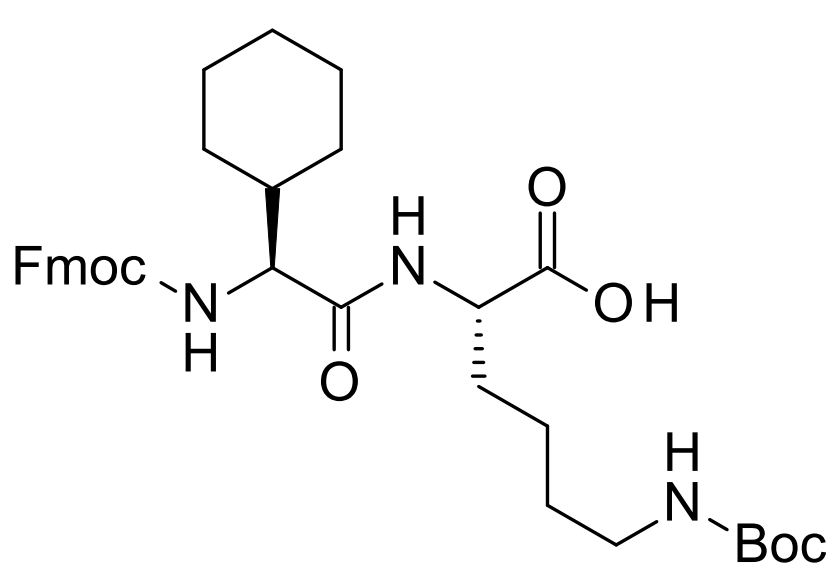

According to General Procedure B, dipeptide **I8** (435 mg, 87%) was obtained from NHS-ester **I4** (390 mg, 0.8 mmol). Note that evidence for partial loss (<2%) of the Fmoc group was observed using ^{1}H NMR spectroscopy.

White amorphous solid; **^{1}H NMR** (500 MHz, DMSO-*d$_6$*): δ = 12.48 (br, 1H), 8.08 (d, *J* = 7.5 Hz, 1H), 7.89 (d, *J* = 7.6 Hz, 2H), 7.76-7.72 (app.t, 2H), 7.41 (t, *J* = 7.5 Hz, 2H), 7.36-7.30 (m, 3H), 6.73 (t, J = 5.4 Hz, 1H), 4.32-4.26 (m, 1H), 4.24-4.19 (m, 2H), 4.15-4.11 (m, 1H), 3.92 (dd, *J* = 9.1, 7.1 Hz, 1H), 2.89-2.85 (app. q, 2H), 1.71-1.53 (m, 8H), 1.37-1.21 (m, 13H), 1.19-0.93 ppm (m, 5H); **^{13}C NMR** (125 MHz, DMSO-*d$_6$*): δ = 173.5, 171.1, 156.0, 155.5, 144.0, 143.8, 140.72, 140.70, 127.6 (2C), 127.0 (2C), 125.4 (2C), 120.0(9), 120.0(6), 77.3, 65.6, 59.2, 51.8, 46.7, 39.6 (determined by HSQC), 39.5 (determined by HSQC), 30.6, 29.1, 29.0, 28.3 (3C), 28.1, 25.9, 25.6(1), 25.5(6), 22.7 ppm; **IR** (film): ṽ = 3308, 3069, 3017, 2927, 2853, 1712, 1663, 1526, 1478, 1450, 1411, 1366, 1335, 1288, 1252, 1171, 1139, 1105, 1083, 1057, 1030 cm^{-1}; **HRMS** (ESI): *m/z* calculated for $C_{34}H_{44}O_7N_3$ $[M\text{-H}]^-$: 606.3185, found: 606.3177; $[\alpha]_D^{25}$ = +8.3 (c = 0.50, $CHCl_3$).

***tert*-Butyl (2-((cyanomethyl)amino)-2-oxoethyl)carbamate** (**I9**)

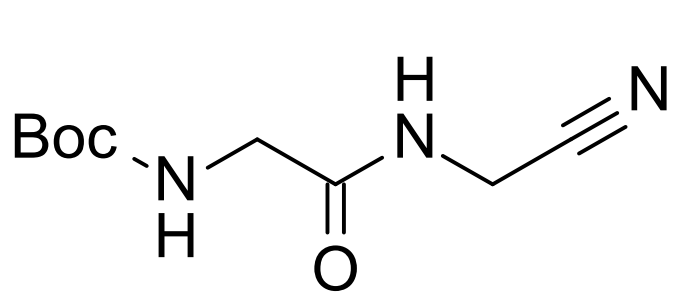

According to General Procedure C, to a solution of Boc-Gly-OH (3.00 g, 17.1 mmol, 1.0 equiv.) in THF (45 mL) were sequentially added *N*-methylmorpholine (1.88 mL, 17.1 mmol, 1.0 equiv.) and isobutyl chloroformate (2.22 mL, 17.1 mmol, 1.0 equiv.) at -25 °C under an atmosphere of nitrogen. Immediately after the formation of a white precipitate, a solution of aminoacetonitrile sulfate (1.99 g, 9.5 mmol, 1.1 equiv. of the free amine) in precooled water (1.5 mL) and 1 N NaOH (18.9 mL) was added to the mixture. The reaction mixture was allowed to warm to ambient temperature and stirred for 2h before THF was removed under reduced pressure. The residue was diluted with water, adjusted to pH 1 with 2N H_2SO_4 and extracted three times with ethyl acetate. The combined organic layers were washed with water (1x), a saturated aqueous solution of $NaHCO_3$ (2x) and brine (1x), dried over Na_2SO_4 and filtered. After

evaporation of the solvent, the solid residue was recrystallized from cyclohexane / ethyl acetate to afford *tert*-butyl (2-((cyanomethyl)amino)-2-oxoethyl)carbamate **I9** (2.18 g, 60%).

White solid; **m.p.**: 113-115 °C; **^{1}H NMR** (600 MHz, DMSO-d_6): δ = 8.50 (t, J = 5.4 Hz, 1H), 7.06 (t, J = 6.0 Hz, 1H), 4.12 (d, J = 5.6 Hz, 2H), 3.57 (d, J = 6.1 Hz, 2H), 1.38 ppm (s, 9H); **^{13}C NMR** (150 MHz, DMSO-d_6): δ = 170.2, 155.8, 117.6, 78.2, 43.0, 28.2 (3C), 27.0 ppm; **IR** (solid): ṽ = 3354, 3319, 2995, 2979, 2942, 1706, 1688, 1671, 1543, 1522, 1432, 1391, 1366, 1350, 1288, 1254, 1239, 1223, 1160, 1081, 1055, 1034 cm^{-1}; **HRMS** (ESI): *m/z* calculated for $C_9H_{15}O_3N_3Na$ [*M*+Na]$^+$: 236.1006, found: 236.1004.

***tert*-Butyl (2-oxo-2-(prop-2-yn-1-ylamino)ethyl)carbamate (I10)**

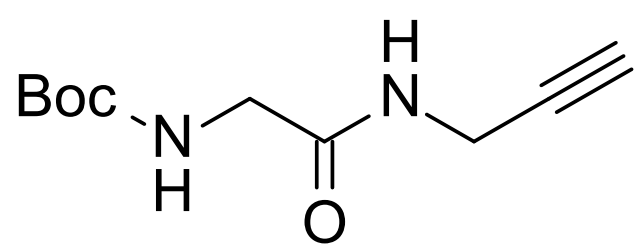

According to General Procedure C, to a solution of Boc-Gly-OH (350 mg, 2.0 mmol, 1.1 equiv.) in anhydrous dichloromethane (7.5 mL) were sequentially added propargylamine (0.12 mL, 1.8 mmol, 1.0 equiv.), N-methylmorpholine (0.22 ml, 2.0 mmol, 1.1 equiv.), HOBt hydrate (306 mg, 2.0 mmol, 1.1 equiv.) and EDCI•HCl (383 mg, 2.0 mmol, 1.1 equiv.) at ambient temperature under an atmosphere of nitrogen. The resulting suspension turned into a solution which was stirred overnight (16 h). The reaction mixture was diluted with ethyl acetate and the resulting suspension was sequentially washed with a 10% aqueous solution of $NaHSO_4$ (3x), a saturated aqueous solution of $NaHCO_3$ (3x) and brine (3x). The organic layer was dried over Na_2SO_4, filtered and evaporated to afford alkyne **I10** (193 mg, 50%) which was used in the following step without further purification.

Off-white solid, **m.p.**: 108.0-111.5 °C; **^{1}H NMR** (600 MHz, DMSO-d_6): δ = 8.21 (t, J = 5.6 Hz, 1H), 6.94 (t, J = 6.2 Hz, 1H), 3.85 (dd, J = 5.5 Hz, 2.5 Hz, 2H), 3.51 (d, J = 6.2 Hz, 2H), 3.09 (t, J = 2.6 Hz, 1H), 1.38 ppm (s, 9H); **^{13}C NMR** (150 MHz, DMSO-d_6): δ = 169.1, 155.8, 81.1, 78.0, 72.9, 43.0, 28.2 (3C), 27.8 ppm; **IR** (solid): ṽ = 3314, 3297, 3059, 3009, 2981, 2931, 2919, 2850, 1704, 1677, 1677, 1542, 1473, 1454, 1420, 1389, 1368, 1293, 1255, 1235, 1172, 1071, 1050, 1031, 1018 cm^{-1}; **HRMS** (ESI): *m/z* calculated for $C_{10}H_{16}O_3N_2Na$ [*M*+Na]$^+$: 235.1053, found: 235.1052.

(9*H*-Fluoren-9-yl)methyl ((*S*)-1-(((*S*)-1-cyano-15,15-dimethyl-3,6,13-trioxo-14-oxa-2,5,12-triazahexadecan-7-yl)amino)-4-methyl-1-oxopentan-2-yl)carbamate (I13)

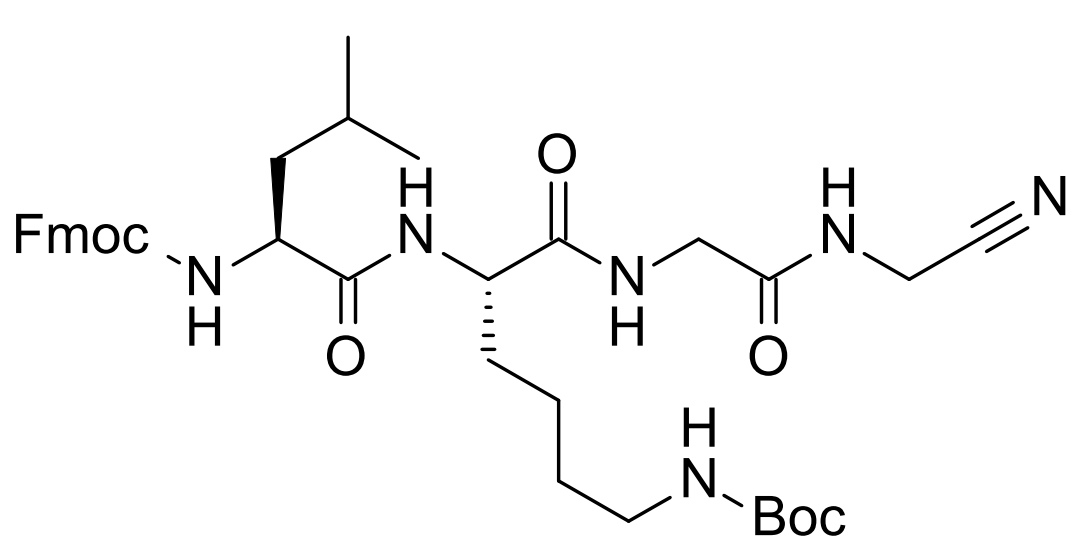

According to General Procedure E, tetrapeptide **I13** (2.01 g, 91%) along with minor impurities was obtained from dipeptide **I5** (1.91 g, 3.3 mmol) and was used in the next step without further purification. Note that evidence for partial loss (~5%) of the Fmoc group was observed using ^{1}H NMR and ^{13}C NMR spectroscopy.

Off-white amorphous solid; **^{1}H NMR** (600 MHz, DMSO-d_6): δ = 8.52 (t, J = 5.3 Hz, 1H), 8.29 (t, J = 5.8 Hz, 1H), 8.00 (d, J = 7.3 Hz, 1H), 7.89 (d, J = 7.5 Hz, 2H), 7.73-7.70 (m, 2H), 7.50 (d, J = 8.5 Hz, 1H), 7.41 (t, J = 7.4 Hz, 2H), 7.33-7.31 (m, 2H), 6.71 (t, J = 5.5 Hz, 1H), 4.36-4.17 (m, 4H), 4.17-4.00 (m, 3H), 3.76 (dd, J = 16.8, 6.1 Hz, 1H), 3.70 (dd, J = 16.8, 5.7 Hz, 1H), 2.88-2.84 (m, 2H), 1.73- 1.57 (m, 2H), 1.56-1.50 (m, 1H), 1.49-1.39 (m, 2H), 1.38-1.29 (m, 11H), 1.29-1.17 (m, 2H, overlap with an impurity signal at 1.23 ppm), 0.88 (d, J = 6.6 Hz, 3H), 0.85 ppm (d, J = 6.5 Hz, 3H); **^{13}C NMR** (150 MHz, DMSO-d_6): δ = 172.6, 172.0, 169.6, 155.9, 155.5, 143.9, 143.7, 140.7 (2C), 127.6 (2C), 127.0 (2C), 125.3 (2C), 120.1, 120.08, 117.5, 77.3, 65.5, 52.9, 52.8, 46.7, 41.7, 40.6, 39.5 (determined by HSQC), 31.4, 29.2, 28.3 (3C), 27.0, 24.2, 23.1, 22.5, 21.4 ppm; **IR** (solid): ṽ = 3293, 3062, 2956, 2936, 2868, 1688, 1659, 1646, 1530, 1479, 1450, 1404, 1392, 1367, 1336, 1320, 1250, 1171, 1120, 1079, 1044 cm^{-1}; **HRMS** (ESI): *m/z* calculated for $C_{36}H_{49}O_7N_6$ [*M*+H]$^+$: 677.3657, found: 677.3658; $[\alpha]_D^{25}$ = –17.2 (c = 0.50, MeOH).

(9*H*-Fluoren-9-yl)methyl ((*R*)-1-(((*S*)-1-cyano-15,15-dimethyl-3,6,13-trioxo-14-oxa-2,5,12-triazahexadecan-7-yl)amino)-4-methyl-1-oxopentan-2-yl)carbamate (I14)

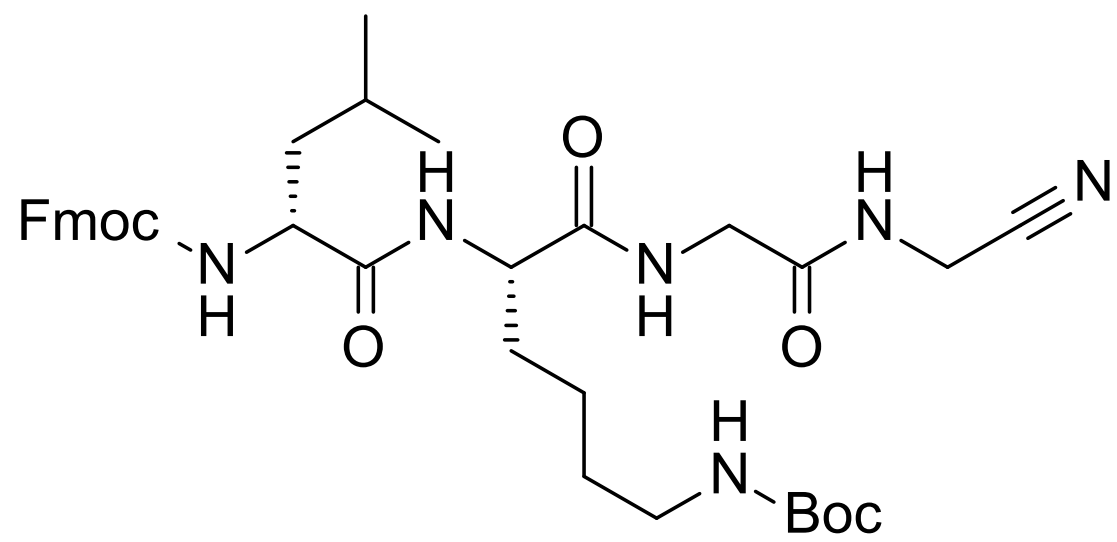

According to General Procedure E, tetrapeptide **I14** (378 mg, 61%) along with minor impurities was obtained from dipeptide **I6** (535 mg, 0.9 mmol), following column chromatography (25 g Sfär; 80 mL/min; initially, 100% dichloromethane (3 CV), followed by a linear gradient (15 CV): 0%$_{v/v}$ → 50%$_{v/v}$ methanol in dichloromethane). Note that evidence for partial loss (~5%) of the Fmoc group was observed using ^{1}H NMR spectroscopy.

Off-white amorphous solid; **^{1}H NMR** (600 MHz, DMSO-*d$_6$*): *δ* = 8.47 (t, *J* = 5.6 Hz, 1H), 8.23 (t, *J* = 6.0 Hz, 1H), 8.20 (d, *J* = 7.7 Hz, 1H), 7.89 (d, *J* = 7.6 Hz, 2H), 7.73-7.70 (app.t, 2H), 7.54 (d, *J* = 8.0 Hz, 1H), 7.41 (t, *J* = 7.4 Hz, 2H), 7.33-7.30 (m, 2H), 6.71 (t, *J* = 5.3 Hz, 1H), 4.32-4.17 (m, 4H), 4.15-4.08 (m, 3H), 3.75 (dd, *J* = 16.7, 6.0 Hz, 1H), 3.70 (dd, *J* = 16.7, 5.8 Hz, 1H), 2.89-2.81 (m, 2H), 1.70-1.62 (m, 1H), 1.61-1.56 (m, 1H), 1.55-1.40 (m, 3H), 1.39-1.27 (m, 11H), 1.26-1.15 (m, 2H, overlap with an impurity signal at 1.23 ppm), 0.89 (d, *J* = 6.5 Hz, 3H), 0.86 ppm (d, *J* = 6.5 Hz, 3H); **^{13}C NMR** (150 MHz, DMSO-*d$_6$*): *δ* = 172.7, 172.0, 169.5, 156.1, 155.5, 143.9, 143.7, 140.7(2), 140.7(0), 127.6 (2C), 127.1, 127.0, 125.3 (2C), 120.1, 120.0(8), 117.5, 77.3, 65.6, 53.1, 52.6, 46.7, 41.7, 40.7, 39.5 (determined by HSQC), 31.3, 29.1, 28.3 (3C), 27.0, 24.2, 22.9, 22.6, 21.6 ppm; **IR** (solid): ṽ = 3294, 3067, 2930, 2854, 1689, 1639, 1532, 1450, 1391, 1366, 1342, 1283, 1254, 1234, 1171, 1137, 1085, 1032 cm^{-1}; **HRMS** (ESI): *m/z* calculated for $C_{36}H_{49}O_7N_6$ [*M*+H]$^+$: 677.3657, found: 677.3660; $[\alpha]_D^{25}$ = +12.3 (c = 0.50, MeOH).

(9*H*-Fluoren-9-yl)methyl ((*S*)-1-(((*S*)-1-cyano-15,15-dimethyl-3,6,13-trioxo-14-oxa-2,5,12-triazahexadecan-7-yl)amino)-3-cyclohexyl-1-oxopropan-2-yl)carbamate (I15)

According to General Procedure E, tetrapeptide **I15** (554 mg, 95%) was obtained from dipeptide **I7** (497 mg, 0.8 mmol) and was used in the next step without further purification. Note that evidence for partial loss (~5%) of the Fmoc group was observed using ^{1}H NMR spectroscopy.

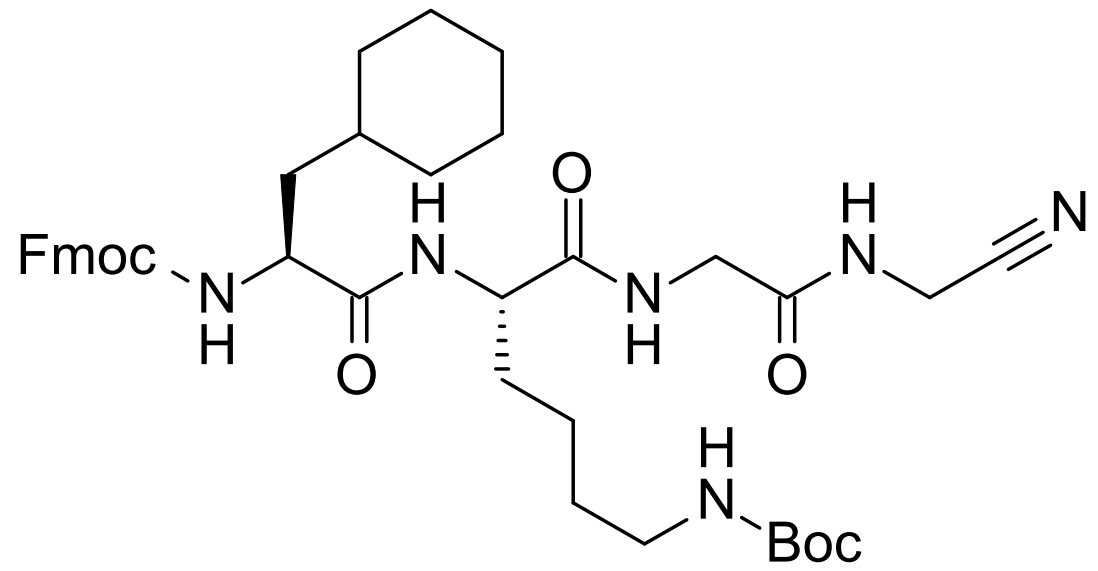

Off-white amorphous solid; **^{1}H NMR** (600 MHz, DMSO-*d$_6$*): *δ* = 8.51 (t, *J* = 5.6 Hz, 1H), 8.27 (t, *J* = 5.8 Hz, 1H), 7.98 (d, *J* = 7.3 Hz, 1H), 7.89 (d, *J* = 7.6 Hz, 2H), 7.73-7.70 (app.t, 2H), 7.51 (d, *J* = 8.4 Hz, 1H), 7.42 (t, *J* = 7.4 Hz, 2H), 7.34-7.30 (m, 2H), 6.71 (t, *J* = 5.3 Hz, 1H), 4.35-4.27 (m, 1H), 4.26-4.16 (m, 3H), 4.14 (d, *J* = 5.6 Hz, 2H), 4.11-4.07 (m, 1H), 3.76 (dd, *J* = 16.8, 5.9 Hz, 1H), 3.70 (dd, *J* = 16.8, 5.5 Hz, 1H), 2.88-2.83 (m, 2H), 1.72-1.56 (m, 6H), 1.55-1.42 (m, 3H), 1.39-1.28 (m, 12H), 1.27-1.14 (m, 3H, overlap with an impurity signal at 1.23 ppm), 1.14-1.07 (m, 2H), 0.93-0.81 ppm (m, 2H); **^{13}C NMR** (150 MHz, DMSO-*d$_6$*): *δ* = 172.7, 172.0, 169.5, 155.9, 155.5, 143.9, 143.7, 140.7 (2C), 127.6 (2C), 127.0 (2C), 125.3 (2C), 120.1 (2C), 117.5, 77.3, 65.5, 52.7, 52.3, 46.6, 41.7, 39.5 (determined by HSQC), 39.0 (determined by HSQC), 33.5, 33.3, 31.6, 31.4, 29.2, 28.2 (3C), 27.0, 26.1, 25.8, 25.6, 22.5 ppm; **IR** (solid): ṽ = 3307, 3066, 2925, 2852, 1648, 1525, 1479, 1449, 1404, 1393, 1366, 1336, 1274, 1248, 1170, 1133, 1104, 1082, 1042 cm^{-1}; **HRMS** (ESI): *m/z* calculated for $C_{39}H_{53}O_7N_6$ [*M*+H]$^+$: 717.3970, found: 717.3972; $[\alpha]_D^{25}$ = –12.4 (c = 0.50, MeOH).

(9*H*-Fluoren-9-yl)methyl ((*S*)-2-(((*S*)-1-cyano-15,15-dimethyl-3,6,13-trioxo-14-oxa-2,5,12-triazahexadecan-7-yl)amino)-1-cyclohexyl-2-oxoethyl)carbamate (I16)

According to General Procedure E, tetrapeptide **I16** (140 mg, 33%) along with minor impurities was obtained from dipeptide **I8** (365 mg, 0.6 mmol) following column chromatography (10 g Sfär; 35 mL/min; initially, 100% ethyl acetate (7 CV), followed by a linear gradient (15 CV): 0%$_{v/v}$ → 100%$_{v/v}$ acetone in ethyl acetate). Note that evidence for partial loss (~15%) of the Fmoc group was observed using ^{1}H NMR and ^{13}C NMR spectroscopy.

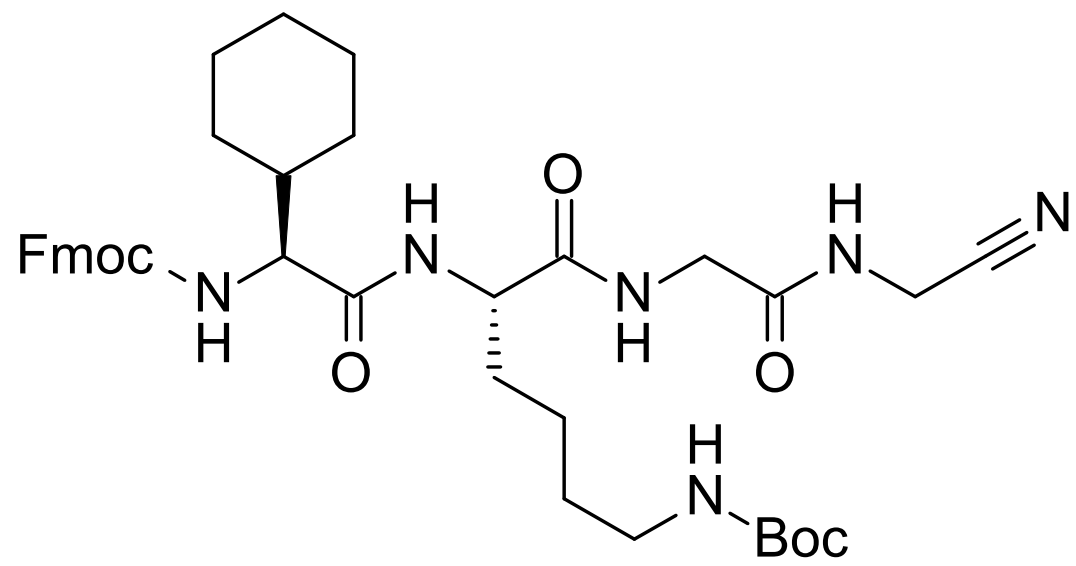

White amorphous solid; **^{1}H NMR** (600 MHz, DMSO-*d$_6$*): *δ* = 8.53 (t, *J* = 5.7 Hz, 1H), 8.30 (t, *J* = 5.6 Hz, 1H), 8.06 (d, 6.9 Hz, 1H), 7.89 (d, *J* = 7.8 Hz, 2H), 7.74 (d, *J* = 7.7 Hz, 1H), 7.72 (d, *J* = 7.9 Hz, 1H), 7.43-7.39 (m, 3H), 7.33-7.31 (m, 2H), 6.71 (t, *J* = 5.4 Hz, 1H), 4.33 (dd, *J* = 9.8, 6.7 Hz, 1H), 4.26-4.21 (m, 2H), 4.19-4.13 (m, 3H), 3.93-3.90 (app.t, 1H), 3.77 (dd, *J* = 16.9, 6.2 Hz, 1H), 3.68 (dd, *J* = 16.8, 5.6 Hz, 1H), 2.88-2.84 (app. q, 2H), 1.87-1.47 (m, 8H), 1.39-1.29 (m, 11H), 1.29-1.18 (m, 2H, overlap with an impurity signal at 1.23 ppm), 1.18-1.03 (m, 3H), 1.01-0.92 ppm (m, 2H); **^{13}C NMR** (150 MHz, DMSO-*d$_6$*): *δ* = 172.0, 171.4, 169.6, 156.0, 155.5, 143.9, 143.7, 140.7(3), 140.7(1), 127.6 (2C), 127.0 (2C), 125.3 (2C), 120.1, 120.0, 117.5, 77.3, 65.5, 59.3, 52.9, 46.7, 41.7, 39.7 (determined by HSQC), 39.6 (determined by HSQC), 31.2, 29.2, 29.0, 28.3 (3C), 28.0, 27.0, 25.8, 25.6 (2C), 22.5 ppm; **IR** (solid): ṽ = 3294, 3066, 2929, 2854, 1690, 1641, 1531, 1450, 1392, 1366, 1335, 1284, 1254, 1233, 1170, 1138, 1103, 1085, 1054, 1030 cm^{-1}; **HRMS** (ESI): *m/z* calculated for $C_{38}H_{50}O_7N_6Na$ [*M*+Na]$^+$: 725.3633, found: 725.3629; $[\alpha]_D^{25}$ = –7.2 (c = 0.50, MeOH).

(9*H*-Fluoren-9-yl)methyl ((*S*)-1-(((*S*)-2,2-dimethyl-4,11,14-trioxo-3-oxa-5,12,15-triazaoctadec-17-yn-10-yl)amino)-4-methyl-1-oxopentan-2-yl)carbamate (I17)

According to General Procedure E, tetrapeptide **I17** (252 mg, 89%) was obtained from dipeptide **I5** (244 mg, 0.4 mmol) and was used in the next step without further purification. Note that evidence for partial loss (~3%) of the Fmoc group was observed using ^{1}H NMR spectroscopy.

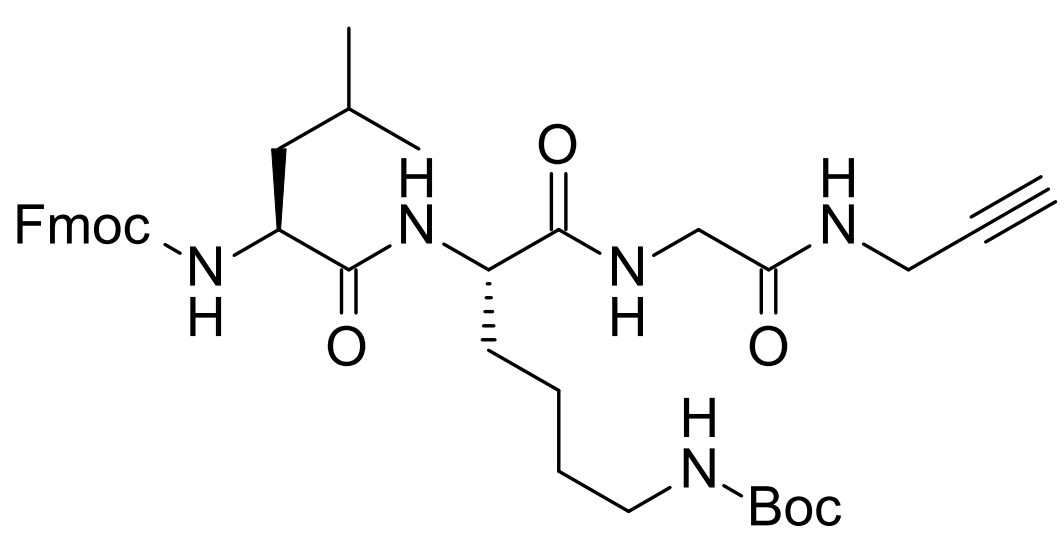

White amorphous solid; **^{1}H NMR** (600 MHz, DMSO-*d$_6$*): *δ* = 8.22 (t, *J* = 5.5 Hz, 1H), 8.16 (t, *J* = 5.8 Hz, 1H), 7.94 (d, *J* = 7.5 Hz, 1H), 7.89 (d, *J* = 7.6 Hz, 2H), 7.73-7.70 (app.t, 2H), 7.51 (d, *J* = 8.3 Hz, 1H), 7.41 (t, *J* = 7.4 Hz, 2H), 7.33-7.30 (m, 2H), 6.71 (t, *J* = 5.3 Hz, 1H), 4.33-4.30 (m, 1H), 4.27-4.25 (m, 1H), 4.23-4.18 (m, 2H), 4.08-4.04 (m, 1H), 3.87 (ddd, *J* = 17.5, 5.6, 2.5 Hz, 1H), 3.84 (ddd, *J* = 17.5, 5.6, 2.5 Hz, 1H), 3.70 (dd, *J* = 16.8, 6.1 Hz, 1H), 3.65 (dd, *J* = 16.7, 5.7 Hz, 1H), 3.09 (t, *J* = 2.5 Hz, 1H), 2.88-2.84 (app. q, 2H), 1.68-1.57 (m, 2H), 1.54-1.40 (m, 3H), 1.39-1.29 (m, 11H), 1.27-1.16 (m, 2H), 0.88 (d, *J* = 6.6 Hz, 3H), 0.85 ppm (d, *J* = 6.5 Hz, 3H); **^{13}C NMR** (150 MHz, DMSO-*d$_6$*): *δ* = 172.4, 171.8, 168.5, 155.9, 155.5, 143.9, 143.7, 140.7 (2C), 127.6 (2C), 127.0 (2C), 125.3 (2C), 120.1, 120.0(7), 81.0, 77.3, 73.0, 65.5, 53.0, 52.6, 46.7, 41.8, 40.6, 39.5 (determined by HSQC), 31.5, 29.2, 28.3 (3C), 27.9, 24.1, 23.1, 22.5, 21.4 ppm; **IR** (solid): ṽ = 3292, 3067, 2930, 2866, 1708, 1681, 1632, 1522, 1464, 1450, 1392, 1366, 1335, 1280, 1254, 1233, 1172, 1121, 1081, 1044 cm^{-1}; **HRMS** (ESI): *m/z* calculated for $C_{37}H_{50}O_7N_5$ [*M*+H]$^+$: 676.3705, found: 676.3697; $[\alpha]_D^{25}$ = –17.4 (c = 0.25, MeOH).

(9*H*-Fluoren-9-yl)methyl ((*R*)-1-(((*S*)-2,2-dimethyl-4,11,14-trioxo-3-oxa-5,12,15-triazaoctadec-17-yn-10-yl)amino)-4-methyl-1-oxopentan-2-yl)carbamate (I18)

According to General Procedure E, tetrapeptide **I18** (496 mg, 94%) along with minor impurities was obtained from dipeptide **I6** (454 mg, 0.8 mmol) and was used in the following step without further purification.

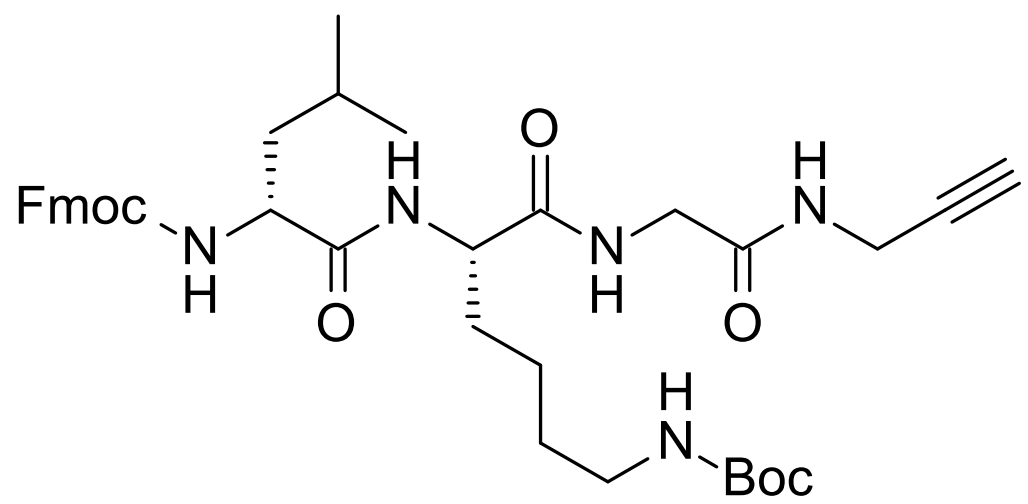

White amorphous solid; **^{1}H NMR** (400 MHz, DMSO-d_6): δ = 8.18-8.11 (m, 3H), 7.89 (d, *J* = 7.6 Hz, 2H), 7.73-7.70 (m, 2H), 7.53 (d, *J* = 7.9 Hz, 1H), 7.41 (t, *J* = 7.4 Hz, 2H), 7.32 (tt, *J* = 7.4, 1.4 Hz, 2H), 6.71 (t, *J* = 5.3 Hz, 1H), 4.32-4.15 (m, 4H), 4.11-4.05 (m, 1H), 3.86 (ddd, *J* = 17.5, 5.7, 2.5 Hz, 1H), 3.80 (ddd, *J* = 17.5, 5.6, 2.5 Hz, 1H), 3.70 (dd, *J* = 16.4, 5.6 Hz, 1H), 3.65 (dd, *J* =16.5, 5.7 Hz, 1H), 3.07 (t, *J* = 2.5 Hz, 1H), 2.90-2.81 (m, 2H), 1.70-1.42 (m, 5H), 1.41-1.29 (m, 11H), 1.27- 1.14 (m, 2H), 0.89 (d, *J* = 6.5 Hz, 3H), 0.86 ppm (d, *J* = 6.5 Hz, 3H); **^{13}C NMR** (150 MHz, DMSO-d_6): δ = 172.6, 171.9, 168.4, 156.1, 155.5, 143.9, 143.7, 140.7 (2C), 127.6 (2C), 127.1 (2C), 125.3 (2C), 120.1, 120.0(8), 80.9, 77.3, 73.0, 65.6, 53.2, 52.6, 46.7, 41.8, 40.7, 39.5 (determined by HSQC), 31.3, 29.0, 28.3 (3C), 27.8, 24.2, 22.9, 22.5, 21.6 ppm; **IR** (solid): ṽ = 3295, 3066, 2924, 2853, 1685, 1638, 1534, 1478, 1449, 1404, 1391, 1366, 1322, 1277, 1250, 1235, 1172, 1123, 1105, 1083, 1040 cm^{-1}; **HRMS** (ESI): *m/z* calculated for $C_{37}H_{50}O_7N_5$ $[M+H]^+$: 676.3705, found: 676.3707; $[\boldsymbol{\alpha}]_D^{25}$ = +11.4 (c = 0.50, MeOH).

(9*H*-Fluoren-9-yl)methyl ((*S*)-3-cyclohexyl-1-(((*S*)-2,2-dimethyl-4,11,14-trioxo-3-oxa-5,12,15-triazaoctadec-17-yn-10-yl)amino)-1-oxopropan-2-yl)carbamate (I19)

According to General Procedure E, tetrapeptide **I19** (536 mg, 97%) along with minor impurities was obtained from dipeptide **I7** (482 mg, 0.8 mmol) and was used in the following step without further purification. Note that evidence for partial loss (~10%) of the Fmoc group was observed using ^{1}H NMR and ^{13}C NMR spectroscopy.

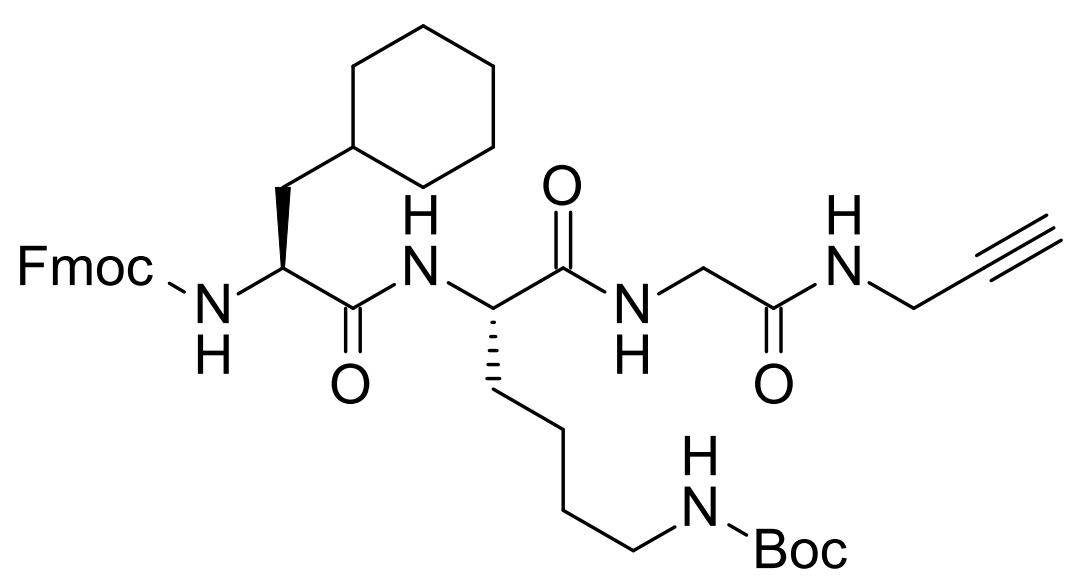

White amorphous solid; **^{1}H NMR** (600 MHz, DMSO-d_6): δ = 8.23 (t, *J* = 5.5 Hz, 1H), 8.15 (t, *J* = 5.8 Hz, 1H), 7.92 (d, *J* = 7.6 Hz, 1H), 7.89 (d, *J* = 7.5 Hz, 2H), 7.72-7.70 (app.t, 2H), 7.52 (d, *J* = 8.3 Hz, 1H), 7.42 (t, *J* = 7.4 Hz, 2H), 7.33-7.31 (m, 2H), 6.71 (t, *J* = 5.3 Hz, 1H), 4.34-4.30 (m, 1H), 4.26-4.18 (m, 3H), 4.10-4.06 (m, 1H), 3.87 (ddd, *J* = 17.5, 5.5, 2.5 Hz, 1H), 3.84 (ddd, *J* = 17.4, 5.5, 2.5 Hz, 1H), 3.71 (dd, *J* = 16.7, 6.1 Hz, 1H), 3.65 (dd, *J* = 16.6, 5.7 Hz, 1H), 3.09 (t, *J* = 2.4 Hz, 1H), 2.87-2.84 (m, 2H), 1.72-1.56 (m, 6H), 1.55-1.41 (m, 3H), 1.39-1.28 (m, 12H), 1.27-1.16 (m, 3H), 1.15-1.07 (m, 2H), 0.93-0.78 ppm (m, 2H); **^{13}C NMR** (150 MHz, DMSO-d_6): δ = 172.5, 171.8, 168.5, 155.9, 155.5, 143.9, 143.7, 140.7(2), 140.6(9), 127.6 (2C), 127.1, 127.0, 125.2(8), 125.2(6), 120.1(2), 120.0(9), 81.0, 77.3, 73.0, 65.6, 52.6, 52.4, 46.7, 41.7, 39.6 (determined by HSQC), 39.0 (determined by HSQC), 33.5, 33.3, 31.7, 31.5, 29.2, 28.3 (3C), 27.9, 26.1, 25.9, 25.7, 22.5 ppm; **IR** (solid): ṽ = 3296, 3068, 2924, 2853, 1686, 1638, 1534, 1448, 1404, 1366, 1311, 1276, 1250, 1229, 1172, 1134, 1104, 1087, 1042 cm^{-1}; **HRMS** (ESI): *m/z* calculated for $C_{40}H_{54}O_7N_5$ $[M+H]^+$: 716.4018, found: 716.4020; $[\boldsymbol{\alpha}]_D^{25}$ = –14.1 (c = 0.50, MeOH).

***tert*-Butyl ((*S*)-5-((*S*)-2-acetamido-4-methylpentanamido)-6-((2-((cyanomethyl)amino)-2-oxoethyl)amino)-6-oxohexyl)carbamate (I20)**

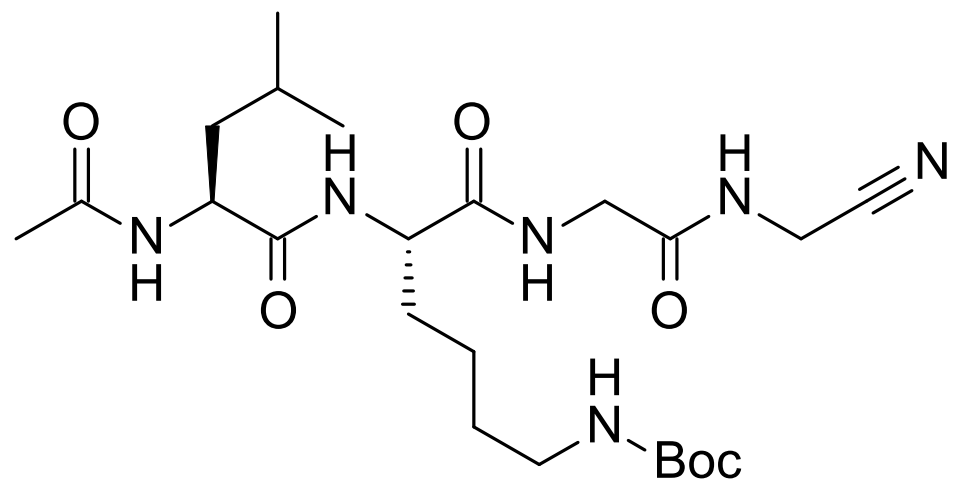

According to General Procedure F, *N*-acetylated peptide **I20** (255 mg, 35%) was obtained from *N*-Fmoc-protected peptide **I13** (1.00 g, 1.5 mmol), following column chromatography (25 g Sfär; 80 mL/min; initially, 100% ethyl acetate (8 CV), followed by a linear gradient (20 CV): 0%$_{v/v}$ → 100%$_{v/v}$ acetone in ethyl acetate).

White amorphous solid; ^{1}**H NMR** (600 MHz, DMSO-*d*$_6$): δ = 8.48 (t, *J* = 5.6 Hz, 1H), 8.20 (t, *J* = 5.9 Hz, 1H), 7.99 (d, *J* = 7.9 Hz, 1H), 7.98 (d, *J* =7.3 Hz, 1H), 6.75 (t, *J* = 5.4 Hz, 1H), 4.30-4.27, (m, 1H), 4.17-4.11 (m, 3H), 3.76 (dd, *J* = 16.8, 6.1 Hz, 1H), 3.69 (dd, 16.8, 5.6 Hz, 1H), 2.89-2.85 (app. q, 2H), 1.84 (s, 3H), 1.68-1.62 (m, 1H), 1.61-1.57 (m, 1H), 1.56-1.49 (m, 2H), 1.47-1.39 (m, 2H), 1.39-1.29 (m, 11H), 1.29-1.16 (m, 2H), 0.88 (d, *J* = 6.6 Hz, 3H), 0.84 ppm (d, *J* = 6.6 Hz, 3H); ^{13}**C NMR** (150 MHz, DMSO-*d*$_6$): δ = 172.5, 172.0, 169.6, 169.4, 155.5, 117.5, 77.3, 52.7, 51.0, 41.7, 40.5, 39.6 (determined by HSQC), 31.3, 29.2, 28.3 (3C), 27.0, 24.2, 23.1, 22.6, 22.5, 21.6 ppm; **IR** (solid): $\tilde{v}$ = 3285, 3067, 2957, 2936, 2870, 1684, 1631, 1539, 1457, 1393, 1367, 1279, 1251, 1172, 1035 cm^{-1}; **HRMS** (ESI): *m/z* calculated for $C_{23}H_{41}O_6N_6$ [*M*+H]$^+$: 497.3082, found: 497.3077; $[\alpha]_D^{25}$ = –21.0 (c = 0.50, MeOH).

***tert*-Butyl ((*S*)-5-((*R*)-2-acetamido-4-methylpentanamido)-6-((2-((cyanomethyl)amino)-2-oxoethyl)amino)-6-oxohexyl)carbamate (I21)**

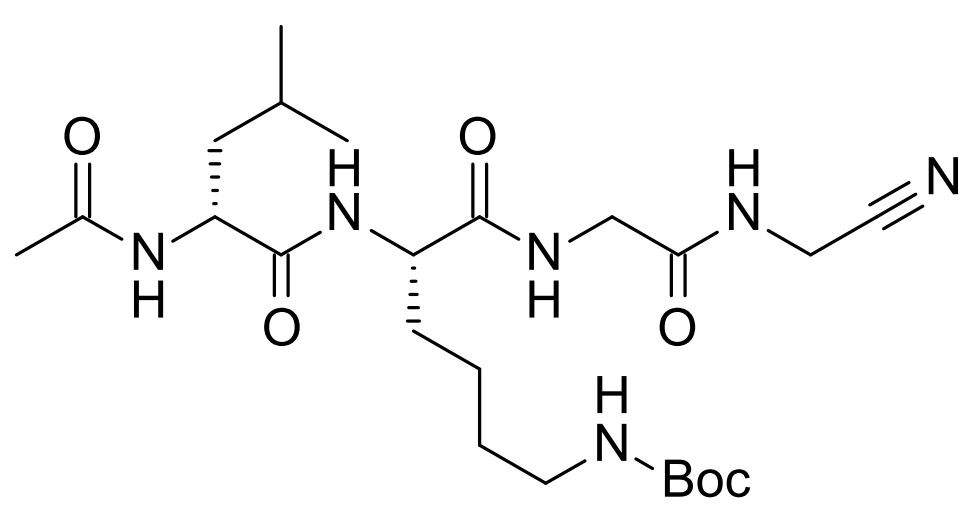

According to General Procedure F, *N*-acetylated peptide **I21** (86 mg, 43%) was obtained from *N*-Fmoc-protected peptide **I14** (357 mg, 0.5 mmol), following column chromatography (10 g Sfär; 35 mL/min; initially, 100% ethyl acetate (7 CV), followed by a linear gradient (15 CV): 0%$_{v/v}$ → 100%$_{v/v}$ acetone in ethyl acetate).

Off-white amorphous solid; ^{1}**H NMR** (600 MHz, DMSO-*d*$_6$): δ = 8.40 (t, *J* = 5.6 Hz, 1H), 8.31 (d, *J* = 7.7 Hz, 1H), 8.22 (t, *J* = 6.0 Hz, 1H), 8.04 (t, *J* = 7.3 Hz, 1H), 6.73 (t, *J* = 5.4 Hz, 1H), 4.28 (q, *J* = 7.5 Hz, 1H), 4.18-4.11 (m, 3H), 3.74 (dd, *J* =16.8, 6.0 Hz, 1H), 3.70 (dd, *J* = 16.7, 6.0 Hz, 1H), 2.91-2.83 (m, 2H), 1.83 (s, 3H), 1.70-1.64 (m, 1H), 1.59-1.49 (m, 2H), 1.42 (t, *J* = 7.3 Hz, 2H), 1.39-1.29 (m, 11H), 1.28-1.16 (m, 2H,), 0.90 (d, *J* = 6.5 Hz, 3H), 0.85 ppm (d, *J* = 6.5 Hz, 3H); ^{13}**C NMR** (150 MHz, DMSO-*d*$_6$): δ = 172.7, 172.1, 169.6, 169.5, 155.5, 117.5, 77.3, 52.7, 51.4, 41.7, 40.8, 39.4 (determined by HSQC), 30.9, 29.0, 28.3 (3C), 27.0, 24.2, 22.7, 22.6, 22.4, 21.9 ppm; **IR** (solid): $\tilde{v}$ = 3284, 3067, 2930, 2853, 1656, 1630, 1535, 1439, 1406, 1391, 1367, 1278, 1250, 1172, 1105, 1038, 1017 cm^{-1}; **HRMS** (ESI): *m/z* calculated for $C_{23}H_{39}O_6N_6$ [*M*-H]$^-$: 495.2937, found: 495.2937; $[\alpha]_D^{25}$ = +1.2 (c = 0.25, MeOH).

***tert*-Butyl ((*S*)-5-((*S*)-2-acetamido-3-cyclohexylpropanamido)-6-((2-((cyanomethyl)amino)-2-oxoethyl)amino)-6-oxohexyl)carbamate (I22)**

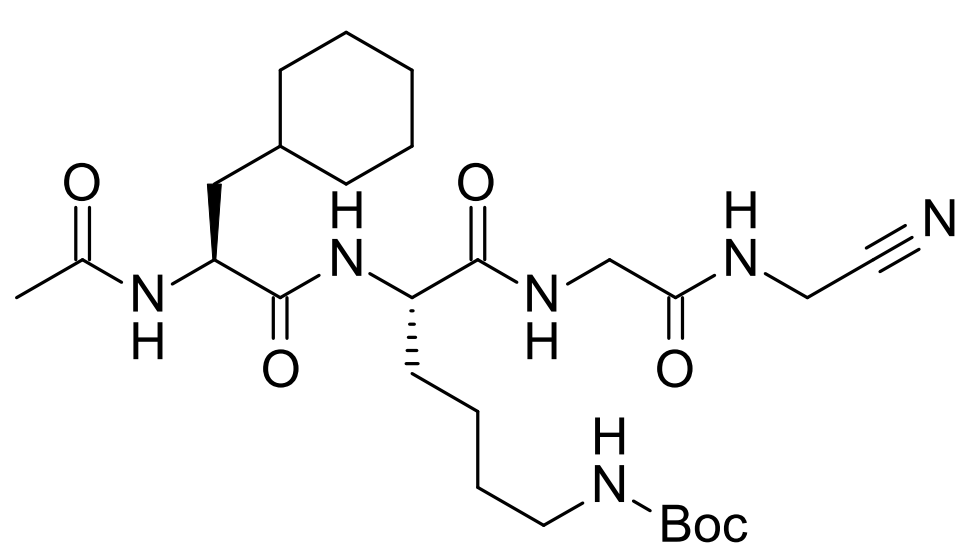

According to General Procedure F, *N*-acetylated peptide **I22** (178 mg, 46%) was obtained from *N*-Fmoc-protected peptide **I15** (717 mg, 0.7 mmol), following column chromatography (25 g Sfär; 80 mL/min; initially, 100% ethyl acetate (7 CV), followed by a linear gradient (15 CV): 0%$_{v/v}$ → 100%$_{v/v}$ acetone in ethyl acetate).

White amorphous solid; ^{1}**H NMR** (600 MHz, DMSO-*d*$_6$): δ = 8.48 (t, *J* = 5.6 Hz, 1H), 8.18 (t, *J* = 5.9 Hz, 1H), 7.99 (d, *J* = 7.9 Hz, 1H), 7.96 (d, *J* = 7.5 Hz, 1H), 6.74 (t, *J* = 5.4 Hz, 1H), 4.29 (ddd, *J* = 9.7, 7.9, 5.1 Hz, 1H), 4.17-4.13 (m, 3H), 3.76 (dd, *J* =

16.8, 6.1 Hz, 1H), 3.69 (dd, J =16.8, 5.6 Hz, 1H), 2.89-2.85 (app. q, 2H), 1.84 (s, 3H), 1.70-1.57 (m, 6H), 1.55-1.50 (m, 1H), 1.49-1.44 (m, 1H), 1.42-1.32 (m, 12H), 1.29-1.23 (m, 2H), 1.22-1.15 (m, 2H), 1.15-1.07 (m, 2H), 1.14-1.07 ppm (m, 2H); **^{13}C NMR** (150 MHz, DMSO-d_6): δ = 172.6, 172.0, 169.6, 169.4, 155.5, 117.5, 77.3, 52.7, 50.4, 41.7, 39.5 (determined by HSQC), 39.1 (determined by HSQC), 33.5, 33.2, 31.9, 31.3, 29.2, 28.3 (3C), 27.0, 26.1, 25.8, 25.6, 22.6, 22.5 ppm; **IR** (solid): ṽ = 3285, 3075, 2924, 2852, 1679, 1631, 1536, 1445, 1393, 1367, 1279, 1251, 1229, 1173, 1038, 1016 cm^{-1}; **HRMS** (ESI): *m/z* calculated for $C_{26}H_{45}O_6N_6$ [M+H]$^+$: 537.3395, found: 537.3391; $[\alpha]_D^{25}$ = –10.3 (c = 0.50, MeOH).

***tert*-Butyl ((*S*)-5-((*S*)-2-acetamido-2-cyclohexylacetamido)-6-((2-((cyanomethyl)amino)-2-oxoethyl)amino)-6-oxohexyl)carbamate (I23)**

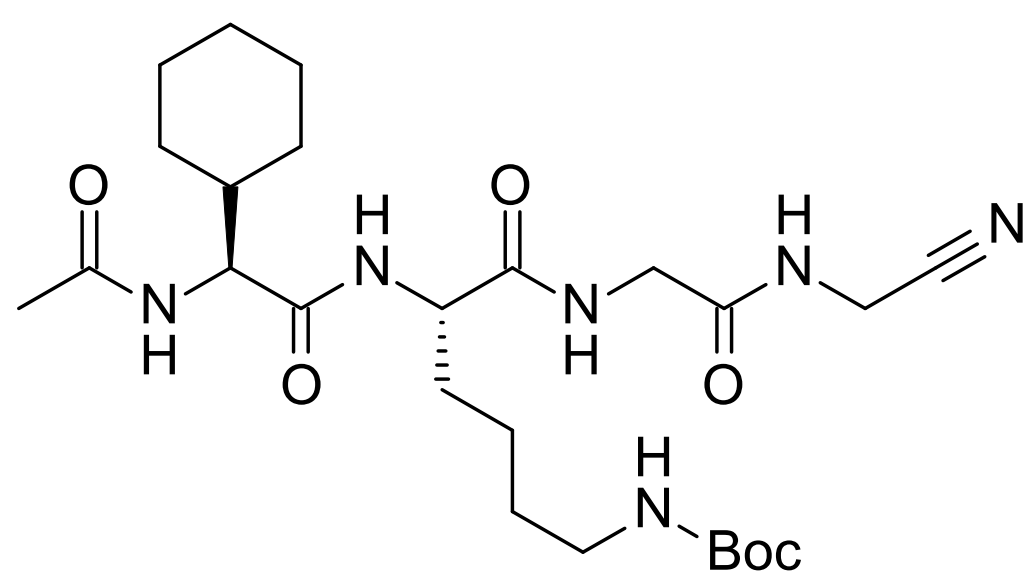

According to a modification of General Procedure F using 2 equiv. of both DMAP (44 mg, 0.36 mmol) and acetic anhydride (34 µL, 0.36 mmol), *N*-acetylated peptide **I23** (22 mg, 23%) along with minor impurities was obtained from *N*-Fmoc-protected peptide **I16** (125 mg, 0.18 mmol), following column chromatography (5 g Sfär; 18 mL/min; initially, 100% ethyl acetate (7 CV), followed by a linear gradient (15 CV): 0%$_{v/v}$ → 100%$_{v/v}$ acetone in ethyl acetate).

Orange amorphous solid; **^{1}H NMR** (600 MHz, DMSO-d_6): δ = 8.50 (t, J = 5.6 Hz, 1H), 8.21 (t, J = 5.8 Hz, 1H), 8.05 (d, J = 7.0 Hz, 1H), 7.87 (d, J = 8.5 Hz, 1H), 6.74 (t, J = 5.5 Hz, 1H), 4.17-4.10 (m, 4H), 3.77 (dd, J = 16.8, 6.1 Hz, 1H), 3.68 (dd, J = 16.8, 5.4 Hz, 1H), 2.88-2.85 (app. q, 2H), 1.86 (s, 3H), 1.67-1.49 (m, 8H), 1.40-1.31 (m, 11H), 1.30-1.17 (m, 2H, overlap with an impurity signal at 1.23 ppm), 1.16-1.03 (m, 3H), 1.01-0.92 ppm (m, 2H); **^{13}C NMR** (150 MHz, DMSO-d_6): δ = 172.0, 171.4, 169.6, 169.4, 155.5, 117.5, 77.3, 57.2, 52.9, 41.8, 39.6 (determined by HSQC), 39.5 (determined by HSQC), 31.1, 29.2, 29.0, 28.3 (3C), 28.1, 27.0, 25.8, 25.6 (2C), 22.6, 22.5 ppm; **IR** (solid): ṽ = 3284, 3079, 2926, 2853, 1684, 1656, 1629, 1536, 1440, 1392, 1367, 1279, 1251, 1205, 1173, 1145, 1107, 1037, 1017 cm^{-1}; **HRMS** (ESI): *m/z* calculated for $C_{25}H_{42}O_6N_6Na$ [M+Na]$^+$: 545.3058, found: 545.3054; $[\alpha]_D^{25}$ = –11.0 (c = 0.125, MeOH).

***tert*-Butyl ((*S*)-5-((*S*)-2-acetamido-4-methylpentanamido)-6-oxo-6-((2-oxo-2-(prop-2-yn-1-ylamino)ethyl)amino)hexyl)carbamate (I24)**

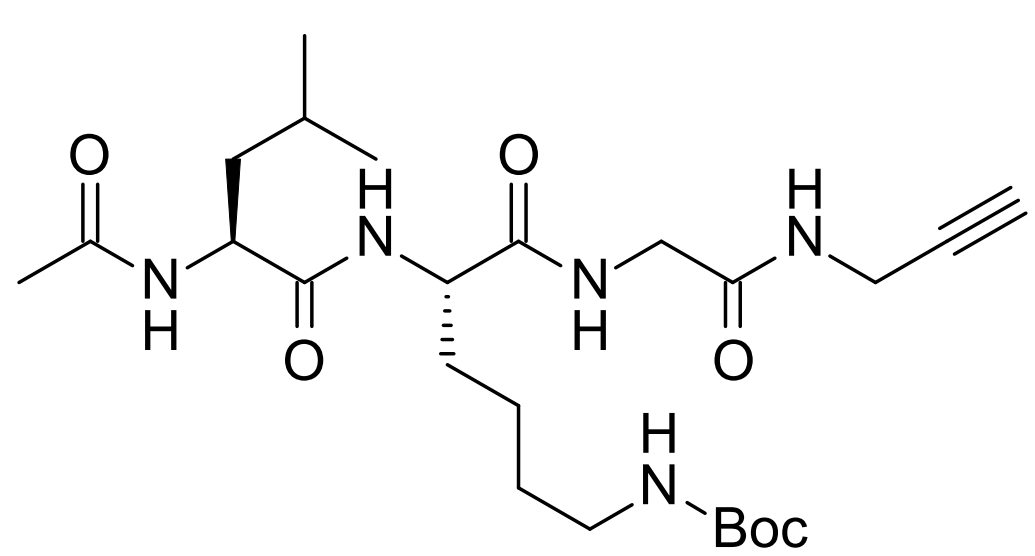

According to General Procedure F, *N*-acetylated peptide **I24** (59 mg, 40%) was obtained from *N*-Fmoc-protected peptide **I17** (200 mg, 0.3 mmol), following column chromatography (10 g Sfär; 35 mL/min; initially, 100% ethyl acetate (10 CV), followed by a linear gradient (20 CV): 0%$_{v/v}$ → 100%$_{v/v}$ acetone in ethyl acetate).

White amorphous solid; **^{1}H NMR** (600 MHz, DMSO-d_6): δ = 8.19 (t, J = 5.6 Hz, 1H), 8.10 (t, J = 5.9 Hz, 1H), 7.99 (d, J = 8.0 Hz, 1H), 7.94 (d, J = 7.5 Hz, 1H), 6.74 (t, J = 5.8 Hz, 1H), 4.29-4.26 (m, 1H), 4.17-4.14 (m, 1H), 3.88 (ddd, J = 17.5, 5.6, 2.6 Hz, 1H), 3.84 (ddd, J = 17.5, 5.6, 2.5 Hz, 1H), 3.70 (dd, J = 16.6, 6.1 Hz, 1H), 3.64 (dd, J = 16.7, 5.7 Hz, 1H), 3.10 (t, J = 2.5 Hz, 1H), 2.89-2.85 (app. q, 2H), 1.84 (s, 3H), 1.68-1.62 (m, 1H), 1.61-1.56 (m, 1H), 1.54-1.46 (m, 1H), 1.45-1.40 (m, 2H), 1.39-1.29 (m, 11H), 1.28-1.16 (m, 2H), 0.88 (d, J = 6.6 Hz, 3H), 0.84 ppm (d, J = 6.6 Hz, 3H); **^{13}C NMR** (150 MHz, DMSO-d_6): δ = 172.4, 171.8, 169.4, 168.5, 155.5, 81.0, 77.3, 73.0, 52.7, 51.0, 41.8, 40.5, 39.5 (determined by HSQC), 31.4, 29.2, 28.3, 27.9, 24.1, 23.1, 22.6, 22.5, 21.6 ppm; **IR** (solid): ṽ = 3289, 3078, 2957, 2934, 2870, 1632, 1532, 1460, 1440, 1391, 1391, 1367, 1277, 1250, 1171, 1097, 1040, 1016 cm-

[1]; **HRMS** (ESI): *m/z* calculated for $C_{24}H_{42}O_6N_5$ [*M*+H]⁺: 496.3130, found: 496.3127; $[\alpha]_D^{25}$ = –20.1 (c = 0.25, MeOH).

***tert*-Butyl ((*S*)-5-((*R*)-2-acetamido-4-methylpentanamido)-6-oxo-6-((2-oxo-2-(prop-2-yn-1-ylamino)ethyl)amino)hexyl)carbamate (I25)**

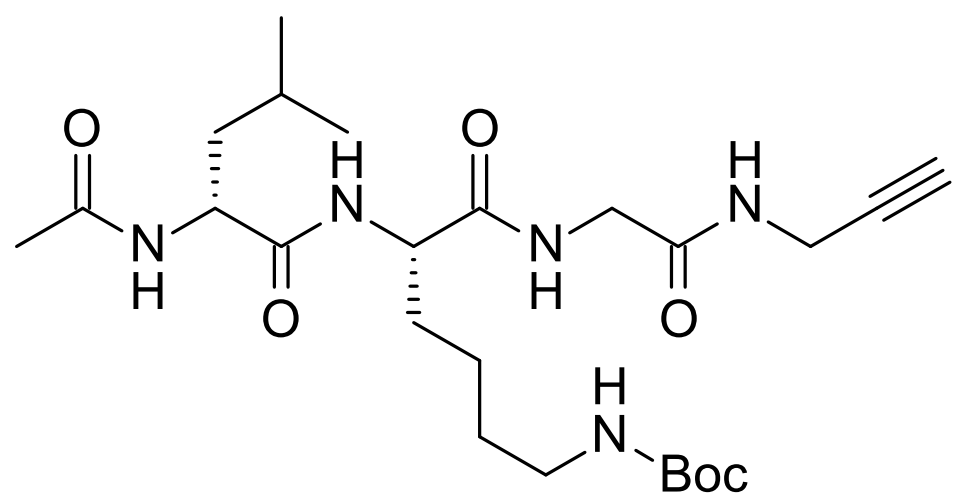

According to General Procedure F, *N*-acetylated peptide **I25** (128 mg, 38%) was obtained from *N*-Fmoc-protected peptide **I18** (456 mg, 0.7 mmol) following column chromatography (25 g Sfär; 80 mL/min; initially, 100% ethyl acetate (7 CV), followed by a linear gradient (15 CV): $0\%_{v/v} \rightarrow 100\%_{v/v}$ acetone in ethyl acetate).

White amorphous solid; **¹H NMR** (600 MHz, DMSO-*d₆*): δ = 8.28 (d, *J* = 7.7 Hz, 1H), 8.13 (t, *J* = 6.0 Hz, 1H), 8.11 (t, *J* = 5.6 Hz, 1H), 8.05 (d, *J* = 7.3 Hz, 1H), 6.73 (t, *J* = 5.4 Hz, 1H), 4.27 (q, *J* = 7.5 Hz, 1H), 4.15-4.12 (m, 1H), 3.88 (ddd, *J* = 17.6, 5.6, 2.5 Hz, 1H), 3.84 (ddd, *J* = 17.5, 5.6, 2.6 Hz, 1H), 3.68 (dd, *J* = 16.6, 5.9 Hz, 1H), 3.65 (dd, *J* = 16.6, 6.0 Hz, 1H), 3.10 (t, *J* = 2.5 Hz, 1H), 2.91-2.82 (m, 2H), 1.83 (s, 3H), 1.70-1.64 (m, 1H), 1.59-1.47 (m, 2H), 1.43-1.41 (m, 2H), 1.40- 1.29 (m, 11H), 1.28-1.16 (m, 2H), 0.90 (d, *J* = 6.6 Hz, 3H), 0.85 ppm (d, *J* = 6.5 Hz, 3H); **¹³C NMR** (150 MHz, DMSO-*d₆*): δ = 172.7, 172.0, 169.6, 168.5, 155.5, 81.0, 77.3, 73.0, 52.7, 51.5, 41.8, 40.7, 39.5 (determined by HSQC), 31.0, 29.0, 28.3 (3C), 27.9, 24.2, 22.7, 22.6, 22.4, 21.9 ppm; **IR** (solid): ṽ = 3294, 3079, 2956, 2934, 2869, 1683, 1655, 1628, 1537, 1452, 1390, 1367, 1279, 1252, 1218, 1174, 1147, 1096, 1040, 1016 cm^{-1}; **HRMS** (ESI): *m/z* calculated for $C_{24}H_{42}O_6N_5$ [*M*+H]⁺: 496.3130, found: 496.3127; $[\alpha]_D^{25}$ = –1.8 (c = 0.25, MeOH).

***tert*-Butyl ((*S*)-5-((*S*)-2-acetamido-3-cyclohexylpropanamido)-6-oxo-6-((2-oxo-2-(prop-2-yn-1-ylamino)ethyl)amino)hexyl)carbamate (I26)**

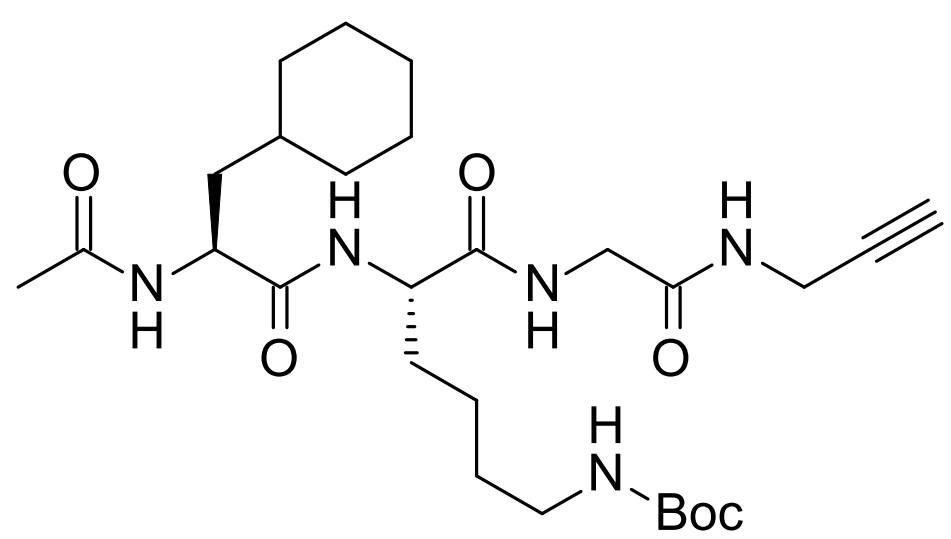

According to General Procedure F, *N*-acetylated peptide **I26** (224 mg, 61%) was obtained from *N*-Fmoc-protected peptide **I19** (491 mg, 0.7 mmol), following column chromatography (25 g Sfär; 80 mL/min; initially, 100% ethyl acetate (7 CV), followed by a linear gradient (15 CV): $0\%_{v/v} \rightarrow 100\%_{v/v}$ acetone in ethyl acetate).

White amorphous solid; **¹H NMR** (600 MHz, DMSO-*d₆*): δ = 8.19 (t, *J* = 5.5 Hz, 1H), 8.08 (t, *J* = 5.8 Hz, 1H), 7.99 (d, *J* = 7.9 Hz, 1H), 7.92 (d, *J* = 7.6 Hz, 1H), 6.74 (t, *J* = 5.4 Hz, 1H), 4.28 (ddd, *J* = 9.7, 7.9, 5.2 Hz, 1H), 4.18-4.14 (m, 1H), 3.88 (ddd, *J* = 17.3, 5.4, 2.5 Hz, 1H), 3.84 (ddd, *J* = 17.3, 5.3, 2.5 Hz), 3.70 (dd, *J* = 16.7, 6.0 Hz, 1H), 3.64 (dd, *J* = 16.6, 5.6 Hz, 1H), 3.10 (t, *J* = 2.5 Hz, 1H), 2.90-2.83 (m, 2H), 1.84 (s, 3H), 1.69-1.58 (m, 6H), 1.54-1.44 (m, 2H), 1.42-1.30 (m, 12H), 1.29-1.15 (m, 4H), 1.14-1.07 (m, 2H), 0.92-0.79 ppm (m, 2H); **¹³C NMR** (150 MHz, DMSO-*d₆*): δ = 172.4, 171.8, 169.4, 168.5, 155.5, 80.9, 77.3, 73.0, 52.6, 50.4, 41.8, 39.6 (determined by HSQC), 39.0 (determined by HSQC), 33.5, 33.2, 31.9, 31.3, 29.2, 28.3 (3C), 27.9, 26.1, 25.8, 25.6, 22.6, 22.5 ppm; **IR** (solid): ṽ = 3340, 3302, 3275, 3070, 2930, 2853, 1702, 1676, 1624, 1534, 1446, 1417, 1403, 1391, 1365, 1287, 1248, 1227, 1170, 1129, 1045, 1020, 1004 cm^{-1}; **HRMS** (ESI): *m/z* calculated for $C_{27}H_{46}O_6N_5$ [*M*+H]⁺: 536.3443, found: 536.3435; $[\alpha]_D^{25}$ = –13.6 (c = 0.25, MeOH).

(*S*)-5-((*S*)-2-Acetamido-4-methylpentanamido)-6-oxo-6-((2-oxo-2-(prop-2-yn-1-ylamino)ethyl)amino)hexan-1-aminium chloride (25)

According to General Procedure H, tetrapeptide salt **25** (19 mg, 78%) was obtained from *N*-Boc-protected tetrapeptide **I24** (28 mg, 0.06 mmol).

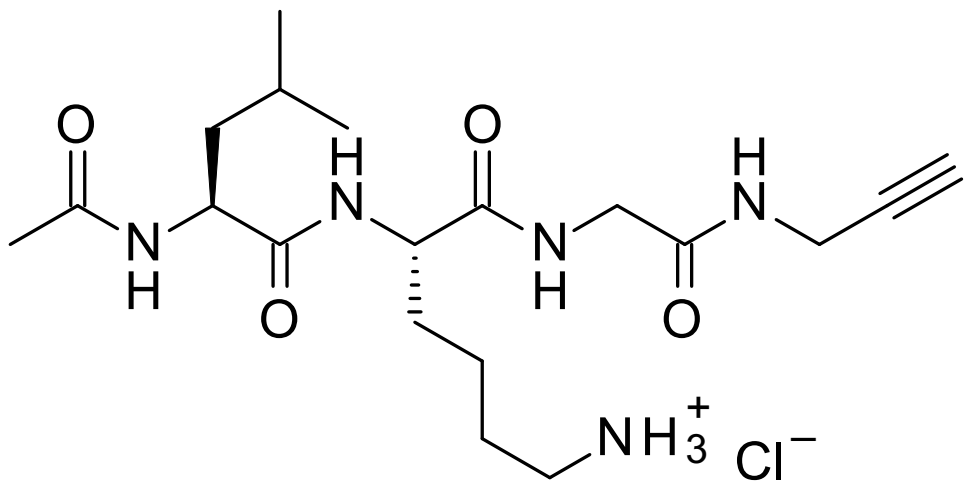

Off-white amorphous solid; **^{1}H NMR** (600 MHz, DMSO-*d*$_6$): δ = 8.26 (t, *J* = 5.5 Hz, 1H), 8.11 (t, *J* = 5.8 Hz, 1H), 8.05 (d, *J* = 7.8 Hz, 1H), 8.02 (d, *J* = 7.8 Hz, 1H), 7.80 (br, 3H), 4.26 (q, *J* = 7.6 Hz, 1H), 4.22-4.19 (m, 1H), 3.88 (ddd, *J* = 17.5, 5.6, 2.5 Hz, 1H), 3.84 (ddd, *J* = 17.5, 5.6, 2.5 Hz, 1H), 3.70 (dd, *J* = 16.6, 6.0 Hz, 1H), 3.66 (dd, *J* = 16.6, 5.9 Hz, 1H), 3.11 (t, *J* = 2.5 Hz, 1H), 2.78-2.70 (m, 2H), 1.84 (s, 3H), 1.75 – 1.66 (m, 1H), 1.64 – 1.58 (m, 1H), 1.57 – 1.50 (m, 3H), 1.43 (t, *J* = 7.3 Hz, 2H), 1.37 – 1.23 (m, 2H), 0.88 (d, *J* = 6.6 Hz, 3H), 0.84 ppm (d, *J* = 6.5 Hz, 3H); **^{13}C NMR** (150 MHz, DMSO-*d*$_6$): δ = 172.5, 171.7, 169.5, 168.5, 81.0, 73.1, 52.3, 51.2, 41.8, 40.5, 38.6, 31.0, 27.9, 26.5, 24.2, 23.1, 22.5, 22.1, 21.5 ppm; **IR** (solid): ṽ = 3277, 3058, 2980, 2960, 2873, 1633, 1542, 1470, 1438, 1372, 1343, 1290, 1244, 1161, 1129, 1090, 1030 cm^{-1}; **HRMS** (ESI): *m/z* calculated for $C_{19}H_{34}O_4N_5$ [*M*+H]$^+$: 396.2605, found: 396.2603; $[\alpha]_D^{25}$ = –37.4 (c = 0.25, MeOH).

(*S*)-5-((*S*)-2-Acetamido-3-cyclohexylpropanamido)-6-oxo-6-((2-oxo-2-(prop-2-yn-1-ylamino)ethyl)amino)hexan-1-aminium chloride (26)

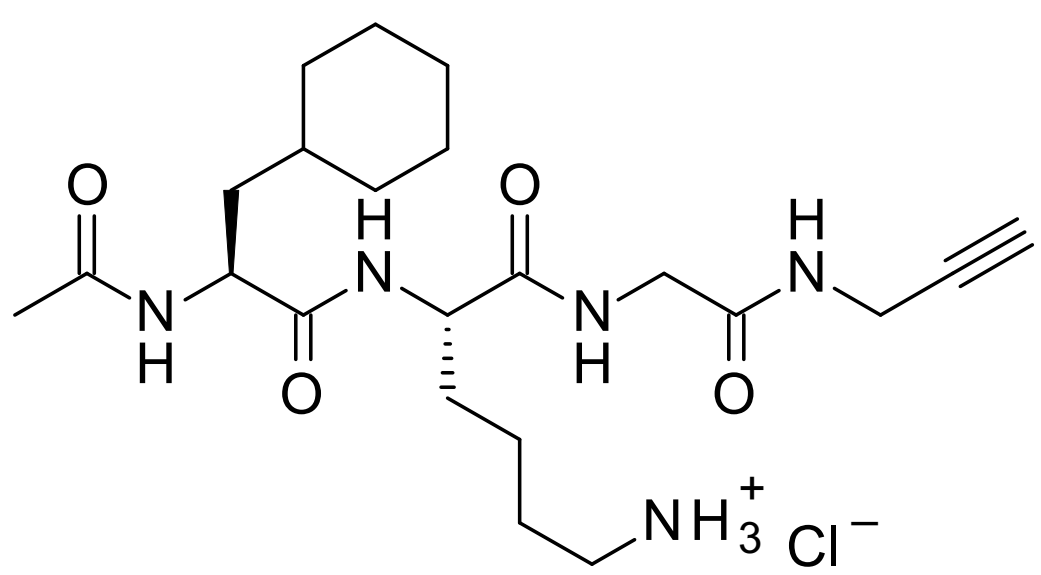

According to General Procedure H, tetrapeptide salt **26** (40 mg, apparent quant.) was obtained from *N*-Boc-protected tetrapeptide **I26** (40 mg, 0.08 mmol).

White amorphous solid; **^{1}H NMR** (600 MHz, DMSO-*d*$_6$): δ = 8.26 (t, *J* = 5.5 Hz, 1H), 8.09 (t, *J* = 5.8 Hz, 1H), 8.03 (d, *J* = 7.7 Hz, 1H), 8.00 (d, *J* = 7.8 Hz, 1H), 7.75 (br, 3H), 4.27 (ddd, *J* = 9.7, 7.7, 5.2 Hz, 1H), 4.23-4.19 (m, 1H), 3.88 (ddd, *J* = 17.5, 5.6, 2.5 Hz, 1H), 3.83 (ddd, *J* = 17.5, 5.6, 2.5 Hz, 1H), 3.70 (dd, *J* = 16.6, 5.8 Hz, 1H), 3.67 (dd, *J* = 16.6, 5.9 Hz, 1H), 3.11 (t, *J* = 2.5 Hz, 1H), 2.77 – 2.71 (m, 2H), 1.84 (s, 3H), 1.73 – 1.57 (m, 6H), 1.56 – 1.49 (m, 3H), 1.49 – 1.39 (m, 2H), 1.37 – 1.23 (m, 3H), 1.22 – 1.07 (m, 3H), 0.92 – 0.80 ppm (m, 2H); **^{13}C NMR** (150 MHz, DMSO-*d*$_6$): δ = 172.5, 171.7, 169.5, 168.5, 80.9, 73.1, 52.3, 50.5, 41.7, 39.0 (determined by HSQC), 38.6, 33.5, 33.2, 31.9, 31.0, 27.9, 26.5, 26.1, 25.8, 25.6, 22.5, 22.1 ppm; **IR** (solid): ṽ = 3279, 3055, 2926, 2855, 1641, 1538, 1468, 1441, 1372, 1345, 1289, 1256, 1244, 1228, 1160, 1092, 1039 cm^{-1}; **HRMS** (ESI): *m/z* calculated for $C_{22}H_{38}O_4N_5$ [*M*+H]$^+$: 436.2918, found: 436.2917; $[\alpha]_D^{25}$ = –24.1 (c = 0.25, MeOH).

(*S*)-5-((*R*)-2-Acetamido-4-methylpentanamido)-6-oxo-6-((2-oxo-2-(prop-2-yn-1-ylamino)ethyl)amino)hexan-1-aminium chloride (27)

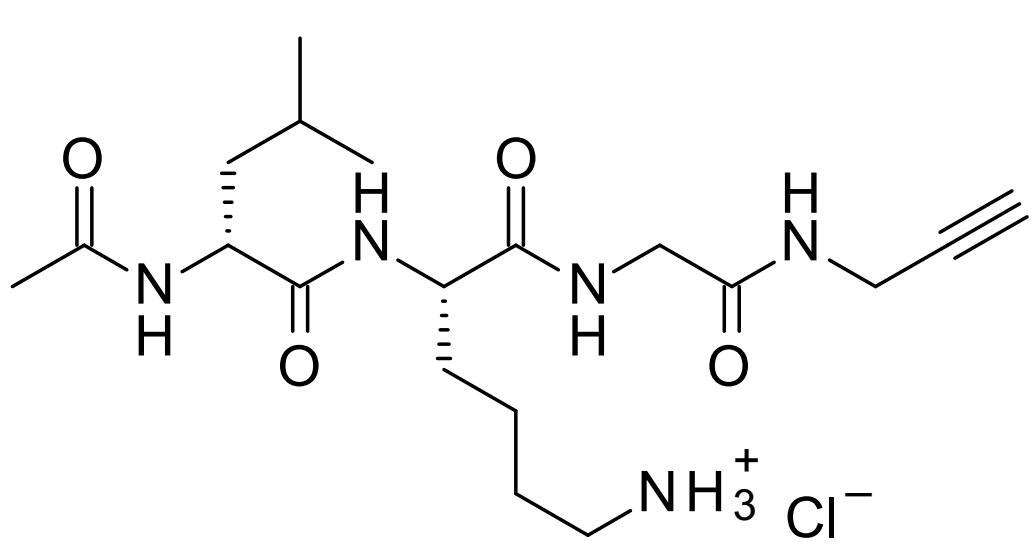

According to General Procedure H, tetrapeptide salt **27** (39 mg, apparent quant.) was obtained from *N*-Boc-protected tetrapeptide **I25** (40 mg, 0.08 mmol).

Pale yellow amorphous solid; **^{1}H NMR** (600 MHz, DMSO-*d*$_6$): δ = 8.29 (d, *J* = 7.9 Hz, 1H), 8.18 – 8.15 (m, 2H), 8.09 (d, *J* = 7.2 Hz, 1H), 7.75 (br, 3H), 4.25 (q, *J* = 7.5 Hz, 1H), 4.19-4.15 (m, 1H), 3.88 (ddd, *J* = 17.5, 5.6, 2.6 Hz, 1H) 3.84 (ddd, *J* = 17.5, 5.6, 2.6 Hz, 1H), 3.71 – 3.64 (m, 2H), 3.11

(t, *J* = 2.5 Hz, 1H), 2.78 – 2.69 (m, 2H), 1.84 (s, 3H), 1.74 – 1.68 (m, 1H), 1.60 – 1.48 (m, 4H), 1.43 (t, *J* = 7.3 Hz, 2H), 1.36 – 1.23 (m, 2H), 0.89 (d, *J* = 6.6 Hz, 3H), 0.85 ppm (d, *J* = 6.6 Hz, 3H); **^{13}C NMR** (150 MHz, DMSO-*d$_6$*): *δ* = 172.6, 171.8, 169.7, 168.4, 81.0, 73.1, 52.4, 51.6, 41.8, 40.6, 38.6, 30.7, 27.9, 26.4, 24.2, 22.8, 22.4, 22.1, 21.9 ppm; **IR** (solid): ṽ = 3278, 3054, 2980, 2962, 2934, 1651, 1633, 1542, 1471, 1457, 1437, 1374, 1340, 1283, 1251, 1224, 1160, 1125, 1089, 1030 cm^{-1}; **HRMS** (ESI): *m/z* calculated for $C_{19}H_{34}O_4N_5$ [*M*+H]$^+$: 396.2605, found: 396.2607; $[\boldsymbol{\alpha}]_D^{25}$ = –7.3 (c = 0.25, MeOH).

(*S*)-5-((*S*)-2-Acetamido-4-methylpentanamido)-6-((2-((cyanomethyl)amino)-2-oxoethyl)amino)-6-oxohexan-1-aminium chloride (28)

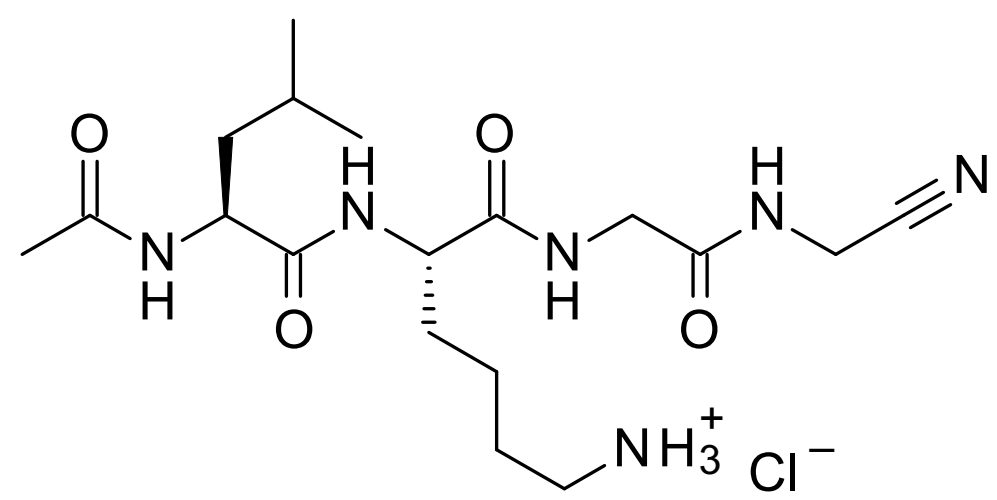

According to General Procedure G, tetrapeptide salt **28** (22 mg, apparent quant.) was obtained from N-Boc-protected tetrapeptide **I20** (25 mg, 0.05 mmol).

White amorphous solid; **^{1}H NMR** (600 MHz, DMSO-*d$_6$*): *δ* = 8.58 (t, *J* = 5.6 Hz, 1H), 8.21 (t, *J* = 5.9 Hz, 1H), 8.06 (d, *J* = 7.6 Hz, 1H), 8.05 (d, *J* = 7.8 Hz, 1H), 7.81 (br, 3H), 4.27 (q, *J* = 7.6 Hz, 1H), 4.23-4.19 (m, 1H), 4.14 (d, *J* = 5.6 Hz, 2H), 3.76 (dd, *J* = 16.8, 6.0 Hz, 1H), 3.72 (dd, *J* = 16.9, 5.8 Hz, 1H), 2.78 – 2.71 (m, 2H), 1.85 (s, 3H), 1.74 – 1.67 (m, 1H), 1.64 – 1.50 (m, 4H), 1.46-1.41 (m, 2H), 1.38 – 1.25 (m, 2H), 0.88 (d, *J* = 6.6 Hz, 3H), 0.84 ppm (d, *J* = 6.6 Hz, 3H); **^{13}C NMR** (150 MHz, DMSO-*d$_6$*): *δ* = 172.5, 171.9, 169.6, 169.5, 117.5, 52.3, 51.1, 41.7, 40.5, 38.6, 30.9, 27.0, 26.5, 24.2, 23.1, 22.5, 22.1, 21.5 ppm; **IR** (solid): ṽ = 3277, 3052, 2980, 2959, 2931, 2872, 1666, 1635, 1537, 1471, 1445, 1415, 1390, 1373, 1339, 1283, 1236, 1162, 1099, 1028 cm^{-1}; **HR-MS** (ESI): *m/z* calculated for $C_{18}H_{33}O_4N_6$ [*M*+H]$^+$: 397.2558, found: 397.2554; $[\boldsymbol{\alpha}]_D^{25}$ = -36.8 (c = 0.25, MeOH).

(*S*)-5-((*S*)-2-Acetamido-2-cyclohexylacetamido)-6-((2-((cyanomethyl)amino)-2-oxoethyl)amino)-6-oxohexan-1-aminium chloride (29)

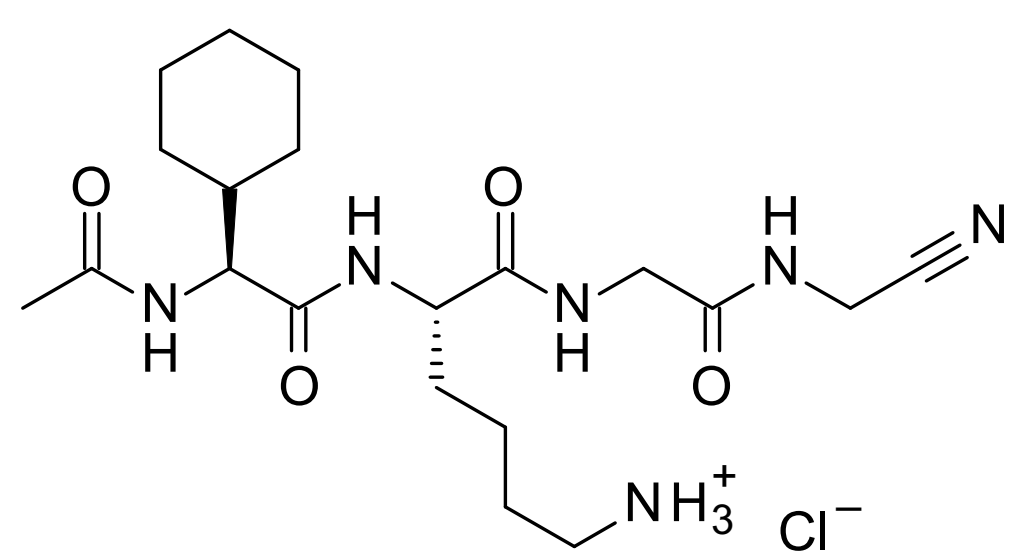

According to General Procedure G, tetrapeptide salt **29** (12 mg, 66%) was obtained from *N*-Boc-protected tetrapeptide **I23** (20 mg, 0.038 mmol).

Orange amorphous solid; **^{1}H NMR** (600 MHz, DMSO-*d$_6$*): *δ* = 8.59 (t, *J* = 5.6 Hz, 1H), 8.22 (t, *J* = 5.9 Hz, 1H), 8.11 (d, *J* = 7.3 Hz, 1H), 7.92 (d, *J* = 8.3 Hz, 1H), 7.80 (br, 3H), 4.21-4.17 (m, 1H), 4.14-4.12 (m, 3H), 3.76 (dd, *J* = 16.8, 6.1 Hz, 1H), 3.71 (dd, *J* = 16.8, 5.7 Hz, 1H), 2.75-2.72 (app.t, 2H), 1.86 (s, 3H), 1.71-1.51 (m, 10H), 1.36-1.27 (m, 2H), 1.15-1.06 (m, 3H), 1.02-0.94 ppm (m, 2H); **^{13}C NMR** (150 MHz, DMSO-*d$_6$*): *δ* = 171.9, 171.4, 169.6, 169.5, 117.5, 57.4, 52.6, 41.7, 39.6 (determined by HSQC), 38.6, 30.8, 29.0, 28.2, 27.0, 26.6, 25.8, 25.5(8), 25.5(6), 22.5, 22.1 ppm; **IR** (solid): ṽ = 3279, 3057, 2927, 2854, 1632, 1546, 1468, 1443, 1403, 1373, 1344, 1291, 1256, 1242, 1161, 1130, 1101, 1032 cm^{-1}; **HRMS** (ESI): *m/z* calculated for $C_{20}H_{35}O_4N_6$ [*M*+H]$^+$: 423.2714, found: 423.2709; $[\boldsymbol{\alpha}]_D^{25}$ = –32.0 (c = 0.125, MeOH).

(*S*)-5-((*R*)-2-Acetamido-4-methylpentanamido)-6-((2-((cyanomethyl)amino)-2-oxoethyl)amino)-6-oxohexan-1-aminium chloride (30)

According to General Procedure G, tetrapeptide salt **30** (22 mg, apparent quant.) was obtained from *N*-Boc-protected tetrapeptide **I21** (25 mg, 0.05 mmol).

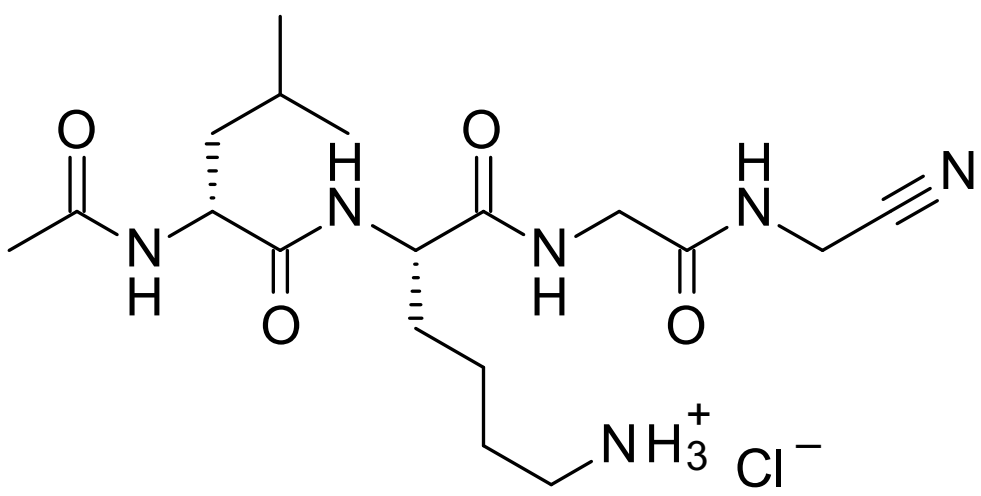

Pale yellow amorphous solid; **^{1}H NMR** (600 MHz, DMSO-*d$_6$*): *δ* = 8.52 (t, *J* = 5.6 Hz, 1H), 8.37 (d, *J* = 7.8 Hz, 1H), 8.27 (t, *J* = 6.0 Hz, 1H), 8.11 (d, *J* = 7.2 Hz, 1H), 7.86 (br, 3H), 4.26 (q, *J* = 7.4 Hz, 1H), 4.20-4.16 (m, 1H), 4.14-4.13 (m, 2H), 3.76 (dd, J = 16.7, 5.9 Hz, 1H), 3.70 (dd, *J* = 16.7, 5.9 Hz, 1H), 2.77– 2.69 (m, 2H), 1.84 (s, 3H), 1.75 – 1.69 (m, 1H), 1.60 – 1.49 (m, 3H), 1.44 (t, *J* = 7.3 Hz, 2H), 1.36 – 1.23 (m, 2H), 0.90 (d, *J* = 6.6 Hz, 3H), 0.85 ppm (d, *J* = 6.6 Hz, 3H); **^{13}C NMR** (150 MHz, DMSO-*d$_6$*): *δ* = 172.7, 171.9, 169.7, 169.5, 117.5, 52.4, 51.6, 41.7, 40.6, 38.5, 30.6, 27.0, 26.4, 24.2, 22.8, 22.4, 22.1, 21.9 ppm; **IR** (solid): ṽ = 3271, 3051, 2955, 2930, 2872, 1657, 1650, 1546, 1535, 1468, 1443, 1412, 1372, 1344, 1284, 1244, 1164, 1100, 1029 cm^{-1}; **HRMS** (ESI): *m/z* calculated for $C_{18}H_{33}O_4N_6$ [*M*+H]$^+$: 397.2558, found: 397.2555; $[\alpha]_D^{25}$ = –8.1 (c = 0.25, MeOH).

(*S*)-5-((*S*)-2-Acetamido-3-cyclohexylpropanamido)-6-((2-((cyanomethyl)amino)-2-oxoethyl)amino)-6-oxohexan-1-aminium chloride (31)

According to General Procedure G, tetrapeptide salt **31** (20 mg, 88%) was obtained from *N*-Boc-protected tetrapeptide **I22** (25 mg, 0.05 mmol).

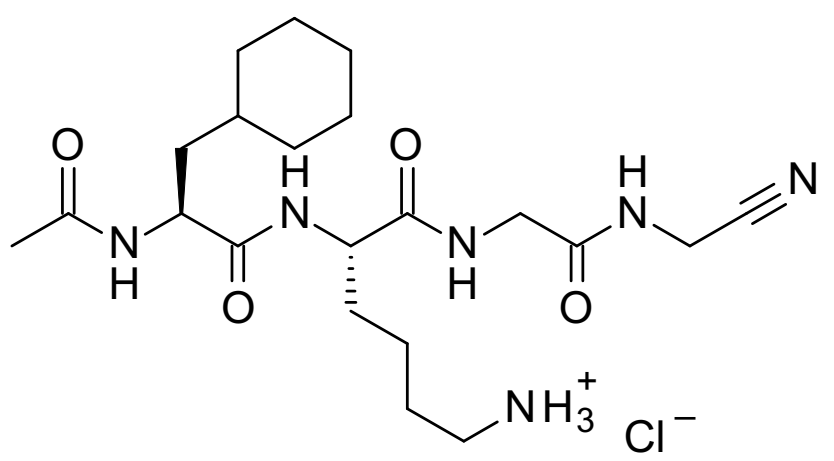

Off-white amorphous solid; **^{1}H NMR** (600 MHz, DMSO-*d$_6$*): *δ* = 8.58 (t, *J* = 5.6 Hz, 1H), 8.21 (t, *J* = 5.9 Hz, 1H), 8.06 – 8.04 (m, 2H), 7.83 (br, 3H), 4.27 (ddd, *J* = 9.8, 7.8, 5.1 Hz, 1H), 4.23-4.20 (m, 1H), 4.14 (d, *J* = 5.7 Hz, 2H), 3.76 (dd, *J* = 16.9, 6.1 Hz, 1H), 3.72 (dd, *J* = 17.0, 5.9 Hz, 1H), 2.76 – 2.71 (m, 2H), 1.85 (s, 3H), 1.74 – 1.50 (m, 9H), 1.50 – 1.39 (m, 2H), 1.37 – 1.23 (m, 3H), 1.21 – 1.07 (m, 3H), 0.92 – 0.80 ppm (m, 2H); **^{13}C NMR** (150 MHz, DMSO-*d$_6$*): *δ* = 172.6, 171.9, 169.5(4), 169.5(2), 117.5, 52.3, 50.5, 41.7, 39.0 (determined by HSQC), 38.6, 33.5, 33.2, 31.8, 30.9, 27.0, 26.5, 26.1, 25.8, 25.6, 22.5, 22.1 ppm; **IR** (solid): ṽ = 3274, 3060, 2981, 2924, 2852, 1666, 1635, 1540, 1474, 1448, 1417, 1393, 1375, 1354, 1251, 1232, 1179, 1152, 1119, 1074, 1023 cm^{-1}; **HRMS** (ESI): *m/z* calculated for $C_{21}H_{37}O_4N_6$ [*M*+H]$^+$: 437.2871, found: 437.2871; $[\alpha]_D^{25}$ = –24.1 (c = 0.25, MeOH).

2,5-Dioxopyrrolidin-1-yl-(((9*H*-fluoren-9-yl)methoxy)carbonyl)-*L*-leucinate (I27)

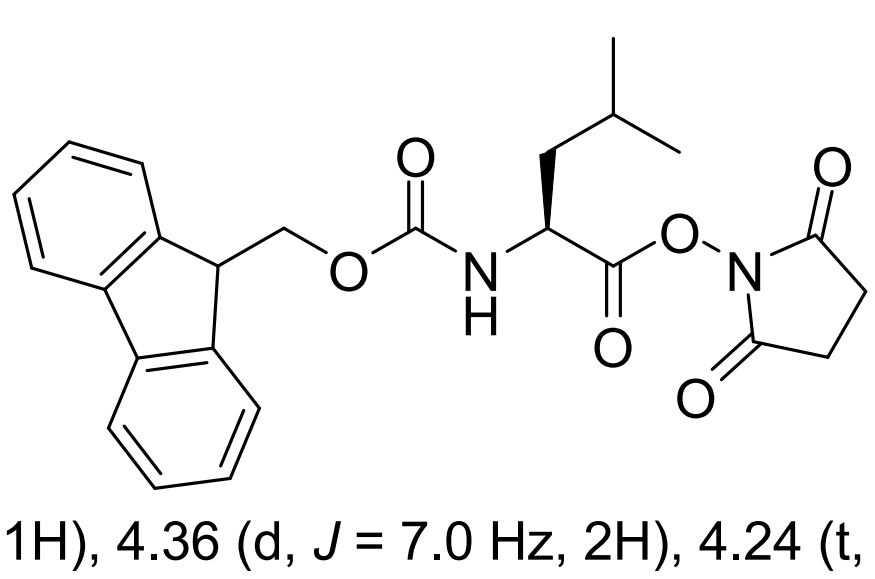

According to General Procedure A, ester **I27** (1.68 g, 65%) was obtained from commercially sourced (((9*H*-fluoren-9-yl)methoxy)carbonyl)-*L*-leucine (2.00 g, 5.7 mmol).

Characteristic analytical data: **^{1}H NMR** (400 MHz, DMSO-*d$_6$*): δ = 8.11 (d, *J* = 8.0 Hz, 1H), 7.89 (d, *J* = 7.6 Hz, 2H), 7.70 (dd, *J* = 7.5 Hz, 2H), 7.42 (dd, *J* = 7.9, 6.8 Hz, 2H), 7.32 (tt, *J* = 7.4 Hz, 2H), 4.42 (ddd, *J* = 10.3, 8.0, 4.5 Hz, 1H), 4.36 (d, *J* = 7.0 Hz, 2H), 4.24 (t, *J* = 7.0 Hz, 1H), 2.80 (s, 4H), 1.76 (td, *J* = 10.4, 4.4 Hz, 2H), 1.62 (td, *J* = 10.4, 6.0 Hz, 1H), 0.90 ppm (dd, *J* = 6.2 Hz, 6H); **^{13}C NMR** (101 MHz, DMSO-*d$_6$*): δ = 170.0, 169.1, 156.0, 143.8, 143.6, 140.7, 127.7, 127.1, 125.2, 120.1, 65.8, 50.5, 46.6, 38.9, 25.5, 24.1, 22.7, 21.0 ppm; **HRMS** (ESI): *m/z* calculated for $C_{25}H_{27}N_2O_6$ [*M*+H]$^+$: 451.1864, found: 451.1874.

N^2-((((9*H*-Fluoren-9-yl)methoxy)carbonyl)-*L*-leucyl)-N^6-(*tert*-butoxycarbonyl)-*L*-lysine (I28)

According to General Procedure B, dipeptide **I28** (3.77 g, 97%) was obtained from *N*-hydroxysuccinimide ester **I27** (3.00 g, 6.7 mmol) and commercially sourced L-Lys(Boc)-OH (2.47 g, 10.0 mmol).

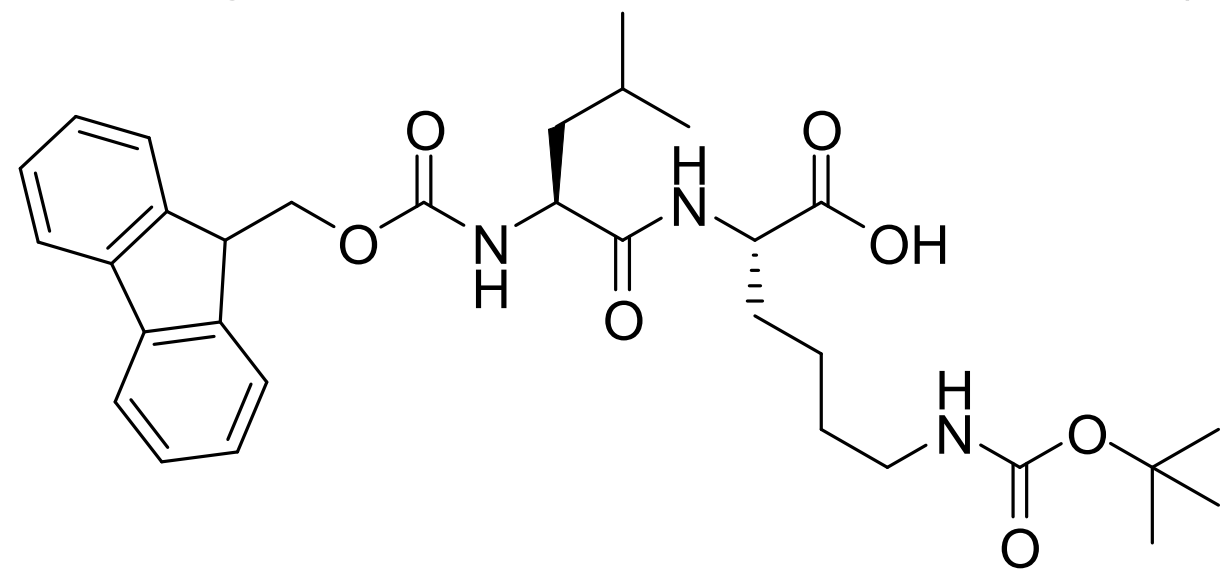

Characteristic analytical data: **^{1}H NMR** (400 MHz, DMSO-*d$_6$*): δ = δ 12.48 (s, 1H), 8.02 (d, *J* = 7.6 Hz, 1H), 7.88 (d, *J* = 7.5 Hz, 2H), 7.72 (t, *J* = 6.7 Hz, 2H), 7.47 (d, *J* = 8.5 Hz, 1H), 7.41 (td, *J* = 7.5, 1.2 Hz, 2H), 7.31 (tdd, *J* = 7.4, 1.2 Hz, 2H), 6.72 (t, *J* = 5.4 Hz, 1H), 4.34 – 4.17 (m, 3H), 4.09 (m, 2H), 2.87 (q, *J* = 6.5 Hz, 2H), 1.71 – 1.49 (m, 3H), 1.44 (m, 2H), 1.34 (s, 9H), 1.33 – 1.27 (m, 4H), 0.87 ppm (dd, *J* = 6.5 Hz, 6H); **^{13}C NMR** (101 MHz, DMSO-*d$_6$*): δ = 173.5, 172.4, 155.9, 155.5, 144.0, 143.7, 140.7, 127.6, 127.0, 125.3, 120.1, 77.3, 65.5, 52.8, 51.8, 46.7, 40.8, 39.2, 30.7, 29.1, 28.3, 24.1, 23.1, 22.8, 21.5 ppm; **HRMS** (ESI): *m/z* calculated for $C_{32}H_{44}N_3O_7$ [*M*+H]$^+$: 582.3174, found: 582.3164.

***tert*-Butyl-(2-oxo-2-(prop-2-yn-1-ylamino)ethyl)carbamate (I29)**

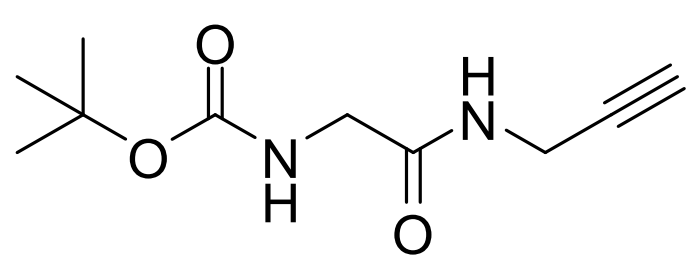

According to General Procedure C, alkyne **I29** (0.94 g, 80%) was obtained from commercially sourced *N*-Boc-glycine (1.1 g, 6.1 mmol) and commercially sourced propargylamine hydrochloride (0.50 g, 5.5 mmol). The characteristic analytical data of **I29** are consistent with those reported(3–6).

Characteristic analytical data: **^{1}H NMR** (400 MHz, DMSO-*d$_6$*): δ = 8.21 (t, *J* = 5.6 Hz, 1H), 6.94 (t, *J* = 6.2 Hz, 1H), 3.85 (dd, *J* = 5.6, 2.5 Hz, 2H), 3.51 (d, *J* = 5.9 Hz, 2H), 3.09 (t, *J* = 2.5 Hz, 1H), 1.38 ppm (s, 9H); **^{13}C NMR** (101 MHz, DMSO-*d$_6$*): δ = 169.2, 155.8, 81.1, 78.0, 72.9, 43.0, 28.2, 27.8 ppm; **HRMS** (ESI): *m/z* calculated for $C_{10}H_{16}N_2O_3Na$ [*M*+Na]$^+$: 235.1053, found: 235.1049.

***tert*-Butyl-(2-(but-2-yn-1-ylamino)-2-oxoethyl)carbamate (I30)**

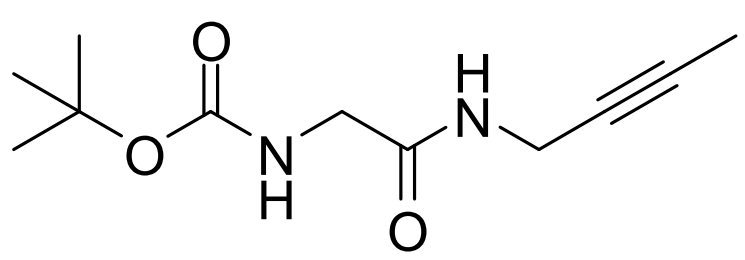

According to General Procedure C, alkyne **I30** (231 mg, 54%) was obtained from commercially sourced *N*-Boc-glycine (365 mg, 2.09 mmol) and commercially sourced but-2-yn-1-amine hydrochloride (200 mg, 1.90 mmol).

Characteristic analytical data: **^{1}H NMR** (400 MHz, DMSO-*d$_6$*): δ = 8.13 (t, *J* = 5.5 Hz, 1H), 6.92 (t, *J* = 6.2 Hz, 1H), 3.81 (dq, *J* = 4.9, 2.5 Hz, 2H), 3.50 (d, *J* = 6.2 Hz, 2H), 1.76 (t, *J* = 2.5 Hz, 3H), 1.38 ppm (s, 9H); **^{13}C NMR** (151 MHz, DMSO-*d$_6$*): δ = 169.0, 155.8, 78.0, 77.9, 76.4, 43.0, 28.2, 28.1, 3.0 ppm; **HRMS** (ESI): *m/z* calculated for $C_{11}H_{18}N_2O_3Na$ [*M*+Na]$^+$: 249.1210, found: 249.1209.

***tert*-Butyl-(2-((1-ethynylcyclopropyl)amino)-2-oxoethyl)carbamate (I31)**

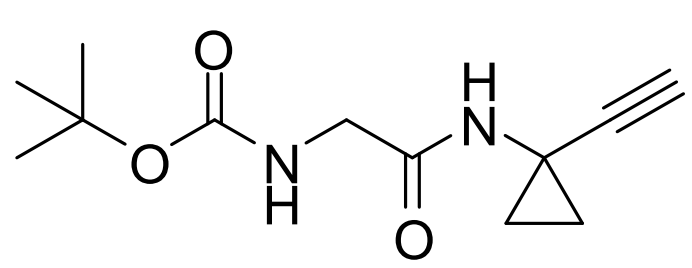

According to General Procedure C, alkyne **I31** (247 mg, 58%) was obtained from commercially sourced *N*-Boc-glycine (345 mg, 1.97 mmol) and commercially sourced 1-ethynylcyclopropanamine hydrochloride (209 mg, 1.78 mmol).

Characteristic analytical data: **^{1}H NMR** (400 MHz, DMSO-*d$_6$*): δ = 8.42 (s, 1H), 6.84 (t, *J* = 6.1 Hz, 1H), 3.44 (d, *J* = 5.6 Hz, 2H), 2.93 (s, 1H), 1.37 (s, 9H), 1.06 (q, *J* = 4.7 Hz, 2H), 0.91 ppm (q, *J* = 4.6 Hz, 2H); **^{13}C NMR** (151 MHz, DMSO-*d$_6$*): δ = 169.6, 155.8, 86.4, 78.0, 68.5, 42.9, 28.2, 21.8, 16.8 ppm; **HRMS** (ESI): *m/z* calculated for $C_{12}H_{19}N_2O_3$ [*M*+H]$^+$: 239.1390, found: 239.1397.

tert-Butyl-(2-oxo-2-((3-phenylprop-2-yn-1-yl)amino)ethyl)carbamate (I32)

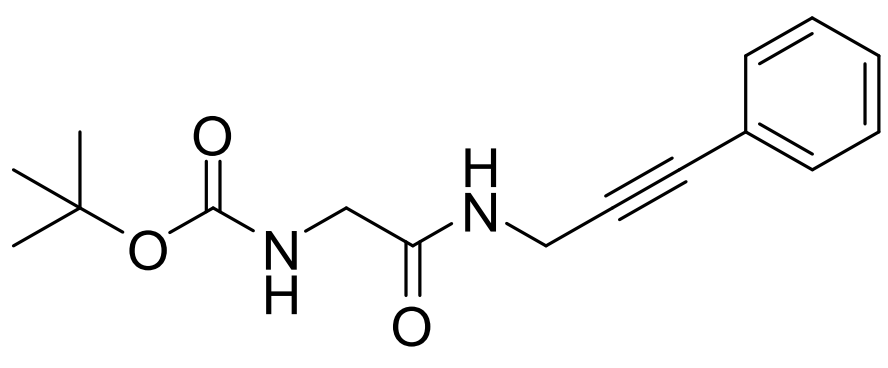

According to General Procedure C, alkyne **I32** (118 mg, 85%) was obtained from commercially sourced *N*-Boc-glycine (94 mg, 0.53 mmol) and commercially sourced 3-phenyl-2-propyn-1-amine hydrochloride (80 mg, 0.48 mmol). The characteristic analytical data of **I32** are consistent with those reported(7).

Characteristic analytical data: **^{1}H NMR** (400 MHz, DMSO-d_6): δ = 8.32 (t, *J* = 5.5 Hz, 1H), 7.45 – 7.33 (m, 5H), 6.97 (t, *J* = 6.2 Hz, 1H), 4.13 (d, *J* = 5.5 Hz, 2H), 3.55 (d, *J* = 6.2 Hz, 2H), 1.38 ppm (s, 9H); **^{13}C NMR** (151 MHz, DMSO-d_6): δ = 169.3, 155.8, 131.4, 128.7, 128.6, 122.3, 87.1, 81.6, 78.1, 43.1, 28.6, 28.2 ppm; **HRMS** (ESI): *m/z* calculated for $C_{16}H_{20}N_2O_3Na$ $[M+Na]^+$: 311.1366, found: 311.1372.

(9*H*-Fluoren-9-yl)methyl-((*S*)-1-(((*S*)-2,2-dimethyl-4,11,14-trioxo-3-oxa-5,12,15-triazaoctadec-17-yn-10-yl)amino)-4-methyl-1-oxopentan-2-yl)carbamate (I38)

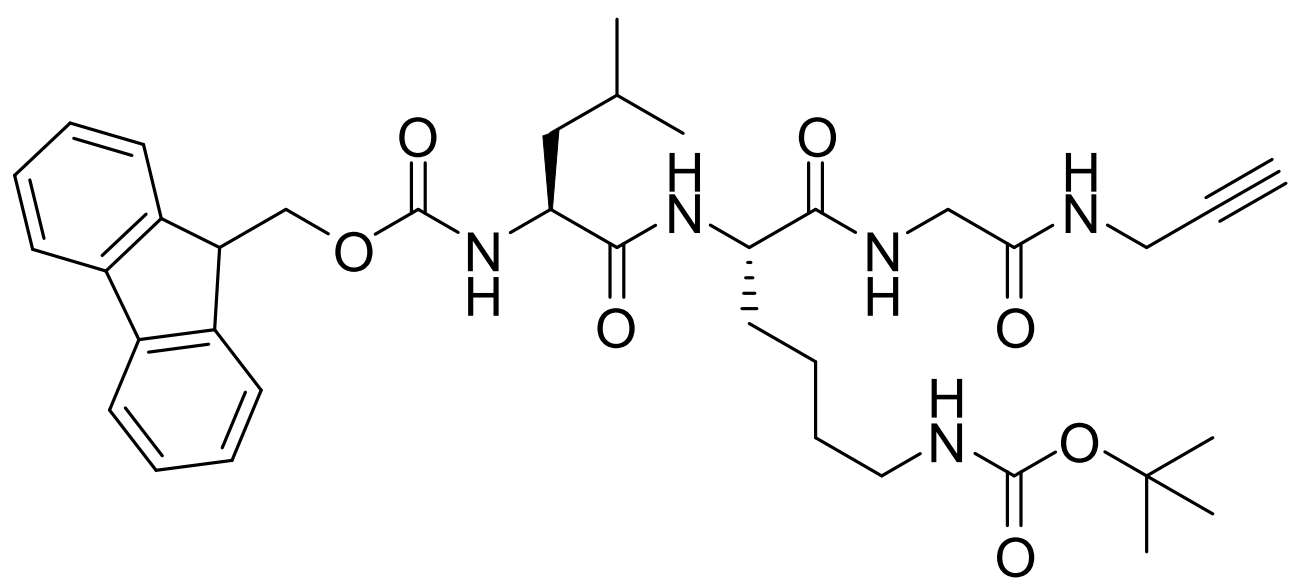

According to General Procedure D, amine hydrochloride **I33** was obtained *in situ* from *N*-Boc-protected peptide **I29** (0.10 mg, 0.48 mmol) and was directly used in the following step: According to General Procedure E, tetrapeptide **I38** (0.33 g, apparent quantitative) was obtained from dipeptide **I28** (0.27 g, 0.46 mmol) and amine hydrochloride **I33**.

Characteristic analytical data: **^{1}H NMR** (400 MHz, DMSO-d_6): δ = 8.24 (t, *J* = 5.6 Hz, 1H), 8.18 (t, *J* = 5.9 Hz, 1H), 7.96 (d, *J* = 7.5 Hz, 1H), 7.92 – 7.85 (m, 2H), 7.72 (t, *J* = 6.9 Hz, 2H), 7.52 (d, *J* = 8.4 Hz, 1H), 7.41 (td, *J* = 7.5, 1.1 Hz, 2H), 7.32 (tt, *J* = 7.4, 1.4 Hz, 2H), 6.71 (t, *J* = 5.7 Hz, 1H), 4.37 – 4.15 (m, 4H), 4.11 – 3.98 (m, 1H), 3.86 (dt, *J* = 6.0, 2.2 Hz, 2H), 3.77 – 3.60 (dd, *J* = 6.3 Hz, 2H), 3.09 (t, *J* = 2.5 Hz, 1H), 2.86 (q, *J* = 6.6 Hz, 2H), 1.67 – 1.55 (m, 2H), 1.55 – 1.37 (m, 3H), 1.41 – 1.13 (m, 4H), 1.34 (s, 9H), 0.87 ppm (dd, *J* = 13.1, 6.5 Hz, 6H); **^{13}C NMR** (101 MHz, DMSO-d_6): δ = 172.5, 171.9, 168.5, 155.9, 155.5, 144.0, 143.7, 140.7, 127.6, 127.1, 125.3, 120.1, 81.0, 77.3, 73.0, 65.5, 53.0, 52.7, 46.7, 41.8, 40.6, 39.5, 31.6, 29.2, 28.3, 27.9, 24.2, 23.1, 22.5, 21.4 ppm; **HRMS** (ESI): *m/z* calculated for $C_{37}H_{50}N_5O_7$ $[M+H]^+$: 676.3705, found: 676.3696.

(9*H*-Fluoren-9-yl)methyl-((*S*)-1-(((*S*)-2,2-dimethyl-4,11,14-trioxo-3-oxa-5,12,15-triazanonadec-17-yn-10-yl)amino)-4-methyl-1-oxopentan-2-yl)carbamate (I39)

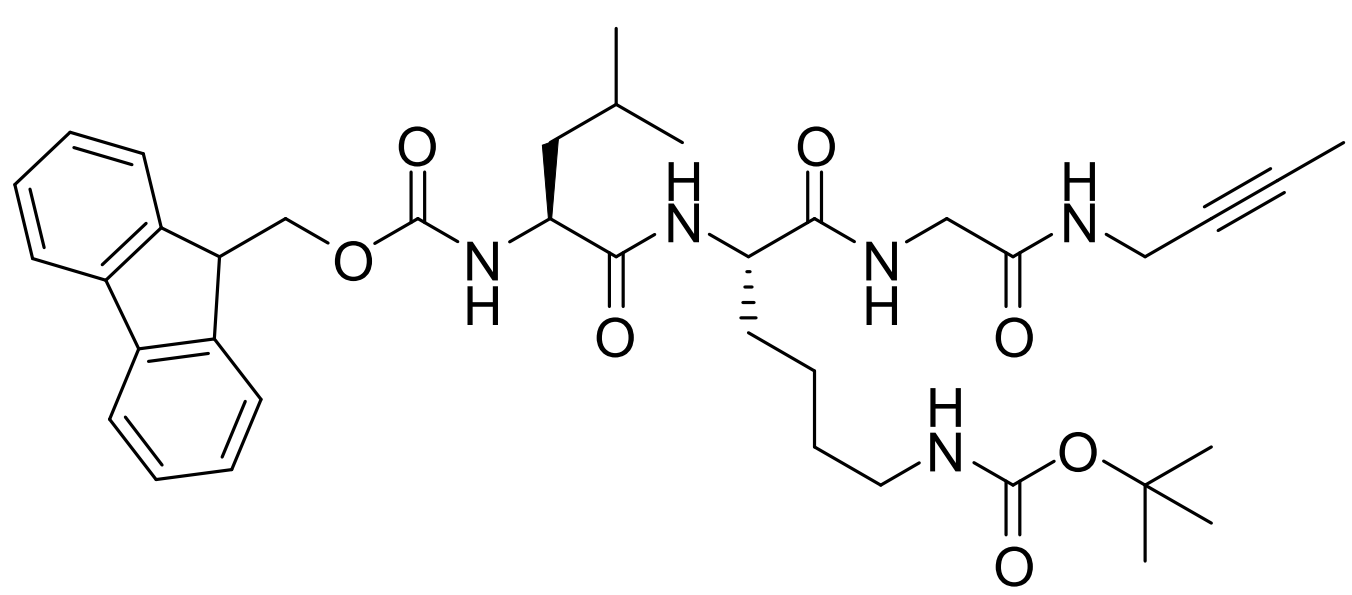

According to General Procedure D, amine hydrochloride **I34** was obtained *in situ* from *N*-Boc-protected peptide **I30** (0.23 g, 1.02 mmol) and was directly used in the following step: According to General Procedure E, tetrapeptide **I39** (0.76 g, apparent quantitative) was obtained from dipeptide **I28** (0.60 g, 1.03 mmol) and amine hydrochloride **I34**.

Characteristic analytical data: **^{1}H NMR** (400 MHz, DMSO-d_6): δ = 8.14 (s, 2H), 7.93 (d, *J* = 7.4 Hz, 1H), 7.89 (d, *J* = 7.6 Hz, 2H), 7.71 (t, *J* = 6.9 Hz, 2H), 7.50 (d, *J* = 8.5 Hz, 1H), 7.41 (t, *J* = 7.4 Hz, 2H), 7.32 (t, *J* = 7.5 Hz, 2H), 6.71 (t, *J* = 6.9 Hz, 1H), 4.36 – 4.19 (m, 4H), 4.10 – 4.00 (m, 1H), 3.81 (dt, *J* = 5.3, 2.6 Hz, 2H), 3.75 – 3.58 (m, 2H), 2.86 (d, *J* = 7.2 Hz, 2H), 1.75 (q, *J* = 2.4 Hz, 3H), 1.65 – 1.58 (m, 2H), 1.51 – 1.35 (m, 3H), 1.34 (s, 9H), 1.32 – 1.17 (m, 4H), 0.86 ppm (dd, *J* = 13.2, 6.5 Hz, 6H); **^{13}C NMR** (151 MHz, DMSO-d_6): δ = 172.4, 171.9, 168.4, 155.9, 155.6,

142.6, 139.4, 137.4, 128.9, 127.3, 121.4, 120.0, 109.8, 78.0, 77.3, 76.2, 65.5, 52.6, 52.2, 46.7, 43.7, 41.8, 40.5, 39.2, 31.8, 29.2, 28.7, 28.3, 24.0, 23.3, 23.1, 22.5, 21.7, 3.0 ppm; **HRMS** (ESI): *m/z* calculated for $C_{38}H_{52}N_5O_7$ [*M*+H]$^+$: 690.3861, found: 690.3833.

(9*H*-Fluoren-9-yl)methyl-((*S*)-1-(((*S*)-6-((*tert*-butoxycarbonyl)amino)-1-((2-((1-ethynylcyclopropyl)amino)-2-oxoethyl)amino)-1-oxohexan-2-yl)amino)-4-methyl-1-oxopentan-2-yl)carbamate (I40)

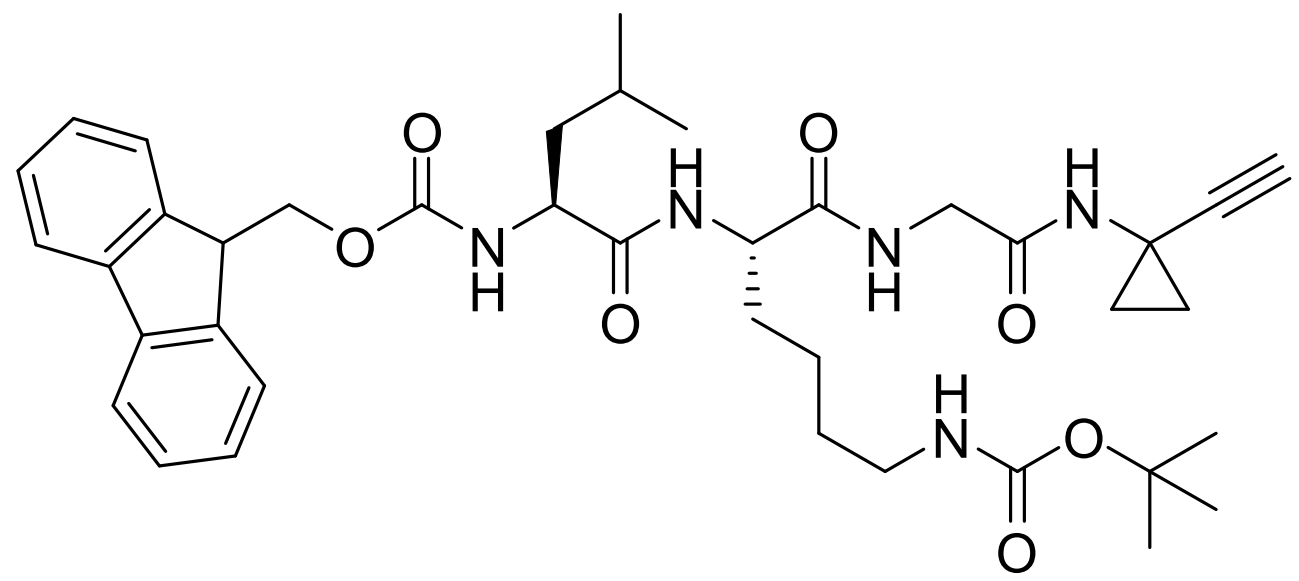

A solution of *N*-Boc-protected peptide **I31** (1.0 equiv., 0.25 g, 1.04 mmol) in commercially sourced formic acid (0.12 M) was stirred under an atmosphere of N_2 gas overnight (16 h). The reaction was concentrated under reduced pressure (30 °C) and the crude residue was redissolved in tetrahydrofuran/water (3:1 v/v, 0.13 M). The solution was basified with AmberLite™ HPR4800 OH (23-27 mesh) to pH 8 and filtered to afford amine hydrochloride **I35** which was directly used in the following step: According to General Procedure E, tetrapeptide **I40** (0.65 g, 90%) was obtained from dipeptide **I28** (0.61 g, 1.04 mmol) and amine hydrochloride **I35**.

Characteristic analytical data: **^{1}H NMR** (400 MHz, DMSO-*d*$_6$): δ = 8.35 (s, 1H), 8.11 (t, *J* = 5.8 Hz, 1H), 7.96 (d, *J* = 7.5 Hz, 1H), 7.89 (d, *J* = 7.6 Hz, 2H), 7.77 – 7.68 (m, 2H), 7.52 (d, *J* = 8.3 Hz, 1H), 7.41 (t, *J* = 7.4 Hz, 2H), 7.32 (t, *J* = 7.4 Hz, 2H), 6.71 (d, *J* = 5.8 Hz, 1H), 4.36 – 4.14 (m, 4H), 4.10 – 3.98 (m, 1H), 3.75 – 3.46 (dd, *J* = 5.5 Hz, 2H), 2.93 (s, 1H), 2.86 (q, *J* = 6.9 Hz, 2H), 1.72 – 1.56 (m, 2H), 1.55 – 1.40 (m, 3H), 1.38 – 1.12 (m, 4H), 1.34 (s, 9H), 1.06 (dq, *J* = 5.2, 2.7 Hz, 2H), 0.96 – 0.77 ppm (m, 8H); **^{13}C NMR** (151 MHz, DMSO-*d*$_6$): δ = 171.9, 168.8, 167.5, 155.6, 147.1, 143.9, 140.6, 140.5, 127.8, 127.3, 124.0, 120.7, 77.4, 73.2, 68.6, 65.1, 52.9, 52.7, 46.2, 41.8, 40.9, 39.5, 31.8, 28.9, 28.3, 24.0, 23.3, 22.4, 21.8, 21.7(5), 16.8 ppm; **HRMS** (ESI): *m/z* calculated for $C_{39}H_{52}N_5O_7$ [*M*+H]$^+$: 702.3861, found: 702.3864.

(9*H*-Fluoren-9-yl)methyl-((*S*)-1-(((*S*)-6-((*tert*-butoxycarbonyl)amino)-1-((2-((1-(1-chlorovinyl)cyclopropyl)amino)-2-oxoethyl)amino)-1-oxohexan-2-yl)amino)-4-methyl-1-oxopentan-2-yl)carbamate (I41)

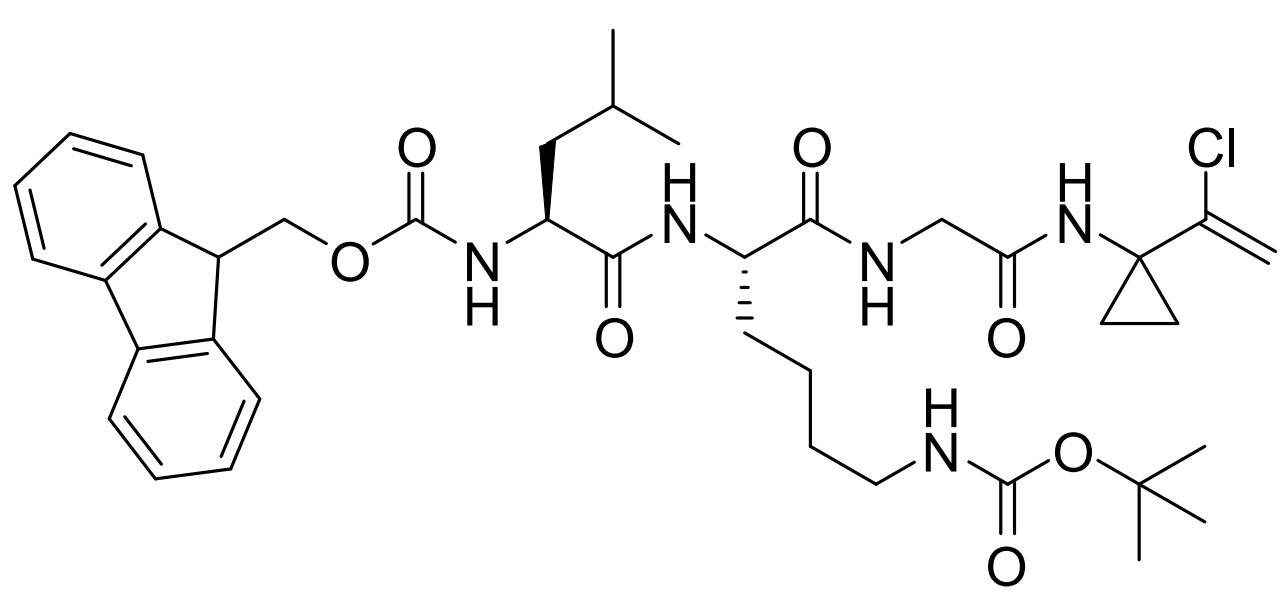

According to General Procedure D, amine hydrochloride **I36** was obtained *in situ* from *N*-Boc-protected peptide **I31** (82.9 mg, 0.348 mmol) and was directly used in the following step: According to General Procedure E, tetrapeptide **I41** (244 mg, apparent quantitative, 1:1 mixture of **I41** and **I40** as per ^{1}H NMR analysis) was obtained from dipeptide **3** (205 mg, 0.35 mmol) and amine hydrochloride **I36** (mixture of **I36** and **I35**).

Characteristic analytical data for **I41**: **^{1}H NMR** (400 MHz, DMSO-*d*$_6$): δ = 8.35 (s, 1H), 8.17 (t, *J* = 5.7 Hz, 1H), 7.98 (d, *J* = 7.5 Hz, 1H), 7.89 (d, *J* = 7.6 Hz, 2H), 7.72 (t, *J* = 6.7 Hz, 2H), 7.50 (d, *J* = 7.2 Hz, 1H), 7.41 (t, *J* = 7.5 Hz, 2H), 7.36 – 7.28 (m, 2H), 6.71 (s, 1H), 5.43 (d, *J* = 1.8 Hz, 1H), 5.22 (d, *J* = 1.8 Hz, 1H), 4.36 – 4.13 (m, 4H), 4.11 – 4.00 (m, 1H), 3.70 – 3.55 (m, 2H), 2.86 (d, *J* = 7.7 Hz, 2H), 1.70 – 1.56 (m, 2H), 1.55 – 1.40 (m, 3H), 1.40 – 1.20 (m, 4H), 1.34 (s, 9H), 1.07 (d, *J* = 6.6 Hz, 2H), 0.92 (d, *J* = 6.6 Hz, 2H), 0.89 – 0.81 ppm (m, 6H); **^{13}C NMR** (151 MHz, DMSO-*d*$_6$): δ = 170.8, 170.4, 155.9, 155.5, 144.0, 143.7, 140.7, 139.4, 128.9, 127.3, 125.3, 120.1, 112.0, 77.3, 65.5, 53.0, 52.2, 46.7, 41.9, 40.6, 39.2, 36.0, 31.7, 29.2, 28.3, 24.1, 23.1, 22.6, 21.4, 16.8 ppm; **HRMS** (ESI): *m/z* calculated for $C_{39}H_{53}ClN_5O_7$ (**I41**) [*M*+H]$^+$: 738.3628, found: 738.3646; *m/z* calculated for $C_{39}H_{53}N_5O_7$ (**I40**) [*M*+H]$^+$: 702.3861, found: 702.3887.

(9*H*-Fluoren-9-yl)methyl-((*S*)-1-(((*S*)-2,2-dimethyl-4,11,14-trioxo-18-phenyl-3-oxa-5,12,15-triazaoctadec-17-yn-10-yl)amino)-4-methyl-1-oxopentan-2-yl)carbamate (I42)

According to General Procedure D, alkyne hydrochloride salt **I37** was obtained *in situ* from *N*-Boc-protected peptide **I32** (0.25 g, 0.86 mmol) and was directly used in the following step: According to General Procedure E, tetrapeptide **I42** (0.61 g, 89%) was obtained from dipeptide **I28** (0.50 g, 0.86 mmol) and amine hydrochloride **I37**.

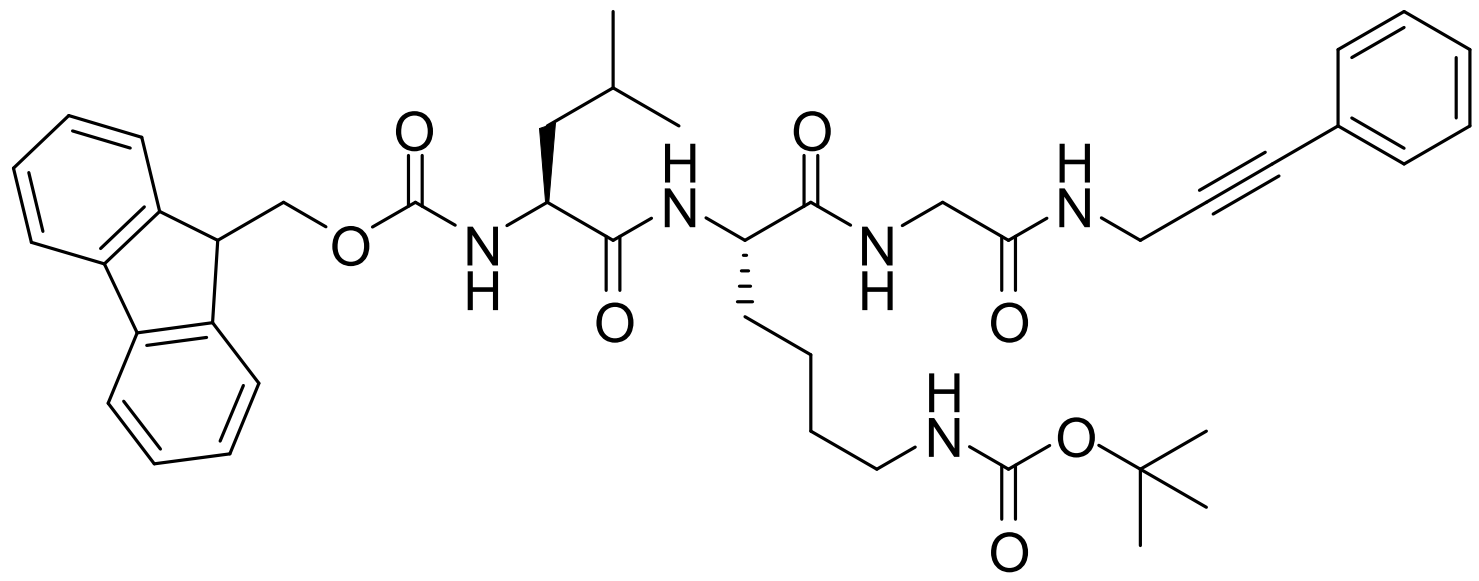

Characteristic analytical data: ^{1}**H NMR** (400 MHz, DMSO-*d*$_6$): δ = 8.34 (t, *J* = 5.5 Hz, 1H), 8.23 – 8.12 (m, 1H), 7.94 (d, *J* = 7.7 Hz, 1H), 7.88 (d, *J* = 7.5 Hz, 2H), 7.71 (t, *J* = 6.8 Hz, 2H), 7.50 (d, *J* = 8.7 Hz, 1H), 7.45 – 7.27 (m, 9H), 6.71 (s, 1H), 4.34 – 4.19 (m, 4H), 4.13 (dd, *J* = 5.5, 1.9 Hz, 2H), 4.09 – 3.98 (m, 1H), 3.72 (t, *J* = 6.8 Hz, 2H), 2.86 (d, *J* = 7.6 Hz, 2H), 1.64 – 1.56 (m, 2H), 1.52 – 1.39 (m, 3H), 1.39 – 1.20 (m, 4H), 1.34 (s, 9H), 0.91 – 0.79 ppm (m, 6H); ^{13}**C NMR** (151 MHz, DMSO-*d*$_6$): δ = 172.5, 171.9, 168.6, 156.0, 155.6, 144.0, 143.7, 140.7, 131.4, 128.7, 128.6, 127.7, 127.1, 125.3, 122.3, 120.1, 86.9, 81.6, 77.4, 65.5, 53.1, 52.7, 46.7, 41.9, 40.6, 39.2, 31.6, 29.2, 28.7, 28.3, 24.2, 23.1, 22.5, 21.4 ppm; **HRMS** (ESI): *m/z* calculated for $C_{43}H_{54}N_5O_7$ [*M*+H]$^+$: 752.4018, found: 752.3991.

***tert*-Butyl-((*S*)-5-((*S*)-2-acetamido-4-methylpentanamido)-6-oxo-6-((2-oxo-2-(prop-2-yn-1-ylamino)ethyl)amino)hexyl)carbamate (25d)**

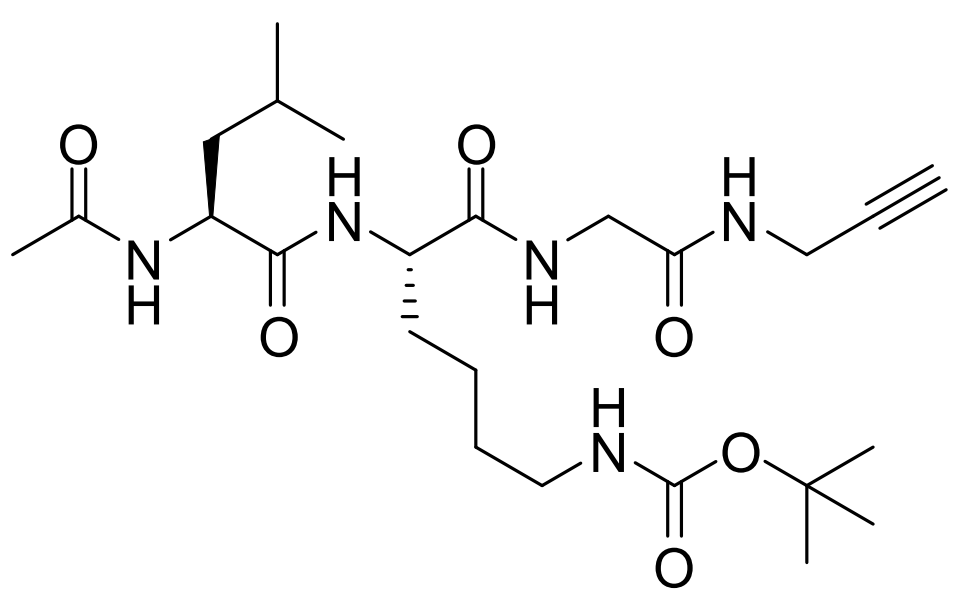

According to General Procedure F, *N*-acetyl tetrapeptide **25d** (20.3 mg, 8%) was obtained from *N*-Fmoc tetrapeptide **I38** (360 mg, 0.53 mmol) and commercially sourced acetic anhydride (53 µL, 0.56 mmol), following flash column chromatography (Biotage® Sfär Silica D Duo 5 g; 18 mL/min; 100%$_{v/v}$ cyclohexane (2 CV), linear gradient (30 CV): 0%$_{v/v}$ → 100%$_{v/v}$ acetone in cyclohexane, 100%$_{v/v}$ acetone (2 CV)), and reversed-phase HPLC (Avantor® ACE® 10 AQ 250 × 21.2 mm; 20 mL/min, linear gradient (30 min): 2%$_{v/v}$ → 98%$_{v/v}$ acetonitrile in water (each containing 0.1%$_{v/v}$ formic acid); t_R = 15.4 min).

White amorphous solid ; ^{1}**H NMR** (600 MHz, DMSO-*d*$_6$): δ = 8.19 (t, *J* = 5.6 Hz, 1H), 8.10 (t, *J* = 5.9 Hz, 1H), 7.99 (d, *J* = 8.0 Hz, 1H), 7.94 (d, *J* = 7.5 Hz, 1H), 6.74 (t, *J* = 5.7 Hz, 1H), 4.27 (td, *J* = 8.3, 6.1 Hz, 1H), 4.15 (td, *J* = 7.8, 5.1 Hz, 1H), 3.86 (td, *J* = 5.1, 2.4 Hz, 2H), 3.67 (dd, *J* = 21.0, 5.8 Hz, 2H), 3.10 (t, *J* = 2.5 Hz, 1H), 2.87 (q, *J* = 6.7 Hz, 2H), 1.84 (s, 3H), 1.65 (ddt, *J* = 14.4, 10.2, 5.5 Hz, 1H), 1.59 (dq, *J* = 13.4, 6.7 Hz, 1H), 1.52 (dtd, *J* = 13.6, 9.4, 5.0 Hz, 1H), 1.43 (ddd, *J* = 9.1, 6.1, 2.3 Hz, 2H), 1.37 (s, 9H), 1.35 – 1.31 (m, 2H), 1.26 – 1.15 (m, 2H), 0.86 ppm (dd, *J* = 24.6, 6.6 Hz, 6H); ^{13}**C NMR** (176 MHz, DMSO-*d*$_6$): δ = 172.4, 171.8, 169.4, 168.5, 155.5, 80.9, 77.3, 73.0, 52.7, 51.0, 41.8, 40.5, 39.5, 31.3, 29.2, 28.3, 27.9, 24.1, 23.1, 22.6, 22.5, 21.6 ppm; **IR** (solid): ṽ = 3289, 3078, 2957, 2934, 2870, 1632, 1532, 1460met, 1440, 1391, 1367, 1277, 1250, 1171, 1097, 1040, 1016 cm^{-1}; **HRMS** (ESI): *m/z* calculated for $C_{24}H_{42}N_5O_6$ [*M*+H]$^+$: 496.3130, found: 496.3123.

tert-Butyl-((_S_)-5-((_S_)-2-acetamido-4-methylpentanamido)-6-((2-(but-2-yn-1-ylamino)-2-oxoethyl)amino)-6-oxohexyl)carbamate (25e)

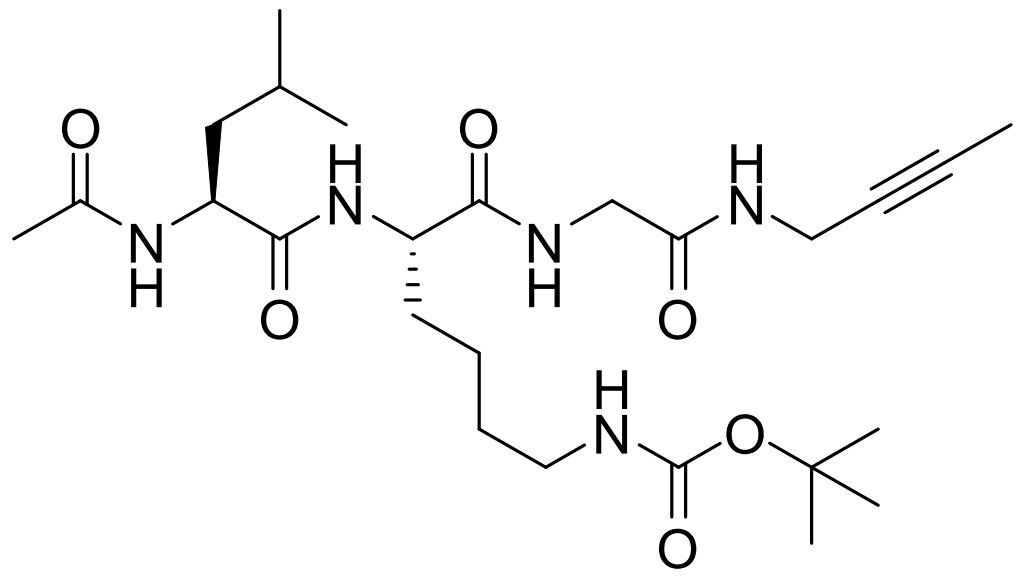

According to General Procedure F, *N*-acetyl tetrapeptide **25e** (5.8 mg, 1%) was obtained from *N*-Fmoc tetrapeptide **I39** (759 mg, 1.01 mmol) and commercially sourced acetic anhydride (0.11 mL, 1.10 mmol), following flash column chromatography (Biotage® Sfär Silica D Duo 10 g; 40 mL/min; 100%$_{v/v}$ cyclohexane (2 CV), linear gradient (8 CV): 0%$_{v/v}$ → 30%$_{v/v}$ acetone in cyclohexane, linear gradient (30 CV): 30%$_{v/v}$ → 100%$_{v/v}$ acetone in cyclohexane, 100%$_{v/v}$ acetone (10 CV)), and reversed-phase HPLC (Avantor® ACE® 10 AQ 250 × 21.2 mm; 20 mL/min, linear gradient (45 min): 2%$_{v/v}$ → 98%$_{v/v}$ acetonitrile in water (each containing 0.1%$_{v/v}$ formic acid); t_R = 20.4 min).

White amorphous solid ; ^{1}**H NMR** (400 MHz, DMSO-*d₆*): δ = 8.11 (t, *J* = 5.5 Hz, 1H), 8.07 (t, *J* = 5.9 Hz, 1H), 8.00 (d, *J* = 7.9 Hz, 1H), 7.94 (d, *J* = 7.6 Hz, 1H), 6.75 (t, *J* = 5.6 Hz, 1H), 4.32 – 4.22 (m, 1H), 4.15 (q, *J* = 7.6 Hz, 1H), 3.82 (dq, *J* = 5.3, 2.8 Hz, 2H), 3.66 (qd, *J* = 16.6, 5.6 Hz, 2H), 2.90 – 2.82 (m, 2H), 1.84 (s, 3H), 1.76 (t, *J* = 2.4 Hz, 3H), 1.70 – 1.55 (m, 2H), 1.54 – 1.46 (m, 1H), 1.45 – 1.39 (m, 2H), 1.37 (s, 9H), 1.35 – 1.28 (m, 2H), 1.28 – 1.16 (m, 2H), 0.92 – 0.81 ppm (m, 6H); ^{13}**C NMR** (151 MHz, DMSO-d_6): δ = 172.4, 171.8, 169.5, 168.4, 155.6, 78.0, 77.4, 76.3, 52.7, 51.1, 41.8, 40.5, 39.5, 31.4, 29.2, 28.3, 28.2, 24.2, 23.1, 22.6, 22.5, 21.6, 3.1 ppm; **IR** (film): ṽ = 3281 (br), 3081, 2956, 2933, 2869, 2361, 2252, 1779, 1689, 1628, 1538 cm^{-1}; **HRMS** (ESI): *m/z* calculated for $C_{25}H_{44}N_5O_6$ [*M*+H]$^+$: 510.3286, found: 510.3291.

tert-Butyl-((_S_)-5-((_S_)-2-acetamido-4-methylpentanamido)-6-((2-((1-ethynylcyclopropyl)amino)-2-oxoethyl)amino)-6-oxohexyl)carbamate (25f)

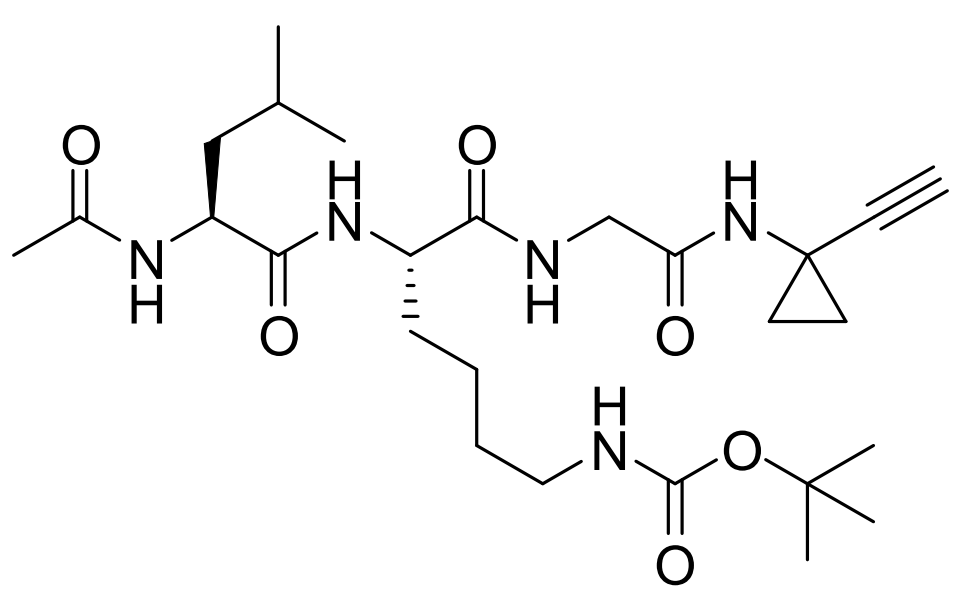

According to General Procedure F, *N*-acetyl tetrapeptide **25f** (56.6 mg, 13%) was obtained from *N*-Fmoc tetrapeptide **I40** (610 mg, 0.87 mmol) and commercially sourced acetic anhydride (87 µL, 0.92 mmol), following flash column chromatography (Biotage® Sfär Silica D Duo 10 g; 40 mL/min; 100%$_{v/v}$ cyclohexane (6 CV), linear gradient (8 CV): 0%$_{v/v}$ → 30%$_{v/v}$ acetone in cyclohexane, linear gradient (30 CV): 30%$_{v/v}$ → 100%$_{v/v}$ acetone in cyclohexane, 100%$_{v/v}$ acetone (10 CV)), and reversed-phase HPLC (Avantor® ACE® 10 AQ 250 × 21.2 mm; 20 mL/min, linear gradient (45 min): 2%$_{v/v}$ → 98%$_{v/v}$ acetonitrile in water (each containing 0.1%$_{v/v}$ formic acid); t_R = 20.1 min).

White amorphous solid ; ^{1}**H NMR** (400 MHz, DMSO-d_6): δ = 8.30 (s, 1H), 8.05 (t, *J* = 5.8 Hz, 1H), 7.98 (t, *J* = 8.1 Hz, 2H), 6.75 (t, *J* = 5.7 Hz, 1H), 4.27 (qd, *J* = 7.3, 4.3 Hz, 1H), 4.11 (td, *J* = 8.0, 5.2 Hz, 1H), 3.69 – 3.50 (m, 2H), 2.94 (s, 1H), 2.87 (q, *J* = 6.6 Hz, 2H), 1.84 (s, 3H), 1.68 – 1.55 (m, 2H), 1.54 – 1.46 (m, 1H), 1.45 – 1.40 (m, 2H), 1.36 (s, 9H), 1.35 – 1.29 (m, 2H), 1.28 – 1.14 (m, 2H), 1.07 (m, 2H), 1.08 – 0.83 (m, 2H), 0.86 ppm (dd, *J* = 16.9, 6.5 Hz, 6H); ^{13}**C NMR** (151 MHz, DMSO-d_6): δ = 172.5, 171.8, 169.5, 169.0, 155.6, 86.2, 77.4, 68.6, 52.8, 51.1, 41.9, 40.5, 39.5, 31.3, 29.2, 28.3, 24.2, 23.1, 22.7, 22.5, 21.8, 21.6, 16.8 ppm (2C); **IR** (film): ṽ = 3307 (br), 2958, 2118, 1645, 1535, 1452, 1367, 1287, 1251, 1171 cm^{-1}; **HRMS** (ESI): *m/z* calculated for $C_{26}H_{44}N_5O_6$ [*M*+H]$^+$: 522.3286, found: 522.3288.

***tert*-Butyl-((*S*)-5-((*S*)-2-acetamido-4-methylpentanamido)-6-((2-((1-(1-chlorovinyl)cyclopropyl)amino)-2-oxoethyl)amino)-6-oxohexyl)carbamate (25c)**

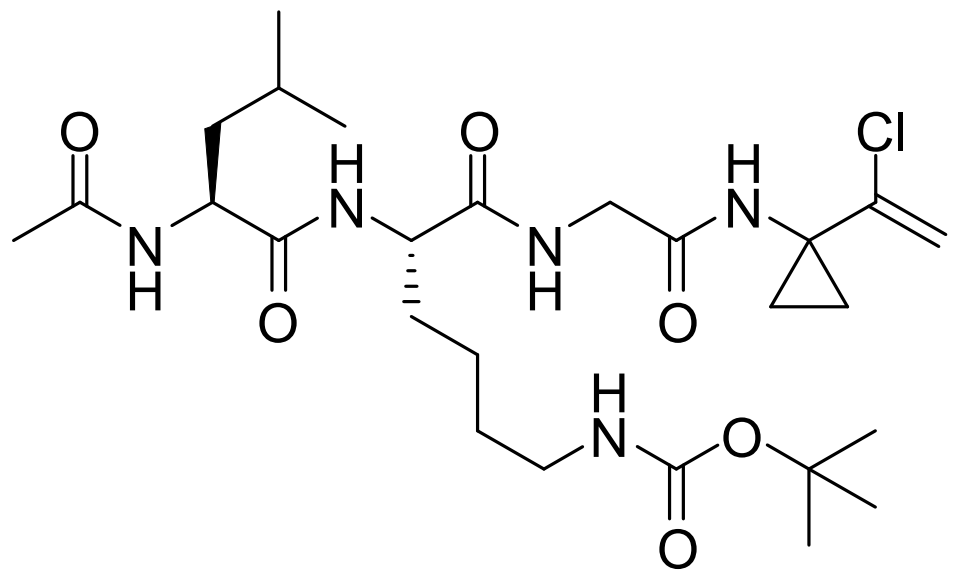

According to General Procedure F, *N*-acetyl tetrapeptide **25c** (10.03 mg, ~ 5%) was obtained from *N*-Fmoc tetrapeptide **I41** (244 mg, 1:1 mixture of **I41** and **I40** as per ^{1}H NMR analysis) and commercially sourced acetic anhydride (34.5 µL, 0.37 mmol), following flash column chromatography (Biotage® Sfär Silica D Duo 10 g; 40 mL/min; 100%$_{v/v}$ cyclohexane (2 CV), linear gradient (8 CV): 0%$_{v/v}$ → 30%$_{v/v}$ acetone in cyclohexane, linear gradient (30 CV): 30%$_{v/v}$ → 100%$_{v/v}$ acetone in cyclohexane, 100%$_{v/v}$ acetone (10 CV)), and reversed-phase HPLC (Avantor® ACE® 10 AQ 250 × 21.2 mm; 20 mL/min, linear gradient (30 min): 2%$_{v/v}$ → 98%$_{v/v}$ acetonitrile in water (each containing 0.1%$_{v/v}$ formic acid); t_R = 17.4 min).

White amorphous solid ; **^{1}H NMR** (600 MHz, DMSO-*d$_6$*): δ = 8.32 (s, 1H), 8.12 (t, *J* = 5.8 Hz, 1H), 7.99 (t, *J* = 8.3 Hz, 2H), 6.74 (t, *J* = 5.7 Hz, 1H), 5.43 (d, *J* = 1.8 Hz, 1H), 5.23 (d, *J* = 1.8 Hz, 1H), 4.30 – 4.23 (m, 1H), 4.11 (q, *J* = 6.3 Hz, 1H), 3.69 – 3.55 (m, 2H), 2.87 (q, *J* = 6.7 Hz, 2H), 1.84 (s, 3H), 1.68 – 1.61 (m, 1H), 1.60 – 1.55 (m, 1H), 1.54 – 1.47 (m, 1H), 1.42 (m, 2H), 1.37 (s, 9H), 1.36 – 1.31 (m, 2H), 1.29 – 1.16 (m, 4H), 0.93 (m, 2H), 0.86 ppm (dd, *J* = 24.8, 6.6 Hz, 6H); **^{13}C NMR** (151 MHz, DMSO-*d$_6$*): δ = 172.5, 172.0, 169.4, 169.1, 155.5, 141.2, 112.2, 77.3, 52.8, 51.0, 42.2, 40.5, 39.5, 35.9, 31.2, 29.2, 28.3, 24.1, 23.0, 22.7, 22.4, 21.6, 14.4, 14.2 ppm; **IR** (film): ṽ = 3281, 3067, 2954, 2936, 2868, 2247, 1685, 1630, 1532 cm^{-1}; **HRMS** (ESI): *m/z* calculated for $C_{26}H_{45}ClN_5O_6$ [*M*+H]$^+$: 558.3053, found: 558.3064.

***tert*-Butyl-((*S*)-5-((*S*)-2-acetamido-4-methylpentanamido)-6-oxo-6-((2-oxo-2-((3-phenylprop-2-yn-1-yl)amino)ethyl)amino)hexyl)carbamate (25g)**

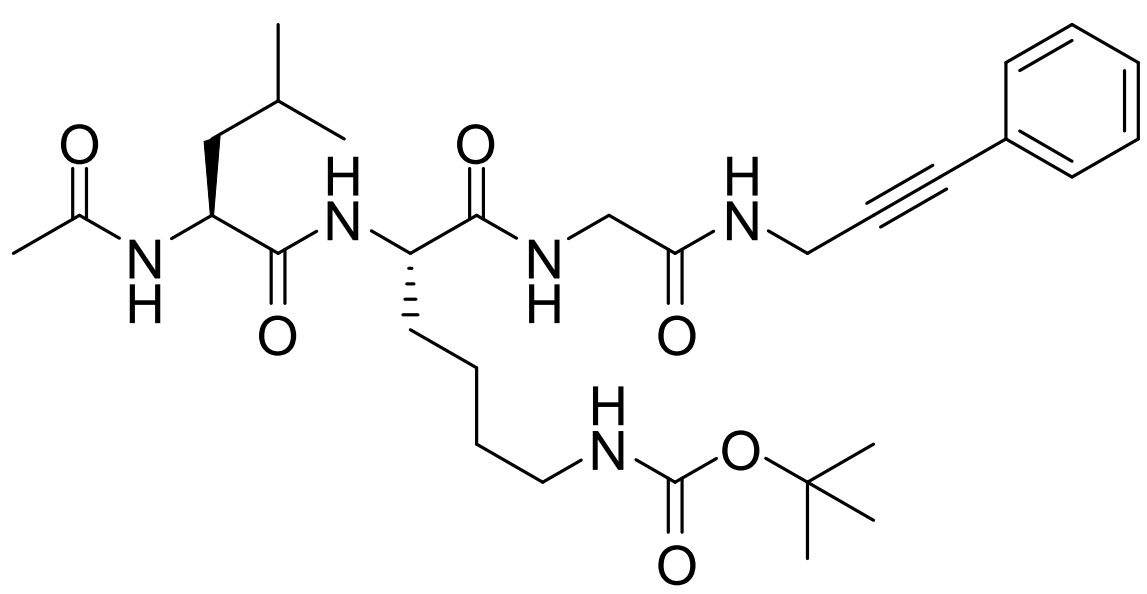

According to General Procedure F, *N*-acetyl tetrapeptide **25g** (93.0 mg, 21%) was obtained from *N*-Fmoc tetrapeptide **I42** (590 mg, 0.78 mmol) and commercially sourced acetic anhydride (80 µL, 0.85 mmol), following flash column chromatography (Biotage® Sfär Silica D Duo 10 g; 40 mL/min; 100%$_{v/v}$ cyclohexane (2 CV), linear gradient (8 CV): 0%$_{v/v}$ → 30%$_{v/v}$ acetone in cyclohexane, linear gradient (30 CV): 30%$_{v/v}$ → 100%$_{v/v}$ acetone in cyclohexane, 100%$_{v/v}$ acetone (10 CV)), and reversed-phase HPLC (Avantor® ACE® 10 AQ 250 × 21.2 mm; 20 mL/min, linear gradient (45 min): 2%$_{v/v}$ → 98%$_{v/v}$ acetonitrile in water (each containing 0.1%$_{v/v}$ formic acid); t_R = 24.4 min).

White amorphous solid ; **^{1}H NMR** (400 MHz, DMSO-*d$_6$*): δ = 8.31 (t, *J* = 5.5 Hz, 1H), 8.13 (t, *J* = 5.9 Hz, 1H), 8.00 (d, *J* = 7.9 Hz, 1H), 7.96 (d, *J* = 7.6 Hz, 1H), 7.45 – 7.31 (m, 5H), 6.75 (t, *J* = 5.6 Hz, 1H), 4.28 (td, *J* = 8.3, 6.3 Hz, 1H), 4.18 (dd, *J* = 8.0, 5.4 Hz, 1H), 4.14 (dd, *J* = 5.5, 3.2 Hz, 2H), 3.71 (dd, *J* = 11.7, 5.8 Hz, 2H), 2.87 (q, *J* = 6.6 Hz, 2H), 1.84 (s, 3H), 1.71 – 1.63 (m, 1H), 1.62 – 1.56 (m, 1H), 1.55 – 1.47 (m, 1H), 1.46 – 1.39 (m, 2H), 1.36 (s, 9H), 1.35 – 1.29 (m, 2H), 1.28 – 1.16 (m, 2H), 0.84 ppm (dd, *J* = 14.1, 6.6 Hz, 6H); **^{13}C NMR** (101 MHz, DMSO-*d$_6$*): δ = 172.4, 171.9, 169.4, 168.6, 155.6, 131.4, 128.7, 128.6, 122.2, 86.9, 81.6, 77.3, 52.7, 51.1, 41.9, 40.5, 39.5, 31.4, 29.2, 28.6, 28.3, 24.2, 23.0, 22.6, 22.5, 21.6 ppm; **IR** (film): ṽ = 3287 (br), 3080, 2971, 2883, 2117, 1683, 1634, 1451, 1368, 1287, 1252, 1172 cm^{-1}; **HRMS** (ESI): *m/z* calculated for $C_{30}H_{46}N_5O_6$ [*M*+H]$^+$: 572.3443, found: 572.3440.

tert-Butyl-((S)-5-((S)-4-methyl-2-(2,2,2-trifluoroacetamido)pentanamido)-6-oxo-6-((2-oxo-2-(prop-2-yn-1-ylamino)ethyl)amino)hexyl)carbamate (25a)

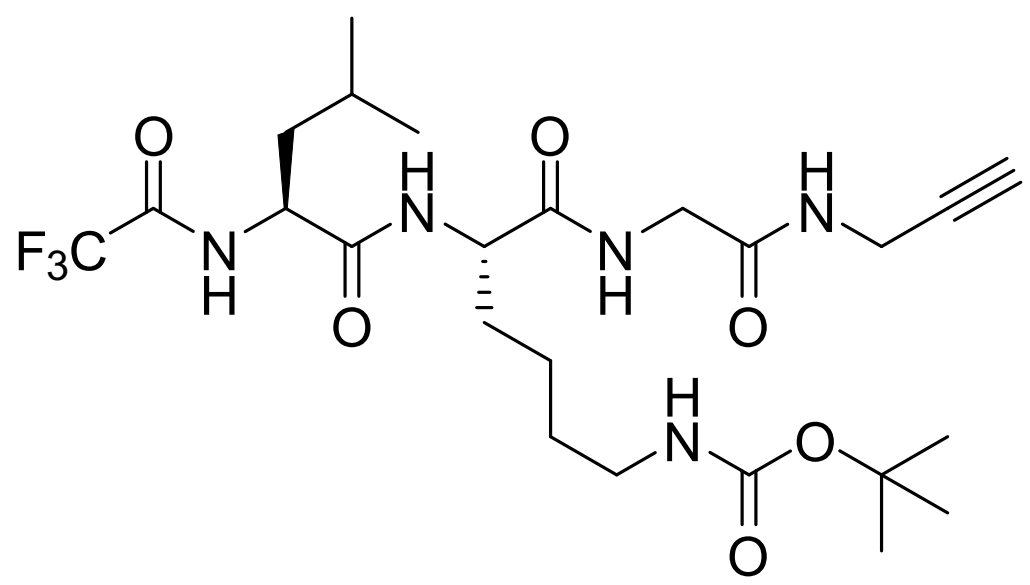

According to General Procedure F, *N*-acetyl tetrapeptide **25a** (28.3 mg, 11%) was obtained from *N*-Fmoc tetrapeptide **I38** (300 mg, 0.44 mmol) and commercially sourced trifluoroacetic anhydride (65 µL, 0.47 mmol), following flash column chromatography (Biotage® Sfär Silica D Duo 25 g; 80 mL/min; 100%$_{v/v}$ cyclohexane (2 CV), linear gradient (8 CV): 0%$_{v/v}$ → 30%$_{v/v}$ acetone in cyclohexane, linear gradient (30 CV): 30%$_{v/v}$ → 100%$_{v/v}$ acetone in cyclohexane, 100%$_{v/v}$ acetone (2 CV)), and reversed-phase HPLC (Avantor® ACE® 10 AQ 250 × 21.2 mm; 20 mL/min, linear gradient (45 min): 2%$_{v/v}$ → 98%$_{v/v}$ acetonitrile in water (each containing 0.1%$_{v/v}$ formic acid); t_R = 22.9 min).

White amorphous solid ; **^{1}H NMR** (400 MHz, DMSO-d_6): δ = 9.43 (d, *J* = 8.2 Hz, 1H), 8.18 – 8.08 (m, 2H), 8.07 (t, *J* = 5.8 Hz, 1H), 6.63 (t, *J* = 5.7 Hz, 1H), 4.42 (ddd, *J* = 10.6, 8.1, 4.2 Hz, 1H), 4.20 (td, *J* = 8.0, 5.6 Hz, 1H), 3.87 (dt, *J* = 5.3, 2.5 Hz, 2H), 3.68 (d, *J* = 5.8 Hz, 2H), 3.04 (t, *J* = 2.5 Hz, 1H), 2.88 (q, *J* = 6.6 Hz, 2H), 1.71 – 1.61 (m, 2H), 1.60 – 1.47 (m, 3H), 1.37 (s, 9H), 1.36 – 1.32 (m, 2H), 1.31 – 1.17 (m, 2H), 0.94 – 0.79 ppm (m, 6H); **^{13}C NMR** (101 MHz, DMSO-d_6): δ = 171.8, 170.6, 168.5, 156.3 (q, *J* = 36.3 Hz), 155.6, 115.9 (t, *J* = 288.0 Hz), 81.0, 77.4, 73.1, 52.9, 51.7, 41.8, 39.2 (2C), 31.5, 29.2, 28.3, 27.9, 24.3, 23.0, 22.6, 21.1 ppm; **^{19}F NMR** (376 MHz, DMSO-d_6): δ = −73.9 ppm (s, 3F); **IR** (film): ṽ = 3288 (br), 2347, 2253, 1673 (br), 1542, 1368, 1180, 1167 cm^{-1}; **HRMS** (ESI): *m/z* calculated for $C_{24}H_{39}F_3N_5O_6$ [*M*+H]$^+$: 550.2847, found: 550.2847.

(S)-5-((S)-2-Acetamido-4-methylpentanamido)-6-oxo-6-((2-oxo-2-(prop-2-yn-1-ylamino)ethyl)amino)hexan-1-aminium chloride (25)

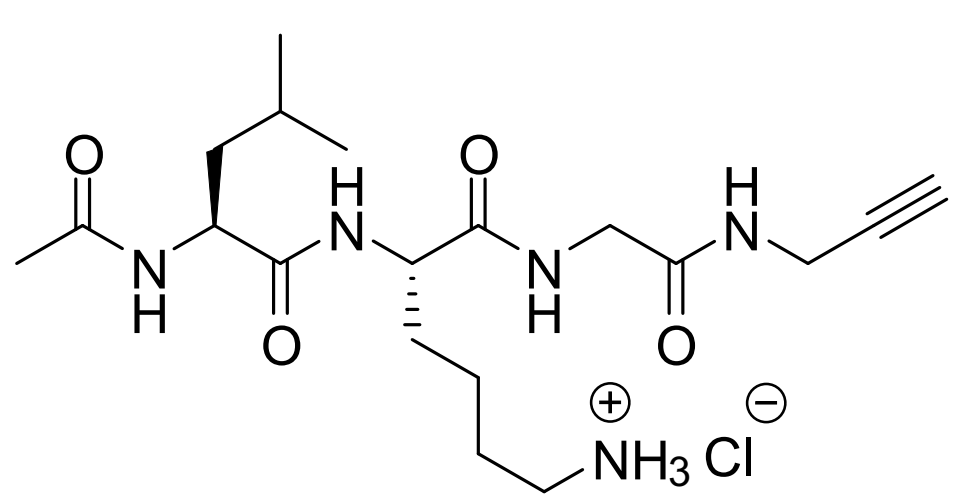

According to General Procedure G, tetrapeptide **25** (6.3 mg, 60%) was obtained from *N*-Boc tetrapeptide **25d** (12.2 mg, 0.025 mmol).

White amorphous solid ; **^{1}H NMR** (400 MHz, DMSO-d_6): δ = 8.26 (t, *J* = 5.5 Hz, 1H), 8.09 (t, *J* = 5.8 Hz, 1H), 8.01 (t, *J* = 7.3 Hz, 2H), 7.67 (s, 2H), 4.30 – 4.17 (m, 2H), 3.86 (ddd, *J* = 5.7, 2.6, 1.4 Hz, 2H), 3.69 (dd, *J* = 5.6, 1.5 Hz, 2H), 3.11 (t, *J* = 2.5 Hz, 1H), 2.75 (d, *J* = 8.4 Hz, 2H), 1.84 (s, 3H), 1.68 (dt, *J* = 13.8, 6.3 Hz, 1H), 1.59 (dt, *J* = 13.5, 6.8 Hz, 1H), 1.55 – 1.46 (m, 3H), 1.43 (dd, *J* = 8.3, 6.3 Hz, 2H), 1.37 – 1.26 (m, 2H), 0.86 ppm (dd, *J* = 16.4, 6.6 Hz, 6H); **^{13}C NMR** (151 MHz, DMSO-d_6): δ = 172.5, 171.7, 169.5, 168.5, 80.9, 73.1, 52.3, 51.1, 41.7, 40.4, 38.7, 31.0, 27.9, 26.5, 24.2, 23.1, 22.5, 22.1, 21.5 ppm; **IR** (film): ṽ = 3010, 2898, 2855, 2265, 1734, 1636, 1541, 1437, 1291 cm^{-1}; **HRMS** (ESI): *m/z* calculated for $C_{19}H_{34}N_5O_4$ [*M*−*Cl*$^-$]$^+$: 396.2605, found: 396.2601.

(S)-5-((S)-2-Acetamido-4-methylpentanamido)-6-((2-(but-2-yn-1-ylamino)-2-oxoethyl)amino)-6-oxohexan-1-aminium chloride (25j)

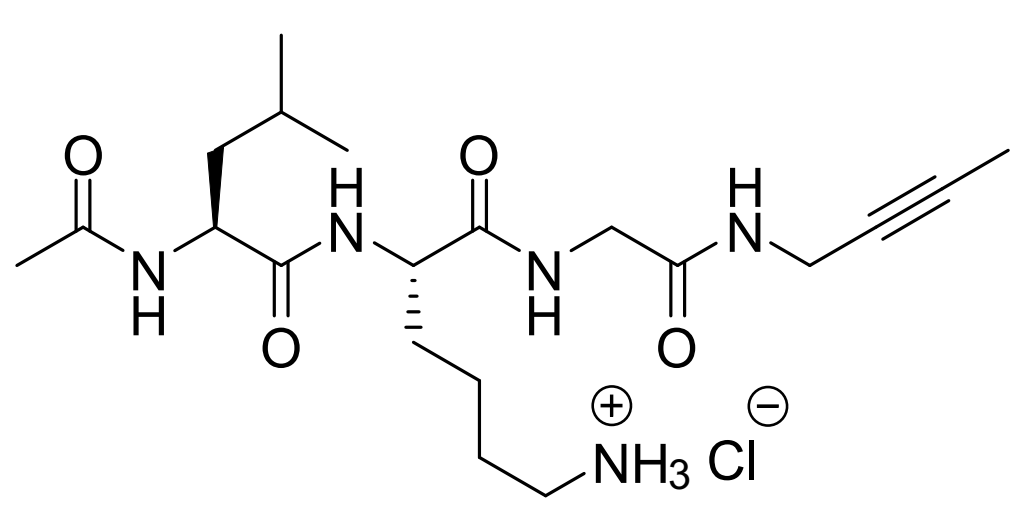

According to General Procedure G, tetrapeptide **25j** (2.0 mg, 50%) was obtained from *N*-Boc tetrapeptide **25e** (3.9 mg, 0.008 mmol).

White amorphous solid ; **^{1}H NMR** (400 MHz, DMSO-d_6): δ = 8.35 (s, 2H), 8.18 (t, *J* = 5.5 Hz, 1H), 8.10 (t, *J* = 5.9 Hz, 1H), 8.04 (dd, *J* = 10.4, 7.8 Hz, 2H), 4.30 – 4.23 (m, 1H), 4.22 – 4.16 (m, 1H), 3.82 (p, *J* = 2.5 Hz, 2H), 3.73 – 3.61 (m, 2H), 2.71 (t, *J* = 7.4 Hz,

2H), 1.84 (s, 3H), 1.76 (t, *J* = 2.5 Hz, 3H), 1.72 – 1.64 (m, 1H), 1.63 – 1.57 (m, 1H), 1.56 – 1.53 (m, 1H), 1.49 (m, 2H), 1.43 (t, *J* = 7.3 Hz, 2H), 1.34 – 1.21 (m, 2H), 0.86 ppm (dd, *J* = 16.5, 6.6 Hz, 6H); **^{13}C NMR** (151 MHz, DMSO-*d$_6$*): δ = 172.4, 171.7, 169.5, 168.3, 78.0, 76.2, 52.3, 51.2, 41.7, 40.5, 38.8, 31.1, 28.2, 27.0, 24.2, 23.1, 22.5, 22.1, 21.5, 3.0 ppm; **IR** (film): ṽ = 3296 (br), 2957, 2925, 2856, 2361, 2270, 1652, 1547, 1469, 1237 cm^{-1}; **HRMS** (ESI): *m/z* calculated for $C_{20}H_{36}N_5O_4$ [*M*−*Cl*$^-$]$^+$: 410.2762, found: 410.2760.

(*S*)-5-((*S*)-2-Acetamido-4-methylpentanamido)-6-((2-((1-ethynylcyclopropyl)amino)-2-oxoethyl)amino)-6-oxohexan-1-aminium chloride (25h)

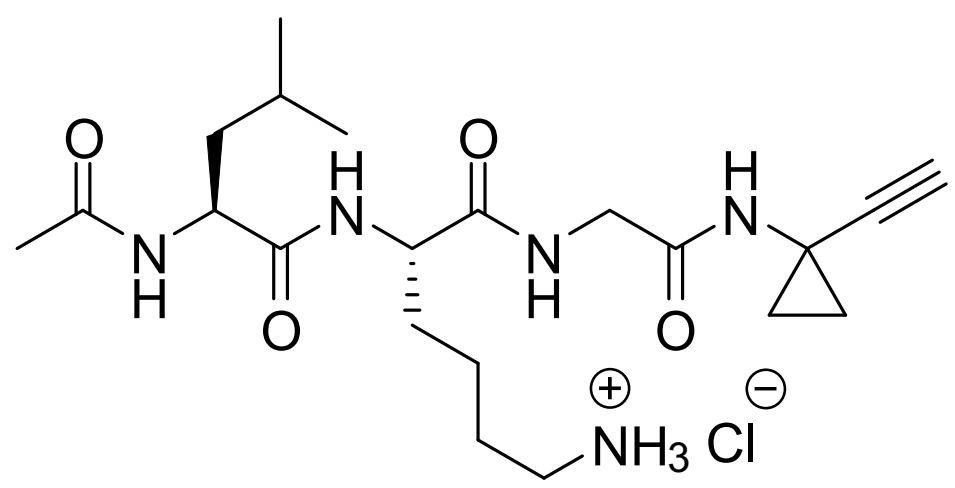

According to General Procedure G, tetrapeptide **25h** (7.6 mg, 77%) was obtained from *N*-Boc tetrapeptide **25f** (11.5 mg, 0.022 mmol).

White amorphous solid ; **^{1}H NMR** (400 MHz, DMSO-*d$_6$*): δ = 8.39 (s, 1H), 8.10 – 8.01 (m, 3H), 7.80 (s, 2H), 4.31 – 4.21 (m, 1H), 4.18 (td, *J* = 8.3, 5.2 Hz, 1H), 3.69 – 3.53 (m, 2H), 2.95 (s, 1H), 2.74 (s, 2H), 1.84 (s, 3H), 1.74 – 1.64 (m, 1H), 1.63 – 1.55 (m, 2H) 1.53 (m, 2H), 1.44 (t, *J* = 7.3 Hz, 2H), 1.37 – 1.25 (m, 2H), 1.10 – 1.05 (m, 2H), 0.96 – 0.91 (m, 2H), 0.87 ppm (dd, *J* = 16.7, 6.5 Hz, 6H); **^{13}C NMR** (151 MHz, DMSO-*d$_6$*): δ = 172.5, 171.6, 169.5, 168.9, 86.2, 68.6, 52.4, 51.1, 41.8, 40.5, 38.6, 31.0, 26.5, 24.1, 23.1, 22.5, 22.1, 21.8, 21.5, 16.7 ppm (2C); **IR** (film): ṽ = 2928, 2264, 1733, 1644, 1548, 1439, 1292, 1109 cm^{-1}; **HRMS** (ESI): *m/z* calculated for $C_{21}H_{36}N_5O_4$ [*M*−*Cl*$^-$]$^+$: 422.2762, found: 422.2742.

(*S*)-5-((*S*)-2-Acetamido-4-methylpentanamido)-6-oxo-6-((2-oxo-2-((3-phenylprop-2-yn-1-yl)amino)ethyl)amino)hexan-1-aminium chloride (25i)

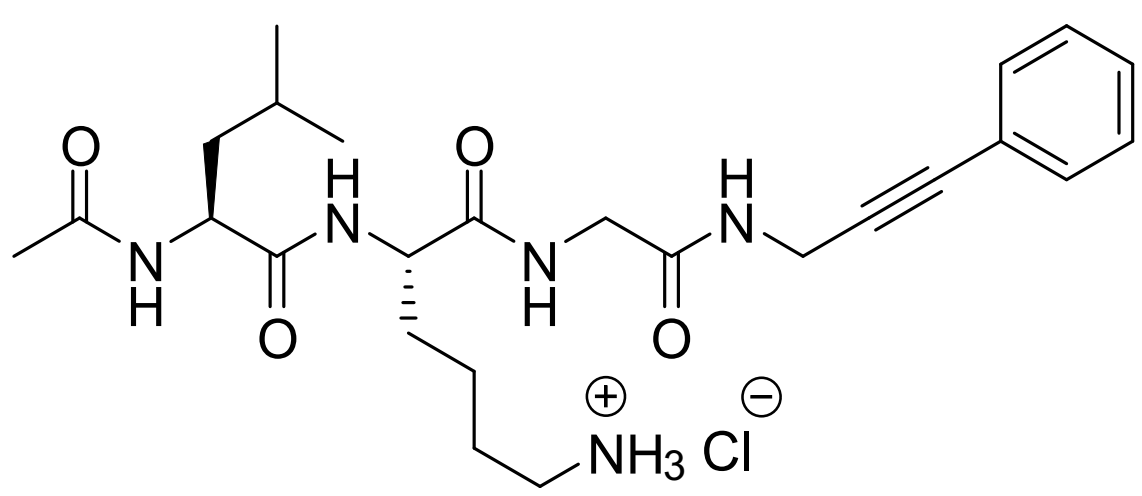

According to General Procedure G, tetrapeptide **25i** (4.1 mg, 89%) was obtained from *N*-Boc-protected tetrapeptide **25g** (5.2 mg, 0.009 mmol).

White amorphous solid ; **^{1}H NMR** (400 MHz, DMSO-*d$_6$*): δ = 8.37 (t, *J* = 5.5 Hz, 1H), 8.24 (s, 1H), 8.13 (t, *J* = 5.8 Hz, 1H), 8.03 (d, *J* = 7.8 Hz, 2H), 7.45 – 7.33 (m, 5H), 4.25 (dt, *J* = 14.9, 7.2 Hz, 2H), 4.17 – 4.11 (m, 2H), 3.74 – 3.70 (m, 2H), 2.74 (t, *J* = 7.5 Hz, 2H), 1.84 (s, 3H), 1.74 – 1.64 (m, 1H), 1.64 – 1.54 (m, 2H), 1.54 – 1.46 (m, 2H), 1.46 – 1.40 (m, 2H), 1.34 – 1.28 (m, 2H), 0.85 ppm (dd, *J* = 14.7, 6.6 Hz, 6H); **^{13}C NMR** (151 MHz, DMSO-*d$_6$*): δ = 172.5, 171.7, 169.5, 168.5, 131.3, 128.7, 128.6, 122.2, 86.8, 81.6, 52.3, 51.2, 41.8, 40.4, 38.7, 31.1, 28.6, 26.6, 24.2, 23.1, 22.5, 22.1, 21.5 ppm; **IR** (film): ṽ = 3270 (br), 2930, 2864, 2335, 2255, 1638, 1549 cm^{-1}; **HRMS** (ESI): *m/z* calculated for $C_{25}H_{38}N_5O_4$ [*M*−*Cl*$^-$]$^+$: 472.2918, found: 472.2916.

(*S*)-5-((*S*)-4-Methyl-2-(2,2,2-trifluoroacetamido)pentanamido)-6-oxo-6-((2-oxo-2-(prop-2-yn-1-ylamino)ethyl)amino)hexan-1-aminium chloride (25k)

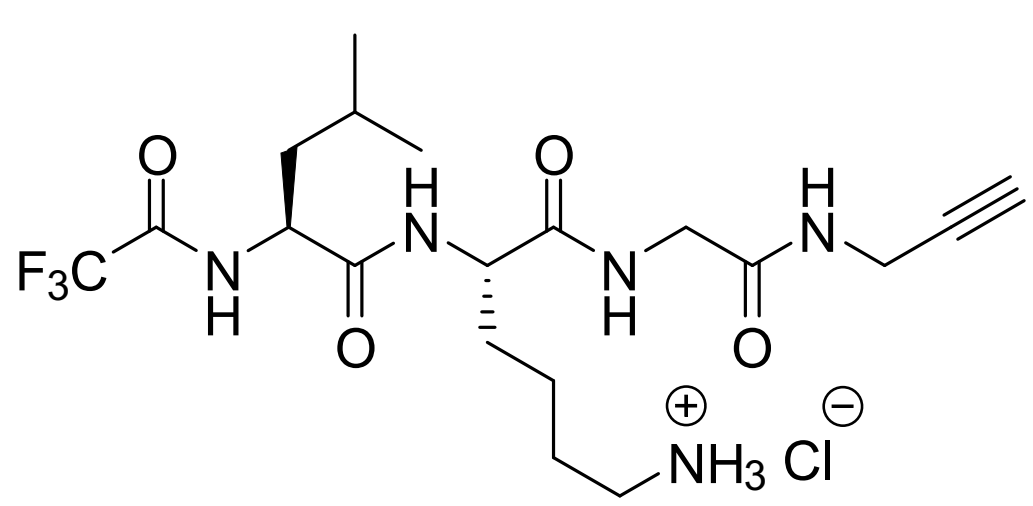

According to General Procedure G, tetrapeptide **25k** (5.5 mg, 73%) was obtained from *N*-Boc tetrapeptide **25a** (8.4 mg, 0.015 mmol).

White amorphous solid ; **^{1}H NMR** (400 MHz, DMSO-*d$_6$*): δ = 9.54 (d, *J* = 7.9 Hz, 1H), 8.29 (dd, *J* = 9.5, 6.5 Hz, 2H), 8.16 (t, *J* = 5.8 Hz, 1H), 7.64 (s, 3H), 4.46 – 4.35 (m, 1H), 4.25 (q, *J* = 8.0 Hz, 1H), 3.90 – 3.84 (m, 2H), 3.73 – 3.62 (m, 2H), 3.12 (t, *J* = 2.5 Hz, 1H), 2.74 (d, *J* = 7.1 Hz, 2H), 1.73 – 1.60 (m, 2H), 1.60 – 1.46 (m, 5H), 1.35 – 1.27 (m, 2H), 0.87 ppm (dd, *J* = 14.0, 6.3 Hz, 6H); **^{13}C NMR** (151 MHz, DMSO-*d$_6$*): δ = 171.6, 170.7, 168.5, 156.3 (d, *J* = 36.9 Hz), 115.9 (d, *J* = 288.1 Hz), 81.0, 73.1, 52.5, 51.7, 41.7, 39.2, 38.7, 31.2, 27.9, 26.6, 24.3, 23.0, 22.1, 21.1 ppm; **^{19}F NMR**

(376 MHz, DMSO-d_6): δ = -73.8 ppm (m, 3F); **IR** (film): ṽ = 3289 (br), 2961 (br), 2253, 1719, 1648, 1541, 1389, 1261, 1215, 1185, 1073 cm^{-1}; **HRMS** (ESI): *m/z* calculated for $C_{19}H_{31}F_3N_5O_4$ $[M-Cl^-]^+$: 450.2323, found: 450.2325.

(*S*)-2-((*S*)-4-Methyl-2-(2,2,2-trifluoroacetamido)pentanamido)-*N*-(2-oxo-2-(prop-2-yn-1-ylamino)ethyl)-6-(2,2,2-trifluoroacetamido)hexanamide (25b)

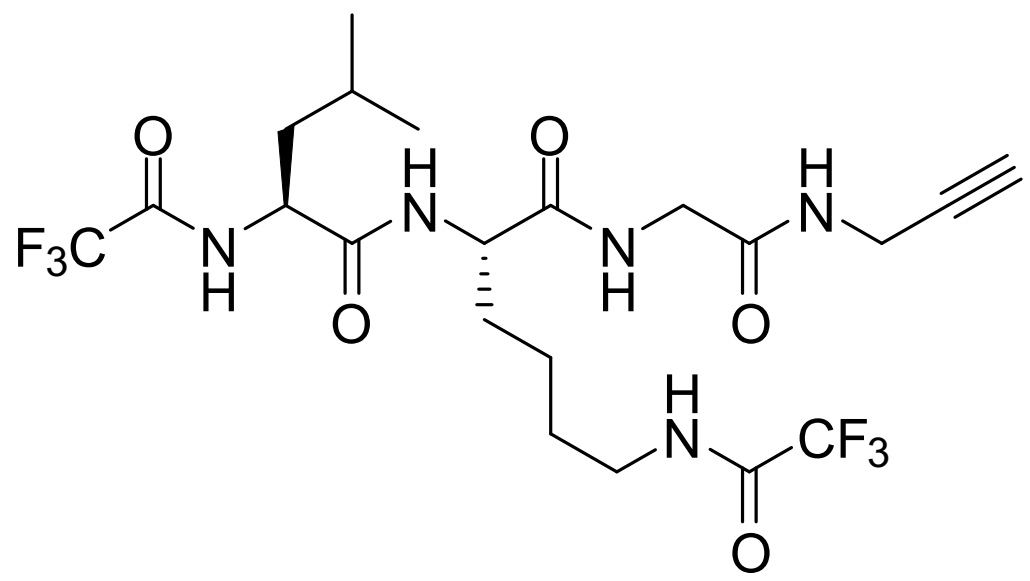

N-Boc tetrapeptide **25k** (60 mg, 0.110 mmol) was deprotected according to General Procedure G. Commercially sourced 4-(*N,N*-dimethylamino)pyridine (1.05 equiv.), anhydrous tetrahydrofuran (0.09 M) and trifluoroacetic anhydride (1.05 equiv.) were added, and the reaction stirred under reflux (1 h). After cooling to ambient temperature, the mixture was concentrated under reduced pressure, and the crude residue was redissolved in ethyl acetate. The mixture was washed with aqueous HCl (1 M), saturated aqueous $NaHCO_3$ and brine, then dried over anhydrous Na_2SO_4, filtered and concentrated under reduced pressure. The residue was purified via flash column chromatography (Biotage® Sfär Silica D Duo 5 g; 18 mL/min; 100%$_{v/v}$ cyclohexane (3 CV), linear gradient (15 CV): 0%$_{v/v}$ → 100%$_{v/v}$ acetone in cyclohexane, 100%$_{v/v}$ acetone (3 CV)) and reversed-phase HPLC (Avantor® ACE® 10 AQ 250 × 21.2 mm; 20 mL/min, linear gradient (45 min): 2%$_{v/v}$ → 98%$_{v/v}$ acetonitrile in water (each containing 0.1%$_{v/v}$ formic acid); t_R = 21.0 min), to give the desired peptide **25b** (0.43 mg, 1%).

White amorphous solid; **^{1}H NMR** (600 MHz, DMSO-d_6): δ = 9.53 (d, *J* = 7.9 Hz, 1H), 9.38 (s, 1H), 8.24 (t, *J* = 5.5 Hz, 2H), 8.18 (t, *J* = 5.8 Hz, 1H), 4.45 – 4.38 (m, 1H), 4.22 (td, *J* = 8.0, 5.3 Hz, 1H), 3.86 (ddd, *J* = 6.1, 3.8, 2.5 Hz, 2H), 3.68 (d, *J* = 5.9 Hz, 2H), 3.15 (q, *J* = 6.6 Hz, 2H), 3.14 – 3.08 (m, 1H), 1.72 – 1.57 (m, 2H), 1.59 – 1.43 (m, 5H), 1.28 (ddd, *J* = 24.2, 16.3, 8.9 Hz, 2H), 0.87 ppm (dd, *J* = 20.2, 6.4 Hz, 6H); **^{13}C NMR** (151 MHz, DMSO-d_6): δ = 171.7, 170.6, 168.4, 156.2 (d, *J* = 36.4 Hz), 156.1 (d, *J* = 35.8 Hz), 116.0 (d, *J* = 288.3 Hz), 115.9 (d, *J* = 287.7 Hz), 81.0, 73.0, 52.7, 51.7, 41.7, 39.5, 39.0, 31.4, 27.9, 27.8(6), 24.3, 23.0, 22.5, 21.1 ppm; **^{19}F NMR** (565 MHz, DMSO-d_6): δ = −73.9 (s, 3F), −74.4 ppm (s, 3F); **IR** (film): ṽ = 3293 (br), 3094, 2949, 2126, 1704, 1640, 1548, 1209, 1186, 1158 cm^{-1}; **HRMS** (ESI): *m/z* calculated for $C_{21}H_{30}F_6N_5O_5$ $[M+H]^+$: 546.2146, found: 546.2141.

***N*-((*S*)-5-((*S*)-4-Methyl-2-(2,2,2-trifluoroacetamido)pentanamido)-6-oxo-6-((2-oxo-2-(prop-2-yn-1-ylamino)ethyl)amino)hexyl)-1-(5-((3a*S*,4*S*,6a*R*)-2-oxohexahydro-1*H*-thieno[3,4-*d*]imidazol-4-yl)pentanamido)-3,6,9,12-tetraoxapentadecan-15-amide (31, bio-a-25a)**

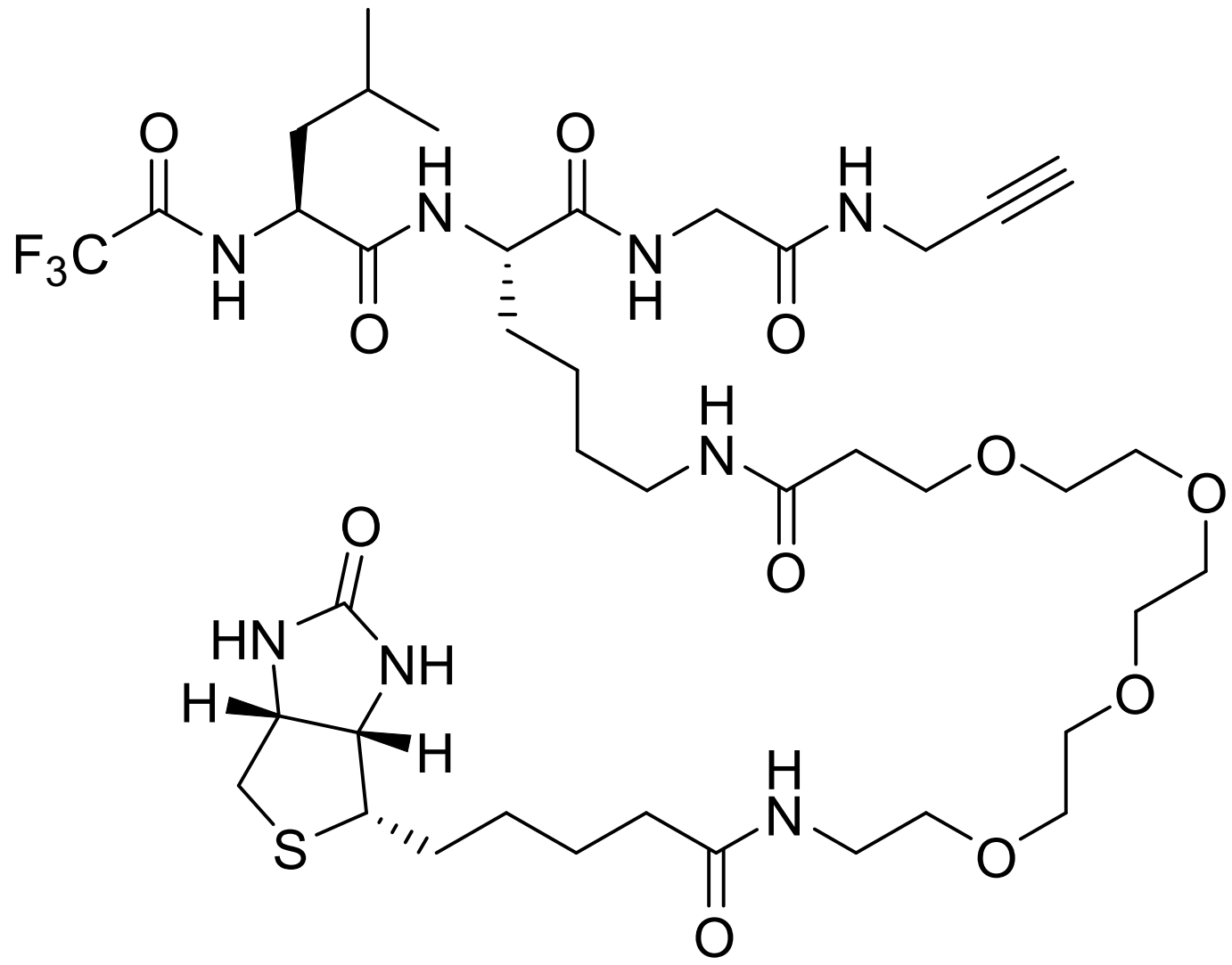

N-Boc tetrapeptide **24** (102.6 mg, 0.187 mmol) was deprotected according to General Procedure G, and was directly used in the following step: According to General Procedure **I**, *N*-PEG-biotinylated tetrapeptide **31** (74.7 mg, 43%) was obtained from commercial Biotin-PEG4-NHS ester (1.2 equiv.), following high-performance liquid chromatography (Avantor® ACE® 10 AQ 250 × 21.2 mm; 20 mL/min), isocratic hold (9.4 min) 2%$_{v/v}$; linear gradient (77.6 min): 2%$_{v/v}$ → 98%$_{v/v}$ acetonitrile in water (each containing 0.1%$_{v/v}$ formic acid); t_R = 36.0 min.

White solid; ^{1}H NMR (400 MHz, DMSO-d_6): δ = 9.52 (d, *J* = 8.1 Hz, 1H), 8.26 – 8.19 (m, 2H), 8.16 (t, *J* = 5.8 Hz, 1H), 7.82 (t, *J* = 5.7 Hz, 1H), 7.78 (t, *J* = 5.6 Hz, 1H), 6.41 (t, *J* = 1.8 Hz, 1H), 6.35 (s, 1H), 4.42 (ddd, *J* = 10.9, 8.0, 4.0 Hz, 1H), 4.30 (dd, *J* = 7.8, 5.0 Hz, 1H), 4.19 (td, *J* = 8.1, 5.5 Hz, 1H), 4.12 (ddd, *J* = 7.8, 4.4, 1.9 Hz, 1H), 3.87 (dt, *J* = 5.2, 2.4 Hz, 2H), 3.68 (d, *J* = 5.8 Hz, 2H), 3.58 (t, *J* = 6.5 Hz, 2H), 3.50 (d, *J* = 1.3 Hz, 8H), 3.47 (q, *J* = 2.0 Hz, 4H), 3.39 (t, *J* = 5.9 Hz, 2H), 3.18 (q, *J* = 5.8 Hz, 2H), 3.12 – 3.06 (m, 2H), 3.00 (q, *J* = 6.6 Hz, 2H), 2.82 (dd, *J* = 12.4, 5.1 Hz, 1H), 2.57 (d, *J* = 12.4 Hz, 1H), 2.29 (t, *J* = 6.5 Hz, 2H), 2.06 (t, *J* = 7.4 Hz, 2H), 1.70 – 1.41 (m, 9H), 1.41 – 1.19 (m, 6H), 0.87 ppm (dd, *J* = 13.5, 6.1 Hz, 6H); ^{13}C NMR (151 MHz, DMSO-d_6): δ = 172.1, 171.8, 170.6, 169.8, 168.5, 162.7, 156.3 (q, *J* = 36.4 Hz), 115.9 (q, *J* = 288.3 Hz), 81.0, 73.1, 69.7(8), 69.7(7), 69.7, 69.6(7), 69.6, 69.5, 69.2, 66.9, 61.0, 59.2, 55.4, 52.8, 51.7, 41.7, 39.8, 39.2, 38.4, 38.3, 36.1, 35.1, 31.4, 28.8, 28.2, 28.0, 27.9, 25.3, 24.3, 23.0, 22.6, 21.1 ppm; ^{19}F NMR (376 MHz, DMSO-d_6): δ = −74.4 ppm (s, 3F); IR (film): ṽ = 3268 (br), 3076, 2929, 2871, 2360, 2341, 1650, 1547, 1350, 1209, 1190, 1155, 1102, 725, 684, 668 cm^{-1}; HRMS (ESI): *m/z* calculated for $C_{40}H_{66}F_3N_8O_{11}S$ [*M*+H]$^+$: 923.4518, found: 923.4561.

(*S*)-2-((*S*)-4-Methyl-2-(2,2,2-trifluoroacetamido)pentanamido)-6-(14-oxo-18-((3a*S*,4*S*,6a*R*)-2-oxohexahydro-1*H*-thieno[3,4-*d*]imidazol-4-yl)-4,7,10-trioxa-13-azaoctadecanamido)-*N*-(2-oxo-2-(prop-2-yn-1-ylamino)ethyl)hexanamide (32, bio-b-25a)

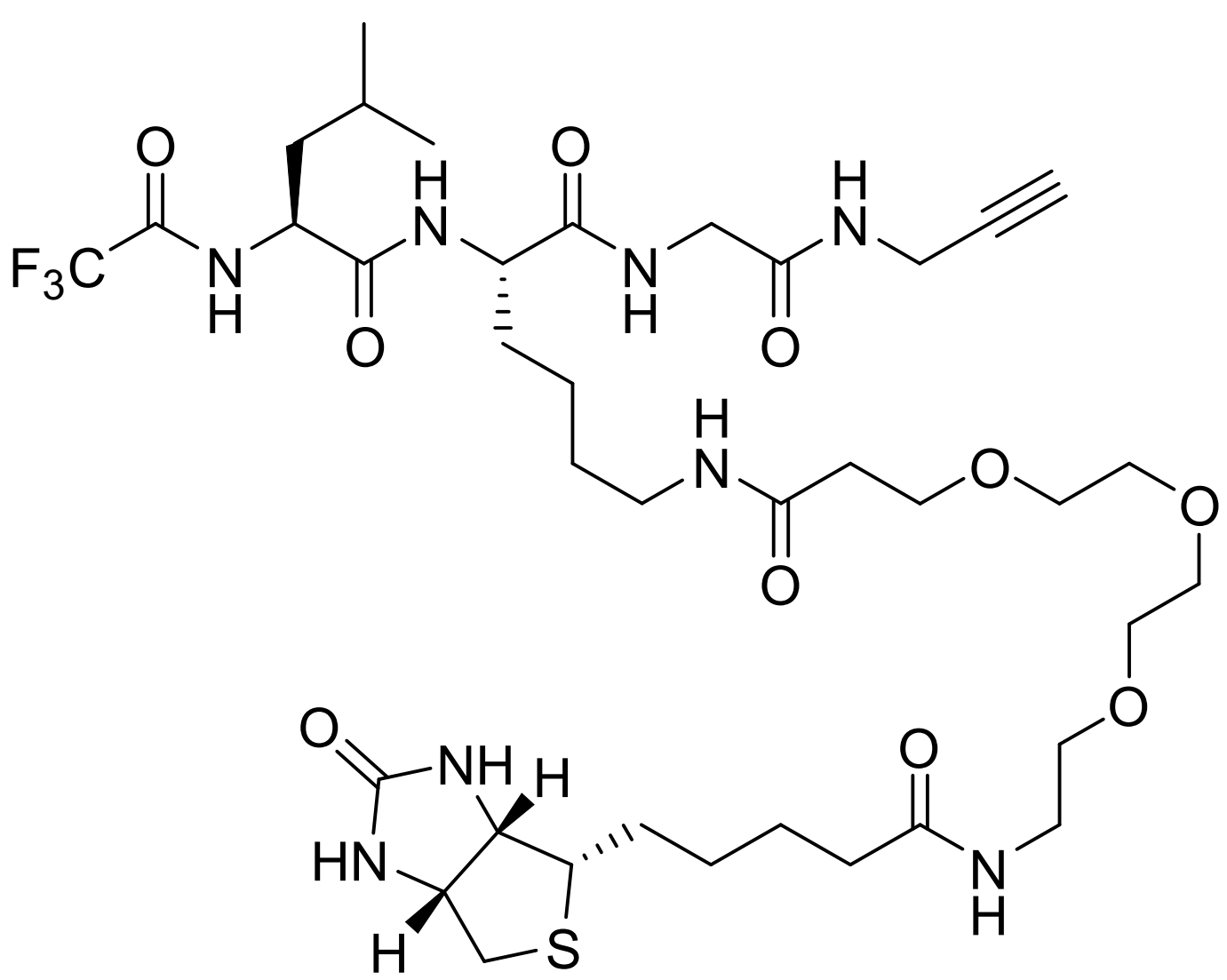

N-Boc tetrapeptide **24** (100.9 mg, 0.184 mmol) was deprotected according to General Procedure G, and was directly used in the following step: According to General Procedure **I**, *N*-PEG-biotinylated tetrapeptide **32** (40.0 mg, 30%) was obtained from commercially sourced Biotin-PEG3-NHS ester (0.8 equiv.), following high-performance liquid chromatography (Avantor® ACE® 10 AQ 250 × 21.2 mm; 20 mL/min), isocratic hold (9.4 min) 2%$_{v/v}$; linear gradient (77.6 min): 2%$_{v/v}$ → 98%$_{v/v}$ acetonitrile in water (each containing 0.1%$_{v/v}$ formic acid); t_R = 36.0 min.

White solid; ^{1}H NMR (400 MHz, DMSO-d_6): δ = 1H NMR (400 MHz, DMSO) δ 9.53 (d, *J* = 8.0 Hz, 1H), 8.26 – 8.19 (m, 2H), 8.17

(t, J = 5.8 Hz, 1H), 7.83 (t, J = 5.6 Hz, 1H), 7.78 (t, J = 5.6 Hz, 1H), 6.42 (s, 1H), 6.35 (s, 1H), 4.42 (ddd, J = 10.8, 7.9, 4.1 Hz, 1H), 4.34 – 4.25 (m, 1H), 4.19 (td, J = 8.2, 5.6 Hz, 1H), 4.12 (ddd, J = 7.8, 4.4, 1.9 Hz, 1H), 3.86 (dt, J = 5.1, 2.4 Hz, 2H), 3.68 (d, J = 5.8 Hz, 2H), 3.58 (t, J = 6.5 Hz, 2H), 3.54 – 3.42 (m, 8H), 3.38 (t, J = 5.9 Hz, 2H), 3.18 (q, J = 5.8 Hz, 2H), 3.12 – 3.06 (m, 2H), 3.00 (q, J = 6.6 Hz, 2H), 2.81 (dd, J = 12.4, 5.0 Hz, 1H), 2.57 (d, J = 12.4 Hz, 1H), 2.29 (t, J = 6.5 Hz, 2H), 2.06 (t, J = 7.4 Hz, 2H), 1.70 – 1.40 (m, 9H), 1.40 – 1.21 (m, 6H), 0.87 ppm (dd, J = 13.6, 6.1 Hz, 6H); ^{13}C NMR (151 MHz, DMSO-d_6): δ = 172.1, 171.7, 170.6, 169.8, 168.5, 162.7, 156.1 (q, J = 36.4 Hz), 115.9 (q, J = 288.2 Hz), 81.0, 73.1, 69.7, 69.6(7), 69.6, 69.5, 69.2, 66.9, 61.0, 59.2, 55.4, 52.8, 51.6, 41.7, 39.8, 39.2, 38.4, 38.3, 36.1, 35.1, 31.4, 28.8, 28.2, 28.0, 27.9, 25.3, 24.3, 23.0, 22.6, 21.1 ppm; ^{19}F NMR (376 MHz, DMSO-d_6): δ = −74.4 ppm (s, 3F); IR (film): ṽ = 3290 (br), 3080, 2930, 2870, 2361, 2341, 1650, 1548, 1460, 1350, 1209, 1190, 1156, 668 cm^{-1}; HRMS (ESI): *m/z* calculated for $C_{38}H_{61}F_3N_8O_{10}SK$ [*M*+K]$^+$: 917.3815, found: 917.3855.

tert-Butyl ((20*S*,23*S*)-20-isobutyl-5,18,21-trioxo-23-((2-oxo-2-(prop-2-yn-1-ylamino)ethyl)carbamoyl)-1-((3a*S*,4*S*,6a*R*)-2-oxohexahydro-1*H*-thieno[3,4-*d*]imidazol-4-yl)-9,12,15-trioxa-6,19,22-triazaheptacosan-27-yl)carbamate (33, bio-b-I38)

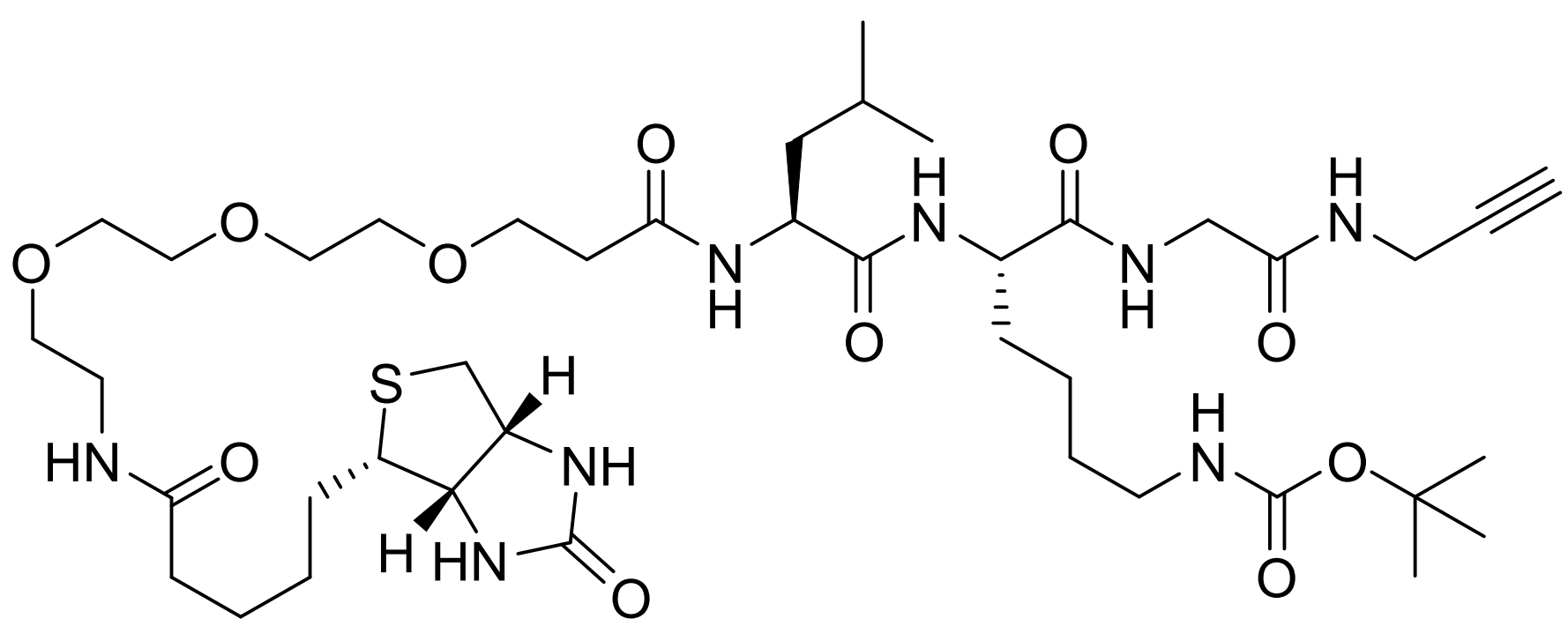

N-Fmoc tetrapeptide **14** (175.6 mg, 0.260 mmol) was deprotected according to General Procedure J, and was directly used in the following step: According to General Procedure I, *N*-PEG-biotinylated tetrapeptide **33** (90.9 mg, 40%) was obtained from commercially sourced Biotin-PEG3-NHS ester (1.0 equiv.), following high-performance liquid chromatography (Avantor® ACE® 10 AQ 250 × 21.2 mm; 20 mL/min), isocratic hold (5.3 min) 18%$_{v/v}$; linear gradient (46.9 min): 2%$_{v/v}$ → 98%$_{v/v}$ acetonitrile in water (each containing 0.1%$_{v/v}$ formic acid); t_R = 19.0 min.

White solid; ^{1}H NMR (600 MHz, DMSO-d_6): δ = 8.20 (t, J = 5.6 Hz, 1H), 8.11 (t, J = 5.9 Hz, 1H), 8.00 (d, J = 8.0 Hz, 1H), 7.94 (d, J = 7.5 Hz, 1H), 7.83 (t, J = 5.7 Hz, 1H), 6.73 (t, J = 5.7 Hz, 1H), 6.41 (d, J = 1.9 Hz, 1H), 6.35 (t, J = 1.4 Hz, 1H), 4.33 – 4.27 (m, 2H), 4.16 (dt, J = 7.9, 4.1 Hz, 1H), 4.12 (ddd, J = 7.7, 4.4, 1.8 Hz, 1H), 3.91 – 3.82 (m, 2H), 3.67 (qd, J = 16.7, 5.9 Hz, 2H), 3.62 – 3.55 (m, 2H), 3.52 – 3.44 (m, 8H), 3.38 (t, J = 6.0 Hz, 2H), 3.18 (q, J = 5.9 Hz, 2H), 3.12 – 3.05 (m, 2H), 2.87 (q, J = 6.7 Hz, 2H), 2.81 (dd, J = 12.4, 5.1 Hz, 1H), 2.57 (d, J = 12.4 Hz, 1H), 2.41 (dt, J = 14.0, 6.8 Hz, 1H), 2.33 (dt, J = 14.5, 6.3 Hz, 1H), 2.06 (t, J = 7.5 Hz, 2H), 1.67 – 1.57 (m, 3H), 1.55 – 1.41 (m, 6H), 1.36 (s, 9H), 1.34 – 1.16 (m, 6H), 0.88 (d, J = 6.6 Hz, 3H), 0.83 ppm (d, J = 6.6 Hz, 3H); ^{13}C NMR (151 MHz, DMSO-d_6): δ = 172.3, 172.2, 171.9, 170.3, 168.5, 162.7, 155.6, 81.0, 77.4, 73.0, 69.7(4), 69.7, 69.6, 69.5, 69.2, 66.8, 61.1, 59.2, 55.4, 52.7, 51.0, 41.8, 40.6, 39.9, 39.7, 38.5, 36.0, 35.1, 31.4, 29.2, 28.3 (3C), 28.2, 28.1, 27.9, 25.3, 24.1, 23.1, 22.6,

21.6 ppm; IR (film): ṽ = 3284 (br), 3078, 2934, 2870, 1649, 1536, 1458, 1366, 1252, 1169, 1098 cm^{-1}; HRMS (ESI): *m/z* calculated for $C_{41}H_{70}N_8O_{11}SK$ [*M*+K]$^+$: 921.4516, found: 921.4542.

***tert*-Butyl ((23*S*,26*S*)-23-isobutyl-5,21,24-trioxo-26-((2-oxo-2-(prop-2-yn-1-ylamino)ethyl)carbamoyl)-1-((3a*S*,4*S*,6a*R*)-2-oxohexahydro-1*H*-thieno[3,4-*d*]imidazol-4-yl)-9,12,15,18-tetraoxa-6,22,25-triazatriacontan-30-yl)carbamate (34, bio-a-I38)**

N-Fmoc tetrapeptide **14** (162.9 mg, 0.241 mmol) was deprotected according to General Procedure J, and was directly used in the following step: According to General Procedure I, *N*-PEG-biotinylated tetrapeptide **34** (137.7 mg, 65%) was obtained from commercially sourced Biotin-PEG4-NHS ester (0.95 equiv.), following high-performance liquid chromatography (Avantor® ACE® 10 AQ 250 × 21.2 mm; 20 mL/min), isocratic hold (5.3 min) 18%$_{v/v}$; linear gradient (46.9 min): 2%$_{v/v}$ → 98%$_{v/v}$ acetonitrile in water (each containing 0.1%$_{v/v}$ formic acid); t_R = 19.0 min.

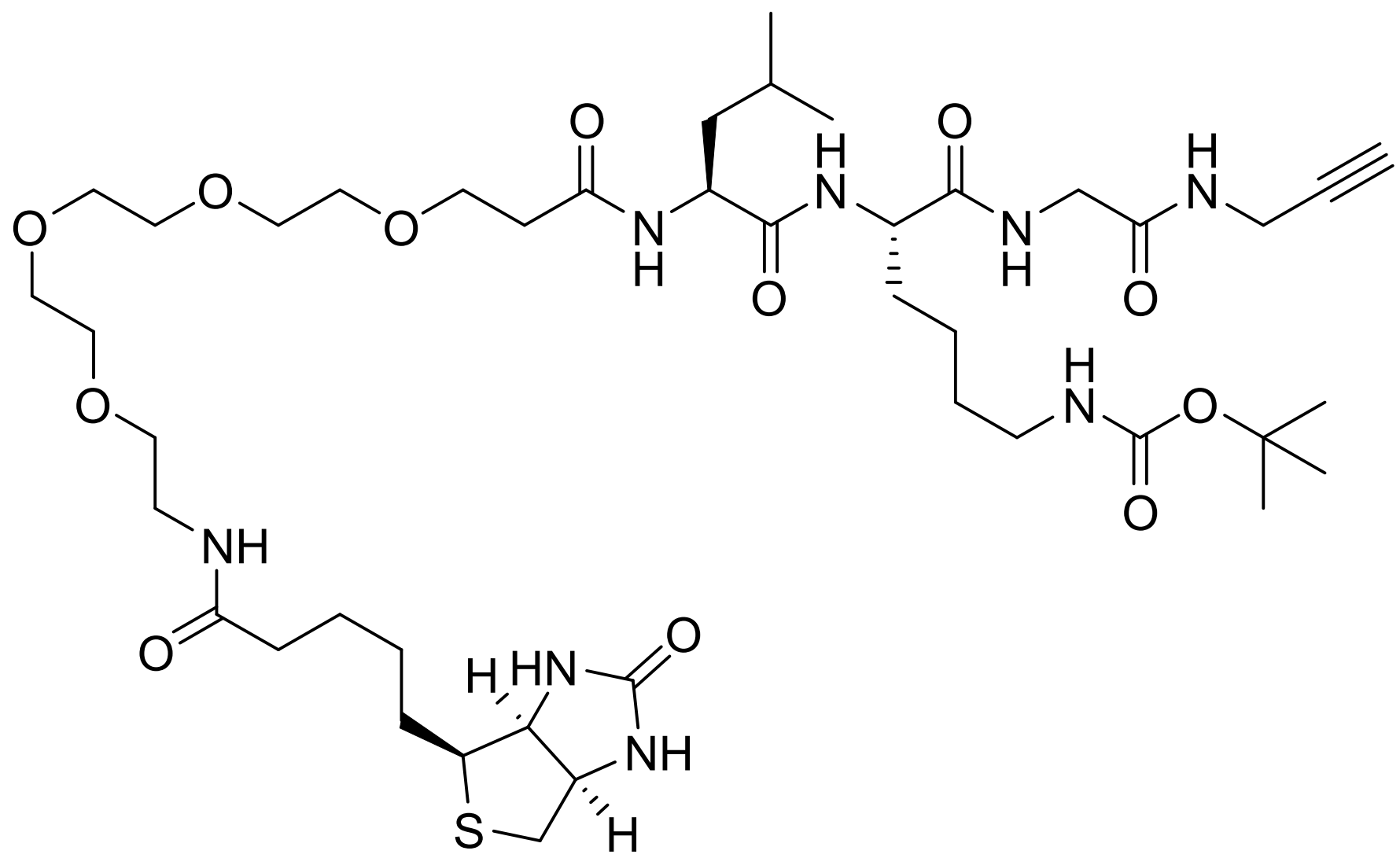

White solid; ^1H NMR (600 MHz, DMSO-d_6): δ = 8.20 (t, *J* = 5.6 Hz, 1H), 8.11 (t, *J* = 5.9 Hz, 1H), 8.00 (d, *J* = 8.0 Hz, 1H), 7.94 (d, *J* = 7.5 Hz, 1H), 7.83 (t, *J* = 5.7 Hz, 1H), 6.73 (t, *J* = 5.7 Hz, 1H), 6.41 (t, *J* = 1.9 Hz, 1H), 6.35 (t, *J* = 1.5 Hz, 1H), 4.33 – 4.26 (m, 2H), 4.18 – 4.10 (m, 2H), 3.86 (ddd, *J* = 5.6, 4.1, 2.5 Hz, 2H), 3.67 (qd, *J* = 16.7, 5.9 Hz, 2H), 3.73 – 3.54 (m, 2H), 3.53 – 3.42 (m, 12H), 3.39 (t, *J* = 5.9 Hz, 2H), 3.18 (q, *J* = 5.9 Hz, 2H), 3.12 – 3.05 (m, 2H), 2.87 (q, *J* = 6.8 Hz, 2H), 2.81 (dd, *J* = 12.4, 5.1 Hz, 1H), 2.57 (d, *J* = 12.4 Hz, 1H), 2.41 (dt, *J* = 13.9, 6.8 Hz, 1H), 2.33 (dt, *J* = 14.5, 6.3 Hz, 1H), 2.06 (t, *J* = 7.5 Hz, 2H), 1.68 – 1.56 (m, 3H), 1.56 – 1.39 (m, 6H), 1.36 (s, 9H), 1.36 – 1.14 (m, 6H), 0.88 (d, *J* = 6.6 Hz, 3H), 0.83 ppm (d, *J* = 6.5 Hz, 3H); ^{13}C NMR (151 MHz, DMSO-d_6): δ = 172.3, 172.2, 171.9, 170.3, 168.5, 162.8, 155.6, 81.0, 77.4, 73.0, 69.8(1) (2C), 69.8, 69.7, 69.6, 69.5, 69.2, 66.9, 61.1, 59.2, 55.4, 52.7, 51.0, 41.8, 40.6, 39.9, 39.7, 38.5, 36.0, 35.1, 31.4, 29.2, 28.3 (3C), 28.2, 28.1, 27.9, 25.3, 24.1, 23.1, 22.6, 21.6 ppm; IR (film): ṽ = 3296 (br), 3082, 2931, 2869, 1650, 1540, 1457, 1366, 1251, 1168, 1101 cm^{-1}; HRMS (ESI): *m/z* calculated for $C_{43}H_{75}N_8O_{12}S$ [*M*+H]$^+$: 927.5220, found: 927.5229.

***N*-((*S*)-4-Methyl-1-oxo-1-(((*S*)-1-oxo-1-((2-oxo-2-(prop-2-yn-1-ylamino)ethyl)amino)-6-(2,2,2-trifluoroacetamido)hexan-2-yl)amino)pentan-2-yl)-1-(5-((3a*S*,4*S*,6a*R*)-2-oxohexahydro-1*H*-**

thieno[3,4-*d*]imidazol-4-yl)pentanamido)-3,6,9,12-tetraoxapentadecan-15-amide (35, bio-a-25b)

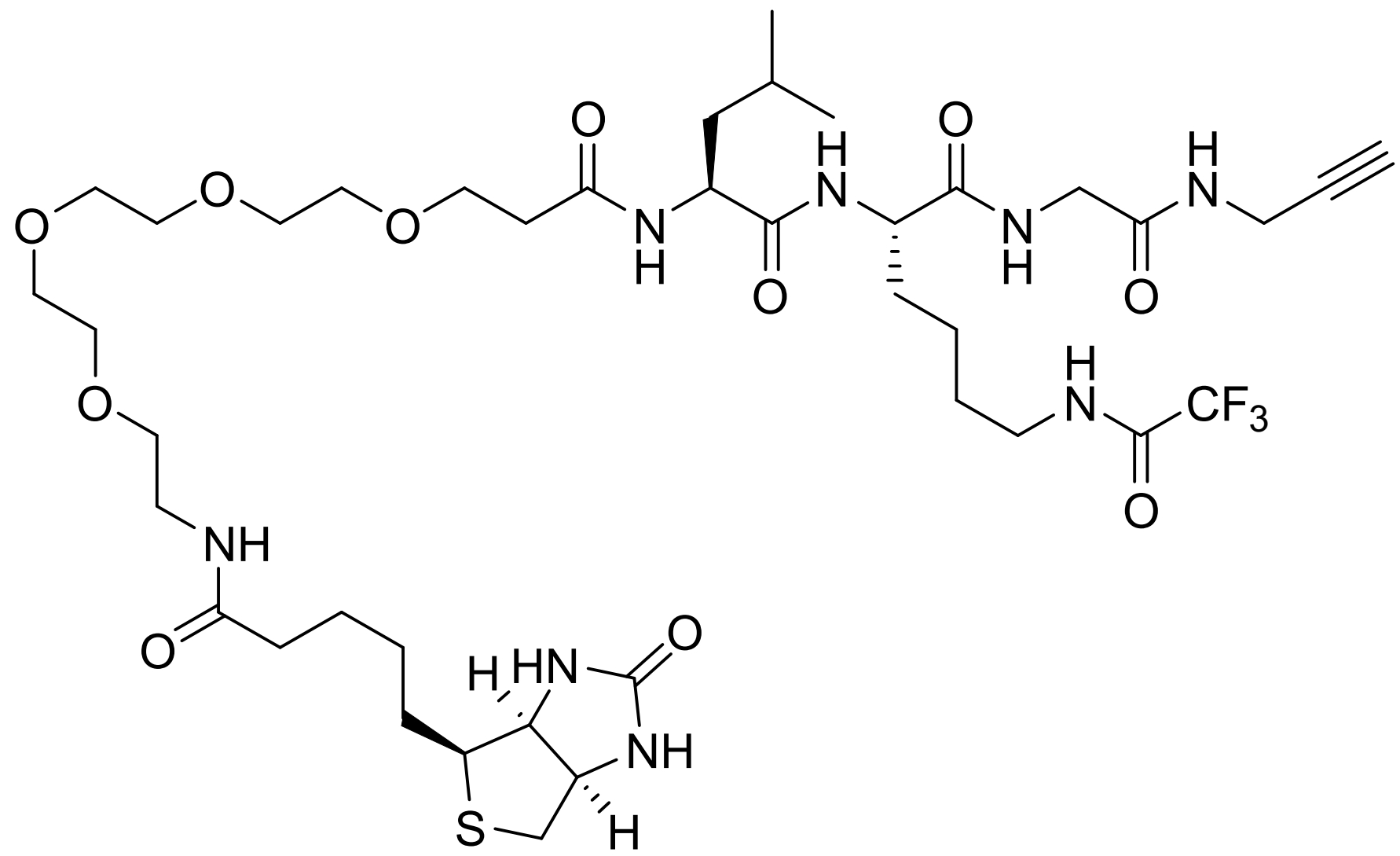

N-Boc tetrapeptide **34** (133.8 mg, 0.144. mmol) was deprotected according to General Procedure G. Commercially sourced 4-(*N,N*-dimethylamino)pyridine (1.05 equiv.), anhydrous tetrahydrofuran (0.09 M) and anhydrous N,N-dimethylformamide (0.27 M) were added. At 0 °C, commercially sourced trifluoroacetic anhydride (1.2 equiv.) was added, and twice more at ambient temperature after 1 h and after at 15 h (1.2 equiv. each). After stirring for 20h, the reaction mixture was concentrated under reduced pressure, and purified via high-performance liquid chromatography (Avantor® ACE® 10 AQ 250 × 21.2 mm; 20 mL/min) isocratic hold (9.9 min) $2\%_{v/v}$; linear gradient (16.2 min): $2\%_{v/v} \rightarrow 15\%_{v/v}$; isocratic hold (30.6 min) $15\%_{v/v}$; linear gradient (52.7 min): $15\%_{v/v} \rightarrow 98\%_{v/v}$ acetonitrile in water (each containing $0.1\%_{v/v}$ formic acid); t_R = 69.0 min, followed by high-performance liquid chromatography (Avantor® ACE® 10 AQ 250 × 21.2 mm; 20 mL/min) isocratic hold (9.7 min) $2\%_{v/v}$; linear gradient (15.8 min): $2\%_{v/v} \rightarrow 15\%_{v/v}$; isocratic hold (24.8 min) $15\%_{v/v}$; linear gradient (26.1 min): $15\%_{v/v} \rightarrow 34\%_{v/v}$; linear gradient (33.1 min): $34\%_{v/v} \rightarrow 98\%_{v/v}$ acetonitrile in water (each containing $0.1\%_{v/v}$ formic acid) t_R = 73.0 min, to give the peptide **35** (2.5 mg, 2%).

White solid; ^{1}H NMR (600 MHz, DMSO-d_6): δ = 9.40 (t, *J* = 5.7 Hz, 1H), 8.21 (t, *J* = 5.6 Hz, 1H), 8.12 (t, *J* = 5.9 Hz, 1H), 8.02 (d, *J* = 8.0 Hz, 1H), 7.97 (d, *J* = 7.6 Hz, 1H), 7.83 (t, *J* = 5.7 Hz, 1H), 6.41 (t, *J* = 1.9 Hz, 1H), 6.35 (s, 1H), 4.33 – 4.26 (m, 2H), 4.23 – 4.14 (m, 1H), 4.12 (ddd, *J* = 7.8, 4.4, 1.9 Hz, 1H), 3.90 – 3.82 (m, 2H), 3.67 (qd, *J* = 16.6, 5.9 Hz, 2H), 3.62 – 3.54 (m, 2H), 3.53 – 3.44 (m, 12H), 3.39 (t, *J* = 5.9 Hz, 2H), 3.18 (q, *J* = 6.0 Hz, 2H), 3.14 (q, *J* = 6.5 Hz, 2H), 3.12 – 3.06 (m, 2H), 2.82 (dd, *J* = 12.4, 5.1 Hz, 1H), 2.57 (d, *J* = 12.4 Hz, 1H), 2.45 – 2.38 (m, 1H), 2.33 (dt, *J* = 14.5, 6.3 Hz, 1H), 2.06 (t, *J* = 7.4 Hz, 2H), 1.71 – 1.53 (m, 4H), 1.52 – 1.41 (m, 7H), 1.37 – 1.23 (m, 4H), 0.88 (d, *J* = 6.6 Hz, 3H), 0.84 ppm (d, *J* = 6.6 Hz, 3H); ^{13}C NMR (151 MHz, DMSO-d_6): δ = 172.3, 172.1, 171.8, 170.2, 168.5, 162.7, 156.1 (q, *J* = 36.0 Hz), 116.0 (q, *J* = 288.2 Hz), 81.0, 73.0, 69.8 (2C), 69.7(1), 69.7, 69.6, 69.5, 69.2, 66.8, 61.0, 59.2, 55.4, 52.5, 51.0, 41.8, 40.6, 40.4, 40.1, 38.4, 35.9, 35.1, 31.2, 28.2, 28.0, 27.9(2), 27.9, 25.3, 24.1, 23.1, 22.5, 21.5 ppm; ^{19}F NMR (565 MHz, DMSO-d_6): δ = −74.4 ppm (s, 3F); IR (film): ṽ = 3306 (br), 3080, 2971, 2931, 2360, 2341, 1652, 1550, 1465, 1380, 1355, 1249, 1204, 1162, 1130, 1105 cm^{-1}; HRMS (ESI): *m/z* calculated for $C_{40}H_{66}F_3N_8O_{11}S$ $[M+H]^+$: 923.4518, found: 923.4541.

^{1}H, ^{13}C, and ^{19}F NMR spectra of final compounds

Compound **25d**

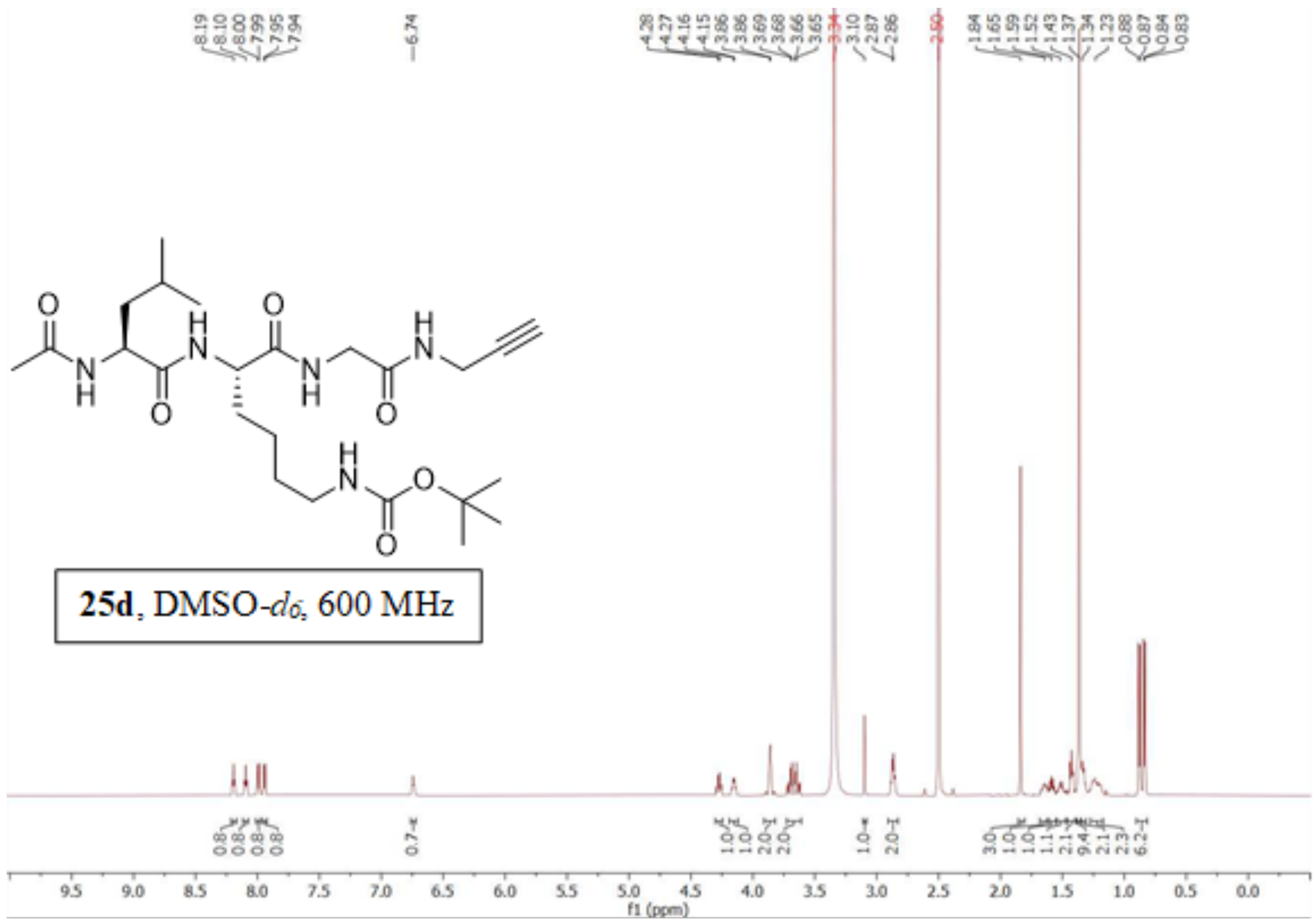

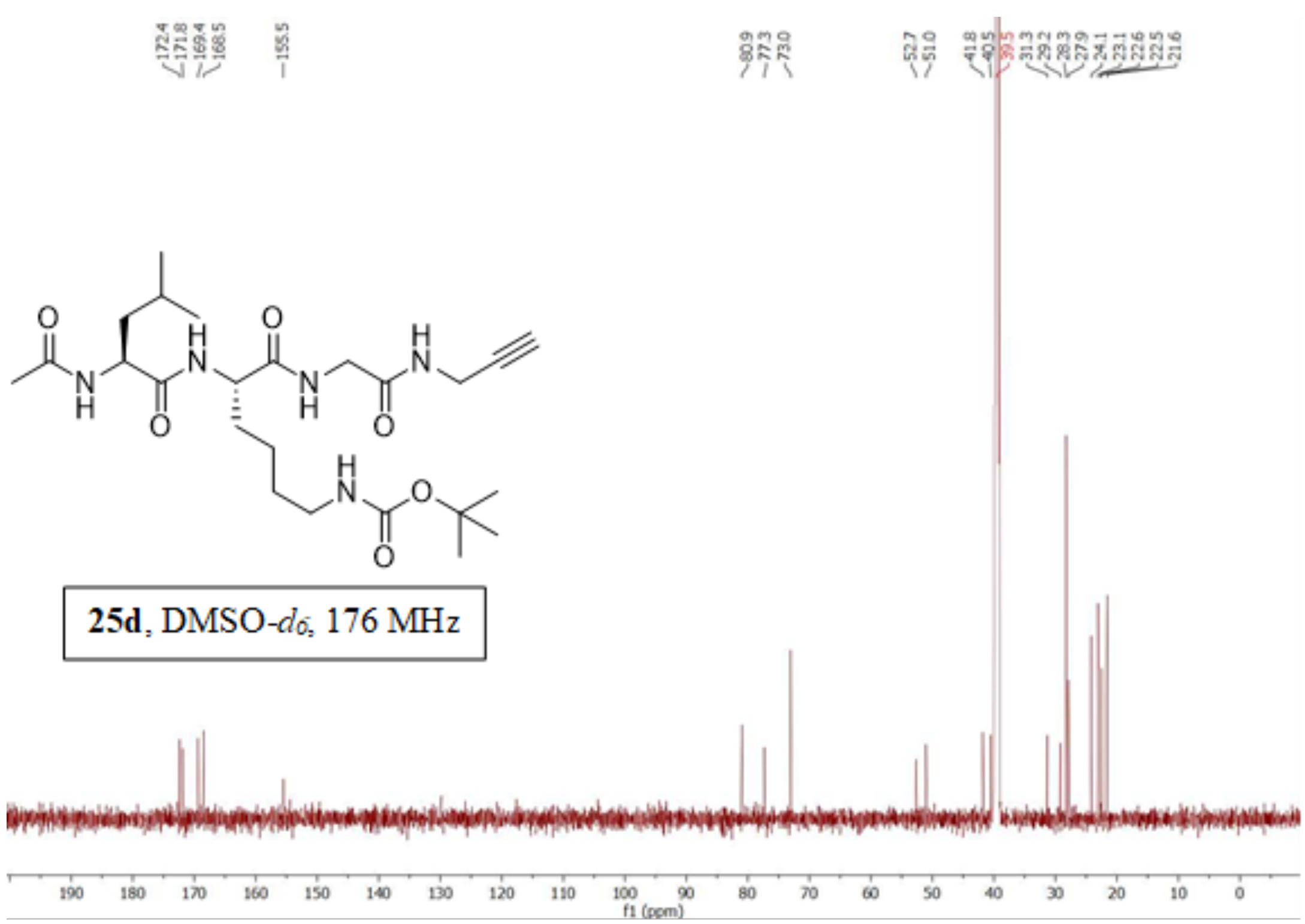

Compound **25e**

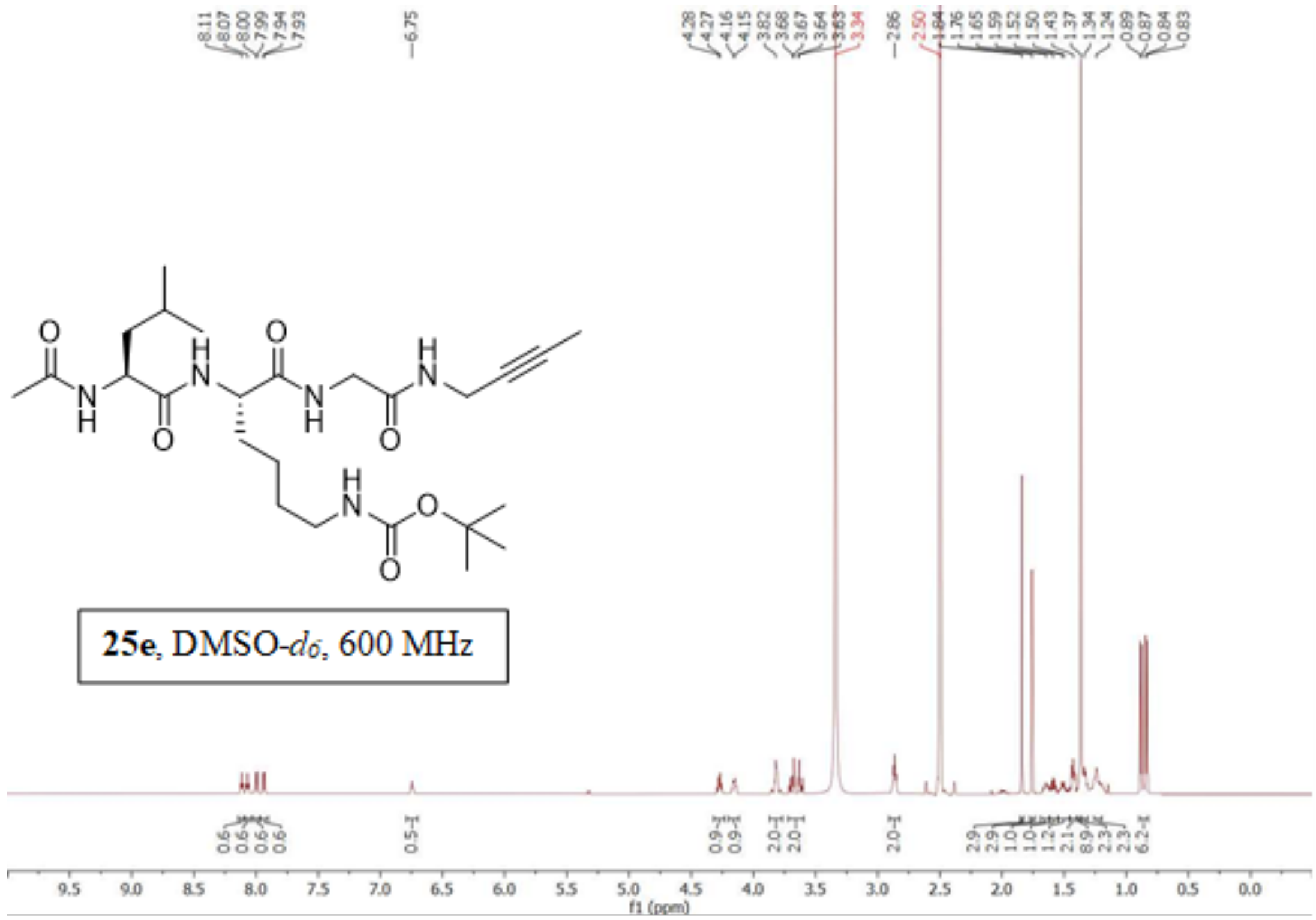

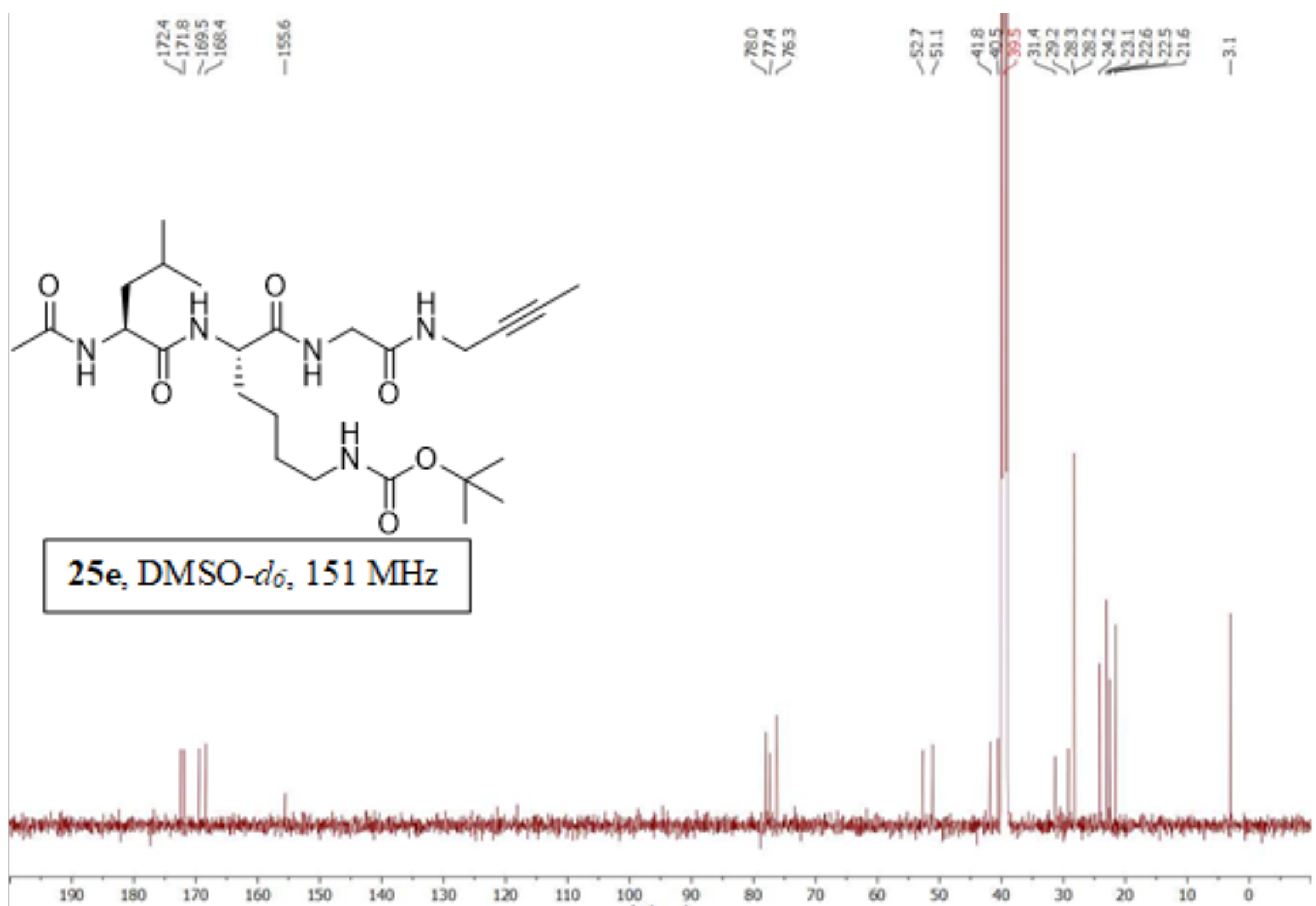

Compound **25f**

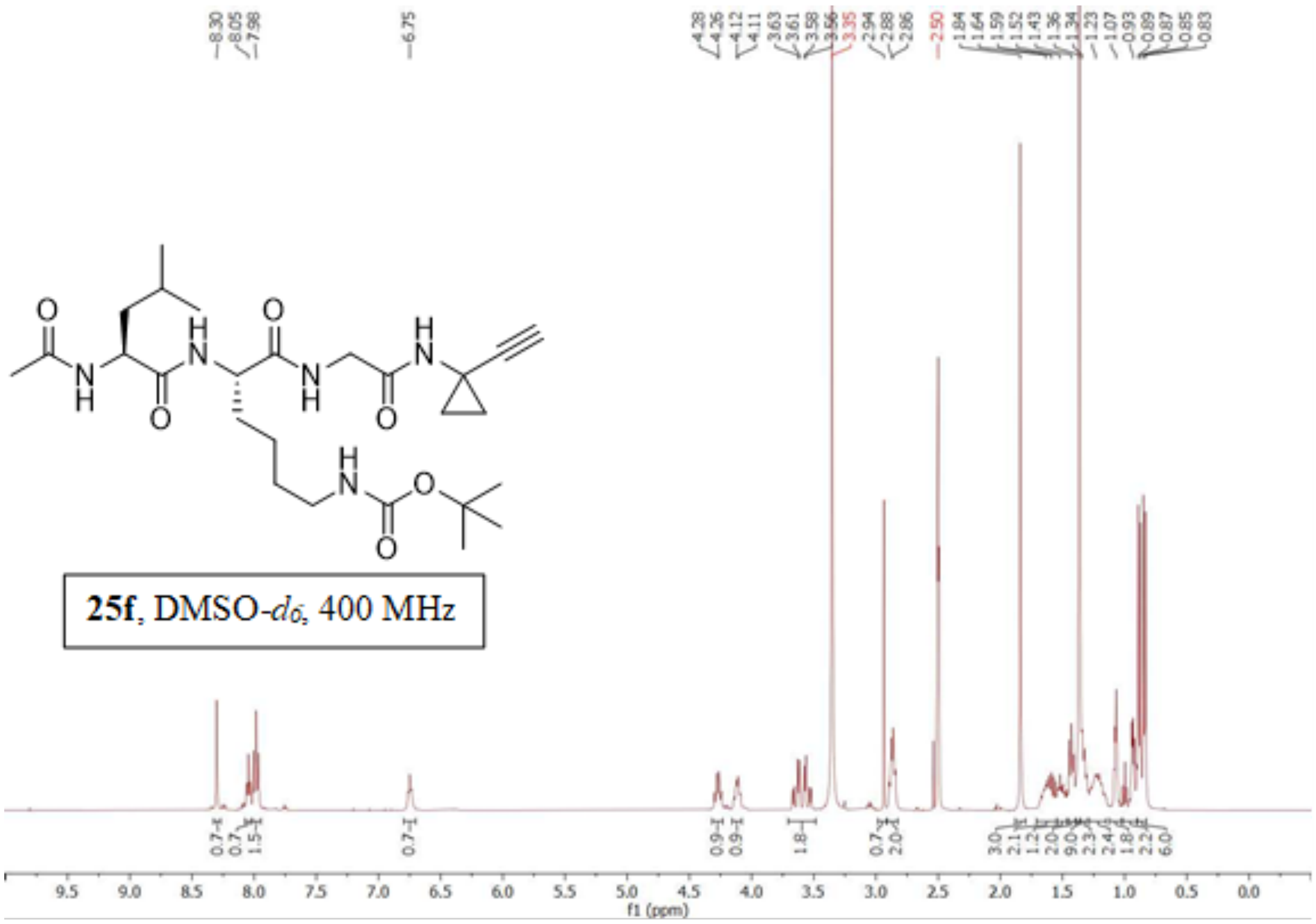

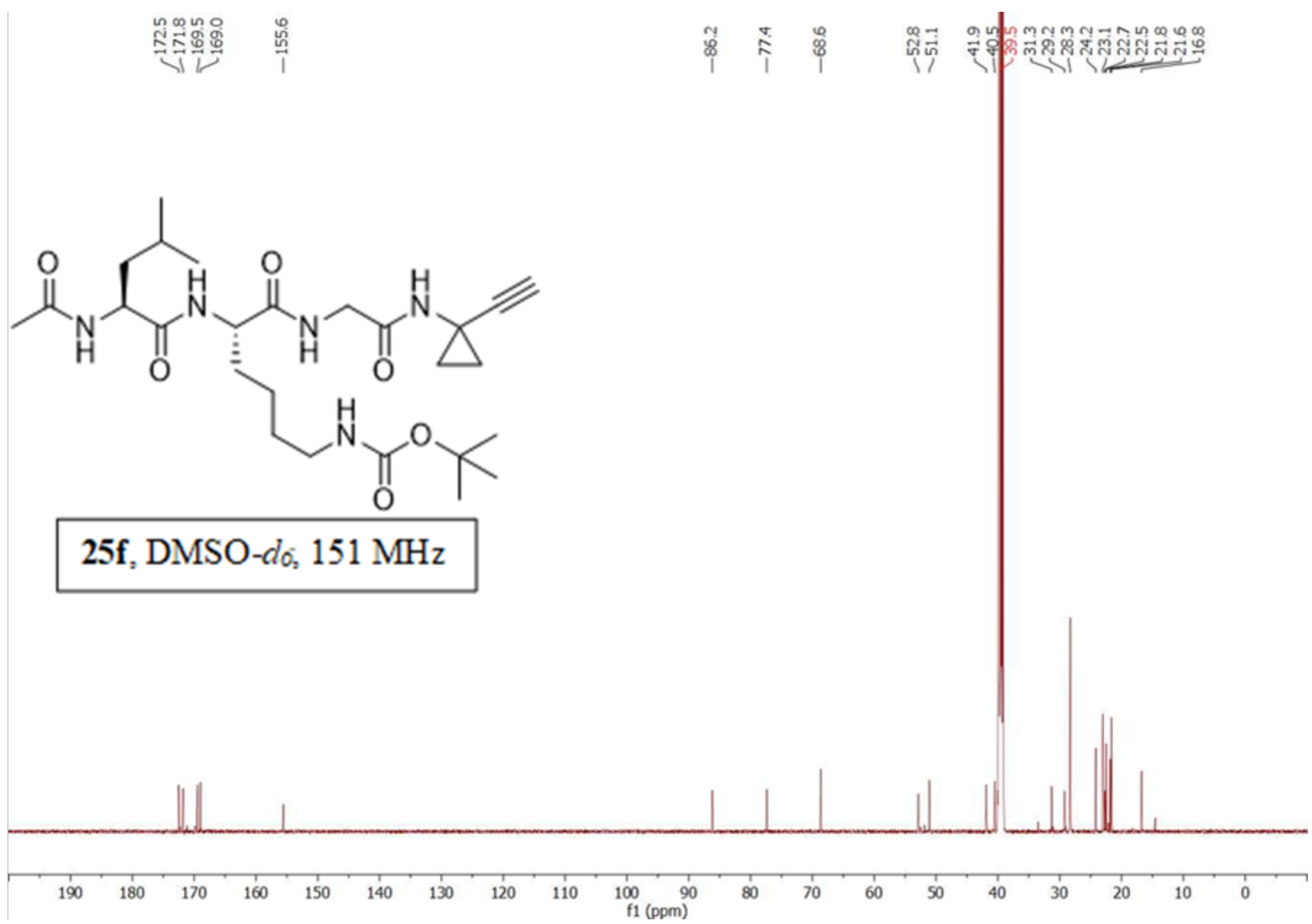

Compound **25c**

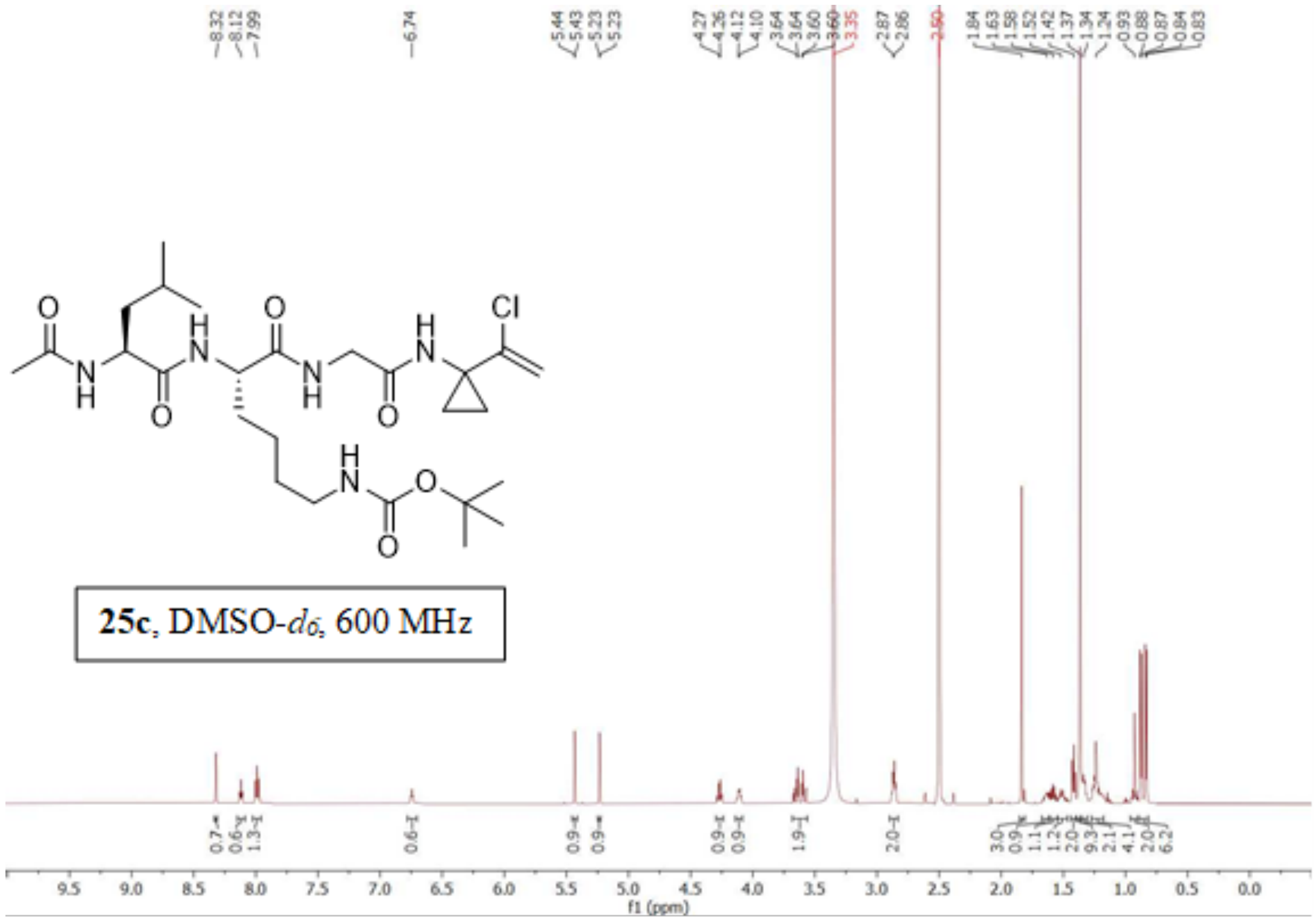

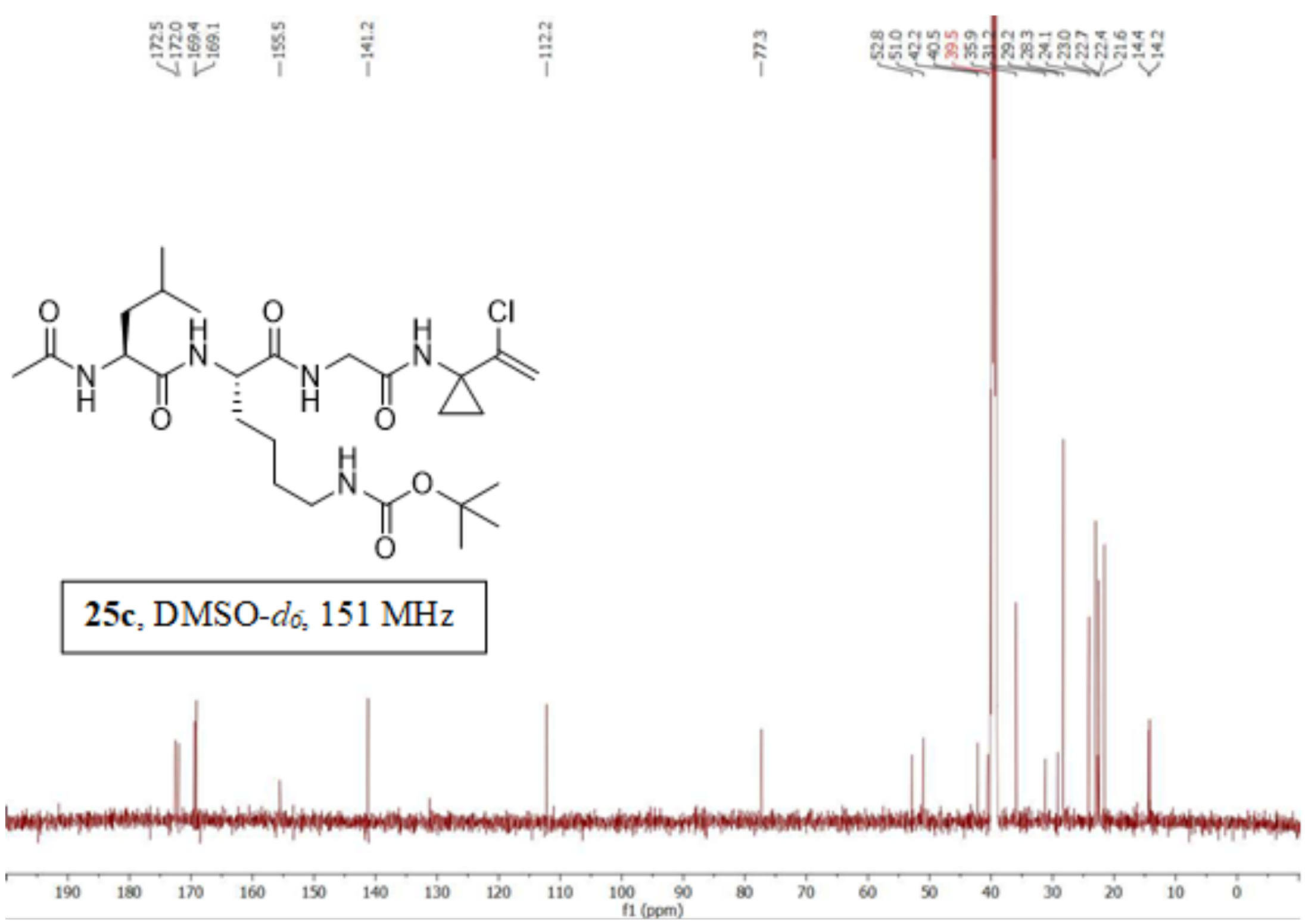

Compound **25g**

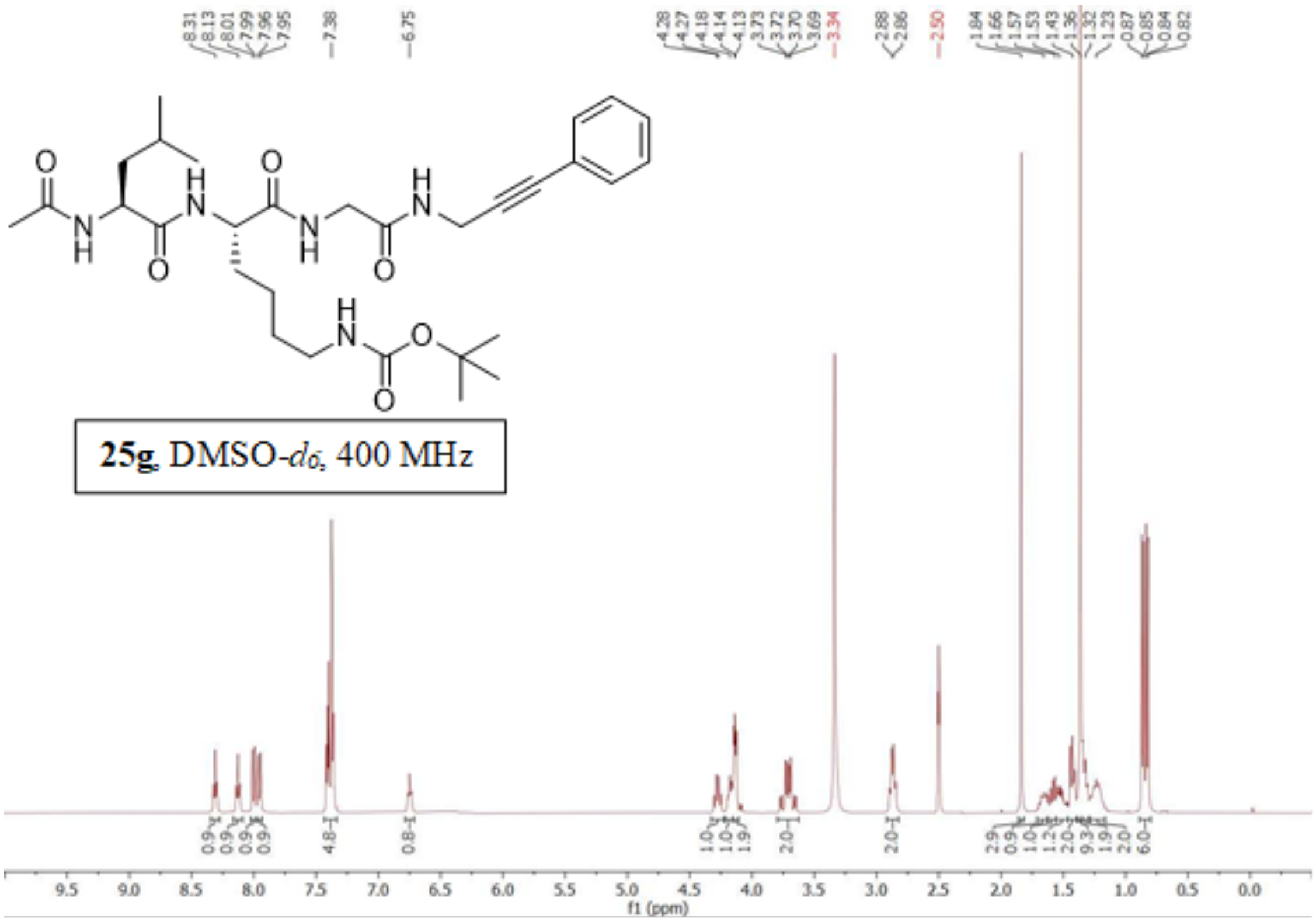

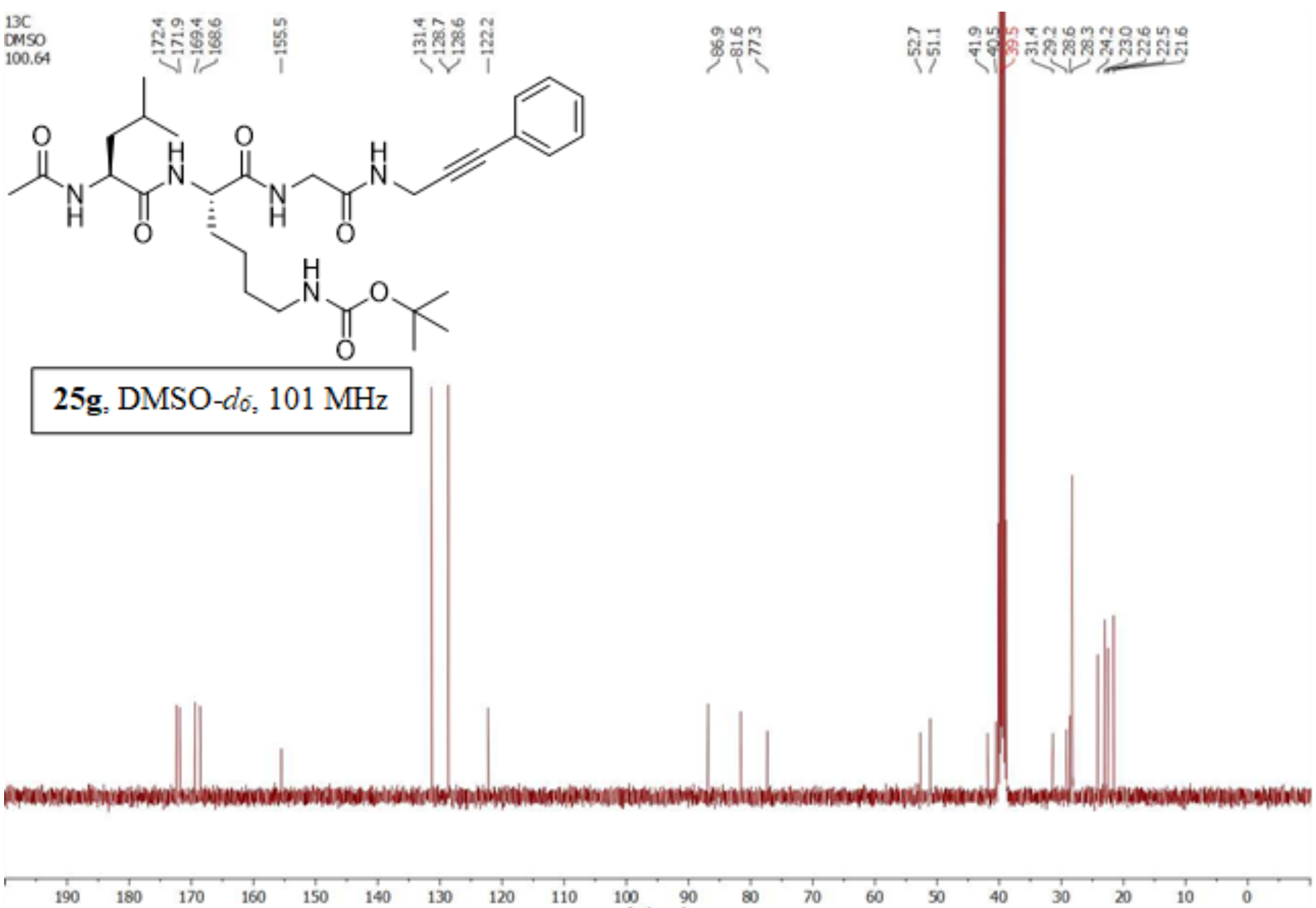

Compound **25a**

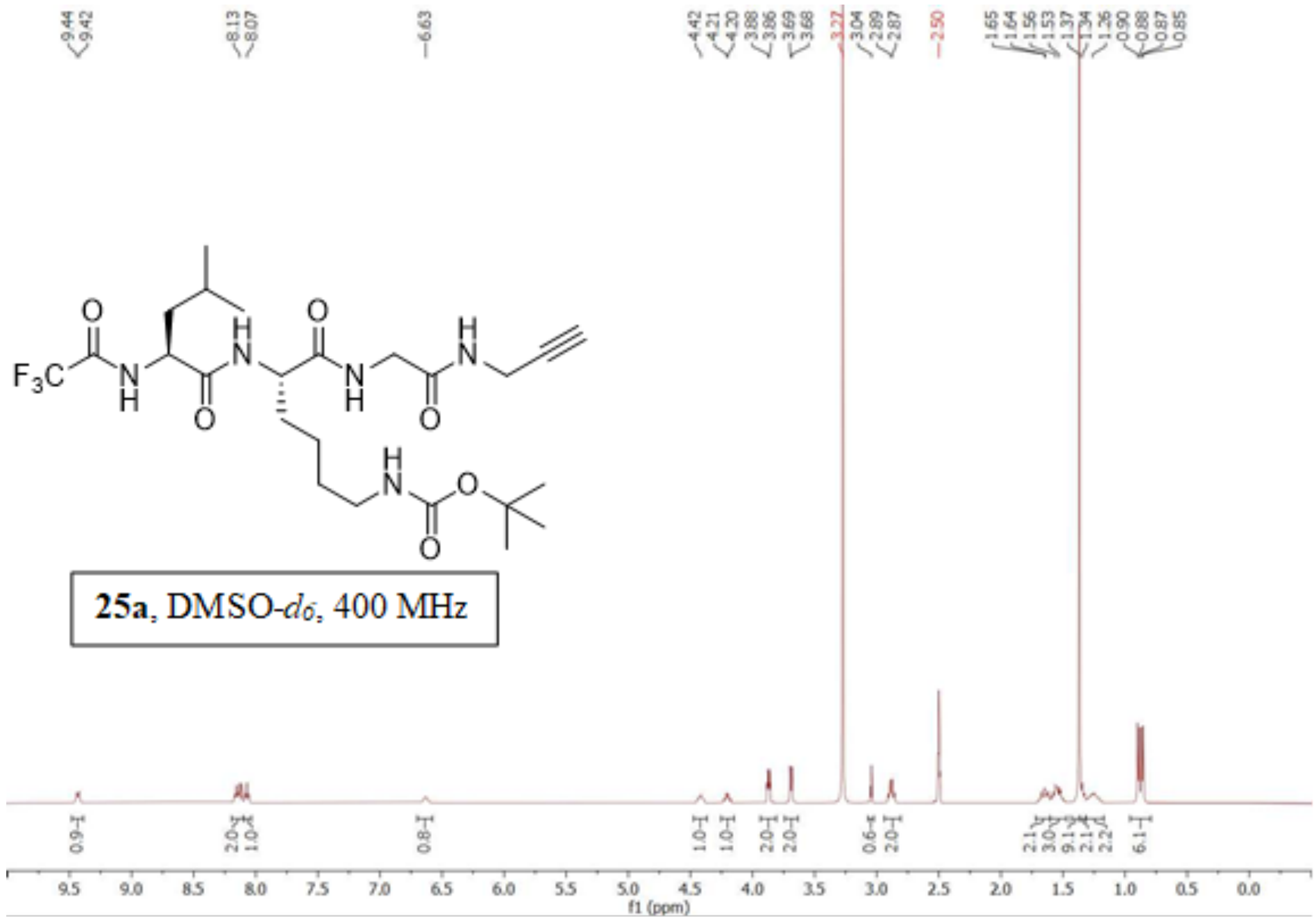

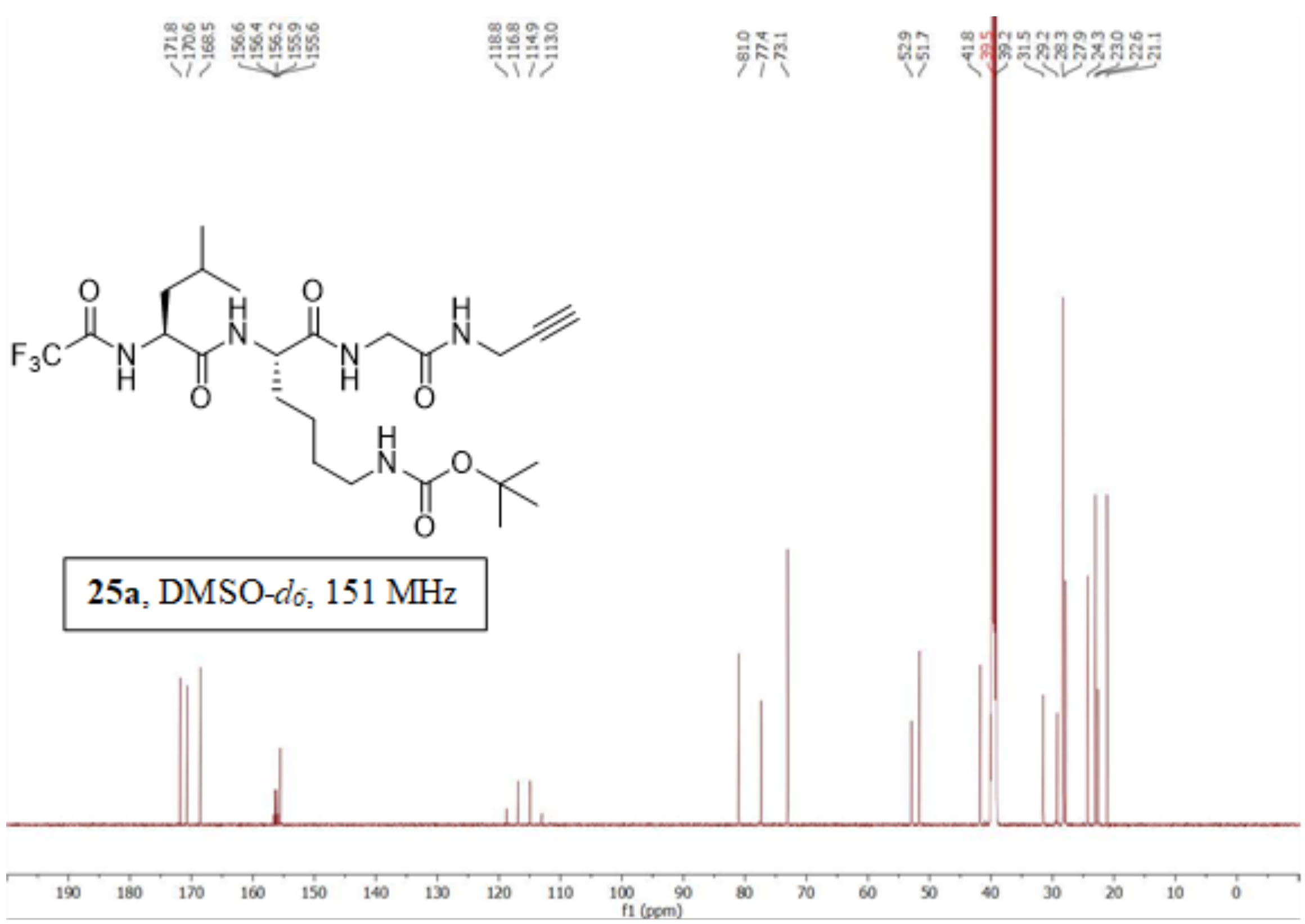

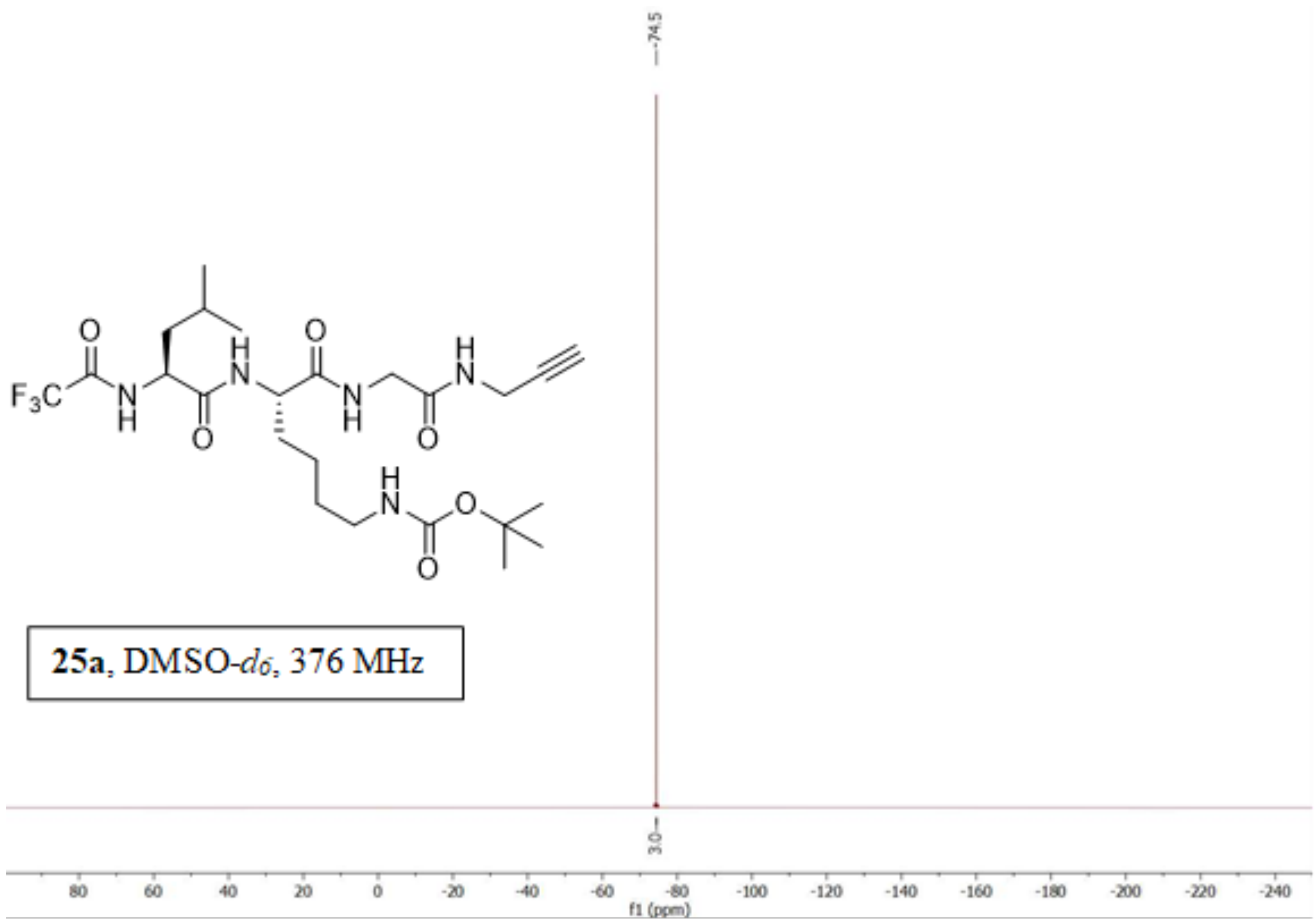

25a, DMSO-*d*6, 376 MHz

Compound **25**

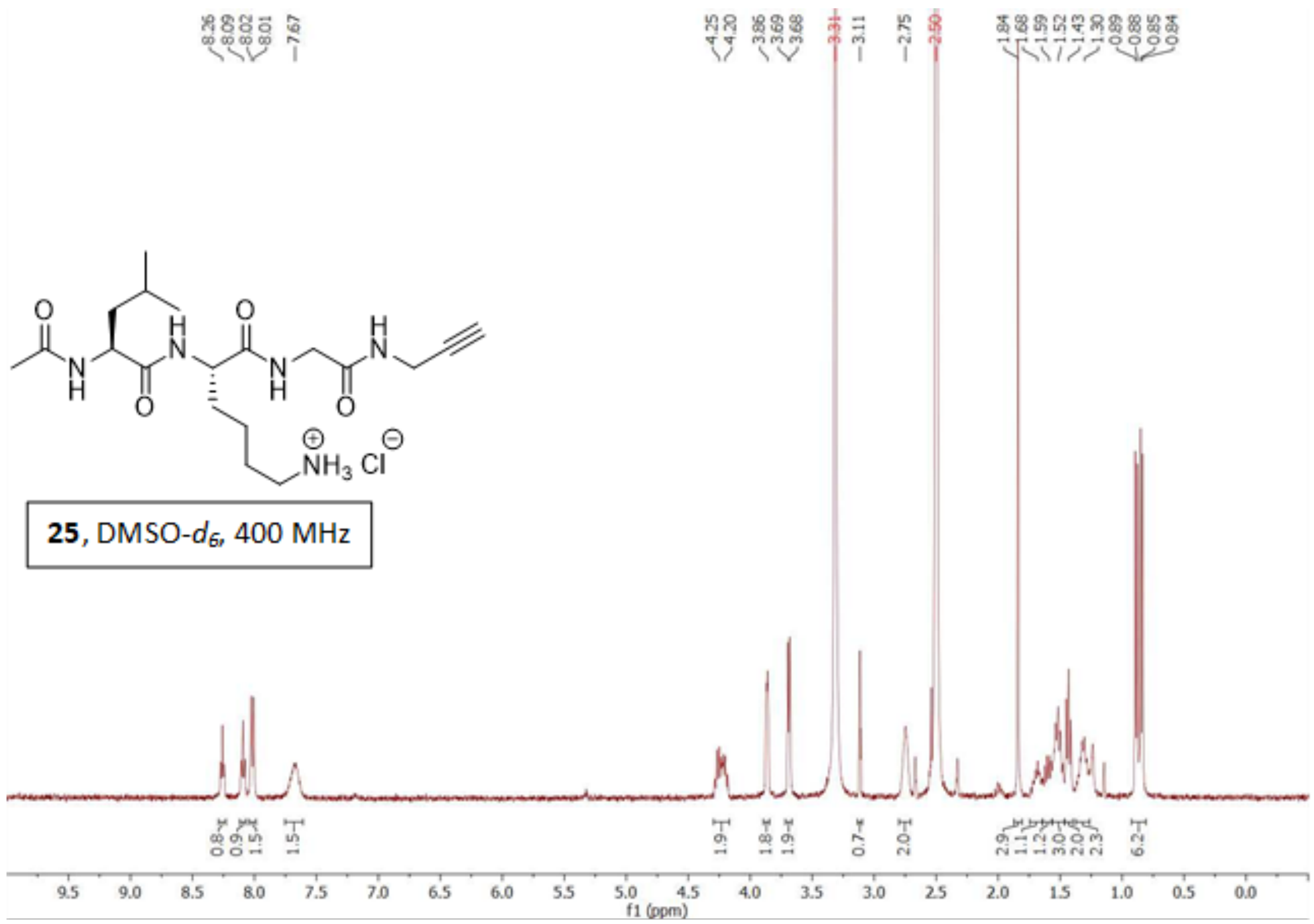

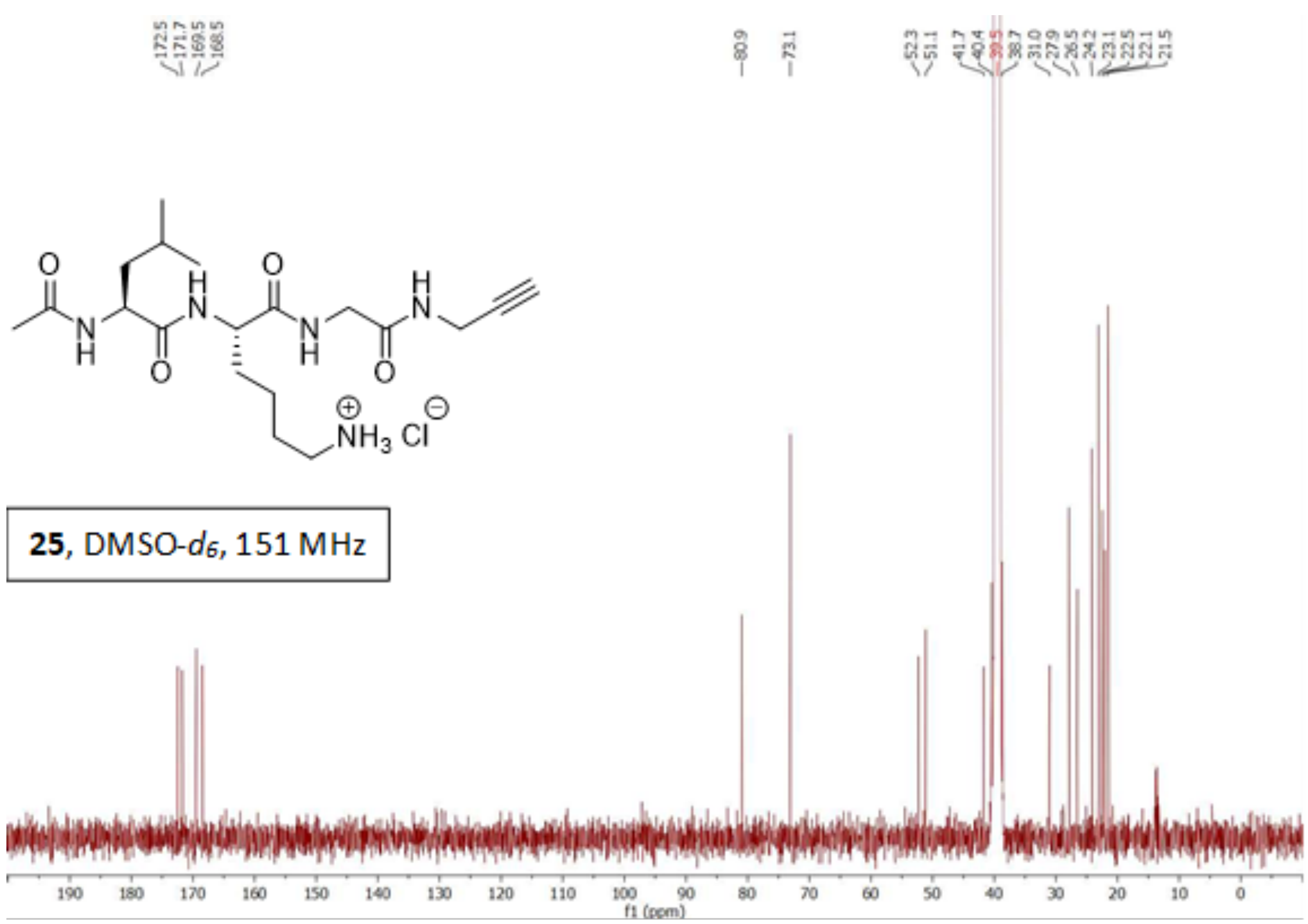

Compound **25j**

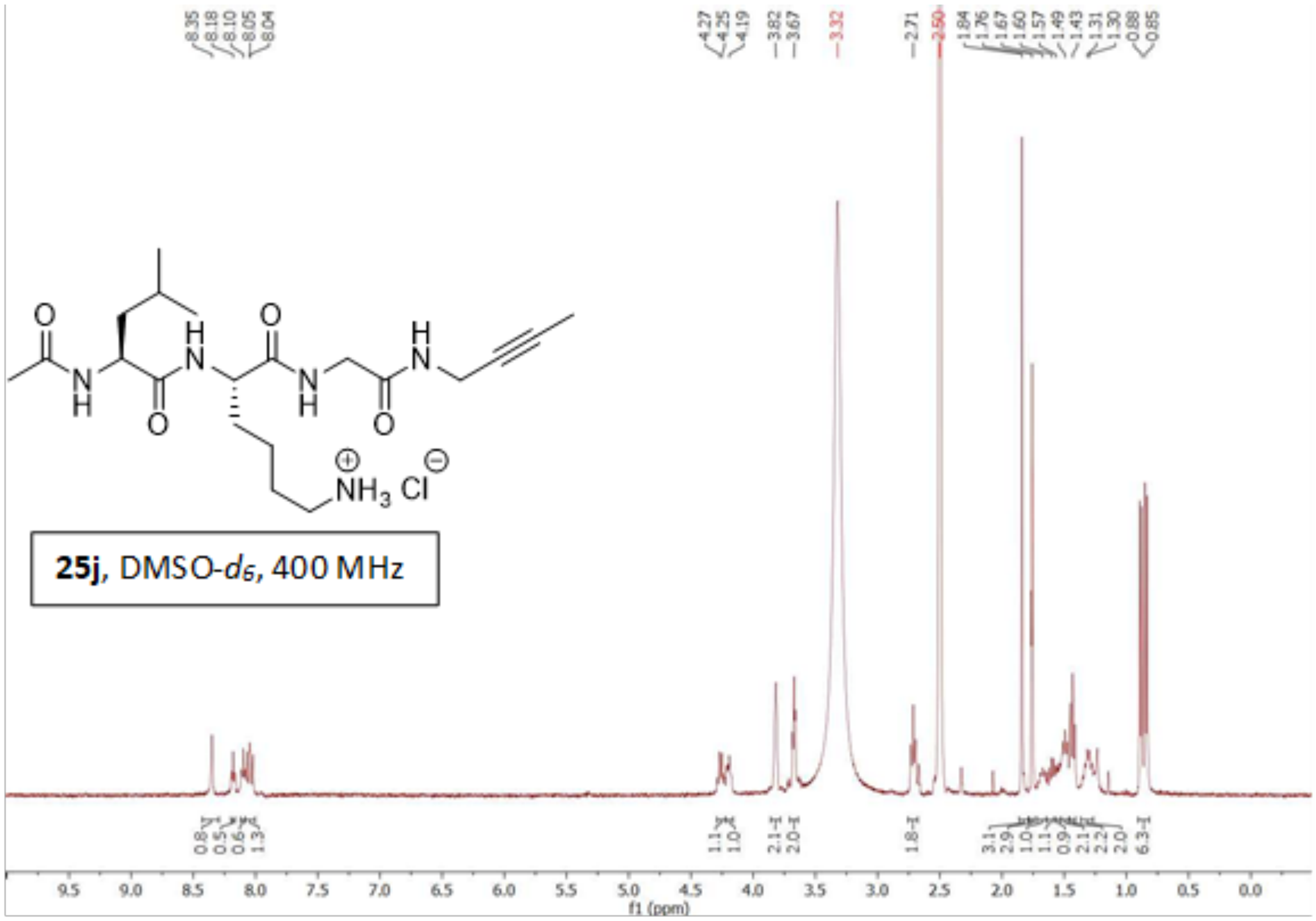

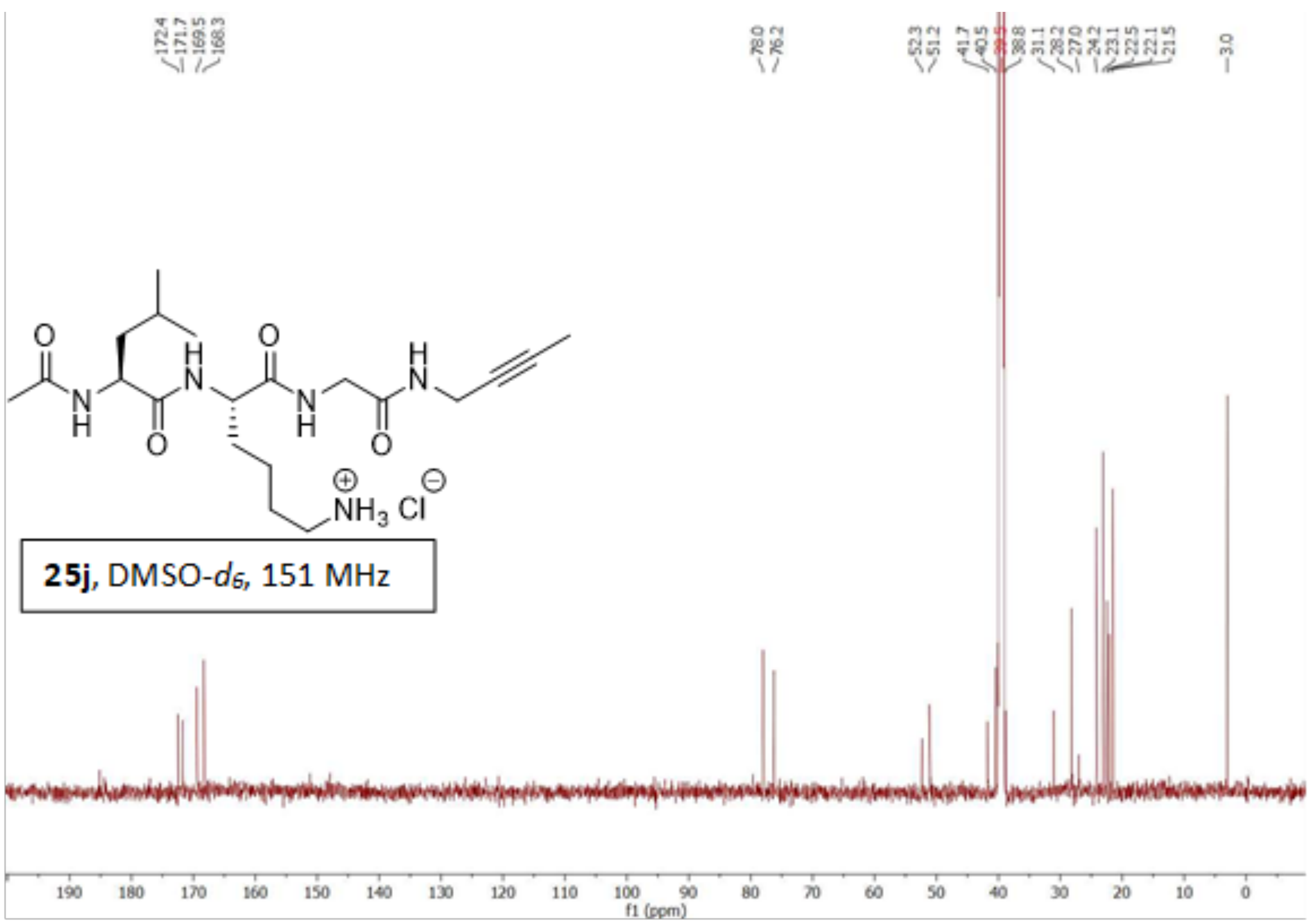

Compound **25h**

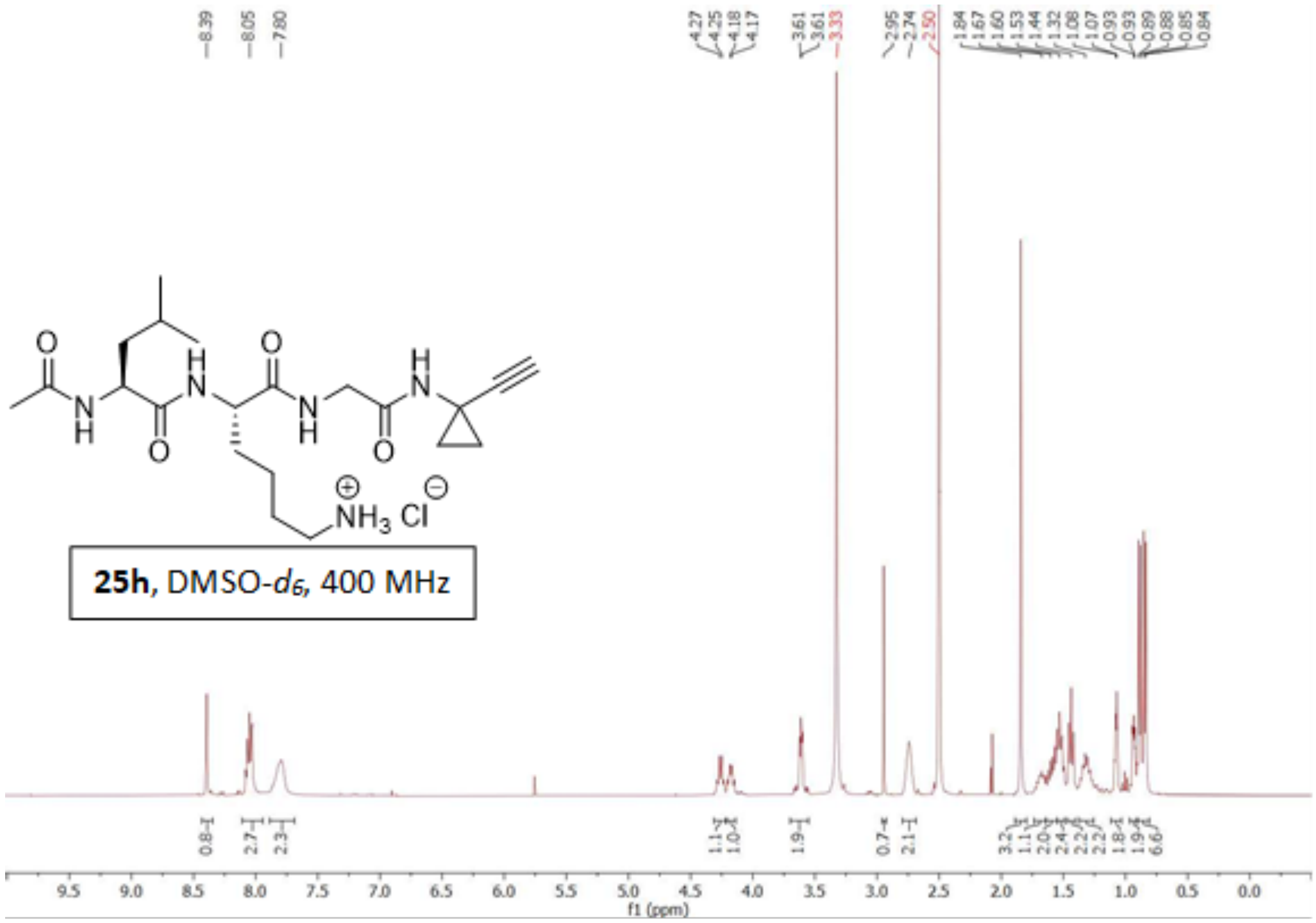

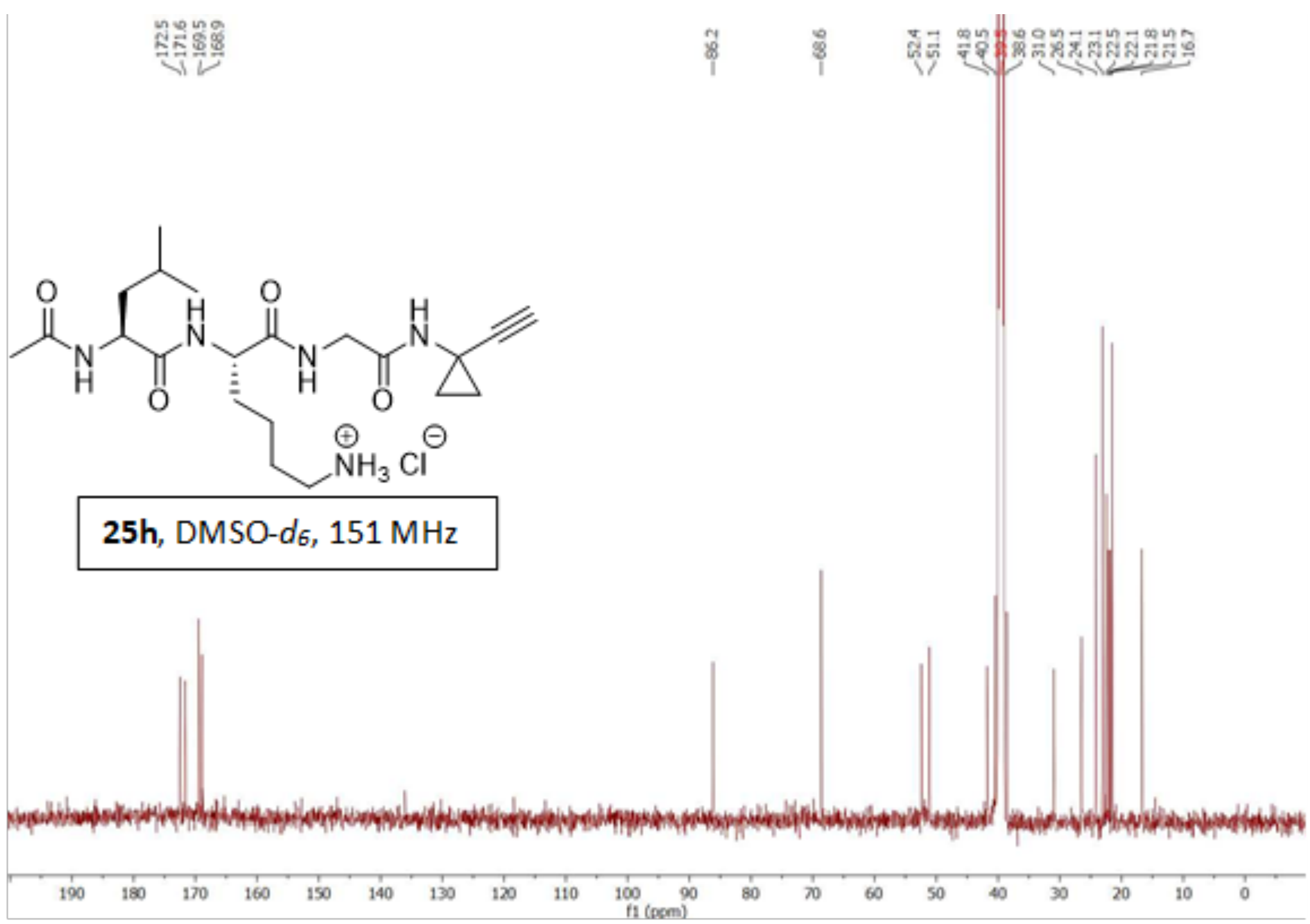

Compound **25i**

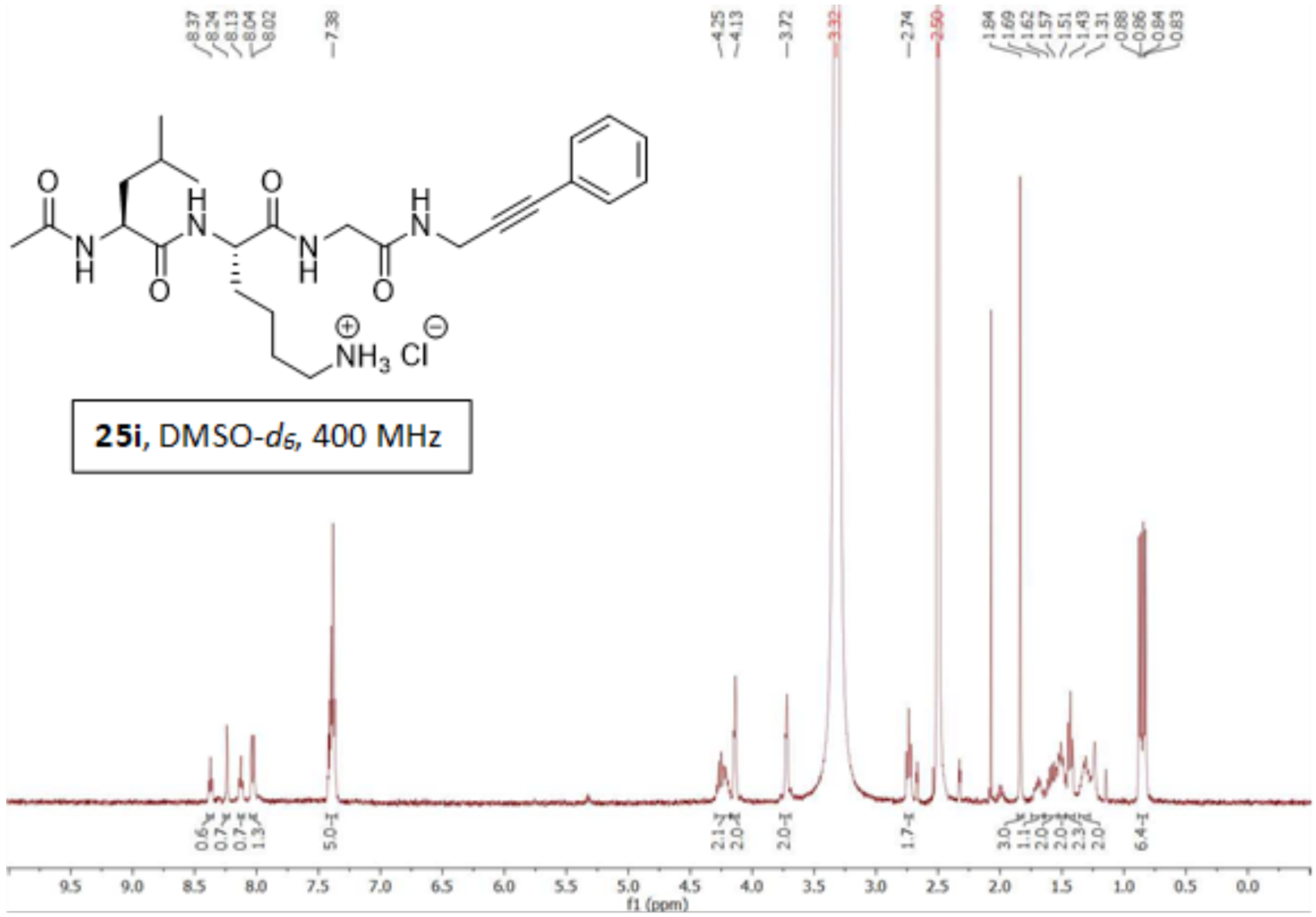

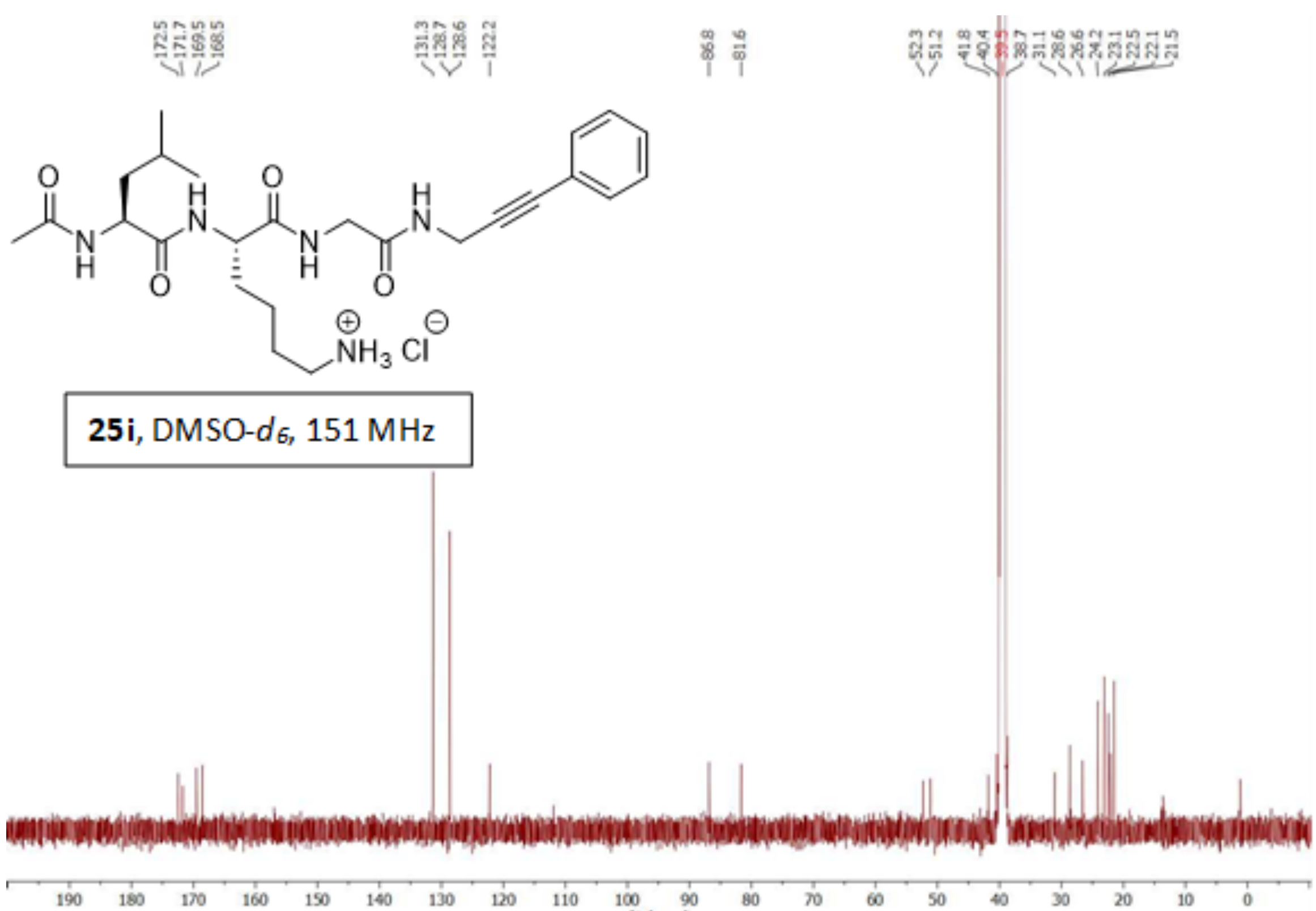

Compound **25k**

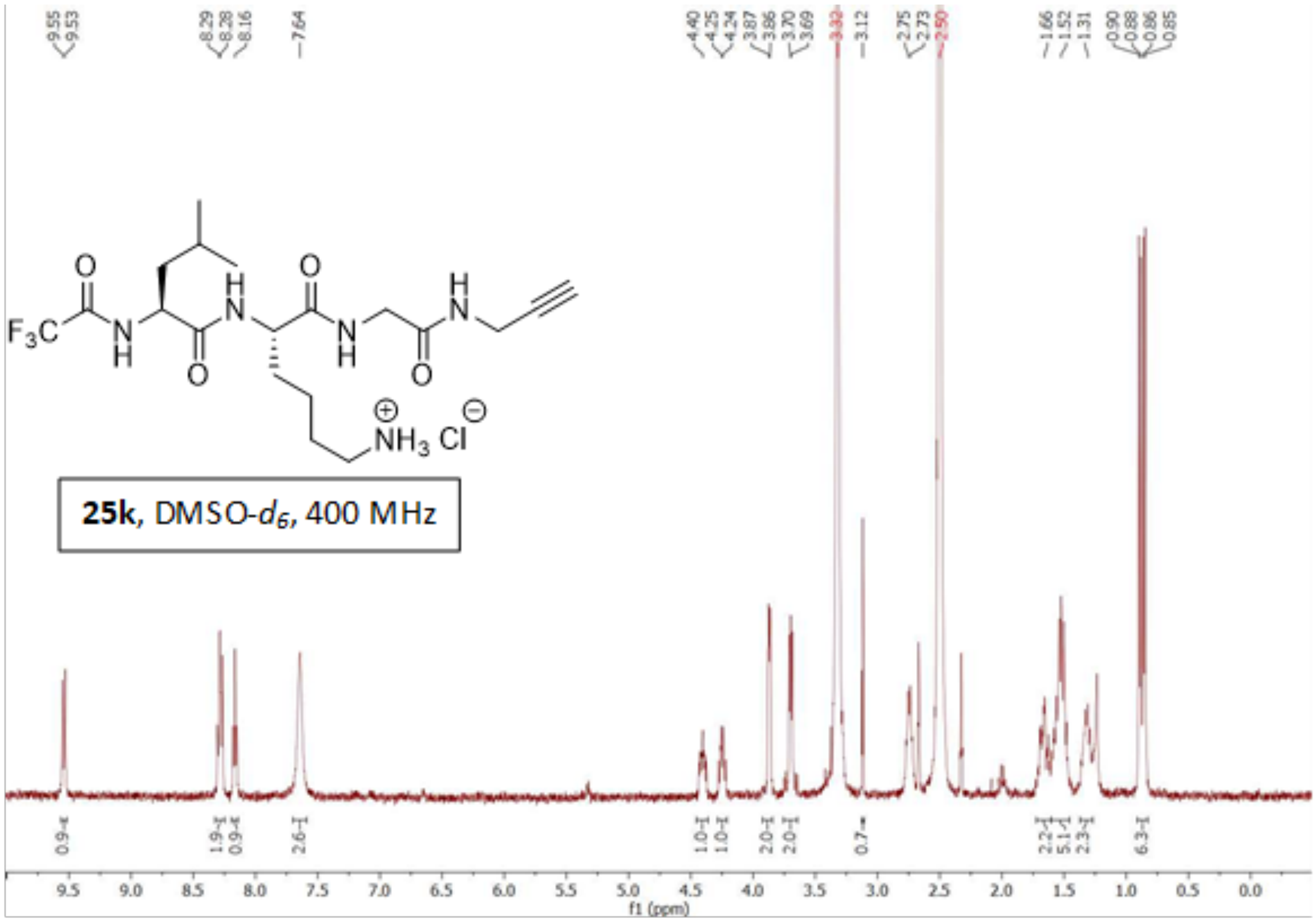

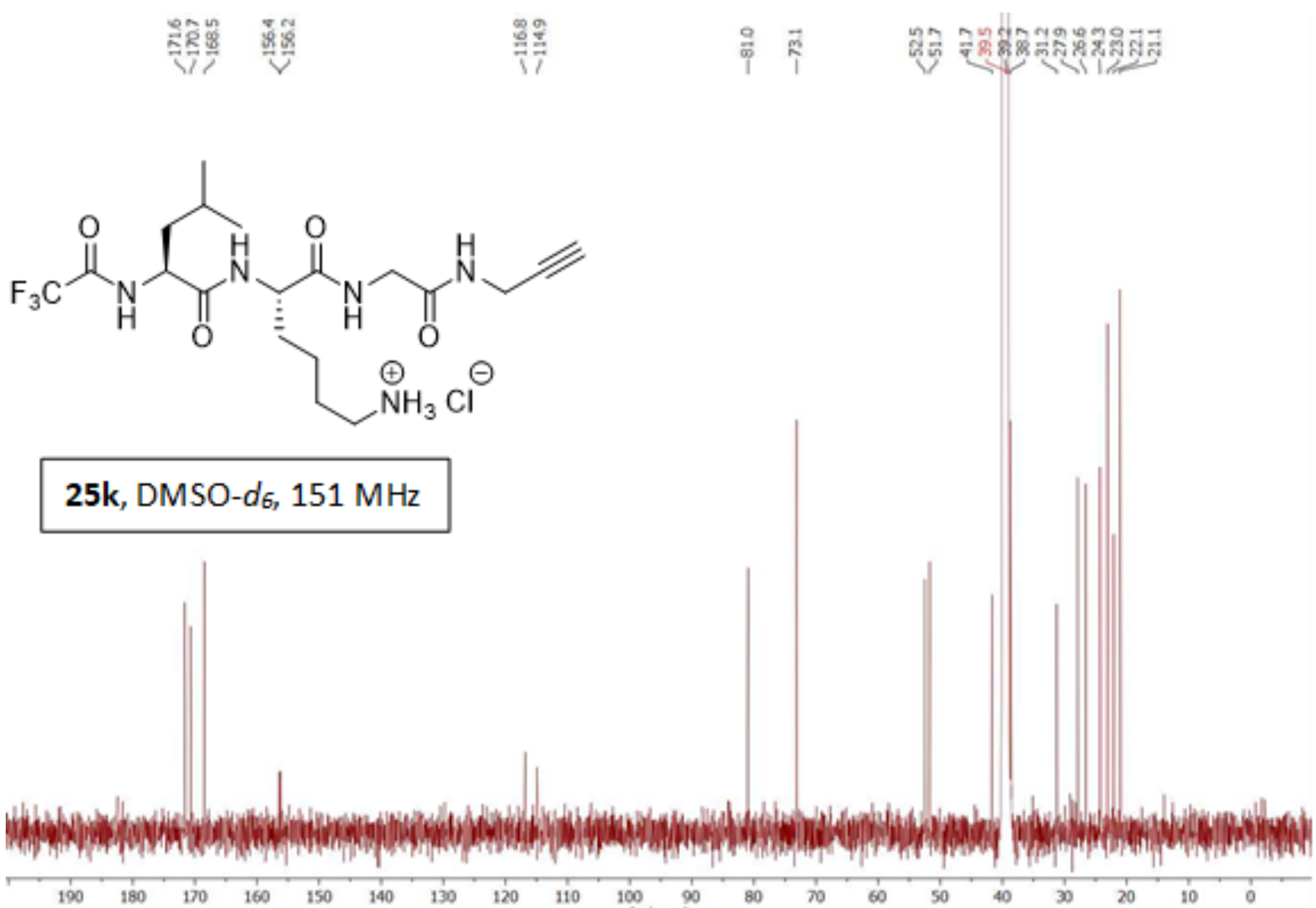

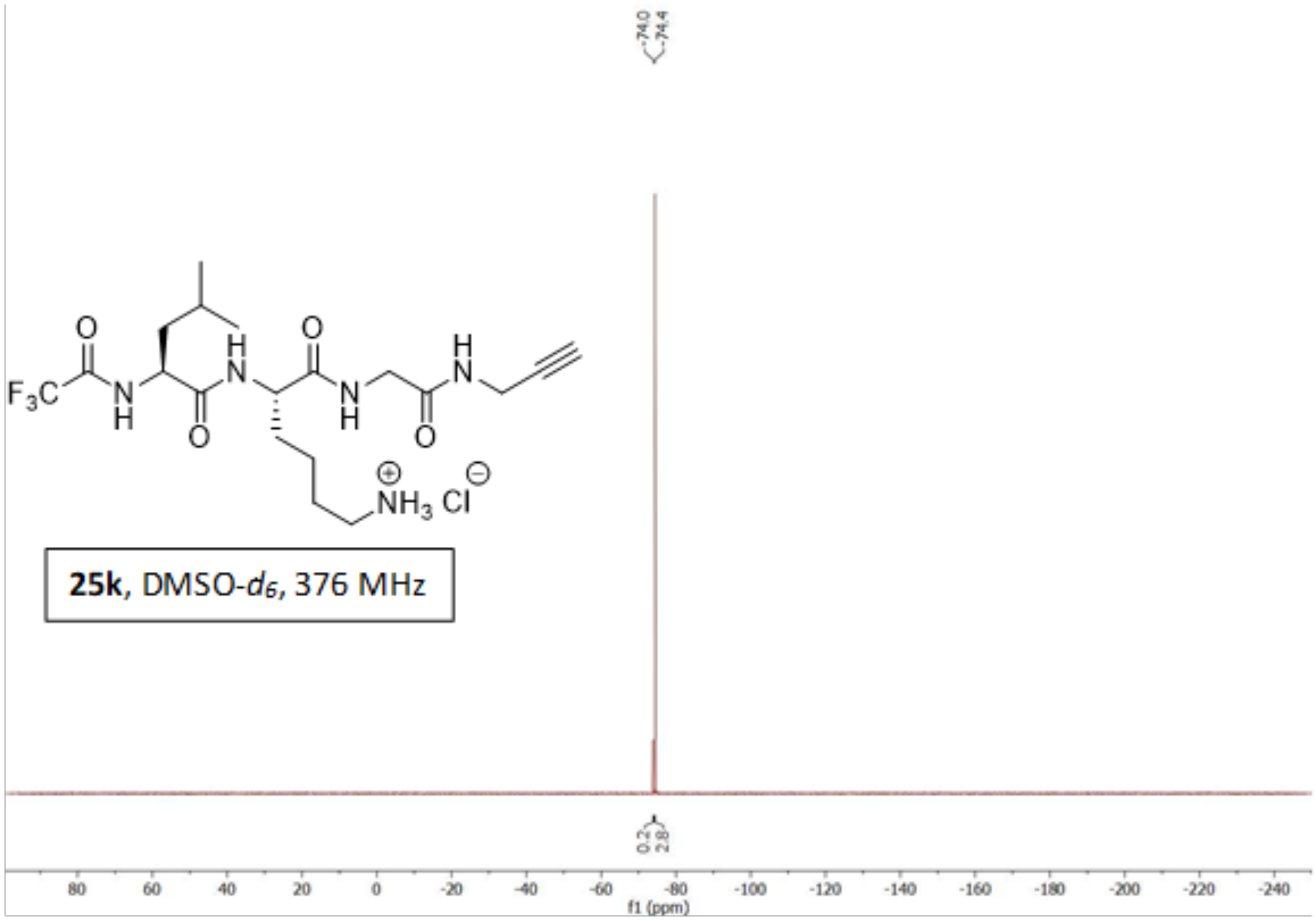

-74.0
-74.4
F3C
NH3 Cl
25k, DMSO-d6, 376 MHz
0.2
2.8
80 60 40 20 0 -20 -40 -60 -80 -100 -120 -140 -160 -180 -200 -220 -240
f1 (ppm)

Compound **25b**

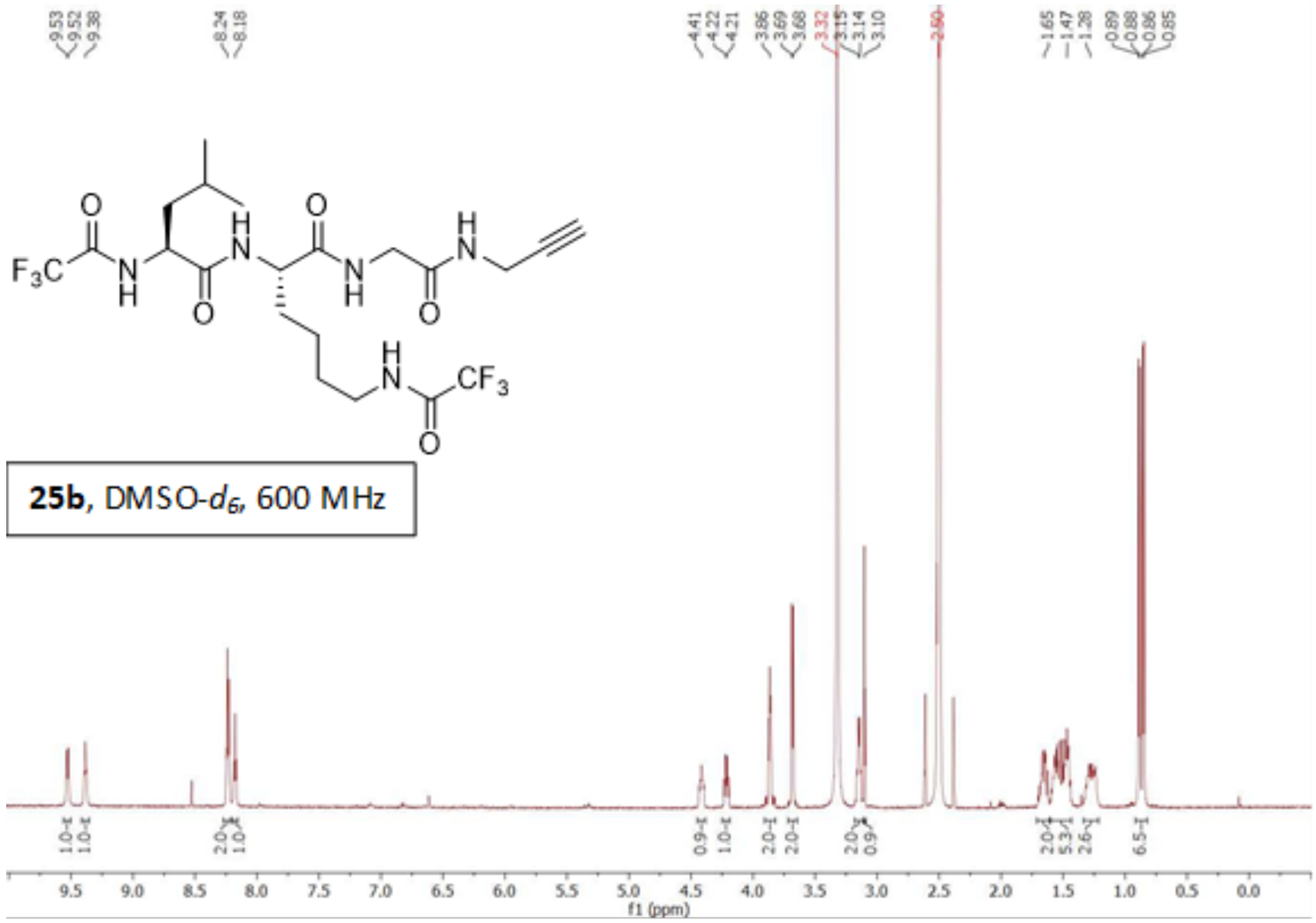

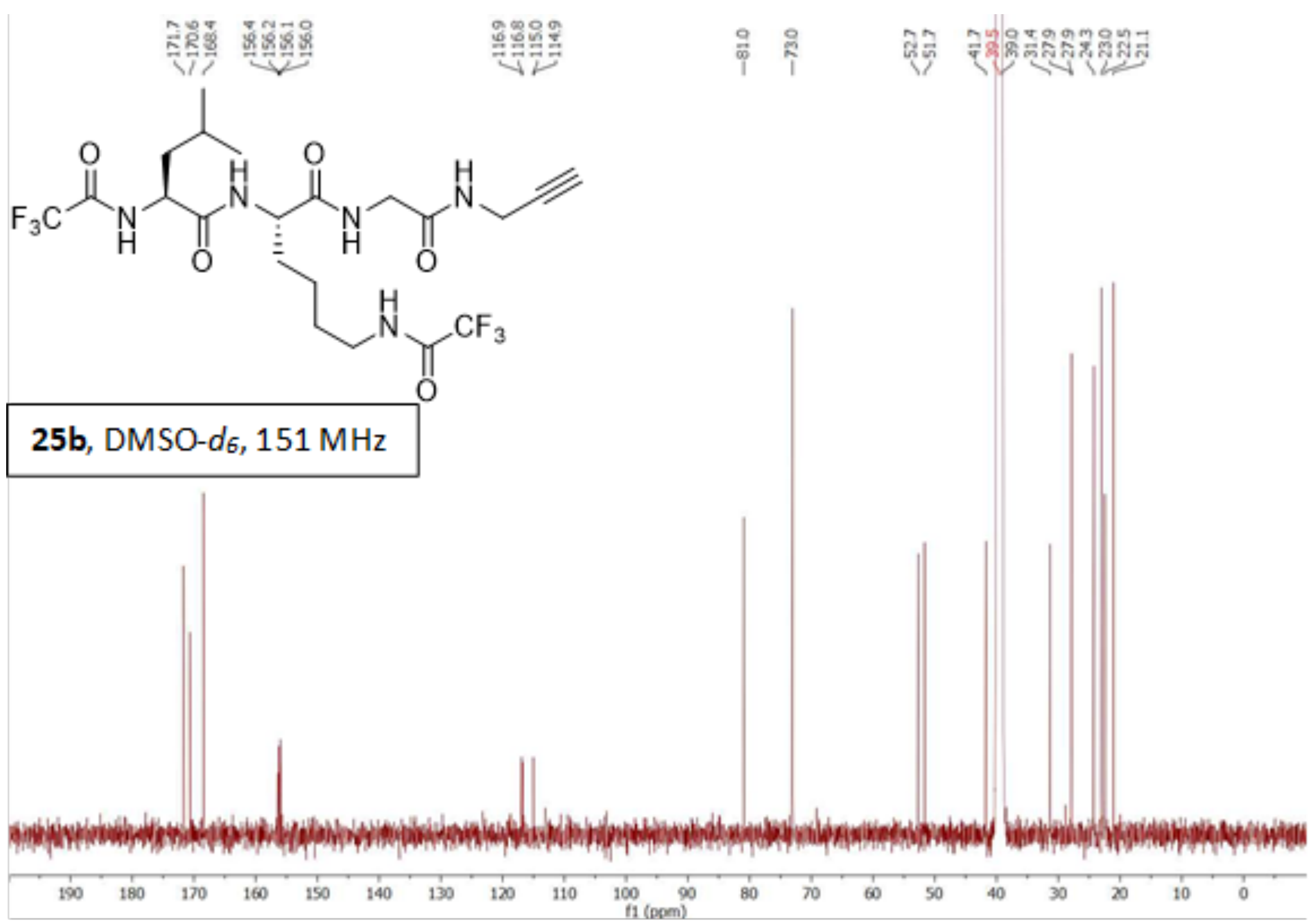

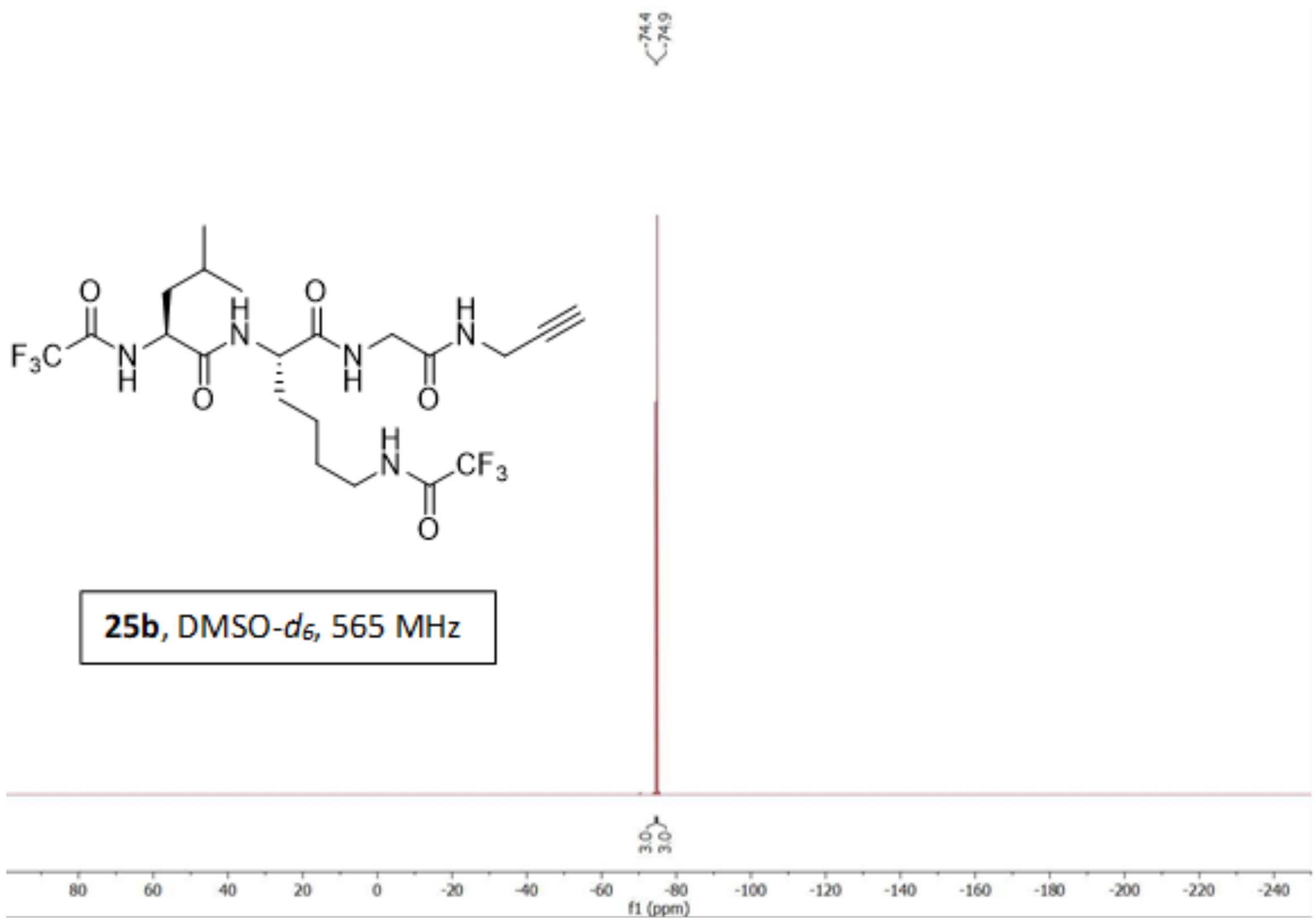
-74.4
-74.9
25b, DMSO-d6, 565 MHz
3.0
3.0
80 60 40 20 0 -20 -40 -60 -80 -100 -120 -140 -160 -180 -200 -220 -240
f1 (ppm)

Compound 31

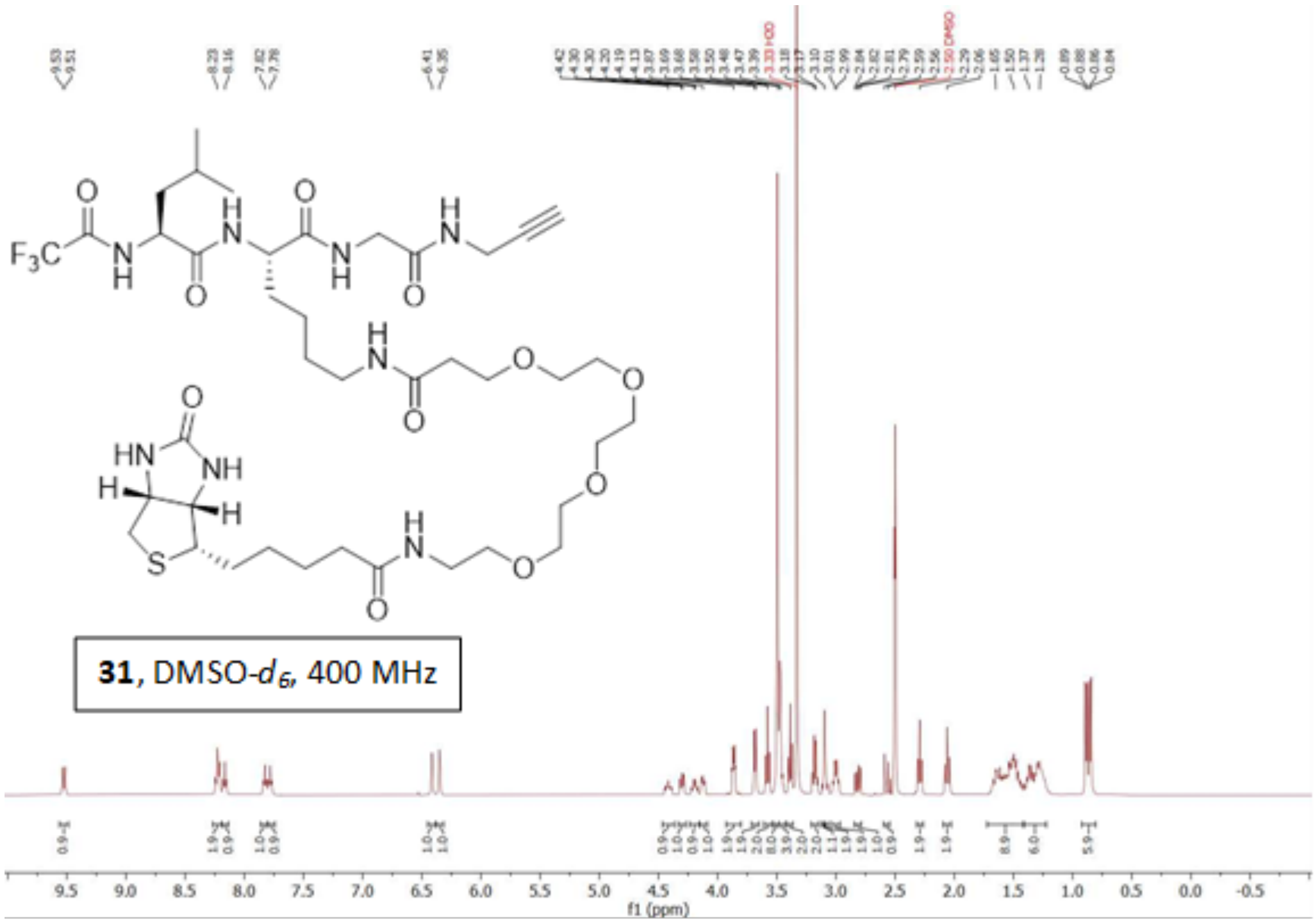

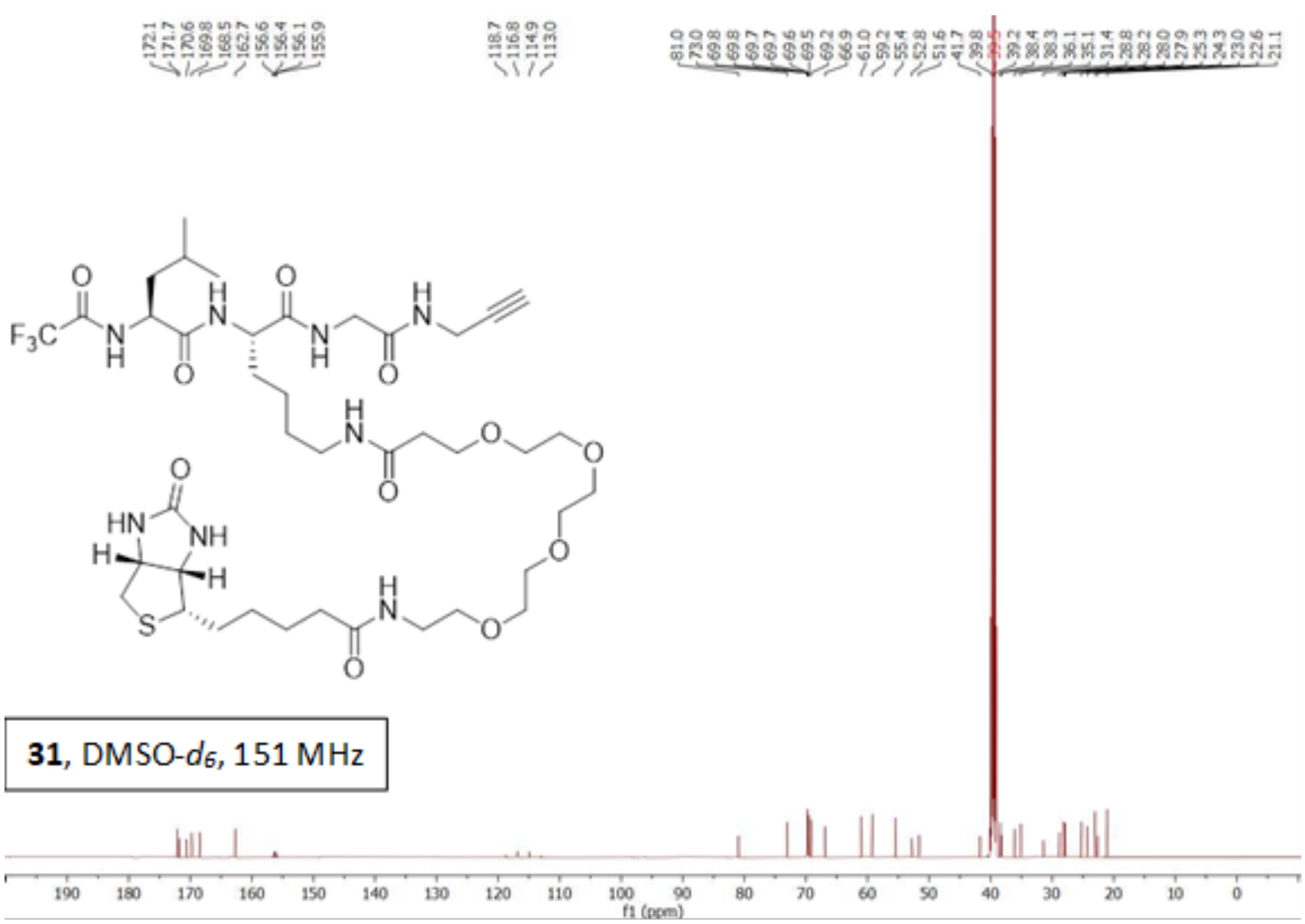

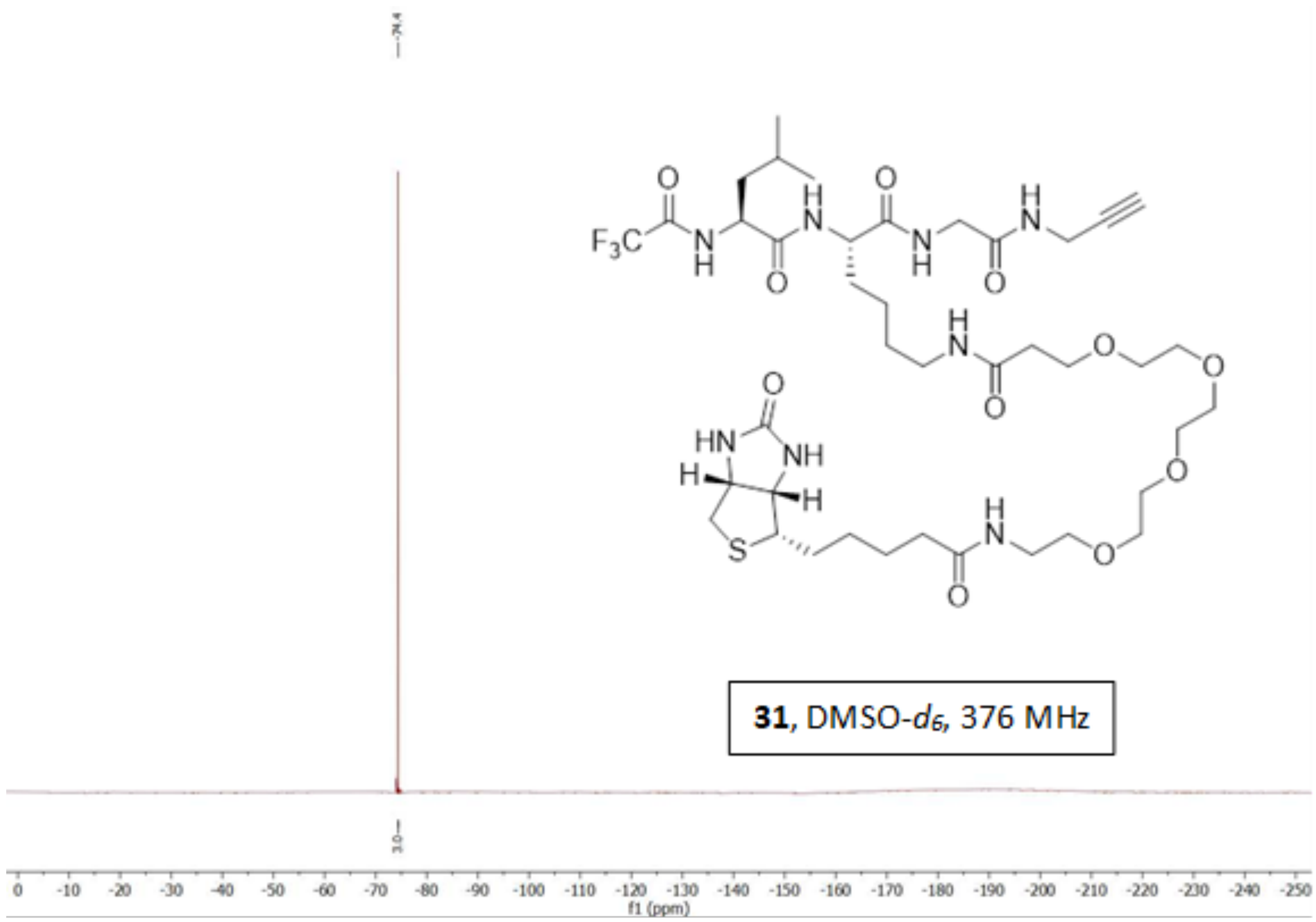
-74.4
F3C
HN
NH
H
H
S
31, DMSO-d6, 376 MHz
3.0
0 -10 -20 -30 -40 -50 -60 -70 -80 -90 -100 -110 -120 -130 -140 -150 -160 -170 -180 -190 -200 -210 -220 -230 -240 -250
f1 (ppm)

Compound 32

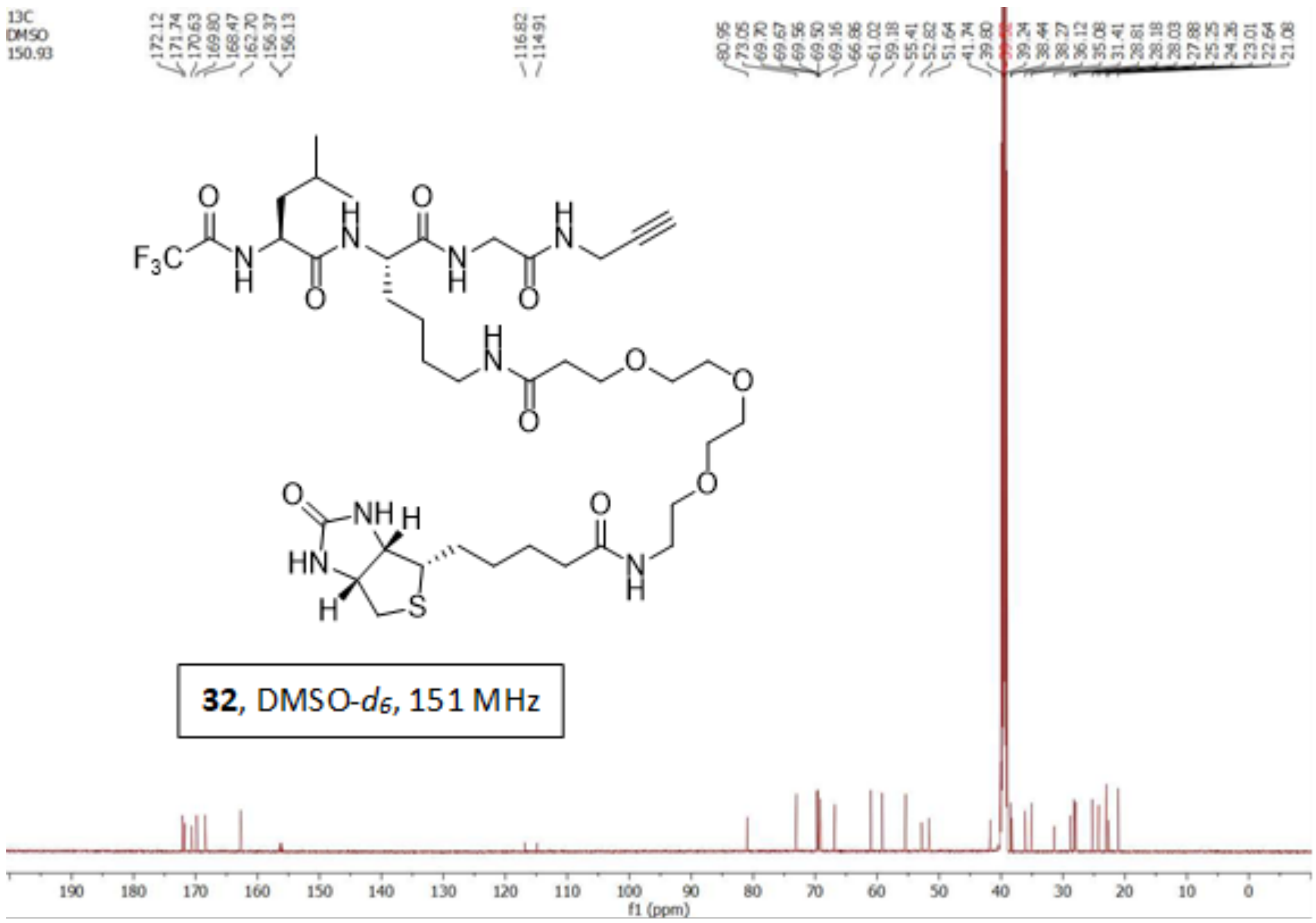

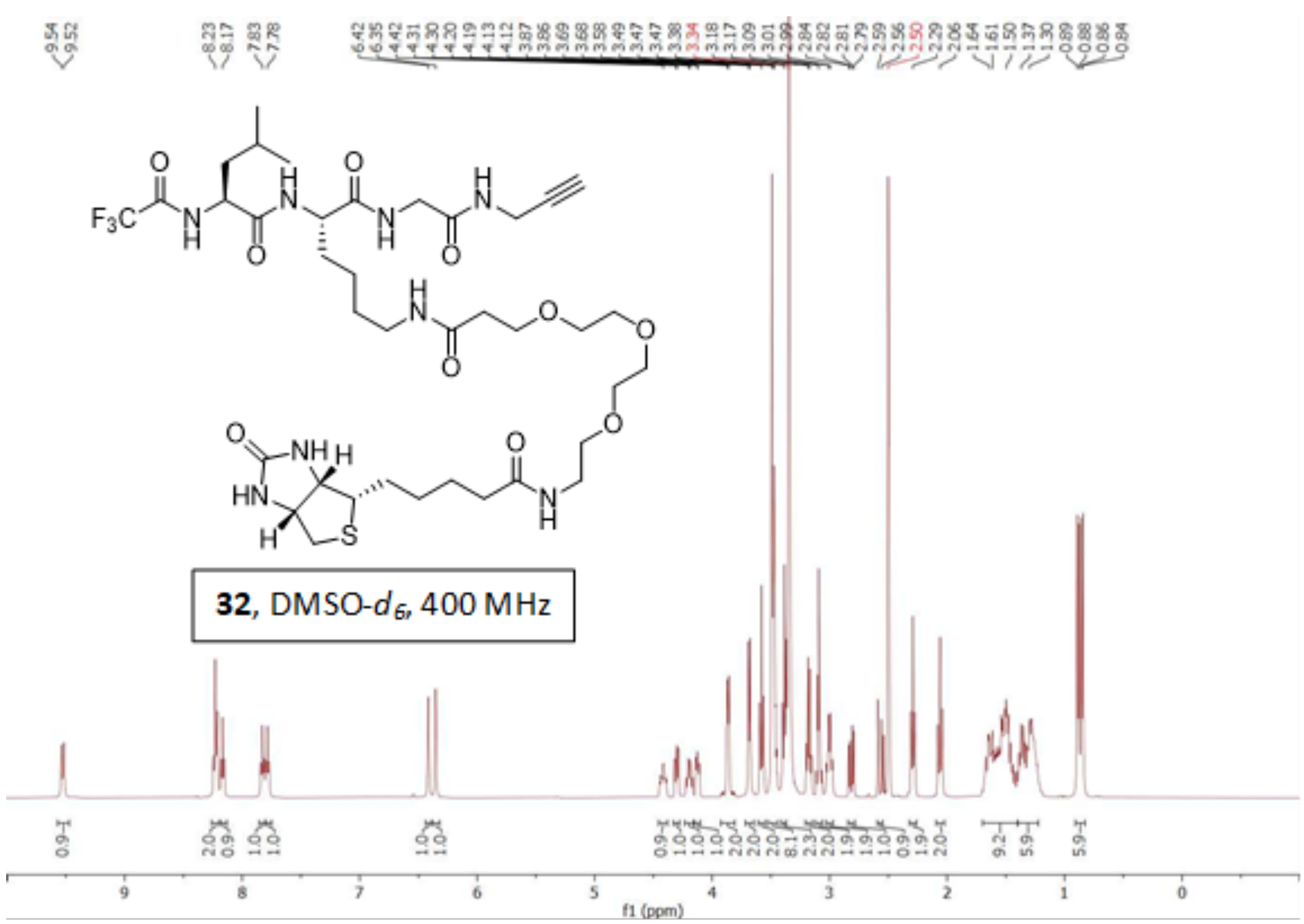

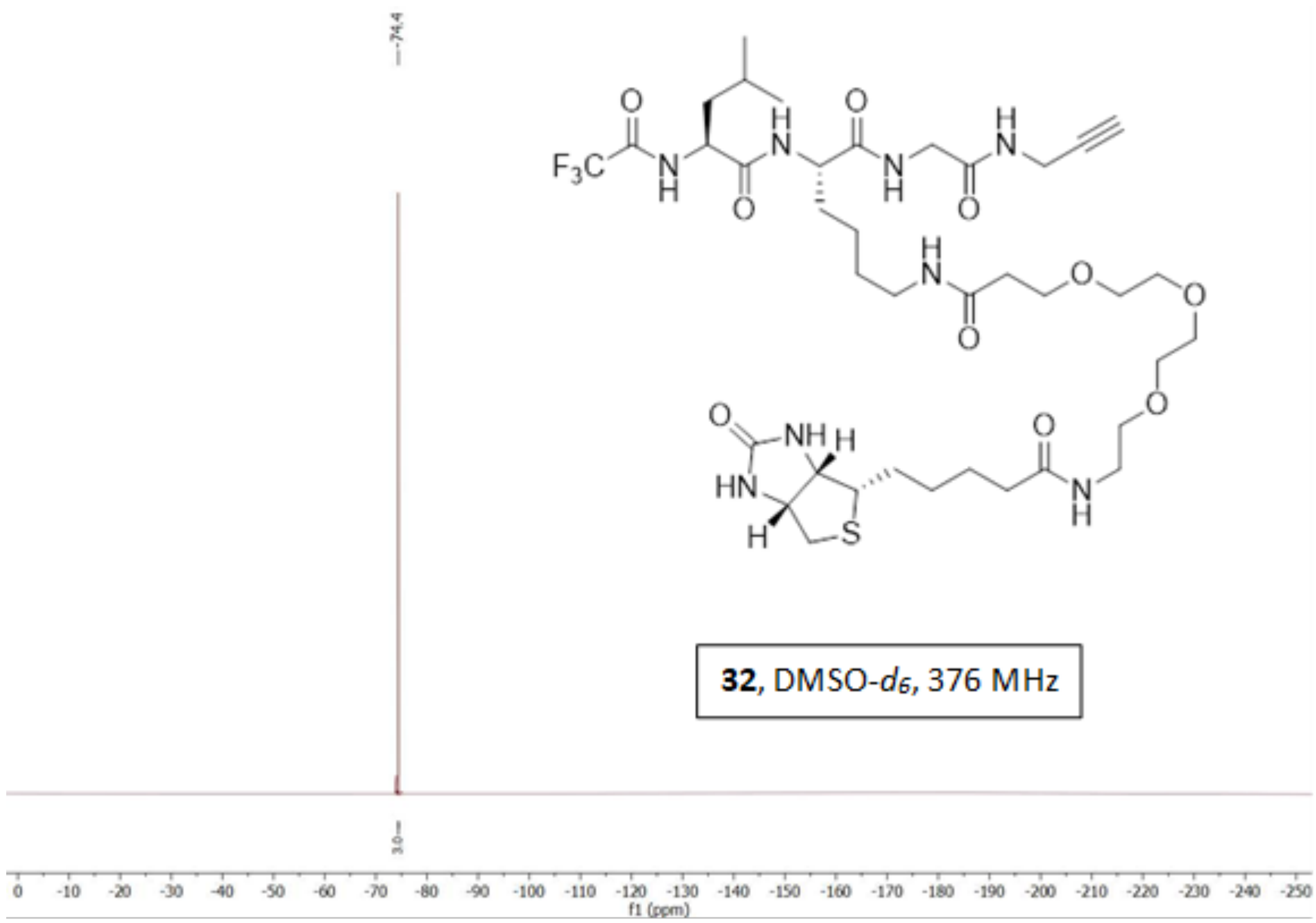

-74.4
32, DMSO-d_6, 376 MHz
3.0
f1 (ppm)

Compound 33

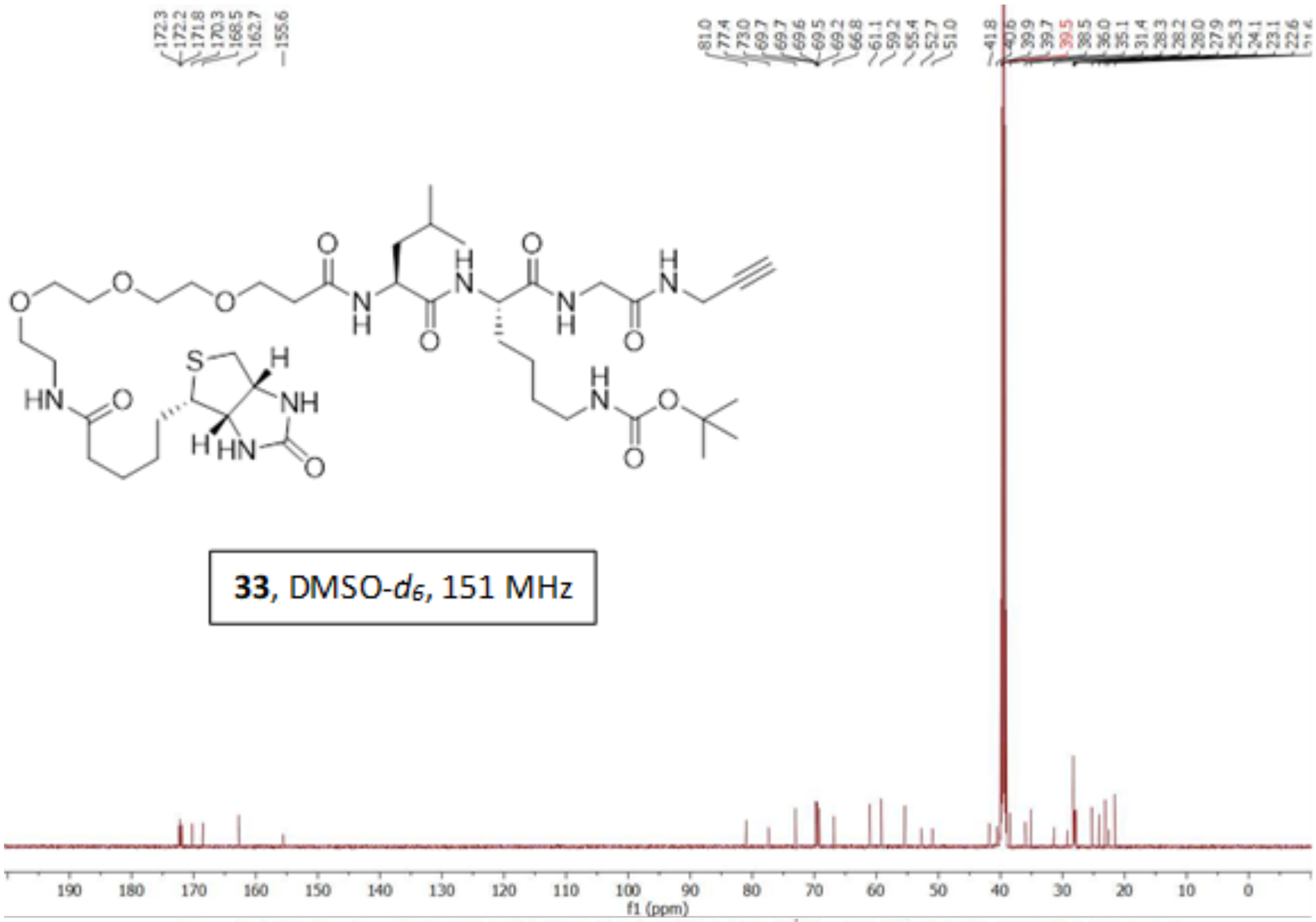

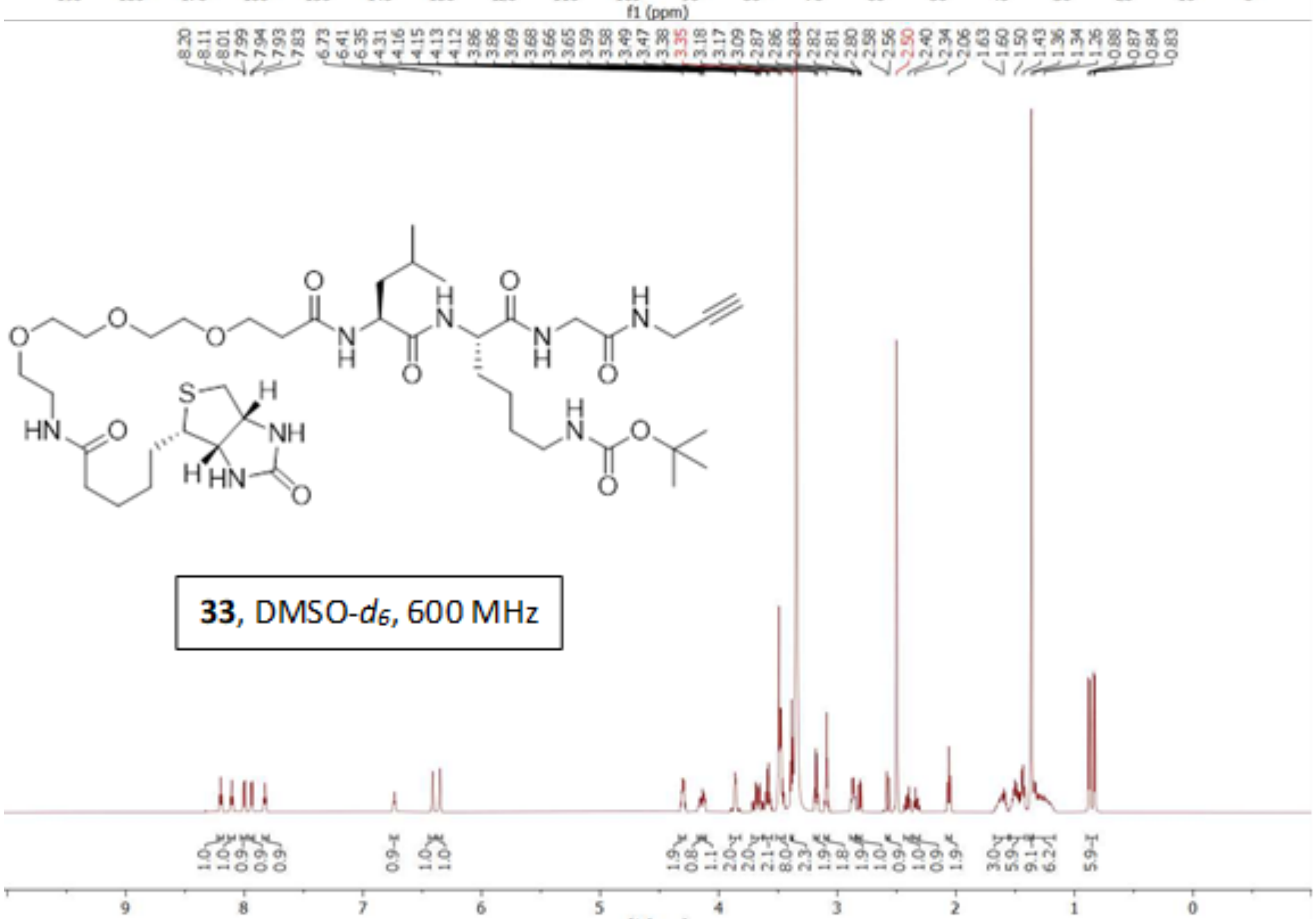

Compound 34

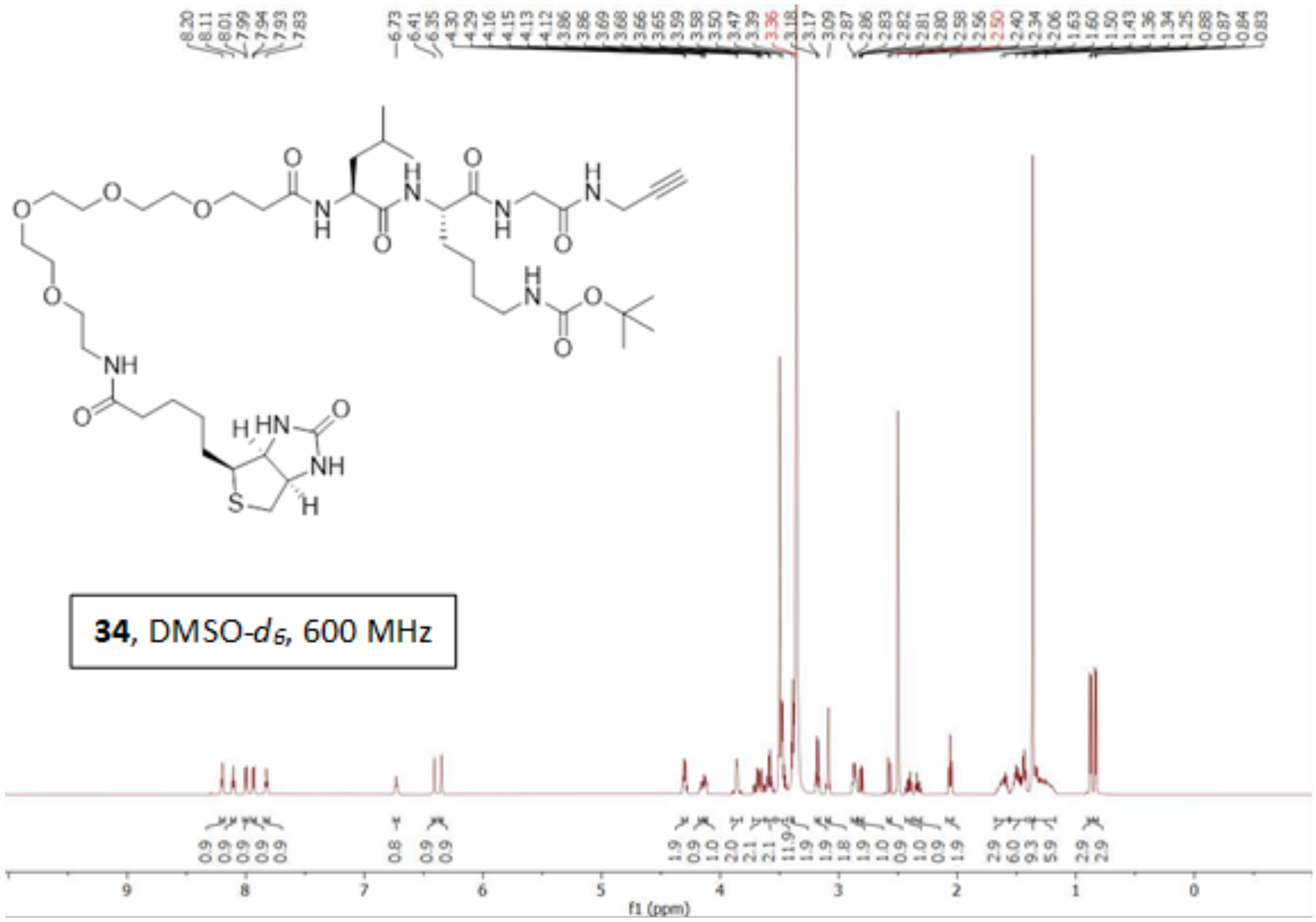

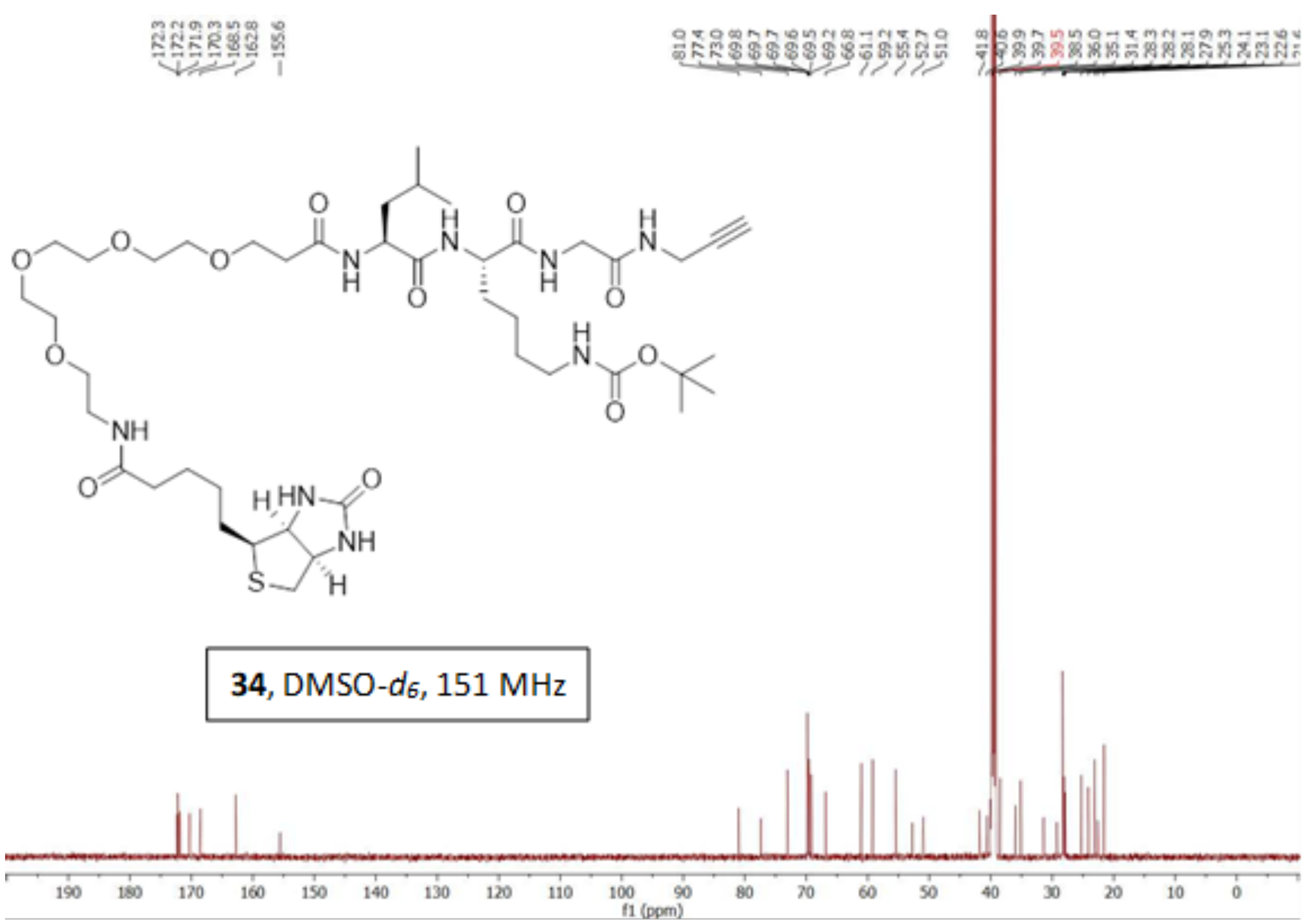

Compound 35

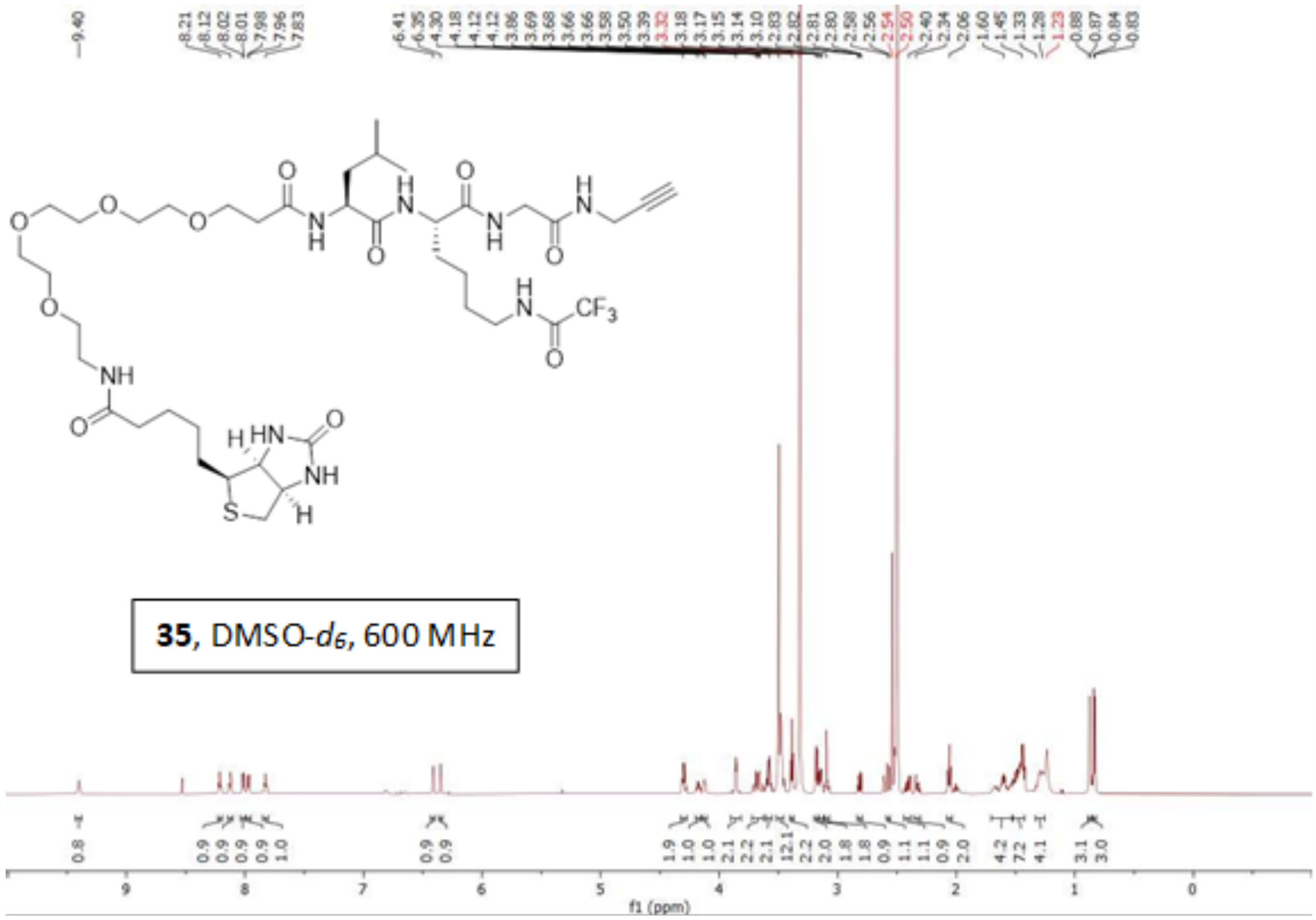

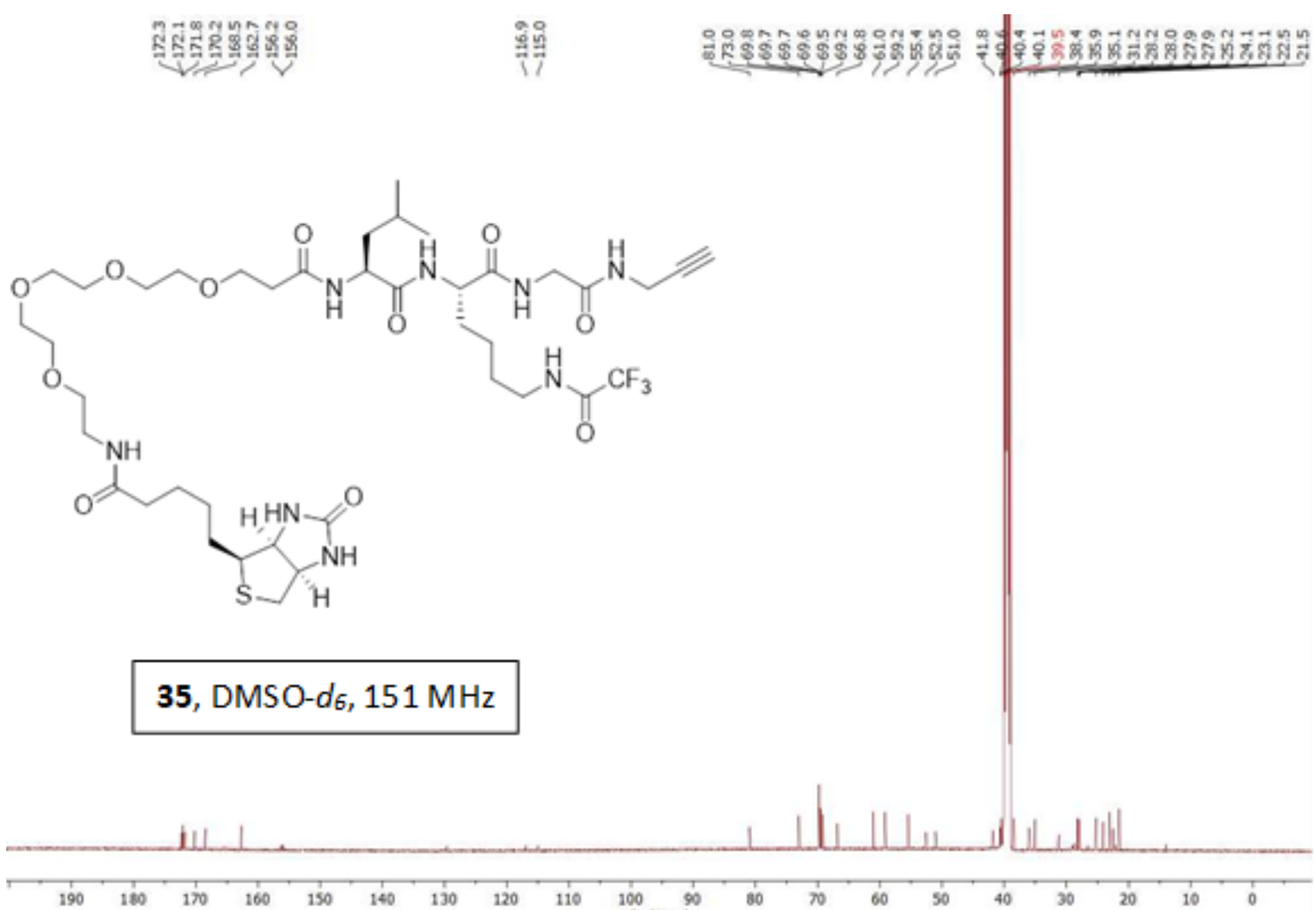

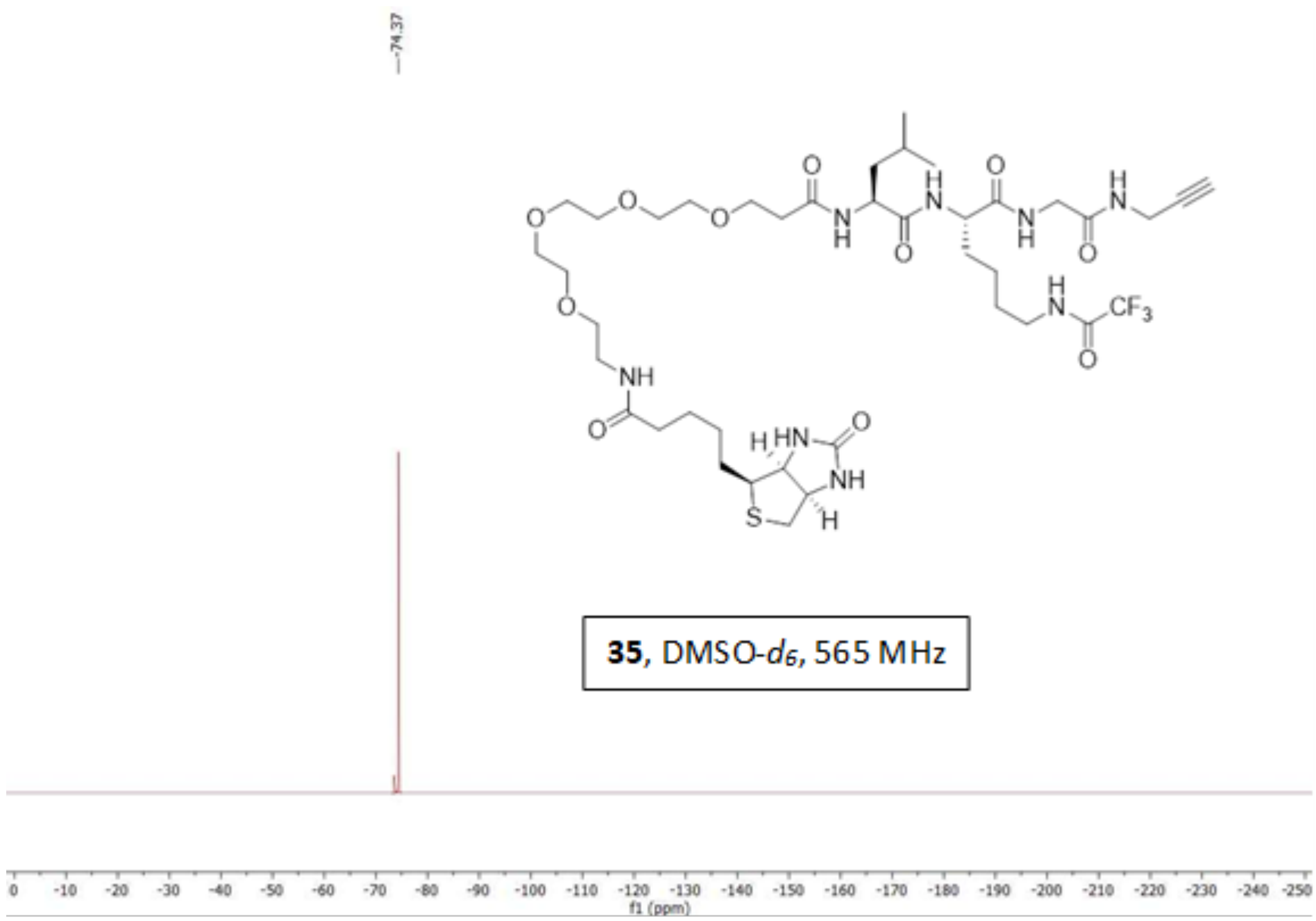
-74.37
35, DMSO-d6, 565 MHz
0 -10 -20 -30 -40 -50 -60 -70 -80 -90 -100 -110 -120 -130 -140 -150 -160 -170 -180 -190 -200 -210 -220 -230 -240 -250
f1 (ppm)

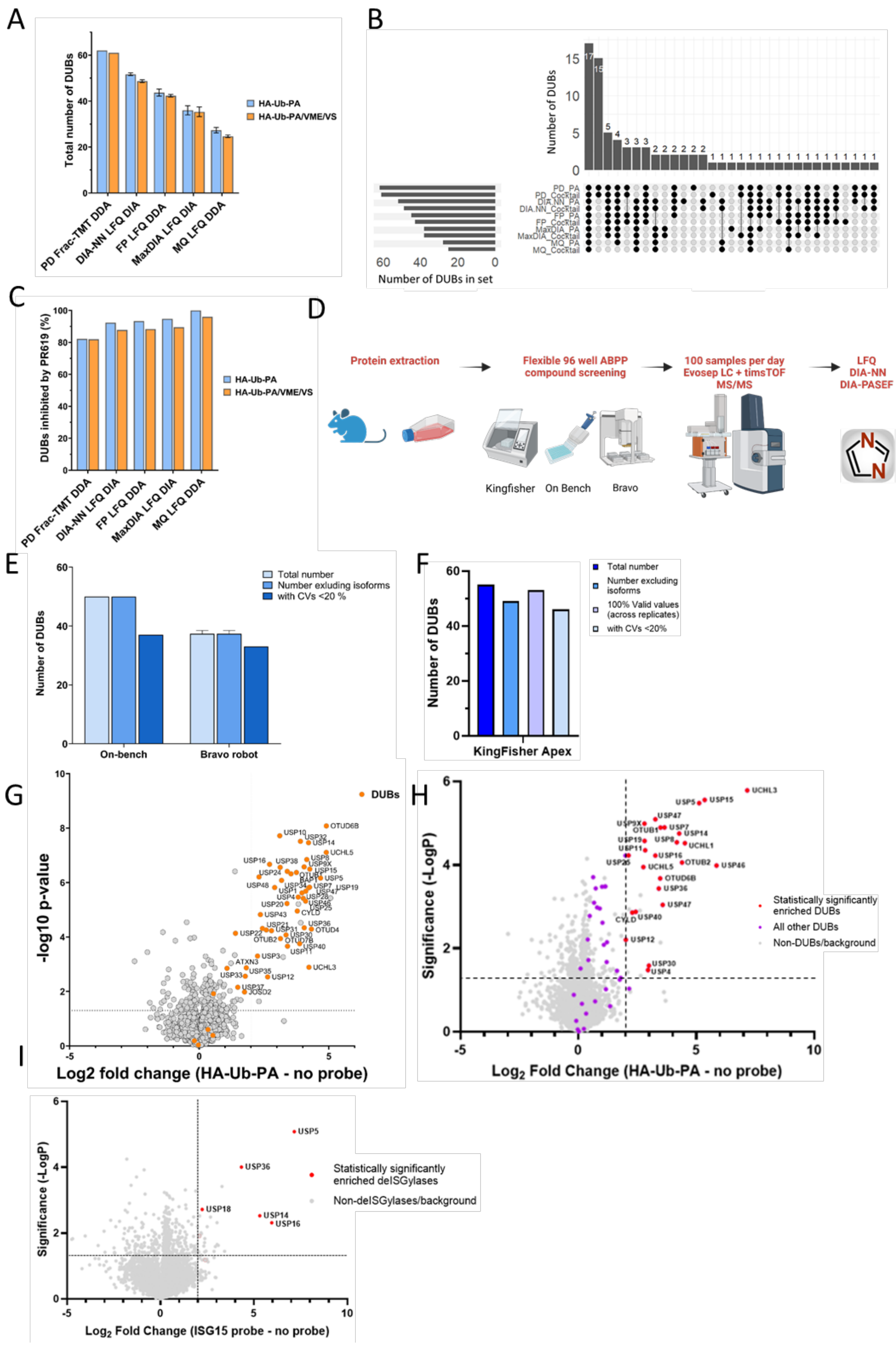
A
B
C
D
E
F
G
H
I
Total number of DUBs
HA-Ub-PA
HA-Ub-PA/VME/VS
PD Frac-TMT DDA
DIA-NN LFQ DIA
FP LFQ DDA
MaxDIA LFQ DIA
MQ LFQ DDA
Number of DUBs
Number of DUBs in set
DUBs inhibited by PR619 (%)
Protein extraction
Flexible 96 well ABPP compound screening
100 samples per day Evosep LC + timsTOF MS/MS
LFQ DIA-NN DIA-PASEF
Kingfisher
On Bench
Bravo
Total number
Number exluding isoforms
with CVs <20 %
On-bench
Bravo robot
Number excluding isoforms
100% Valid values (across replicates)
with CVs <20%
KingFisher Apex
-log10 p-value
DUBs
Log2 fold change (HA-Ub-PA - no probe)
Significance (-LogP)
Statistically significantly enriched DUBs
All other DUBs
Non-DUBs/background
Log$_2$ Fold Change (HA-Ub-PA - no probe)
Statistically significantly enriched deISGylases
Non-deISGylases/background
Log$_2$ Fold Change (ISG15 probe - no probe)

Figure S1. ABPP optimisation and flexibility. (A) Comparison of the total number of DUBs, including isoforms, identified in MCF-7 lysates by ABPP from in-depth ABPP workflows with either HA-Ub-PA ABP or a cocktail of ABPs (HA-Ub-PA/VME/VS). DDA = Data-dependent acquisition. DIA = Data independent acquisition. Frac-TMT = Fractionated ABPP samples labelled with tandem mass tags (TMT). LFQ = Label free quantitation. PD = proteome discoverer. FP = Fragpipe. MQ = Maxquant. (B) Upset plot showing the commonality of DUBs identified from MCF-7 cell lysates in (A) by each high DUBome depth workflows. (C) Percentage of DUBs inhibited by >50% by PR619 from each by each high DUBome depth workflows shown in (A). (D) HA-Ub-PA high-throughput workflows. Created in BioRender. Jones, H. (2025) (License link to be inserted upon publication). DIA-PASEF = data-independent acquisition-Parallel Accumulation–Serial Fragmentation. (E) The number of cysteine-active DUBs identified from MCF-7 cell lysates by ABPP in total using the high-throughput workflows outlined in (D), with on-bench or Bravo robot 96-well immunoprecipitation (error bars = standard deviation (SD)). (F) The number of DUBs identified from HAP1 lysates using the ABPP-HT* high-throughput workflow with KingFisher Apex 96 well automated affinity purification. (G) Volcano plot of the enrichment of DUBs identified with HA-Ub-PA ABPP-HT* enrichment by the on-bench immunoprecipitation. (H) Volcano plot of the enrichment of DUBs from HAP1 lysates (IFN-treated) identified with HA-Ub-PA ABPP-HT* enrichment by KingFisher Apex 96 well automated affinity purification. (I) Volcano plot of the enrichment of deISGylases from HAP1 lysates (IFN-treated) identified with bio-ISG15-PA ABPP-HT* enrichment by KingFisher Apex 96 well automated affinity purification.

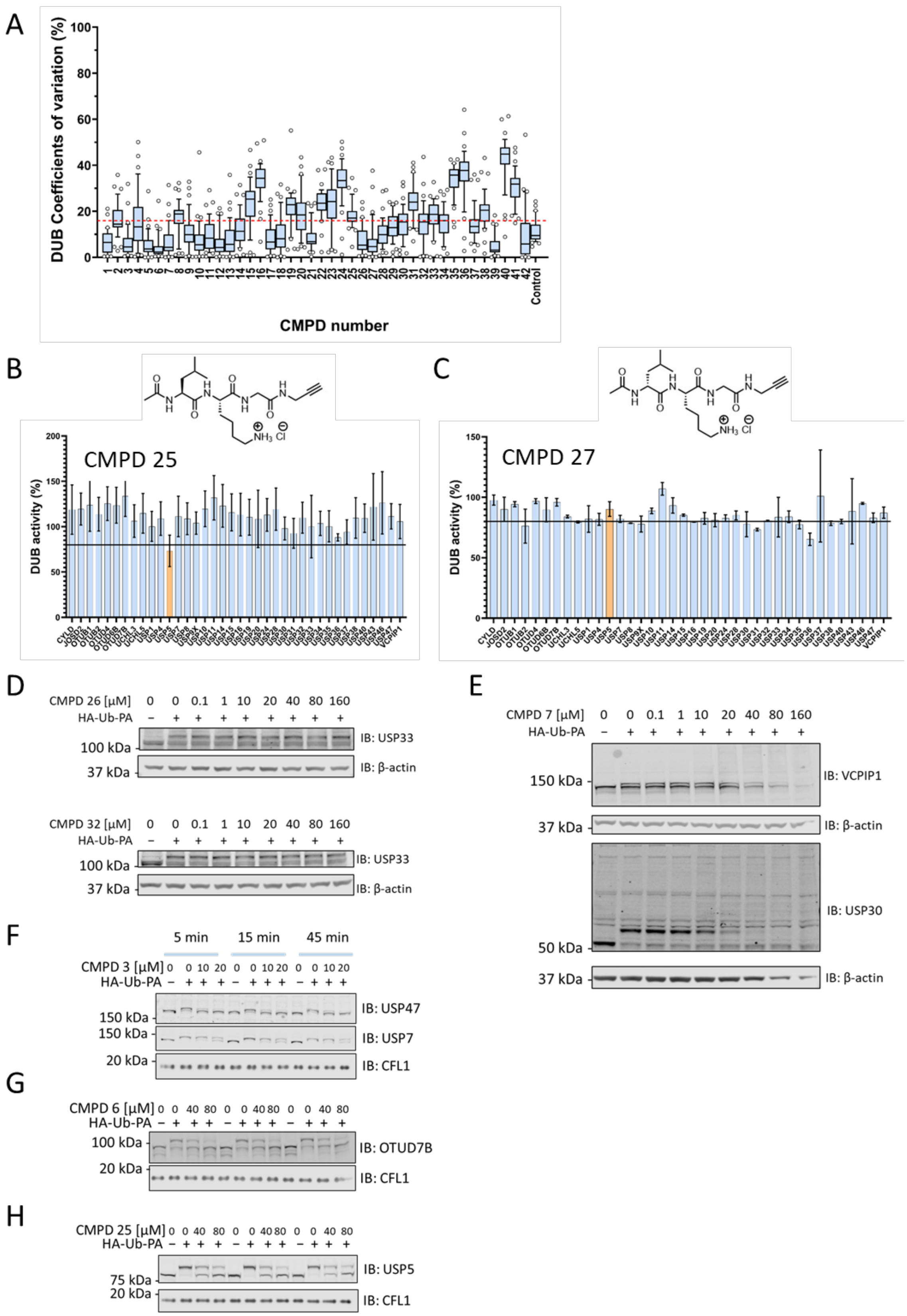
A
DUB Coefficients of variation (%)
CMPD number
Control
B
CMPD 25
DUB activity (%)
C
CMPD 27
DUB activity (%)
D
CMPD 26 [μM] 0 0 0.1 1 10 20 40 80 160
HA-Ub-PA – + + + + + + + +
100 kDa
IB: USP33
37 kDa
IB: β-actin
CMPD 32 [μM] 0 0 0.1 1 10 20 40 80 160
HA-Ub-PA – + + + + + + + +
100 kDa
IB: USP33
37 kDa
IB: β-actin
E
CMPD 7 [μM] 0 0 0.1 1 10 20 40 80 160
HA-Ub-PA – + + + + + + + +
150 kDa
IB: VCPIP1
37 kDa
IB: β-actin
IB: USP30
50 kDa
37 kDa
IB: β-actin
F
5 min 15 min 45 min
CMPD 3 [μM] 0 0 10 20 0 0 10 20 0 0 10 20
HA-Ub-PA – + + + – + + + – + + +
150 kDa
IB: USP47
150 kDa
IB: USP7
20 kDa
IB: CFL1
G
CMPD 6 [μM] 0 0 40 80 0 0 40 80 0 0 40 80
HA-Ub-PA – + + + – + + + – + + +
100 kDa
IB: OTUD7B
20 kDa
IB: CFL1
H
CMPD 25 [μM] 0 0 40 80 0 0 40 80 0 0 40 80
HA-Ub-PA – + + + – + + + – + + +
75 kDa
IB: USP5
20 kDa
IB: CFL1

Fig. S2. Hit validation and dose-response analyses. (A) Coefficient of variation (CV) for DUBs identified across the ABPP-HT* screen for each of the tested compounds. Box and whiskers are at 10-90 percentile. Red dashed line: average CV for DUBs across all conditions. (B-C) HA-Ub-PA ABPP of **Compounds 25** and **27** at 25 µM in MCF-7 cell lysates (error bars = SD). (D) Concentration-dependent binding of **Compounds 26** and **32** to USP33 in MCF-7 cell lysates. Data was obtained using with HA-Ub-PA as ABP. (E) Concentration-dependent for VCPIP1 in MCF-7 lysates and USP30 in SH-SY5Y lysates by **Compound 7** with HA-Ub-PA labeling. The observation of high-molecular weight smearing and reduced intensity of DUBs suggests that **Compound 7** might mediate DUB cross-linking. (F-H) Time-dependent incubation of MCF-7 lysates at the indicated compound concentrations using HA-Ub-PA labeling.

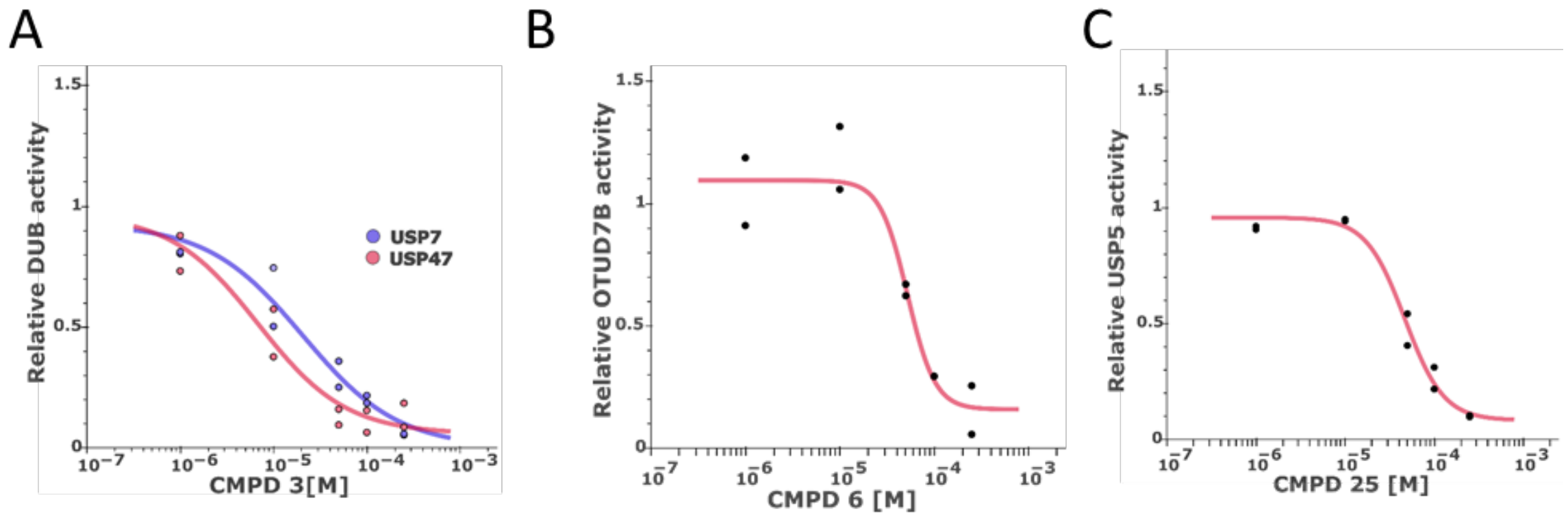

D

DUB	CMPD #	IC_{50} [µM]	R^2
USP7	3	20.7	0.85
USP47	3	6.85	0.85
OTUD7B	6	52.24	0.819
USP5	25	46.88	0.96

Fig S3. DUB inhibitor potency assessment by ABPP-HT*. (A-C) Concentration-dependent compound binding to DUBs identified using ABPP-HT* with HA-Ub-PA. Data were analysed using curve curator (8). (D) IC_{50} values obtained using curve curator from the curves shown in A-C.

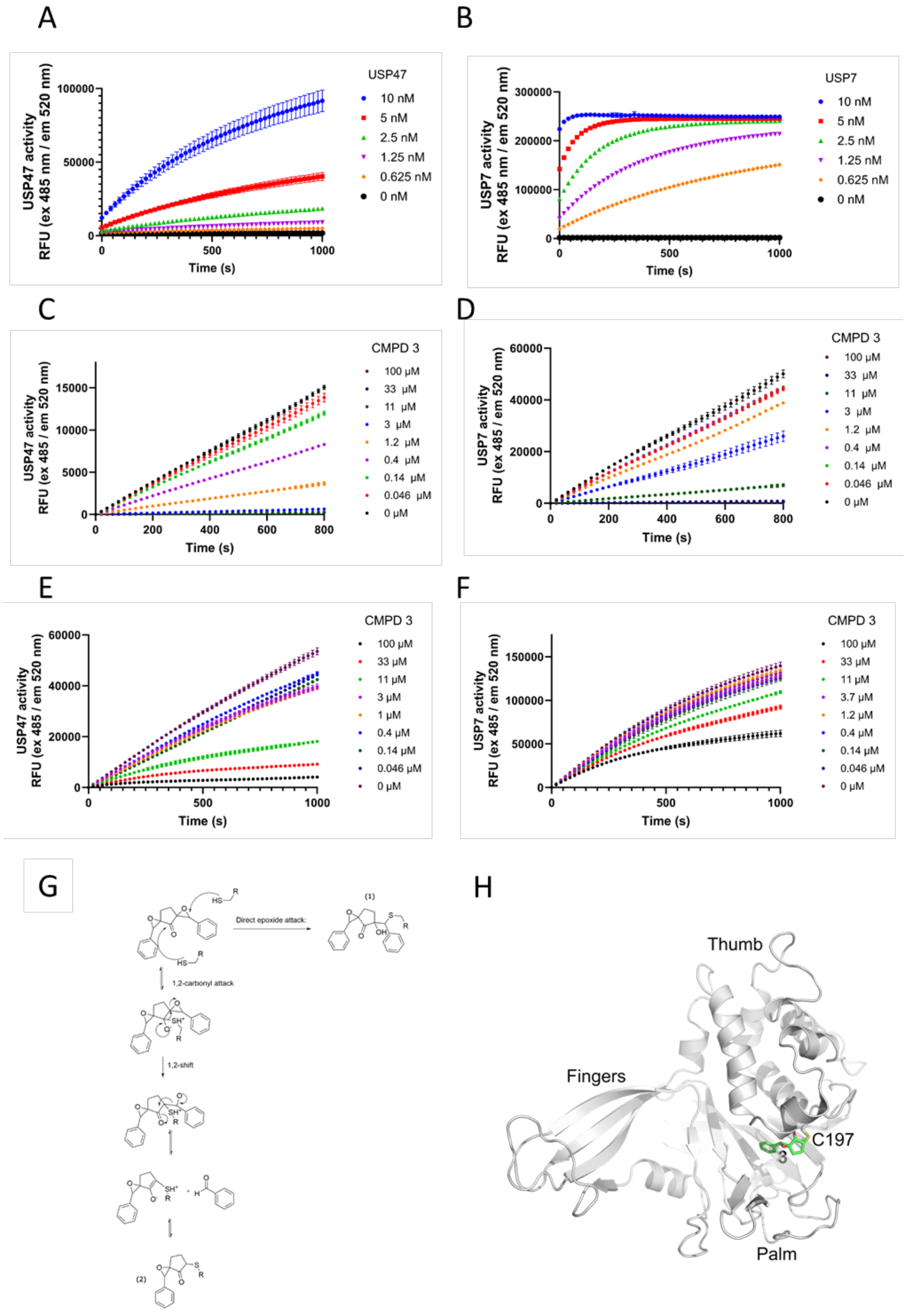
A
USP47
10 nM
5 nM
2.5 nM
1.25 nM
0.625 nM
0 nM
USP47 activity
RFU (ex 485 nm / em 520 nm)
Time (s)
B
USP7
10 nM
5 nM
2.5 nM
1.25 nM
0.625 nM
0 nM
USP7 activity
RFU (ex 485 nm / em 520 nm)
Time (s)
C
CMPD 3
100 μM
33 μM
11 μM
3 μM
1.2 μM
0.4 μM
0.14 μM
0.046 μM
0 μM
USP47 activity
RFU (ex 485 / em 520 nm)
Time (s)
D
CMPD 3
100 μM
33 μM
11 μM
3 μM
1.2 μM
0.4 μM
0.14 μM
0.046 μM
0 μM
USP7 activity
RFU (ex 485 / em 520 nm)
Time (s)
E
CMPD 3
100 μM
33 μM
11 μM
3 μM
1 μM
0.4 μM
0.14 μM
0.046 μM
0 μM
USP47 activity
RFU (ex 485 / em 520 nm)
Time (s)
F
CMPD 3
100 μM
33 μM
11 μM
3.7 μM
1.2 μM
0.4 μM
0.14 μM
0.046 μM
0 μM
USP7 activity
RFU (ex 485 / em 520 nm)
Time (s)
G
Direct epoxide attack:
(1)
1,2-carbonyl attack
1,2-shift
(2)
H
Thumb
Fingers
C197
3
Palm

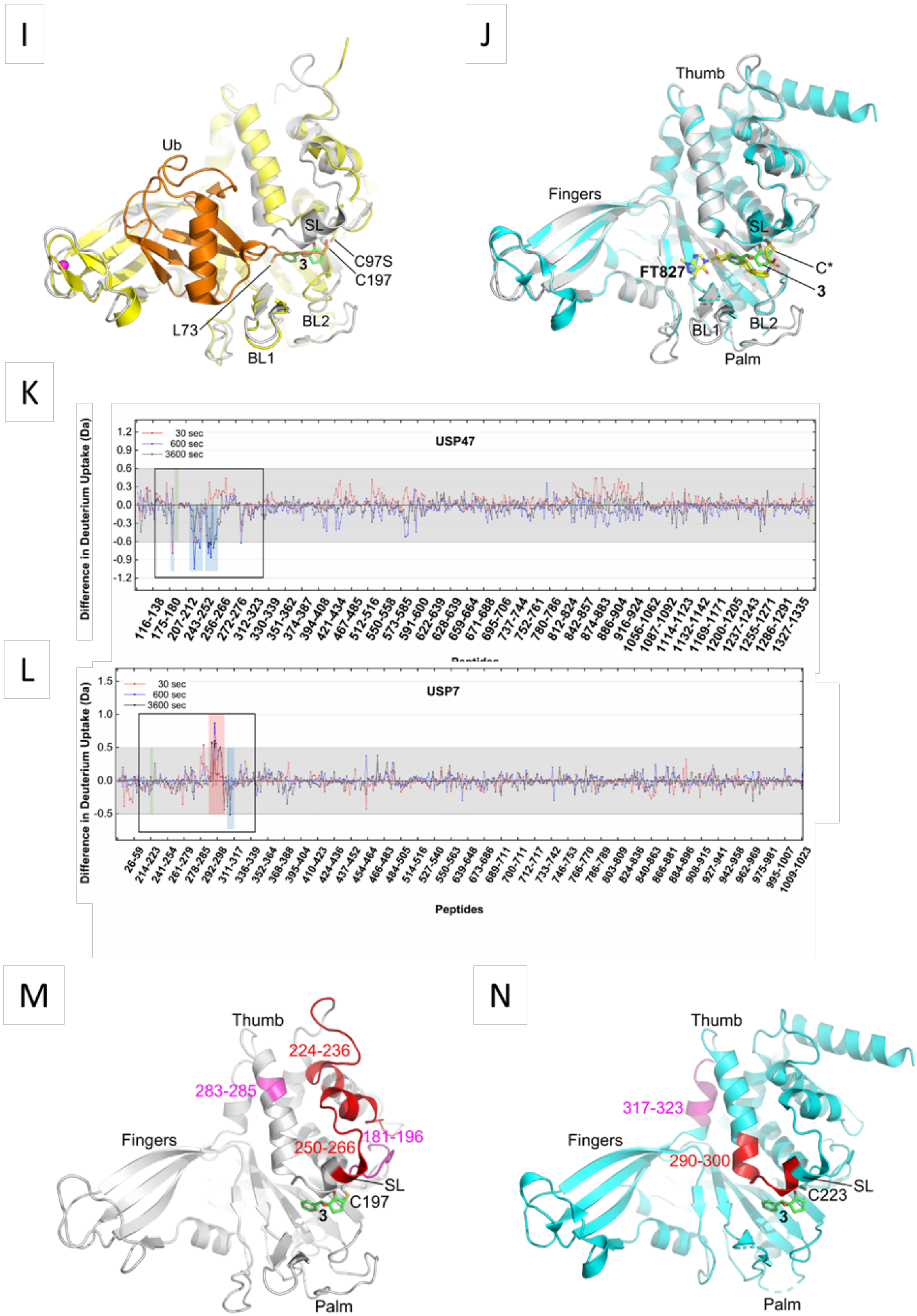
I
Ub
SL
C97S
3
C197
L73
BL2
BL1
J
Thumb
Fingers
SL
FT827
C*
3
BL1
BL2
Palm
K
USP47
30 sec
600 sec
3600 sec
Difference in Deuterium Uptake (Da)
L
USP7
30 sec
600 sec
3600 sec
Difference in Deuterium Uptake (Da)
Peptides
M
Thumb
224-236
283-285
Fingers
250-266
181-196
SL
C197
3
Palm
N
Thumb
317-323
Fingers
290-300
SL
C223
3
Palm

O

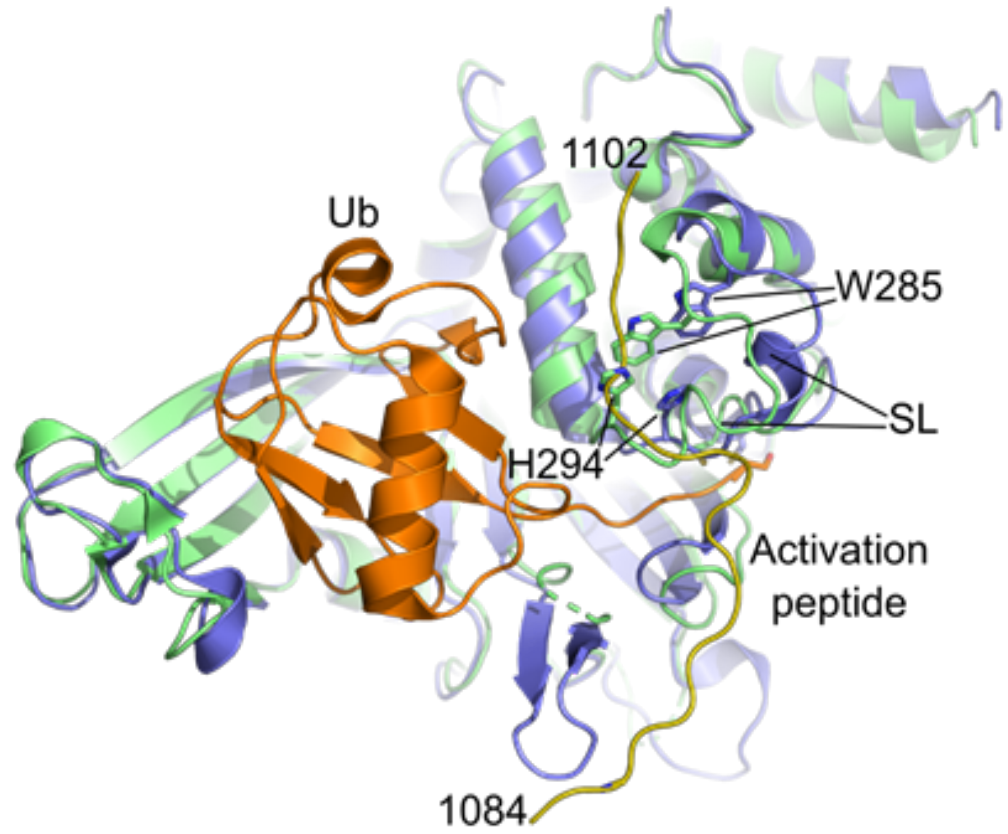

P

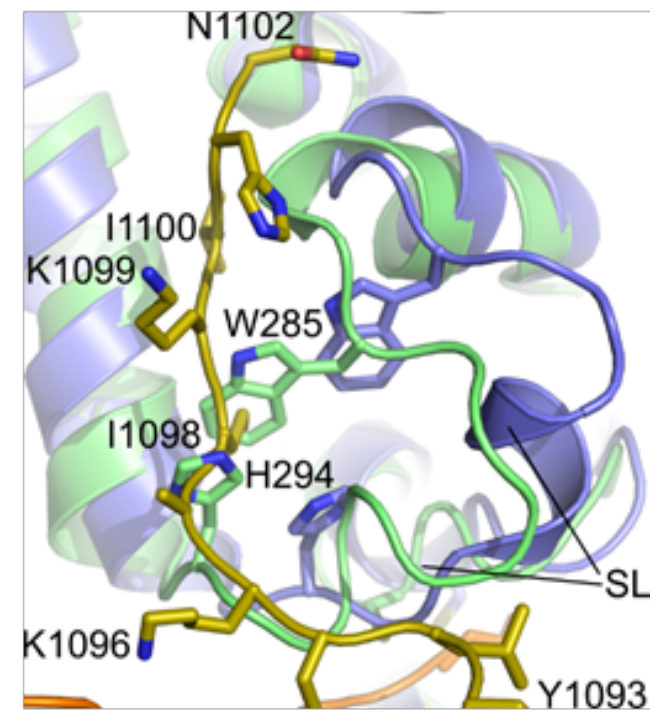

Q

Intensity of USP47 catalytic cysteine-containing peptides before and after incubation with CMPD3

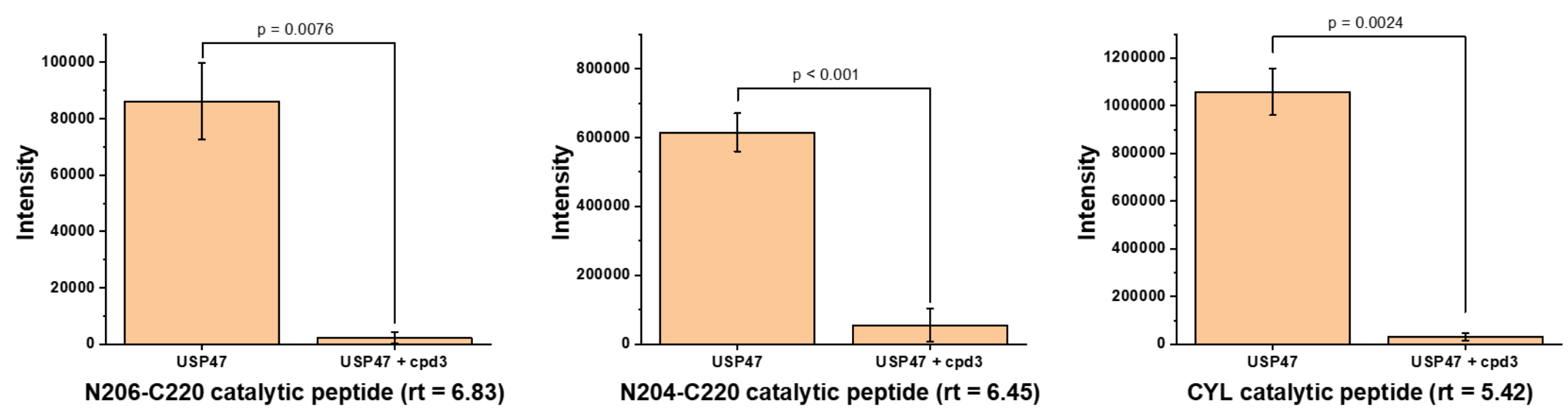

Fig S4. Proposed molecular basis for selective inhibition of USP47 by **Compound 3**. (A-B) Progress curves showing (A) USP47- and (B) USP7-catalysed hydrolysis of Ub-Rho over time (error bars represent SD N=3). (C-D) Progress curves showing (C) USP47- and (D) USP7-catalysed hydrolysis of Ub-Rho in the presence of varying concentrations of **Compound 3**, following preincubation of USP47 or USP7 with **Compound 3** for 30 min (error bars: standard error of the mean, SEM, N=3). (E-F) Progress curves showing (E) USP47 and (F) USP7-catalysed hydrolysis of Ub-Rho in the presence of varying concentrations of **Compound 3**, without preincubation (error bars: SEM, N=3). (G) Proposed outline mechanism for covalent reaction of **Compound 3** with the catalytic cysteine C197 of human USP47. At least two mechanisms for the reaction of **Compound 3** with the nucleophilic cysteine are plausible, with the reaction proceeding via 1,2-carbonyl attack and retro-aldol fragmentation to form (2) possibly being the most probable based on previous observations using a related diepoxide(9). (H) Modelled structure of human USP47 in complex with **Compound 3** shown as a stick representation with carbon atoms colored green. The thumb, palm and fingers subdomains of the catalytic domain and the catalytic cysteine, Cys197, are highlighted. The figure was prepared using PyMOL (The PyMOL Molecular Graphics System, version 2.5.8; Schrödinger, LLC). (**I**) Superimposition of a modelled structure of human USP47 in complex with **Compound 3** based on the X-ray structure of *C.elegans* USP47 in complex with ubiquitin (PDB code: 8ITP(10). The thumb, palm and fingers subdomains, the *C. elegans* USP47 catalytic cysteine point mutation (C97S), and the catalytic cysteine in the human USP47 model (Cys197) are highlighted. The

catalytic domain of the modelled structure of human USP47 in complex with **Compound 3** is colored grey, and **Compound 3** carbons are colored green. The catalytic domain of *C. elegans* USP47 in complex with Ub is colored yellow with Ub in orange. **Compound 3** is predicted to bind in the thumb-palm cleft that is reported to guide the ubiquitin C-terminus into the active site. The positions of BL1, (BL2), and SL are highlighted. (J) Superposition of the modelled structure of human USP47 (grey) in complex with **Compound 3** (green carbon atoms) on a X-ray structure of human USP7 (cyan) in complex with the covalently reacting inhibitor **FT827** (yellow carbon atoms; PDB code: 5NGF (11).The thumb, palm and fingers subdomains, and positions of blocking loops 1 and 2 (BL1, BL2) and switching loop (SL) are highlighted. C* denotes the nucleophilic cysteine (corresponding to C197 in human USP47 and C223 in human USP7). **Compound 3** is predicted to bind within the thumb-palm cleft in a similar manner as **FT827** with **FT827** extending out further towards the fingers subdomain. (K-L) Differential HDX-MS analysis of USP47 and USP7 in apo- and holo-states. A total of 491 peptides covering 88% of the full-length USP47 protein sequence were identified, with an average residue redundancy of 4.2. Similarly, a total of 536 peptides covering 84% of the full-length USP7 sequence were identified, with an average residue redundancy of 5.9. Residual ΔHDX plots showing differences in deuterium uptake between holo- and apo- states for USP47 (K) and USP7 (L), across all mapped peptide fragments and time points (30 s to 1 h). Positive ΔHDX values indicate increased solvent exposure or flexibility in the presence of **Compound 3**, whilst negative values suggest protection or structural stabilisation. A grey shaded area marks the significance threshold for ΔHDX applied in each study. (M) Modelled structure of human USP47 in complex with **Compound 3** (carbon atoms in green) highlighting regions identified in the HDX-MS analysis colored red (mean perturbation over 60 min of 5–20%) and magenta (mean perturbation over 60 min of <60%). The thumb, palm and fingers subdomains, catalytic cysteine (C197), and SL are labelled (N) Human USP7 catalytic domain (from PDB code: 5NGF(11)) colored cyan with the superimposed modelled position of **Compound 3** in human USP47 (green carbon atoms) highlighting regions identified in the HDX-MS analysis colored red (mean perturbation over 60 min of 5–20%) and magenta (mean perturbation over 60 min of <60%). The thumb, palm and fingers subdomains, catalytic cysteine (C223), and SL are labelled. (O) Structure of apo-form human USP7 catalytic domain (PDB code: 1NB8(12); lime) superimposed with a structure of the human USP7 catalytic domain from the USP7-catalytic domain-UBL45-ubiquitin complex (PDB code: 5JTV(13); catalytic domain in violet, the C-terminal activation peptide in gold and ubiquitin in orange). The 19-residue activation peptide at the C-terminus of full length human USP7 stabilizes the SL (especially H294 and W285) in an active "out" conformation. (P) Close-up view of (O) highlighting the conformational shift in SL between the inactive "in" conformation in apoform USP7 and active "out" conformation in its complex with ubiquitin. (Q) Relative intensities of USP47 catalytic cysteine-containing peptides detected by MS/MS following incubation of recombinant USP47 with vehicle or Compound 3. Peptides spanning N206–C220 and the catalytic CYL motif showed markedly reduced signal intensity after Compound 3 treatment, consistent with Compound 3-dependent modification or reduced detectability of peptides containing the USP47 catalytic cysteine. Bars represent mean ± SEM from three independent measurements. Statistical significance was determined using an unpaired two-tailed Welch's t-test; p values are indicated on the plots.

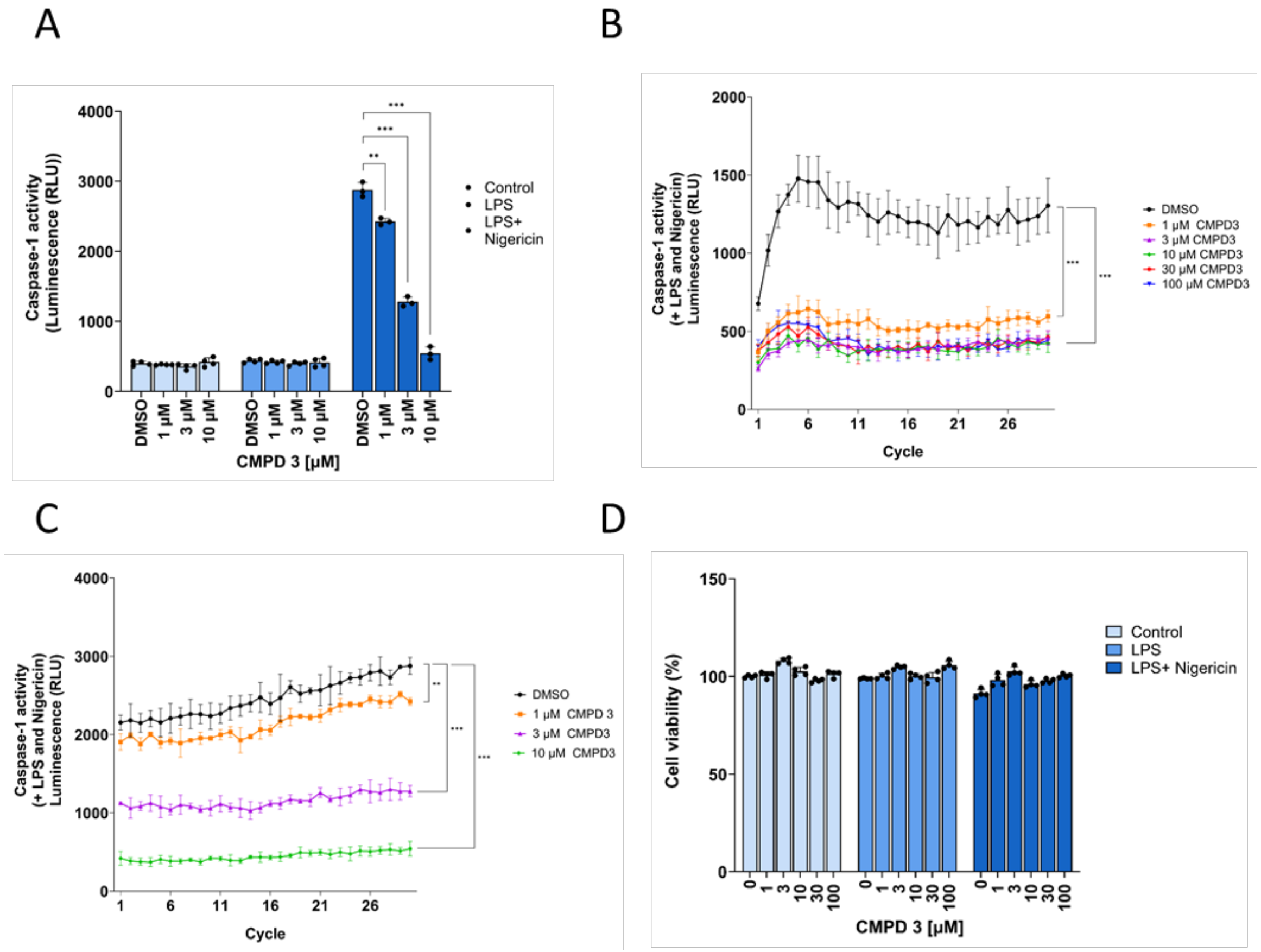

Fig S5. Compound 3 inflammasome activation and toxicity. (A) Caspase-1 activity in the media (supernatant) of PMA-differentiated THP-1 cells treated with the indicated concentrations of **Compound 3**, determined using the commercial Caspase-Glo 1 luminescence assay. **Compound 3** incubation for 4 h, ± 1 μg/mL LPS incubation for 4 h, and 10 μM Nigericin incubation for 2 h (error bars = SD, N=4). (B-C) Caspase-1 activity over time in the media (supernatant) of PMA-differentiated THP-1 cells treated with the indicated concentrations of **Compound 3**, determined using the Caspase-Glo 1 luminescence assay. **Compound 3** incubation for 4 h, 1 μg/mL LPS incubation for 4 h, and 10 μM Nigericin incubation for 45 min (B) and 2 h (C) (error bars: standard deviation, SD, (B N=4) (C N=3)). Two-way ANOVA with Dunn's multiple comparison test was used. ***P < 0.001. (D) MTS cell viability of THP-1 cells treated with indicated concentrations of **Compound 3** ± LPS or LPS/Nigericin. Compound treatment for 4 h, 1 μg/mL LPS treatment for 4 h, 10 μM Nigericin treatment for 45 min (error bars = SD, N=4).

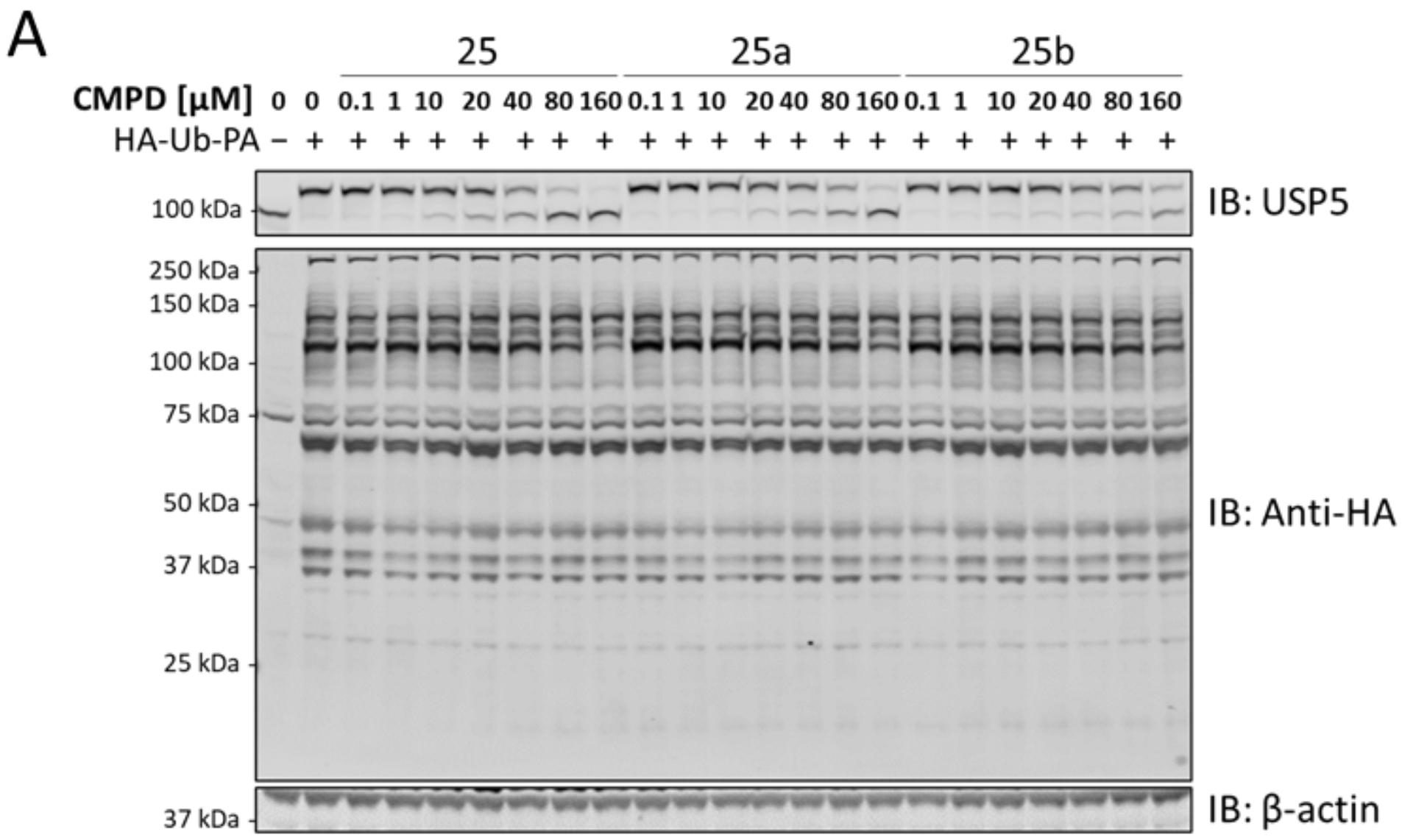

Fig S6. Selective inhibition of USP5 by **Compounds 25**, **25a** and **25b**. HA immunoblot showing concentration-dependent selective binding of **Compounds 25**, **25a** and **25b** to USP5 in MCF-7 cell lysates using HA-Ub-PA ABP.

A

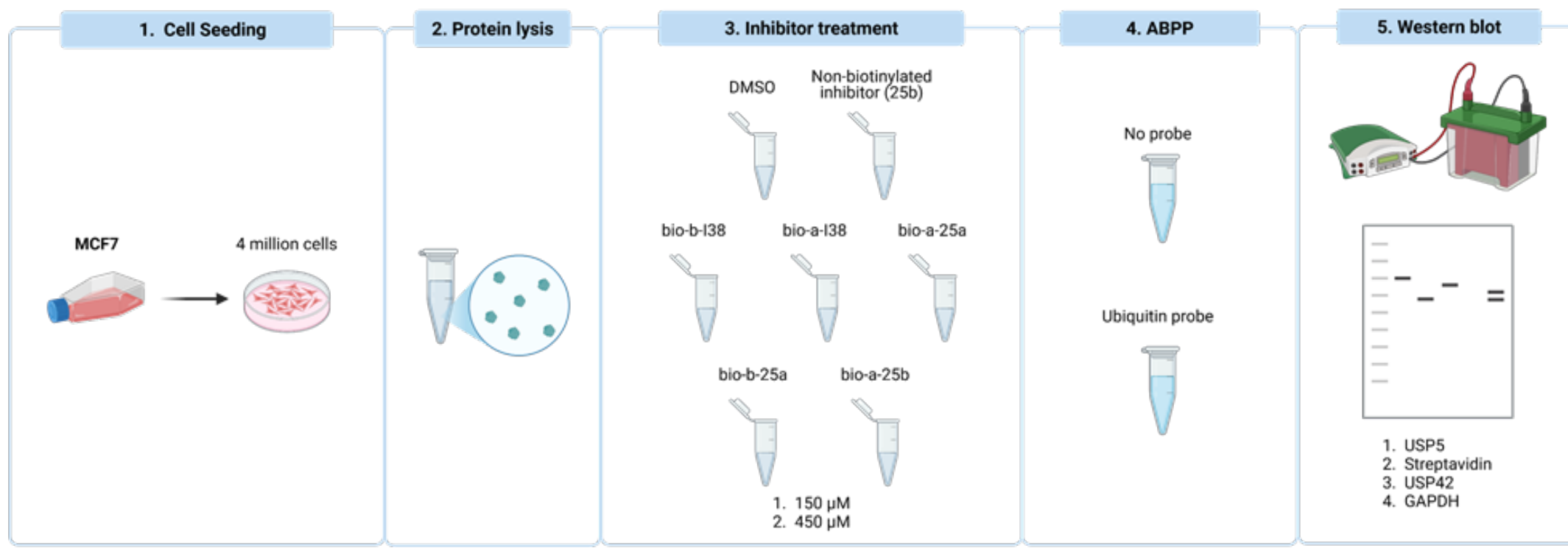
1. Cell Seeding
MCF7
4 million cells
2. Protein lysis
3. Inhibitor treatment
DMSO
Non-biotinylated inhibitor (25b)
bio-b-I38
bio-a-I38
bio-a-25a
bio-b-25a
bio-a-25b
1. 150 μM
2. 450 μM
4. ABPP
No probe
Ubiquitin probe
5. Western blot
1. USP5
2. Streptavidin
3. USP42
4. GAPDH

B

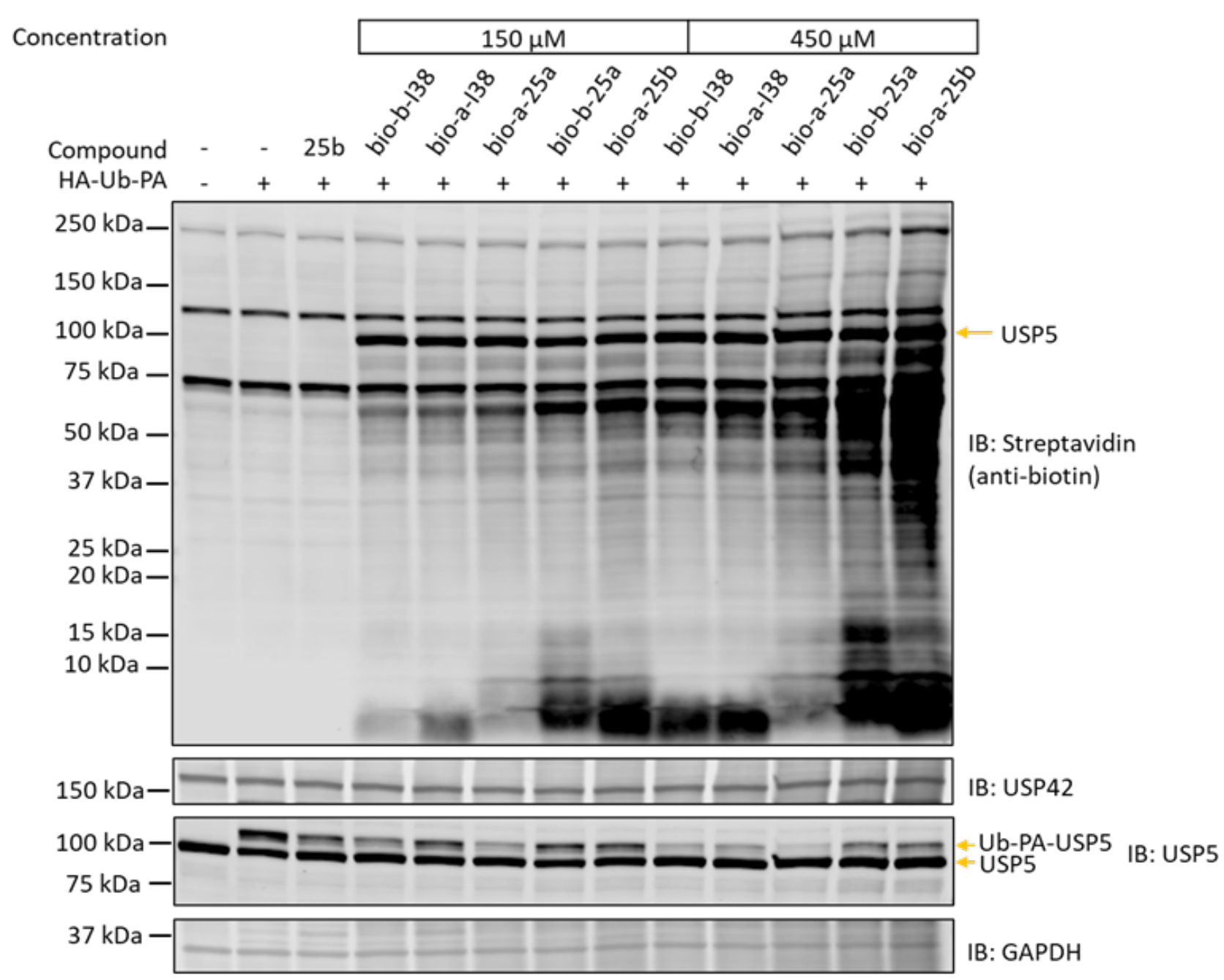
Concentration
150 μM
450 μM
Compound
HA-Ub-PA
25b
bio-b-I38
bio-a-I38
bio-a-25a
bio-b-25a
bio-a-25b
250 kDa
150 kDa
100 kDa
75 kDa
50 kDa
37 kDa
25 kDa
20 kDa
15 kDa
10 kDa
USP5
IB: Streptavidin (anti-biotin)
IB: USP42
Ub-PA-USP5
USP5
IB: USP5
IB: GAPDH

C

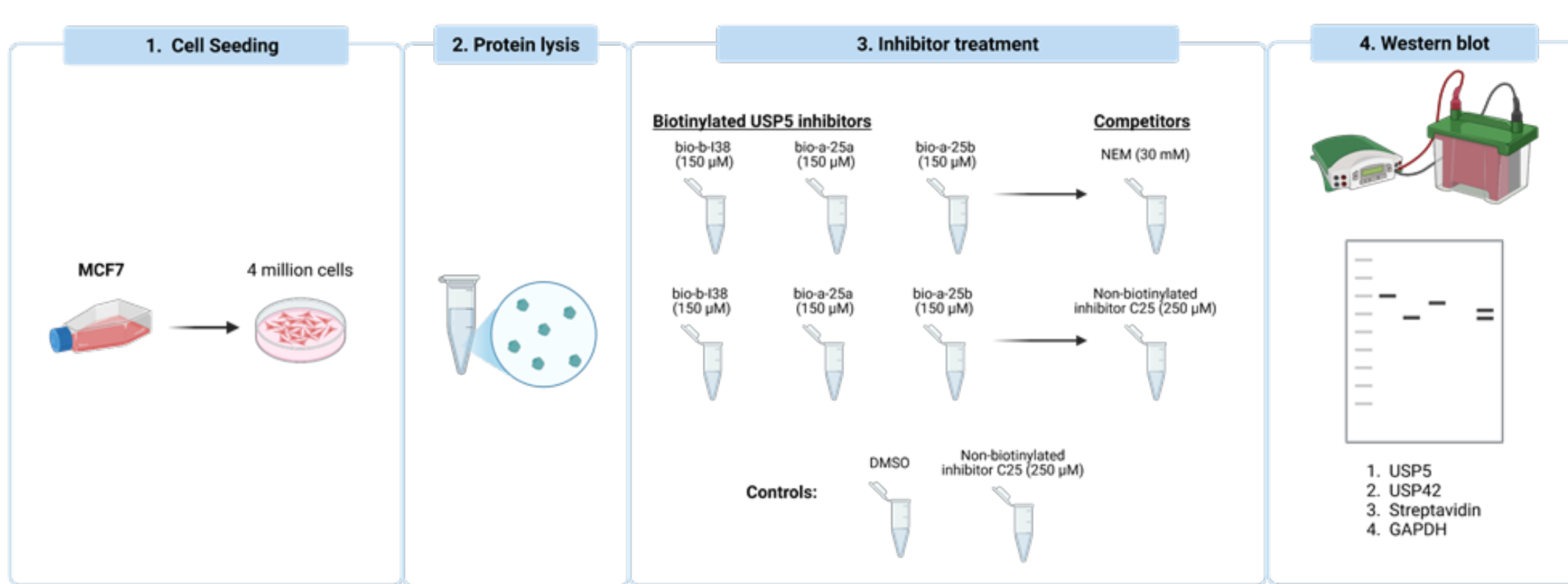
1. Cell Seeding
MCF7
4 million cells
2. Protein lysis
3. Inhibitor treatment
Biotinylated USP5 inhibitors
bio-b-I38 (150 μM)
bio-a-25a (150 μM)
bio-a-25b (150 μM)
Competitors
NEM (30 mM)
Non-biotinylated inhibitor C25 (250 μM)
Controls:
DMSO
Non-biotinylated inhibitor C25 (250 μM)
4. Western blot
1. USP5
2. USP42
3. Streptavidin
4. GAPDH

D

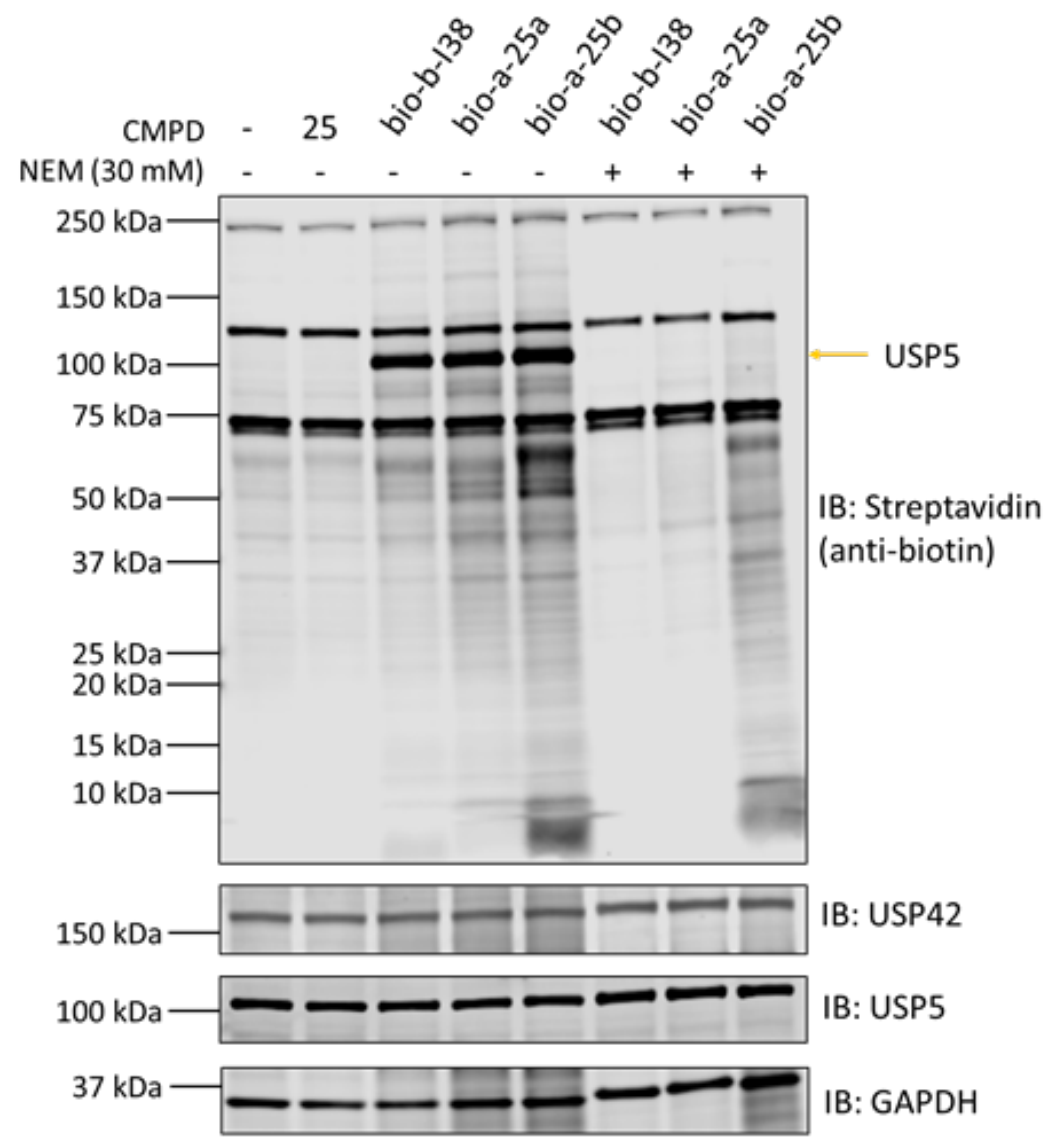

Fig S7. USP5 binding studies using biotinylated **Compound 5**. (A) Experimental Workflow for ABPP (created with BioRender.com) HA-Ub-PA ABP labeling of USP5 in MCF-7 cell lysates with biotinylated USP5 inhibitor treatments. (B) MCF-7 cell lysates were incubated with a non-biotinylated USP5 inhibitor (Compound 25b at 150 μM), or with biotinylated USP5 inhibitors (at 150 or 450 μM) prior to HA-Ub-PA ABP labeobabling. Unlabelled and probe-labelled USP5 was detected using an anti-USP5 antibody; inhibitor-labelled USP5 was detected using streptavidin (anti-biotin) immunoblotting. (C) Experimental workflow for assessing binding of biotinylated USP5 inhibitors (created with BioRender.com). (D) MCF-7 lysates were incubated with DMSO or 30 mM of N-ethylmaleimide (NEM) for 1 h, followed by incubation with 150 μM of biotinylated Compound 25 derivative inhibitors for 1 h. USP5, USP42 and GAPDH were detected with immunoblotting. An anti-biotin immunoblot with streptavidin was used to visualize USP5-bound biotinylated USP5 inhibitors.

A

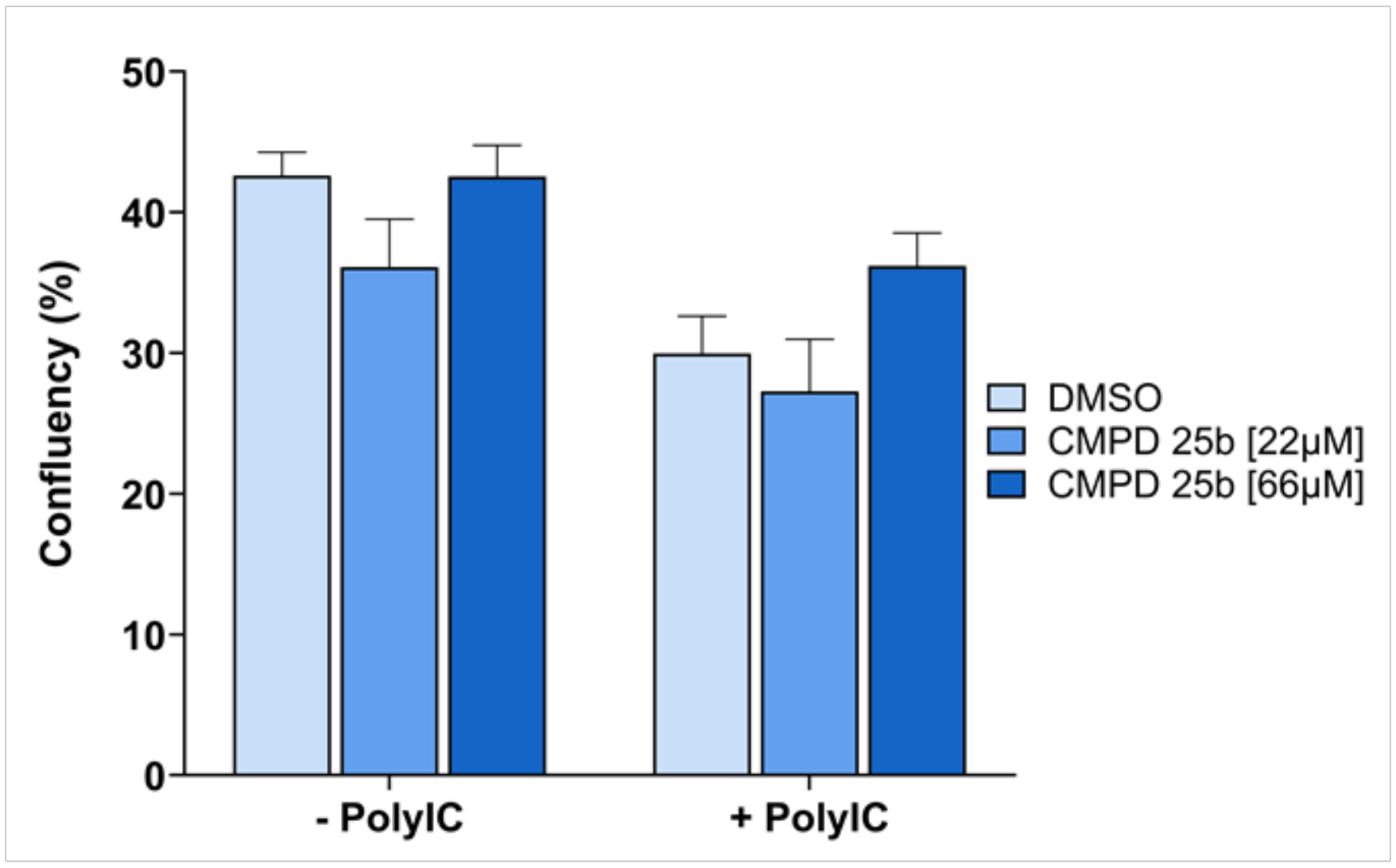

B

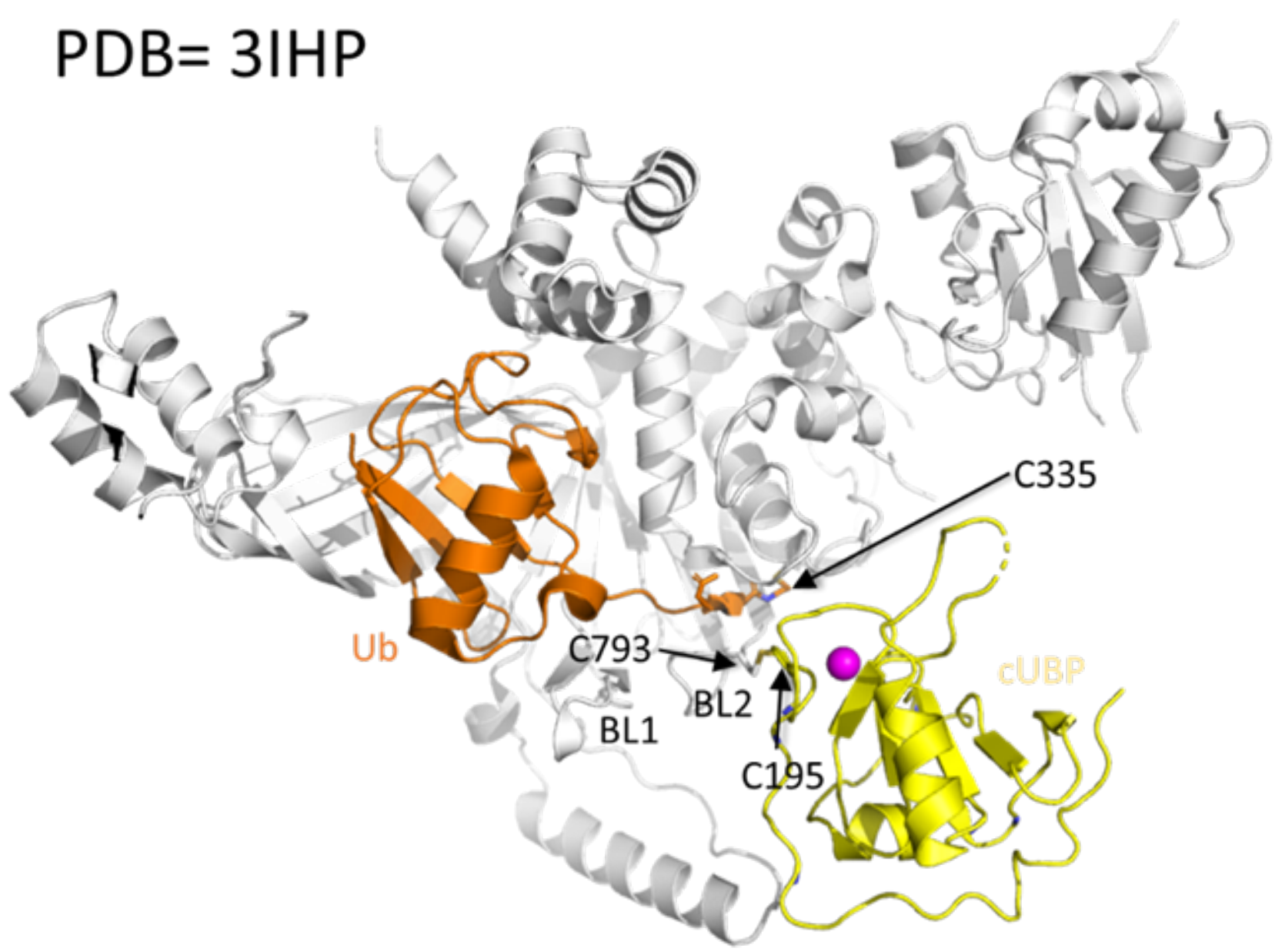

Fig S8. Studies on cellular toxicity and mode of action of **Compound 25b**. (A) HAP1 cells were seeded in triplicate at 5,000 cells per well in a 96-well plate, then treated with **Compound 25b** ± Poly(I:C) 0.5 µg/mL for 48 h. Confluence at 48 h was determined by live-cell imaging using an IncuCyte SX5 machine (Sartorius) (error bars = SEM, N = 3). (B) Structure of the reported covalent USP5–Ub complex (PDB ID: 3IHP(14)) revealing a disulphide bridge between C793 in the BL2 loop and C195 in the cUBP domain, observed in chains A and B. This tethering may restrict BL2 flexibility and position cUBP near the putative **Compound 25** binding site, potentially enabling additional interactions. As C195 is also implicated in USP5 dimerisation, the functional relevance of this disulphide bond warrants further investigation.

A

Recombinant USP47 *vs.* Canonical Human USP47
His-Tagged
14-24del
G163V
D808E
V834G
H1049Y

B

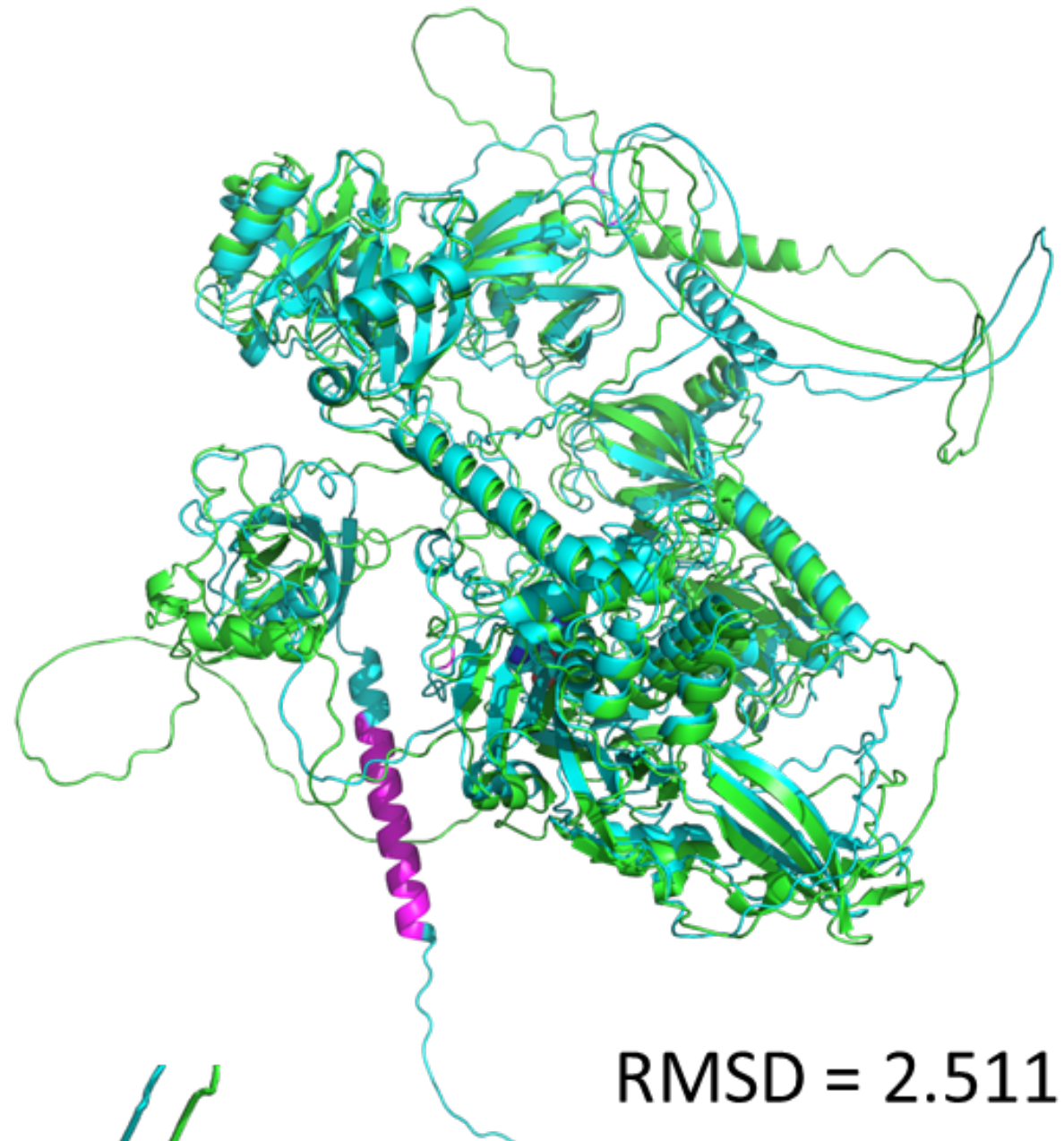

C

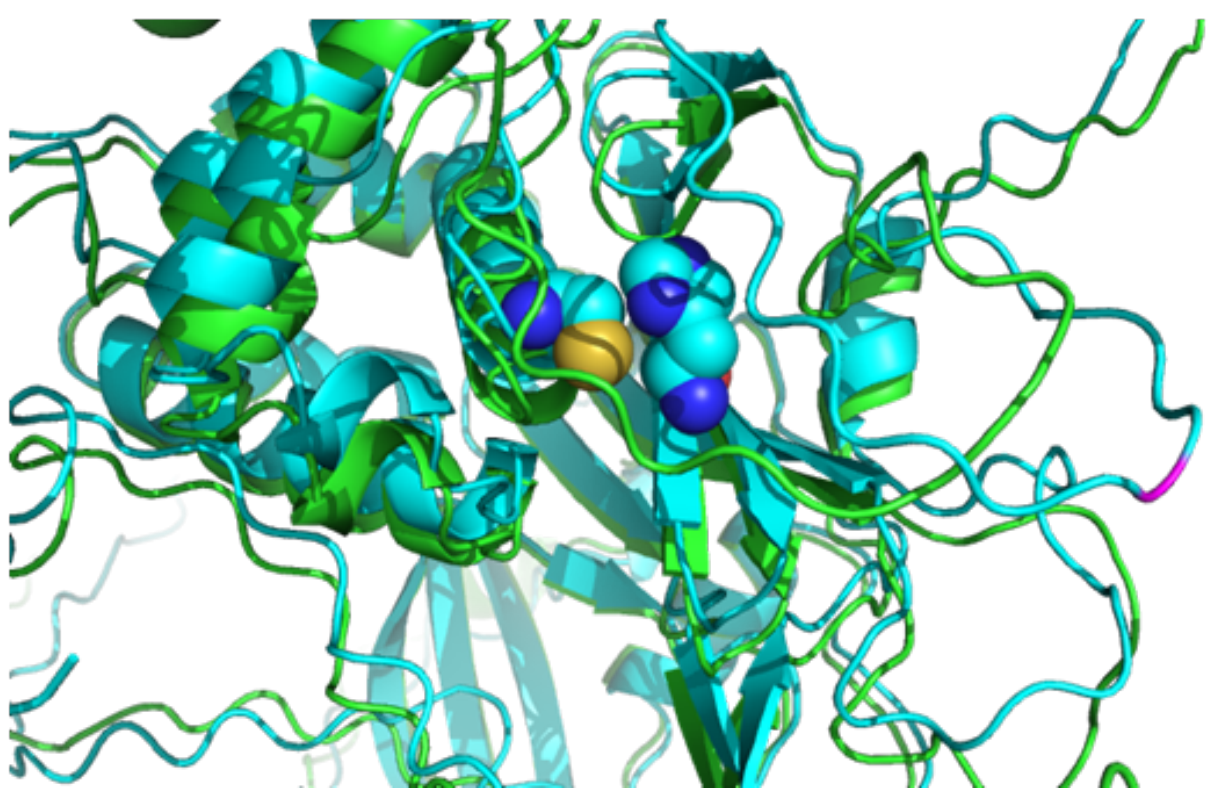

Fig S9. USP47 protein *Pan troglodytes* isoform 2 *vs Homo sapiens* isoform 1. (A) Sequence differences between USP47 of *P. troglodytes* isoform 2 (Accession XP_001171097) and canonical human USP47 (Accession NP_001317137). (B) Alignment of AlphaFold structures of USP47 based on *P. troglodytes* isoform 2 (Accession XP_001171097) used in kinetic and HDX-MS studies and canonical human USP47 (Accession NP_001317137). Sequence differences are in pink, the catalytic cysteine 197 and histidine 503 residues are as spheres. (C) Close up view of alignment showing the catalytic cysteine 197 and histidine 503 residues as spheres and the nearest sequence difference in pink.

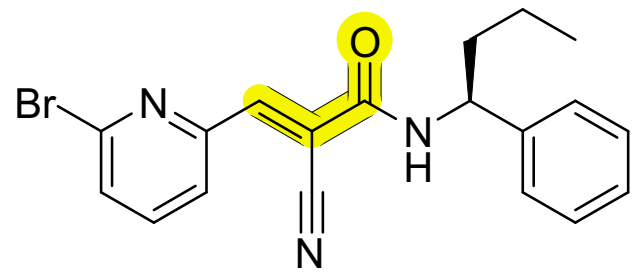

Degrasyn (WP1130)

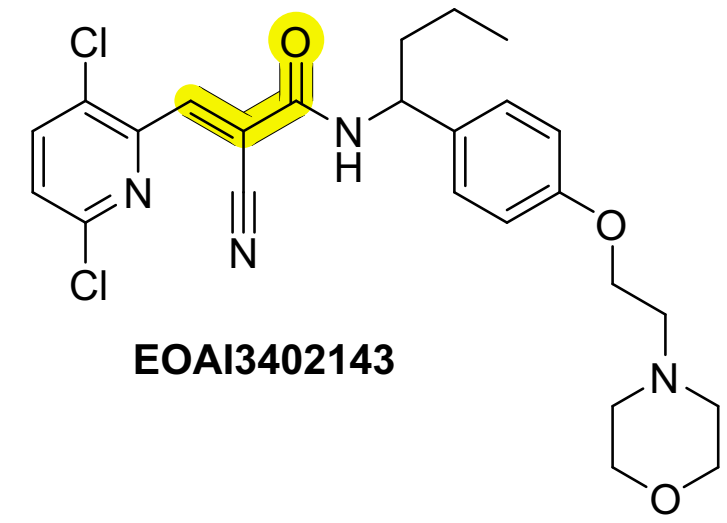

EOAI3402143

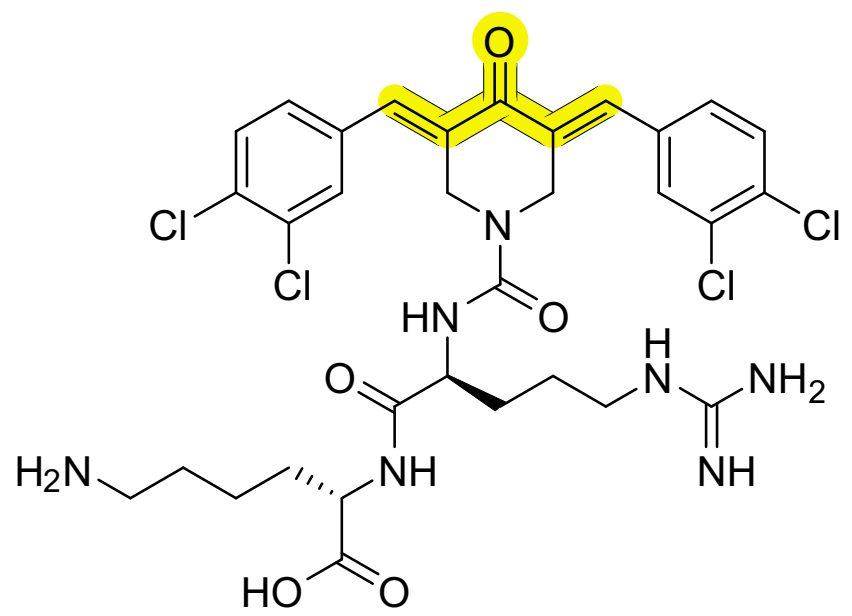

RA-9

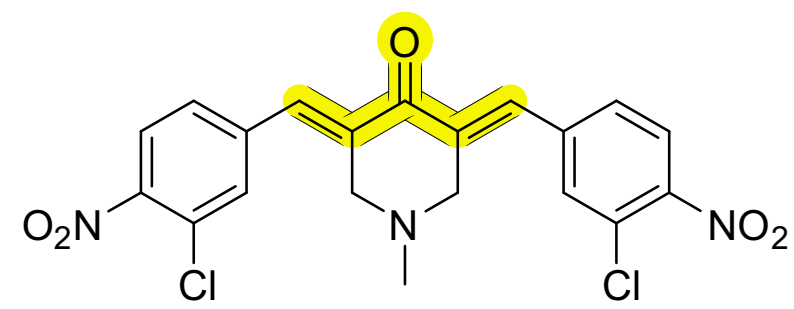

RA-14

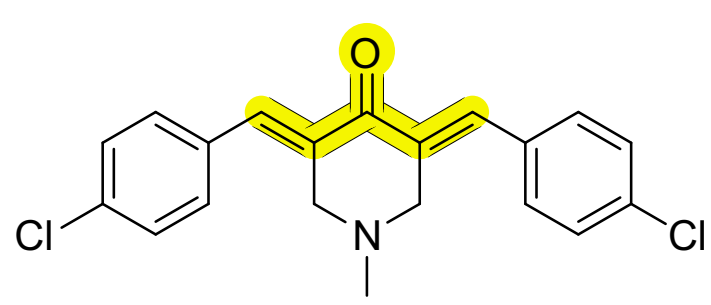

AM146

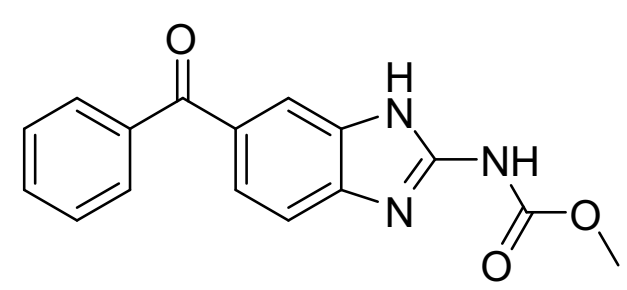

Mebendazole

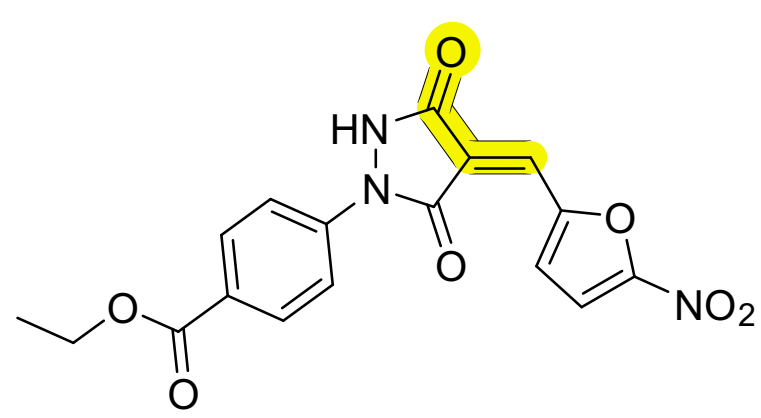

PYR-41

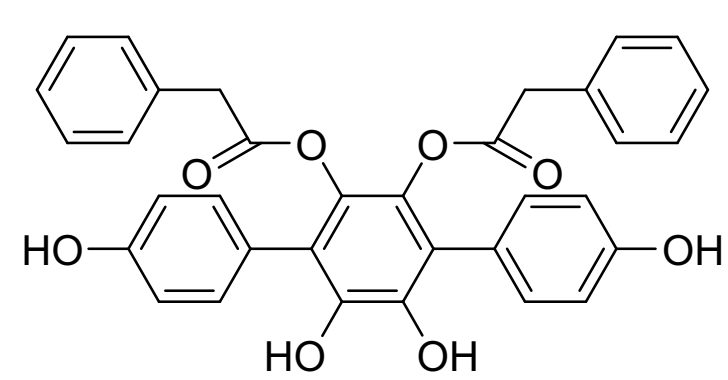

Vialinin A

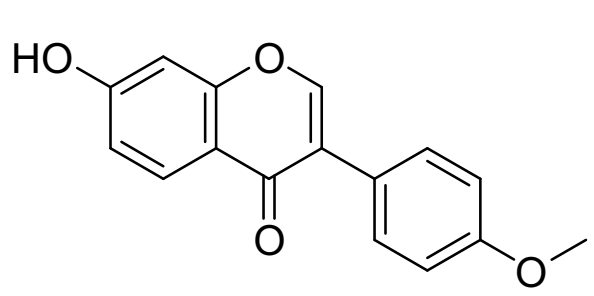

Formononetin

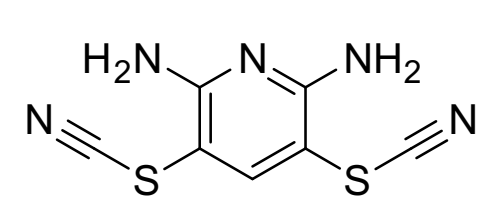

PR 619

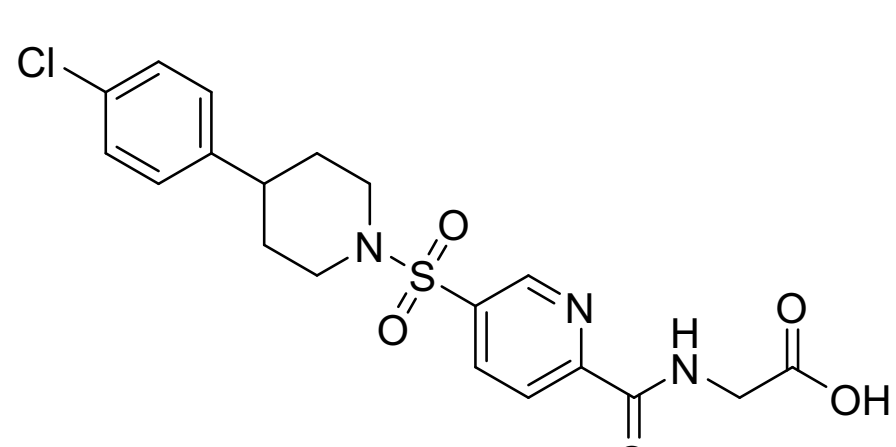

64

Fig S10. Selected reported small-molecule USP5 inhibitors. Several small-molecule USP5 inhibitors are reported, none of which to our knowledge, have yet advanced into clinical testing (15). Some of the reported small-molecule USP5 inhibitors likely work via covalently reaction with USP5. **Degrasyn** (WP1130): Degrasyn is a pan-DUB inhibitor, which in addition to USP5 (IC_{50} = 2.5 μM), is also reported to inhibit USP9x, USP14, USP37, and UCHL1, as well as other nucleophilic cysteine enzymes (16, 17). It likely inhibits USP5 via covalent reaction. **EOAI3402143**: Like the structurally related Degrasyn. EOAI3402143 is a broad-spectrum-DUB inhibitor (A375 cell line IC_{50} = 1 μM), which in addition to USP5, is reported to also inhibit USP9x and USP24(18, 19, 20). **PR619**: PR619 is a pan-DUB inhibitor, which in addition to USP5 (EC_{50} = 8.6 μM), is reported to also inhibit, *e.g.*, USP2, USP4, USP7, USP8, USP15, USP20, USP28;(21) it likely inhibits via cyanide transfer.(17) **Dibenzylideneacetones RA-9, RA-14, AM146**: These dibenzylideneacetone derivatives are cell-permeable USP5 inhibitors, which likely works via covalent reaction. However, they display imperfect selectivity and also inhibit UCHL1, UCHL3, USP2, and USP8.(22) They inhibit HeLa cell viability with IC_{50} values of 2.37 μM (RA-9), 2.03 μM (RA-14), and 1.83 μM (AM146).(22) **PYR-41**: Inhibits various DUBs including USP5 (IC_{50} = ~10 μM), USP9x, USP14, UCH37, and UCHL3.(23) **Vialinin A**: Inhibits USP5 (IC_{50} = 5.9 μM), USP4 and UCHL1.(24) **Mebendazole**: Mebendazole is clinically used to treat parasite infections.(25) Notably, it is reported that Mebendazole also efficiently inhibits USP5 and USP7, suppressing c-Maf transcription, and inhibits MCF-10 cells at IC_{50} = 0.80 μM. (26) Note that its effects on other DUBs have, to our knowledge, not yet been described. **Formononetin** inhibits USP5 (K_D = 25.1 μM) and structurally related USP13.(27, 28) **Compound 64** manifests at least partial selectivity for USP5 inhibition (IC_{50} = 46 μM, K_D = 2.6 μM).(17, 29)

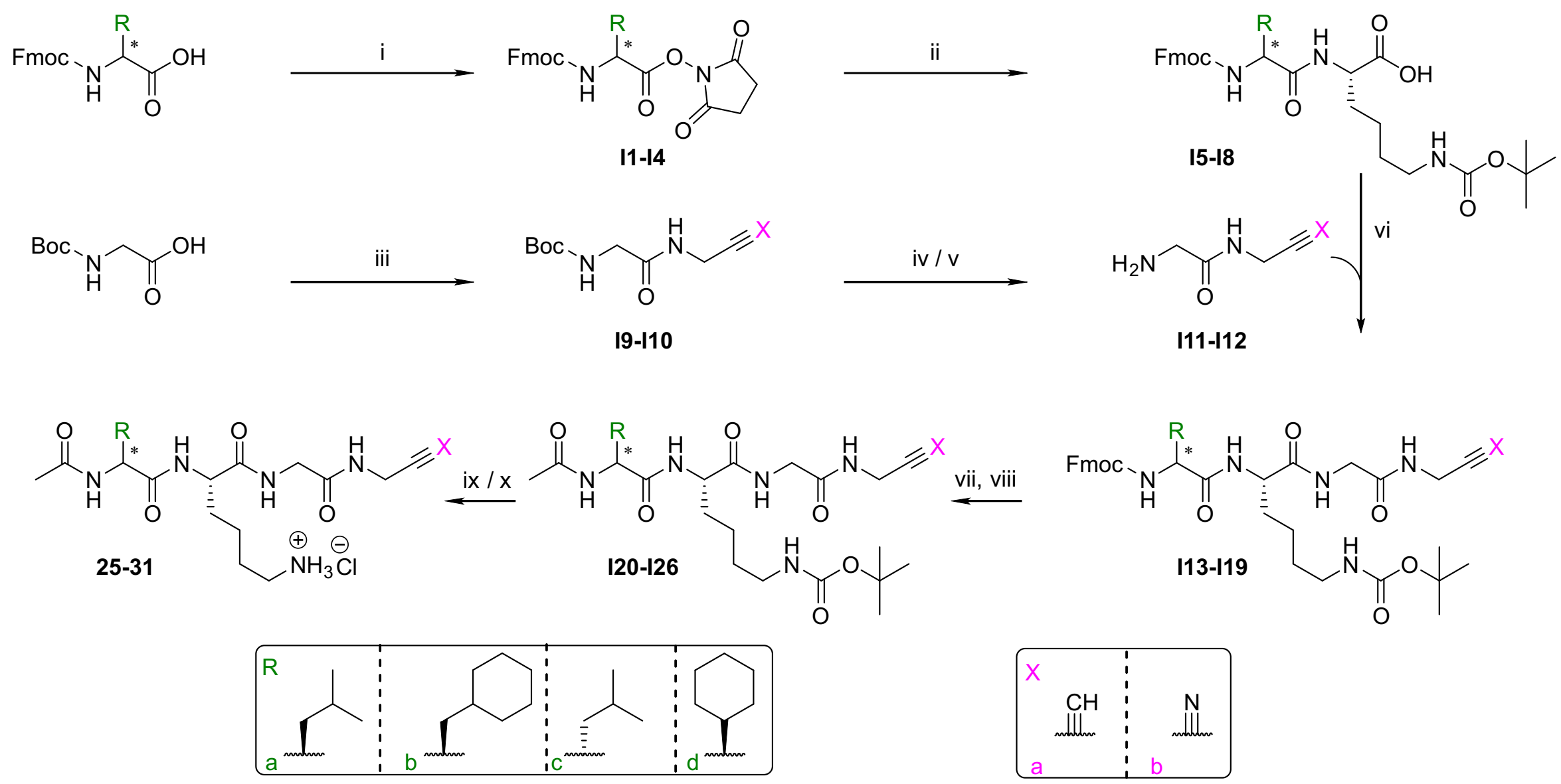

Compound	R	X	Conditions
**25**	a	a	iv, x
**26**	b	a	iv, x
**27**	c	a	iv, x
**28**	a	b	v, ix
**29**	d	b	v, ix
**30**	c	b	v, ix
**31**	b	b	v, ix

Fig S11. Synthesis of Ub-derived **Compounds 25-31** for DUB inhibition. Reagents and conditions: i) *N*-hydroxysuccinimide (NHS), *N*,*N*'-dicyclohexylcarbodiimide (DCC) (1), tetrahydrofuran, 0 °C to rt, 89%-app. quant.; ii) Lys(Boc)-OH, Na_2CO_3, tetrahydrofuran:water (2:1$_{v/v}$), 0 °C, 87-98%; iii) amine salt, *N*-(3-dimethylaminopropyl)-*N*'-ethylcarbodiimide (EDC) (2), 1-hydroxybenzotriazole hydrate (HOBt), *N*-methylmorpholine (NMM), DCM, rt, 50-60%; iv) HCl in dioxane (4M), DCM, rt, app. quant.; v) formic acid, rt, app. quant.; vi) NMM, isobutyl chloroformate (IBCF), tetrahydrofuran:water (3:1$_{v/v}$), –15 °C to rt, 33-97%; vii) *N*,*N*-diethylamine, MeCN, rt, app. quant.; viii) 4-(*N,N*-dimethylamino)pyridine (DMAP), Ac_2O, tetrahydrofuran, reflux, 23-61%; ix) formic acid, rt; then: HCl in dioxane (4M), 66%-app. quant.; x) HCl in dioxane (4M), 2-propanol, rt, 78%-app. quant.

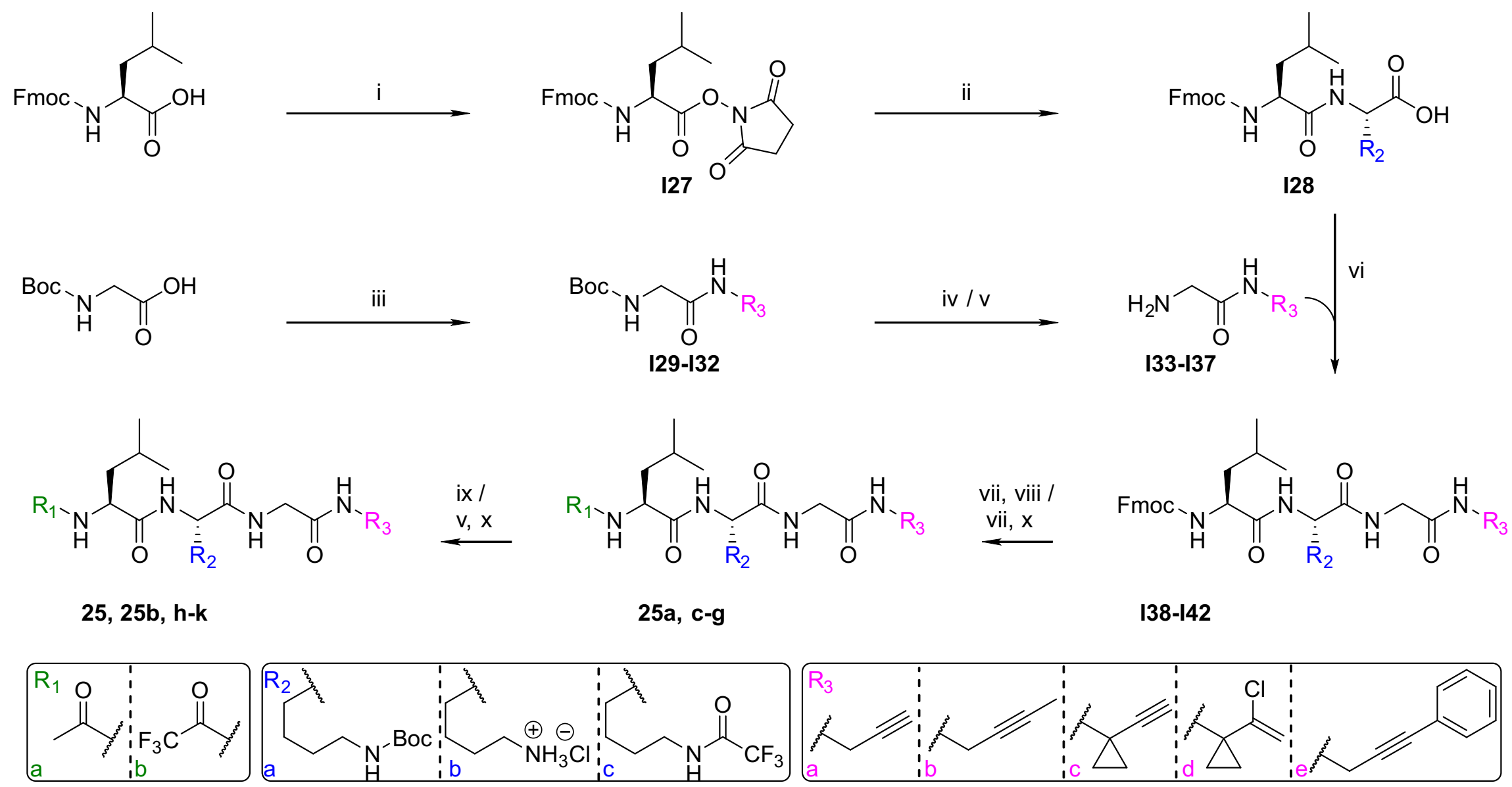

Compound	R_1	R_2	R_3	Conditions	Compound	R_1	R_2	R_3	Conditions
**I28**	-	a	-		**I42**	-	a	e	
**I29**	-	-	a		**25d**	a	a	a	vii, viii
**I30**	-	-	b		**25e**	a	a	b	vii, viii
**I31**	-	-	c		**25f**	a	a	c	vii, viii
**I32**	-	-	e		**25c**	a	a	d	vii, viii
**I33**	-	-	a	iv	**25g**	a	a	e	vii, viii
**I34**	-	-	b	iv	**25a**	b	a	a	vii, x
**I35**	-	-	c	v	**25**	a	b	a	ix
**I36**	-	-	d	iv	**25j**	a	b	b	ix
**I37**	-	-	e	iv	**25h**	a	b	c	ix
**I38**	-	a	a		**25i**	a	b	e	ix
**I39**	-	a	b		**25k**	b	b	a	ix
**I40**	-	a	c		**25b**	b	c	a	v, x
**I41**	-	a	d						

Fig S12. Synthesis of Ub-derived **Compounds 25a-25k** for DUB inhibition.Reagents and conditions: i) *N*-hydroxysuccinimide (NHS), *N*,*N'*-dicyclohexylcarbodiimide (DCC) (1), tetrahydrofuran, 0 °C to rt, 66%; ii) Lys(Boc)-OH, Na_2CO_3, tetrahydrofuran:water (2:1$_{v/v}$), 0 °C, app. quant.; iii) amine salt, *N*-(3-dimethylaminopropyl)-*N'*-ethylcarbodiimide (EDC) (2), 1-hydroxybenzotriazole hydrate (HOBt), *N*-methylmorpholine (NMM), DCM, rt, 54-85%; iv) HCl in dioxane (4M), DCM, rt, app. quant.; v) formic acid, rt, app. quant.; vi) NMM, isobutyl chloroformate (IBCF), tetrahydrofuran:water (3:1$_{v/v}$), –15 °C to rt, 91-100%; vii) *N*,*N*-diethylamine, MeCN, rt, app. quant.; viii) 4-(*N*,*N*-dimethylamino)pyridine (DMAP), Ac_2O, tetrahydrofuran, reflux, 1-21%; ix) formic acid, rt; then: HCl in dioxane (4M), 58-87%; x) DMAP, trifluoroacetic anhydride (TFAA), tetrahydrofuran, reflux, 1-11%.

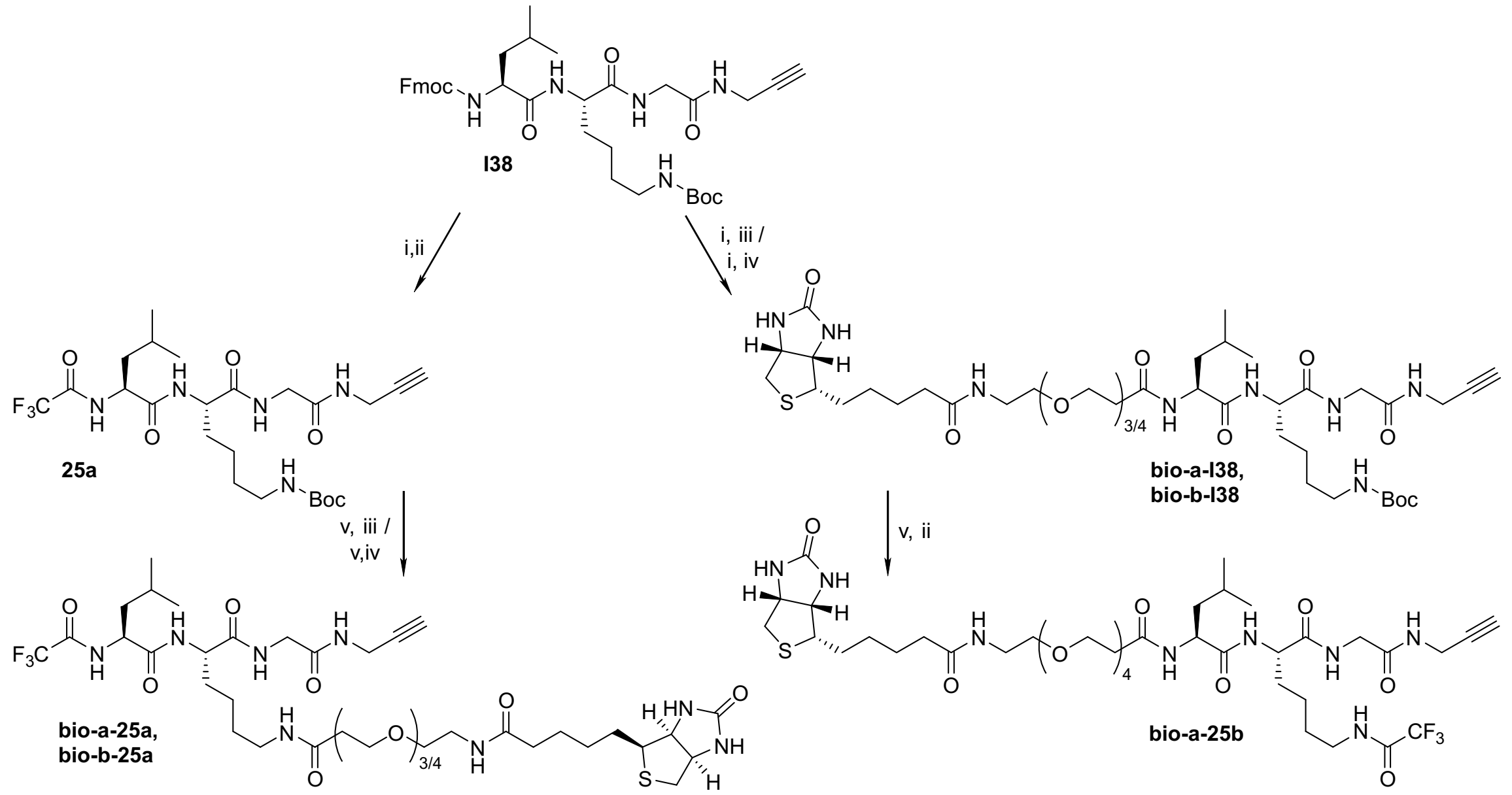

Compound	Conditions
**bio-a-25a (31)**	v, iv
**bio-b-25a (32)**	v, iii
**bio-a-I38 (34)**	i, iv
**bio-b-I38 (33)**	i, iii
**bio-a-25b (35)**	v, ii

Fig S13. Synthesis of biotinylated derivatives of **Compound 25**. Reagents and conditions: i) *N*,*N*-diethylamine, MeCN, rt, app. quant.; ii) 4-(*N,N*-dimethylamino)pyridine (DMAP), trifluoroacetic anhydride (TFAA), tetrahydrofuran, reflux, 2-11%; iii) Biotin-PEG3-NHS ester, aqueous saturated, Na_2CO_3, *N,N*-dimethylformamide (DMF), rt, 30-40%; iv) Biotin-PEG4-NHS ester, aqueous saturated, Na_2CO_3, DMF, rt, 43-65%; v) formic acid, rt, app. quant.

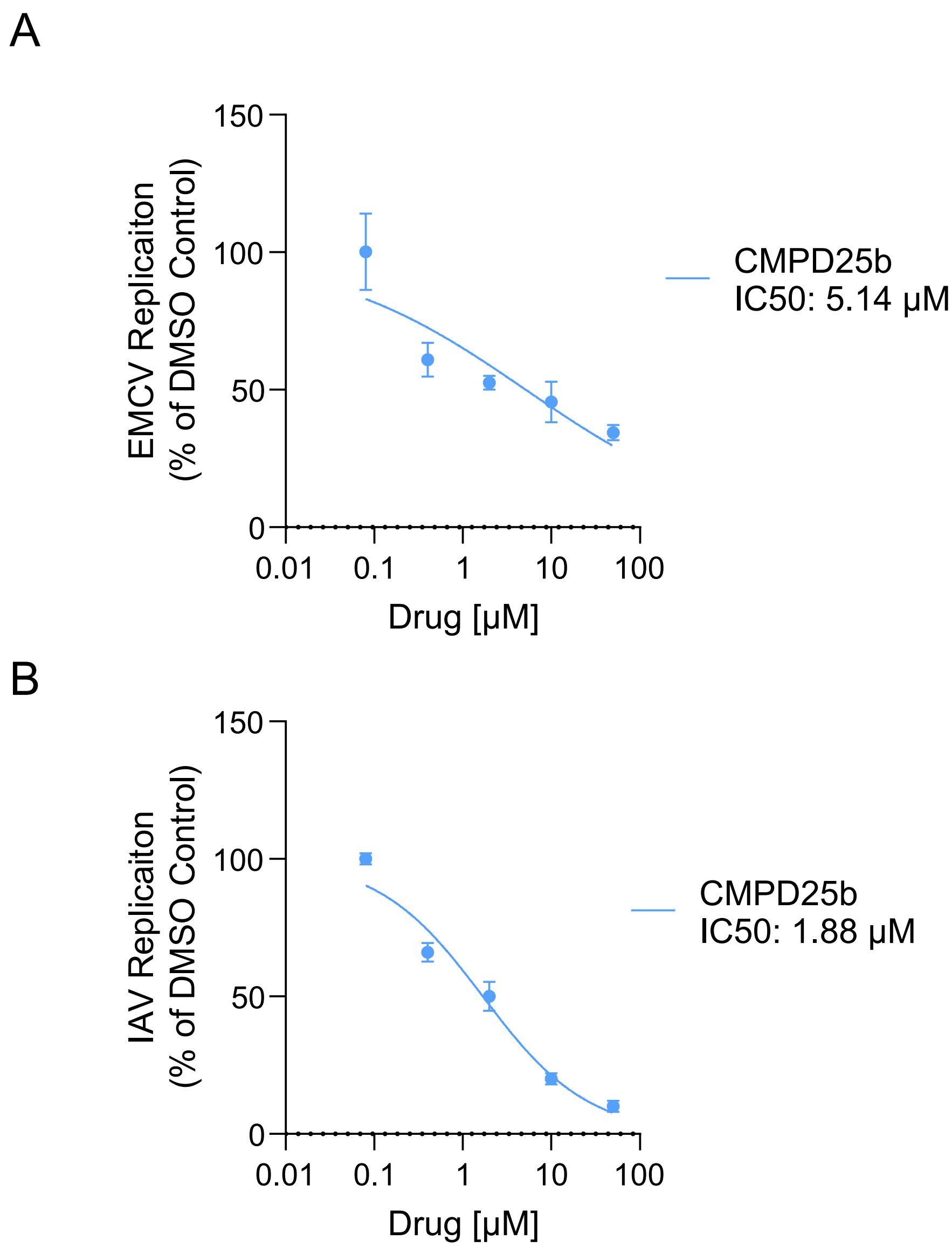

Fig S14. Compound 25b shows antiviral activity against IAV and EMCV. Dose-response curves showing the effect of **Compound 25b** on influenza A virus (IAV) and encephalomyocarditis virus (EMCV) replication. Viral signal was normalized to vehicle-treated infected controls and plotted as mean ± SEM from three independent replicates. IC50 values were determined by nonlinear regression and correspond to 1.88 μM for IAV and 5.14 μM for EMCV.

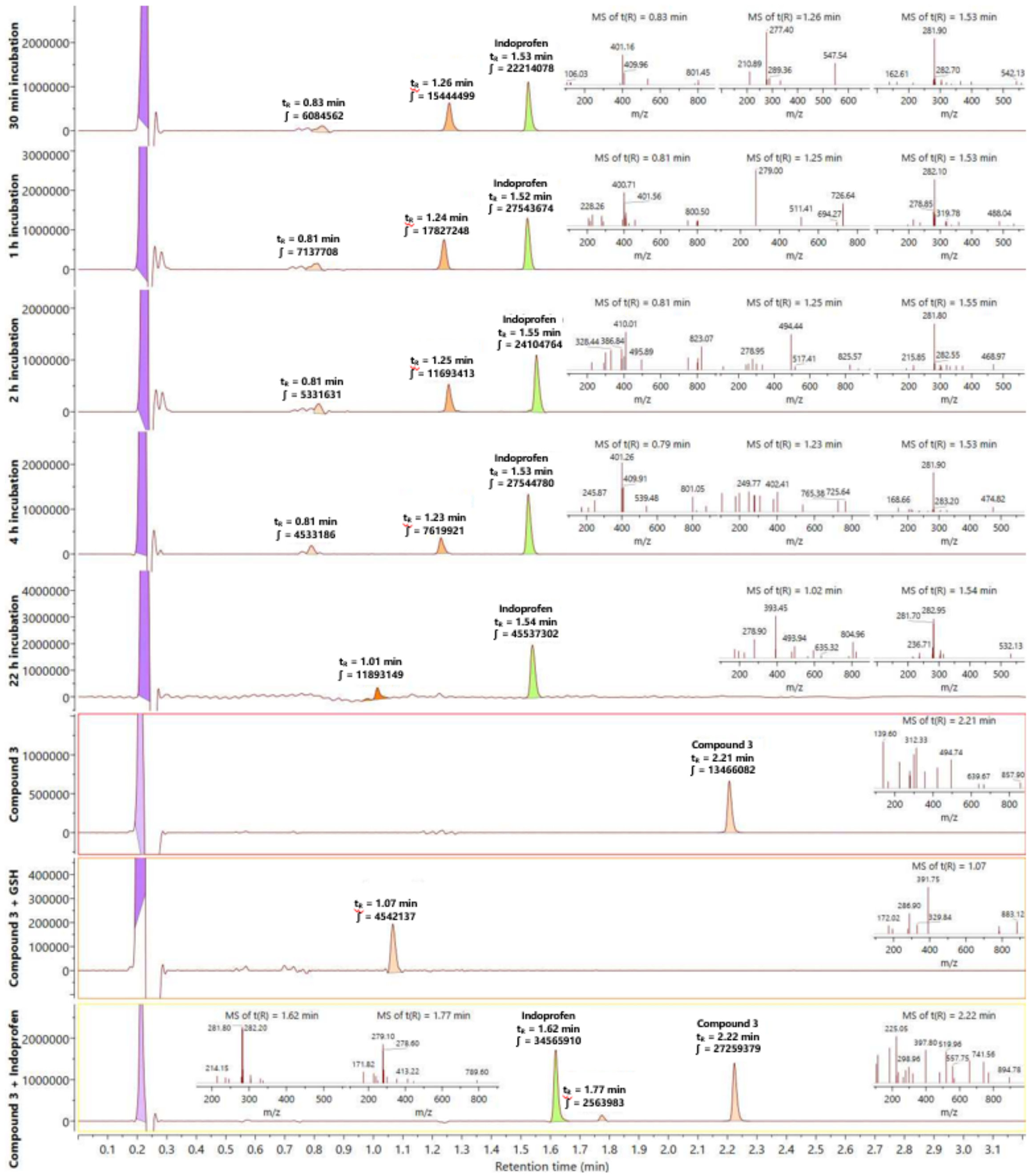

Fig S15. GSH stability assay with L-glutathione (blueviolet), **Compound 3** (orange), and indoprofen (chartreuse). **Compound 3** (292.11 Da) is not stable in assay conditions over 22 h incubation: By itself (no GSH or indoprofen) it ionizes poorly and seems to degrade (red frame), in presence of GSH only (orange frame), the retention time and ionization changes drastically indicating reaction, but the lack of expected mass (493.15 Da) suggests degradation taking place during and/or after reaction. In addition to the poorly ionizing peak corresponding to **Compound 3** at retention time t_R = 2.22 min, in presence of indoprofen without GSH (yellow frame), an additional peak at t_R = 1.77 min indicated the possibility of **Compound 3** reacting with the internal standard indoprofen. t(R) corresponds to retention time (t(R) =t_R).

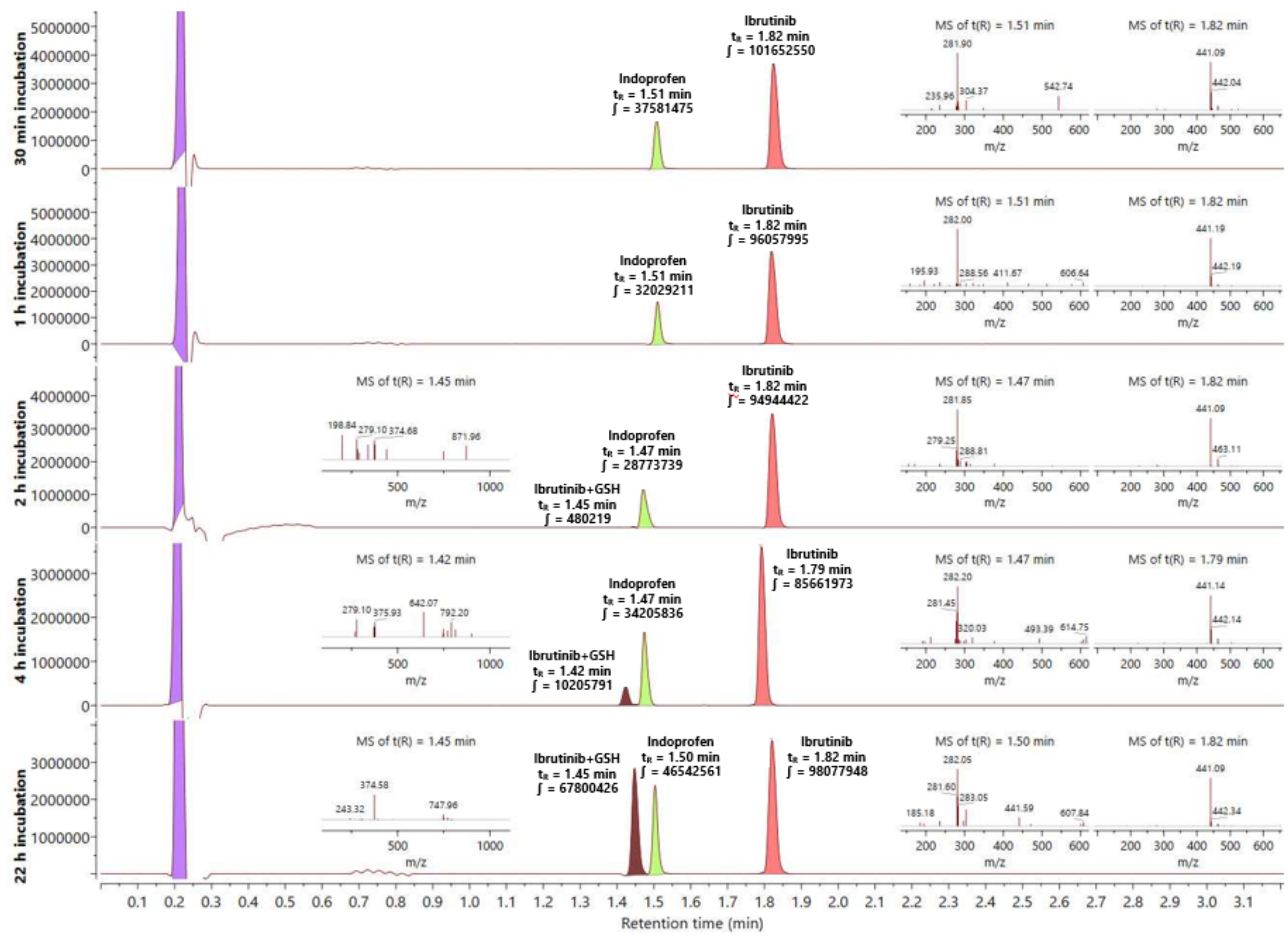

Fig S16. Incubations of L-glutathione (blueviolet), Ibrutinib (red; 440.20 Da), and indoprofen (chartreuse; 281.11 Da). Ibrutinib was used as a control because it is reported to react with GSH (30). The expected adduct masses (747.28 Da and 749. 30 Da) are found in UV traces at 4 and 22 h, in a new peak at a retention time (t_R) = 1.45 min.

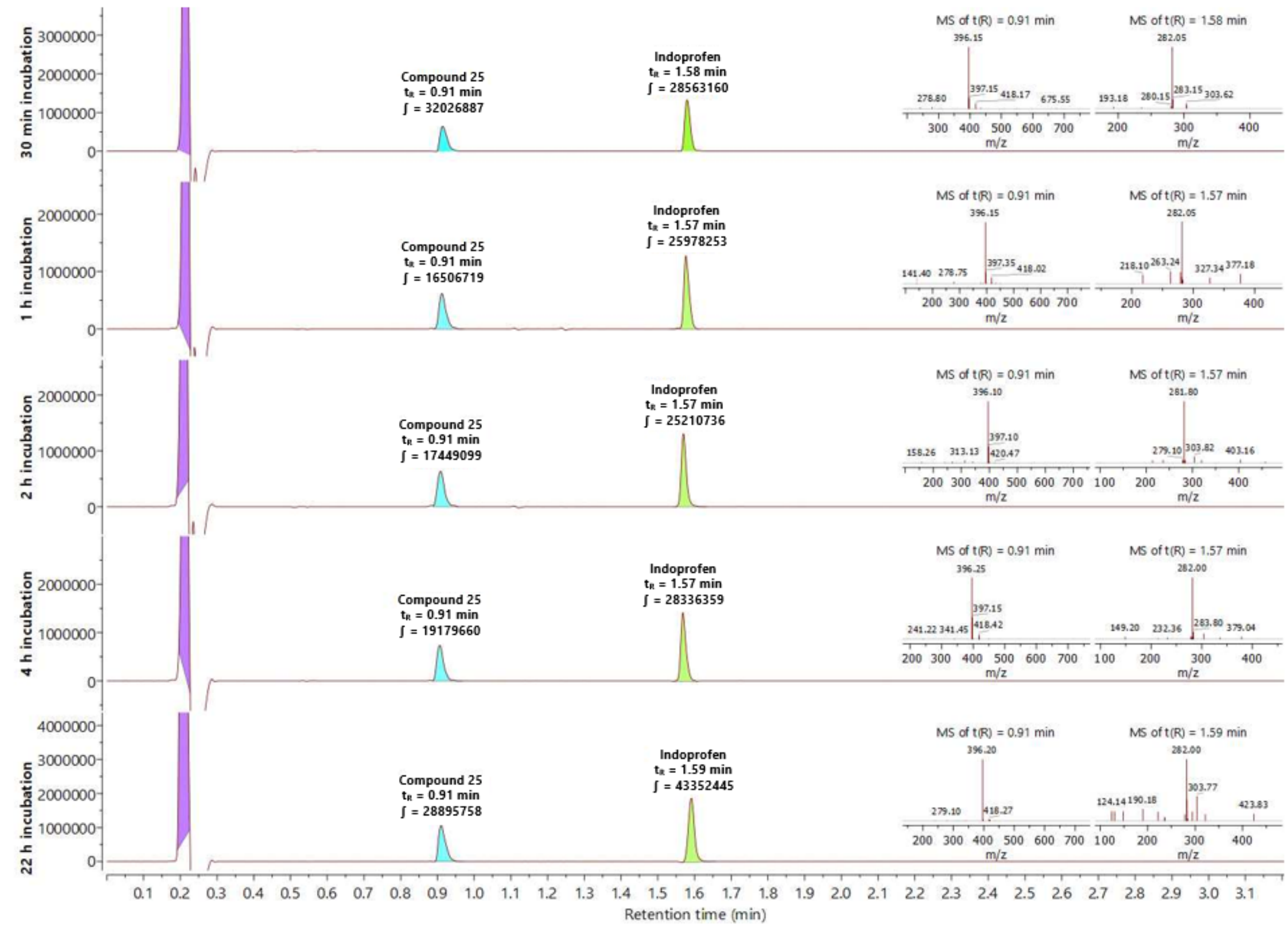

Fig S17. Incubations of L-glutathione (blueviolet), **Compound 25** (cyan; 395.25 Da), and indoprofen (chartreuse; 281.11 Da). **Compound 25** is stable under the assay conditions for >22 h.

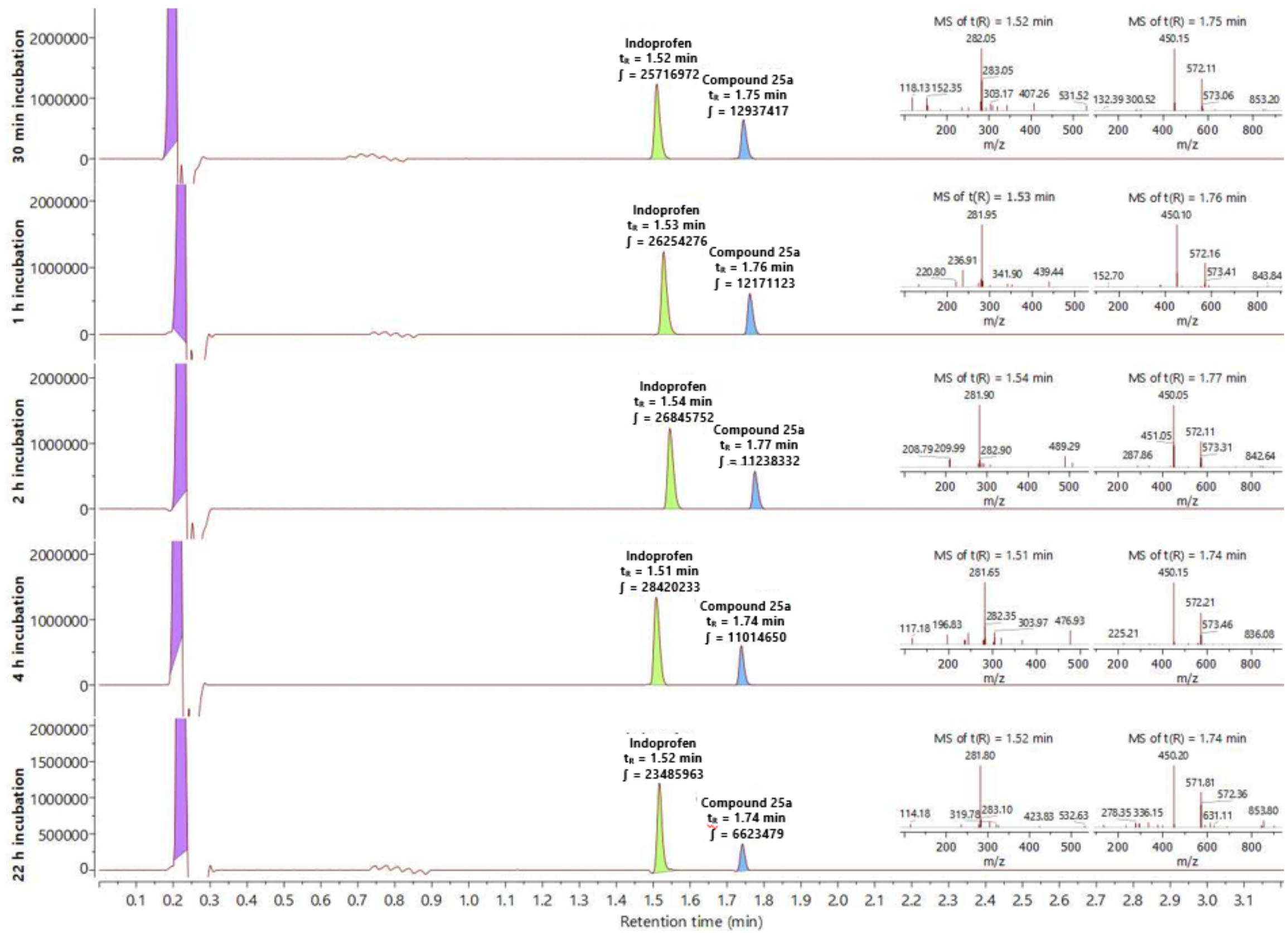

Fig S18. Incubations of L-glutathione (blueviolet), **Compound 25a** (light blue; 549.28 Da), and indoprofen (chartreuse; 281.11 Da). **Compound 25a** is stable under the assay conditions for >22 h. Note that the observed masses of 449.22 Da and 572.27 Da likely correspond to **Compound 25a** without the Boc-group and the **Compound 25a**:Na^+ adduct, respectively.

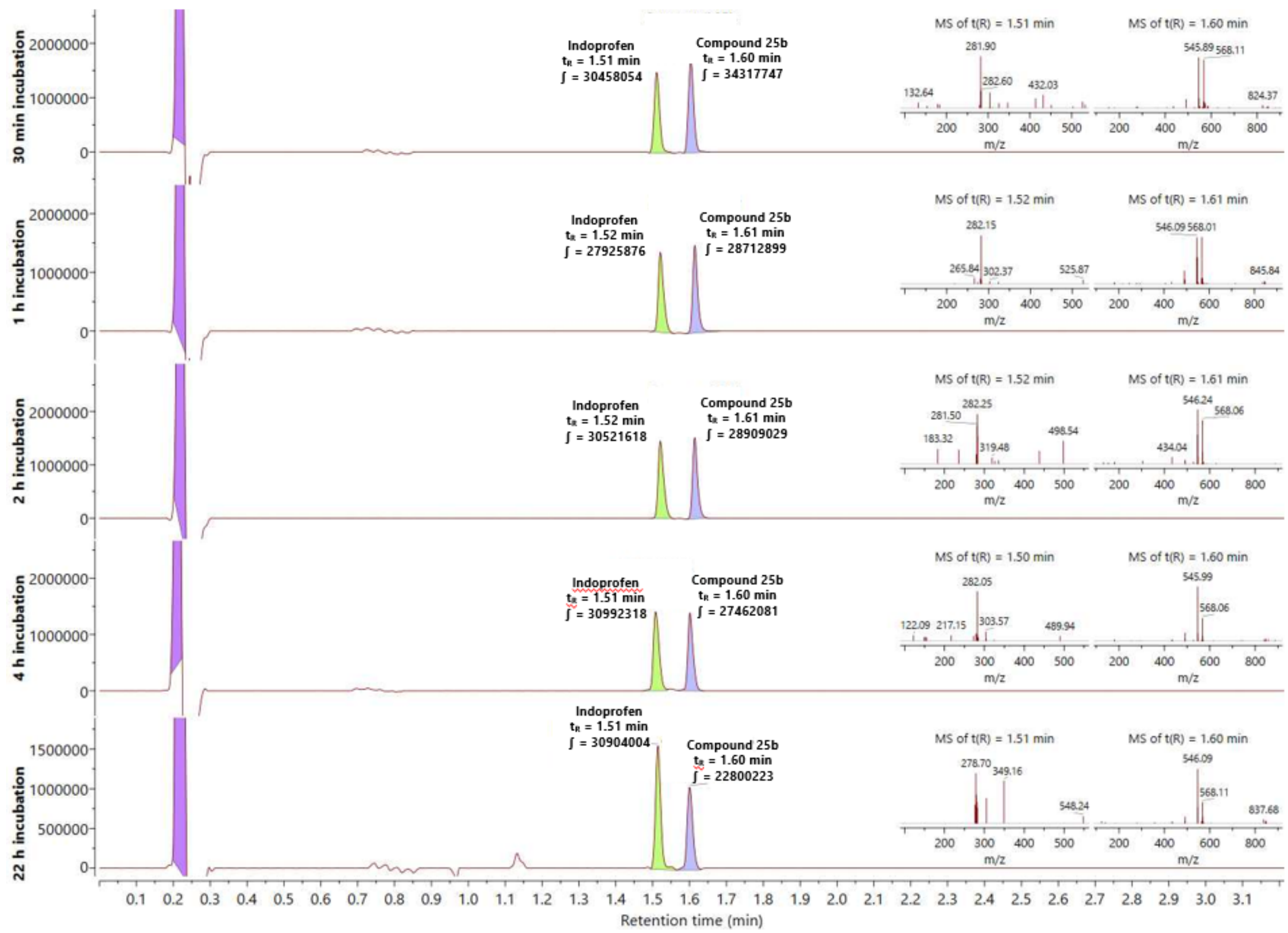

Fig S19. Incubations of L-glutathione (blueviolet), **Compound 25b** (light purple; 545.21 Da), and indoprofen (chartreuse; 281.11 Da). **Compound 25b** is stable under the assay conditions for >22 h. Note that the mass of 568.20 Da likely correspond to the **Compound 25b:**Na^+ adduct.

Uncropped blots for Figure 2B

IB: USP7

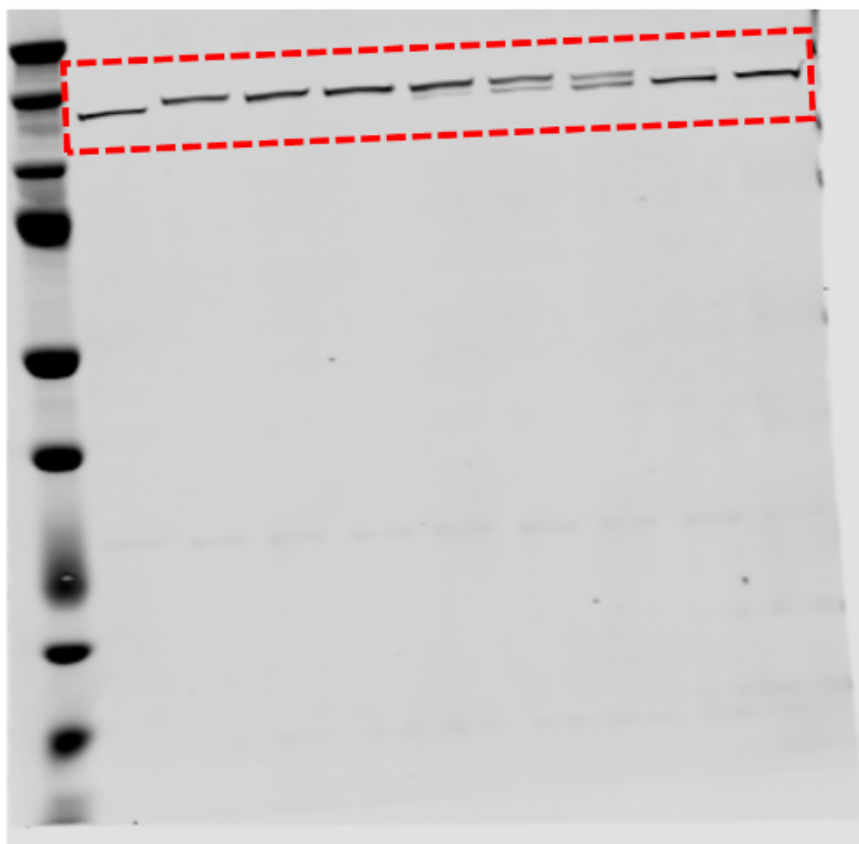

IB: β-actin

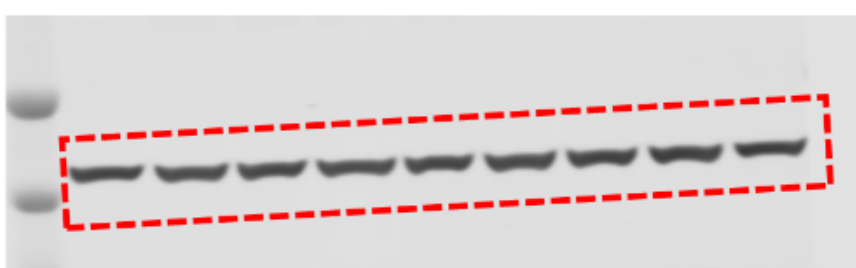

IB: OTUD7B

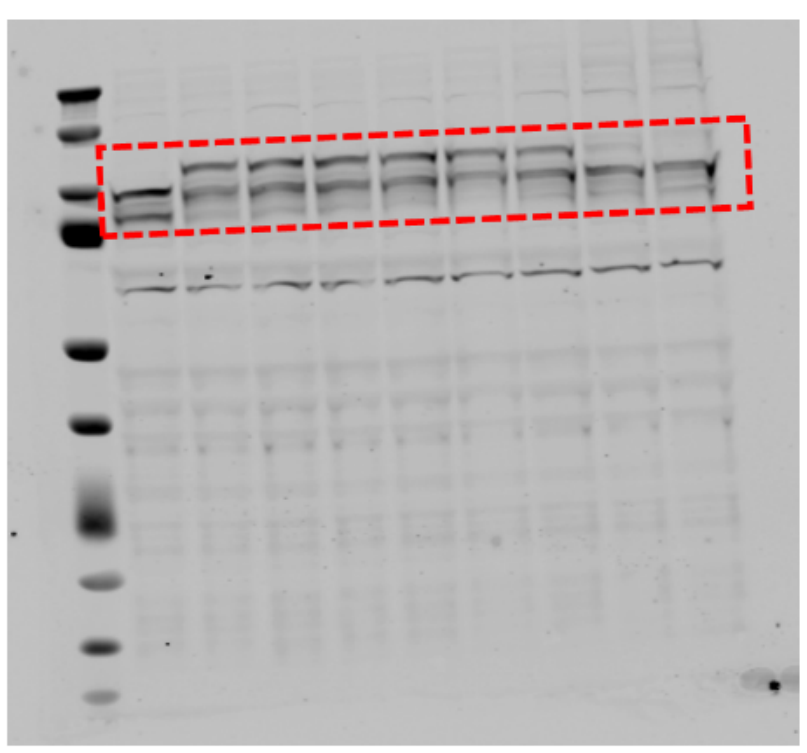

IB: β-actin

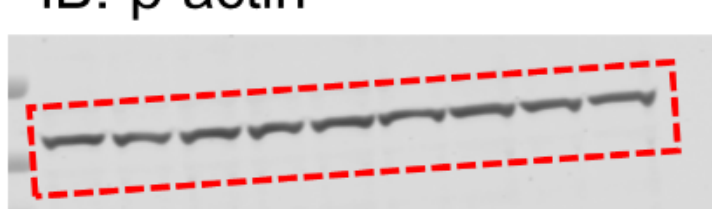

IB: USP47

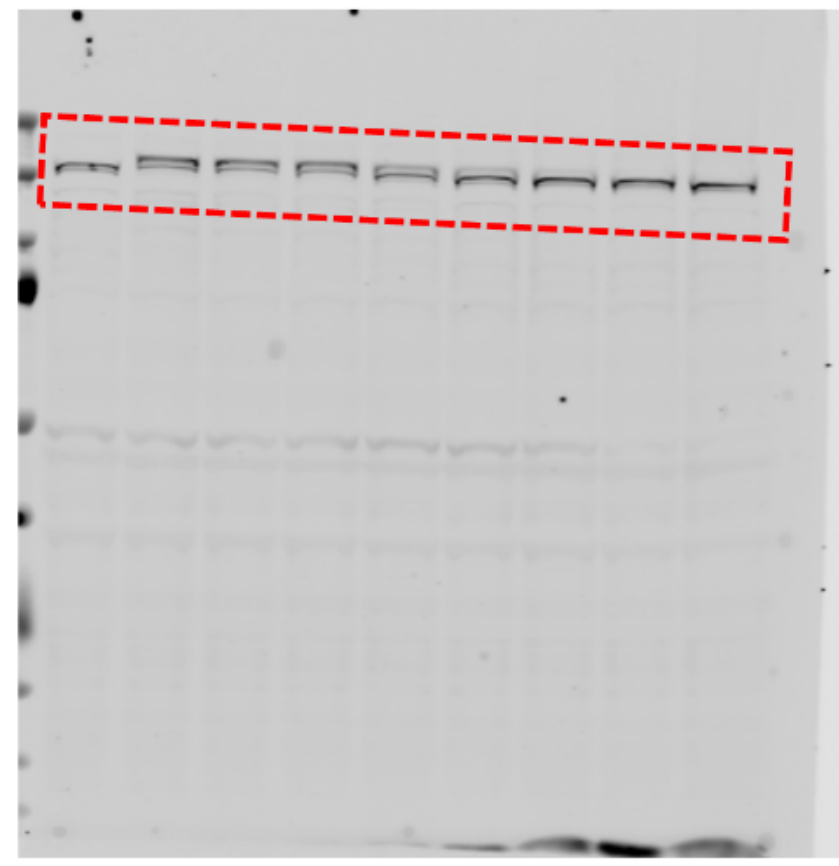

IB: β-actin

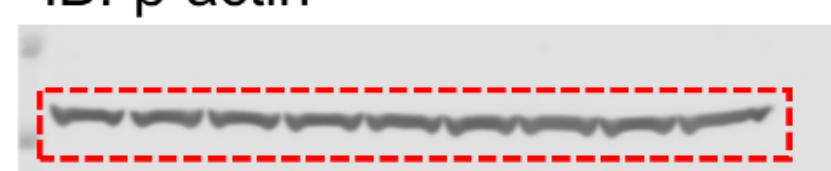

IB: USP5

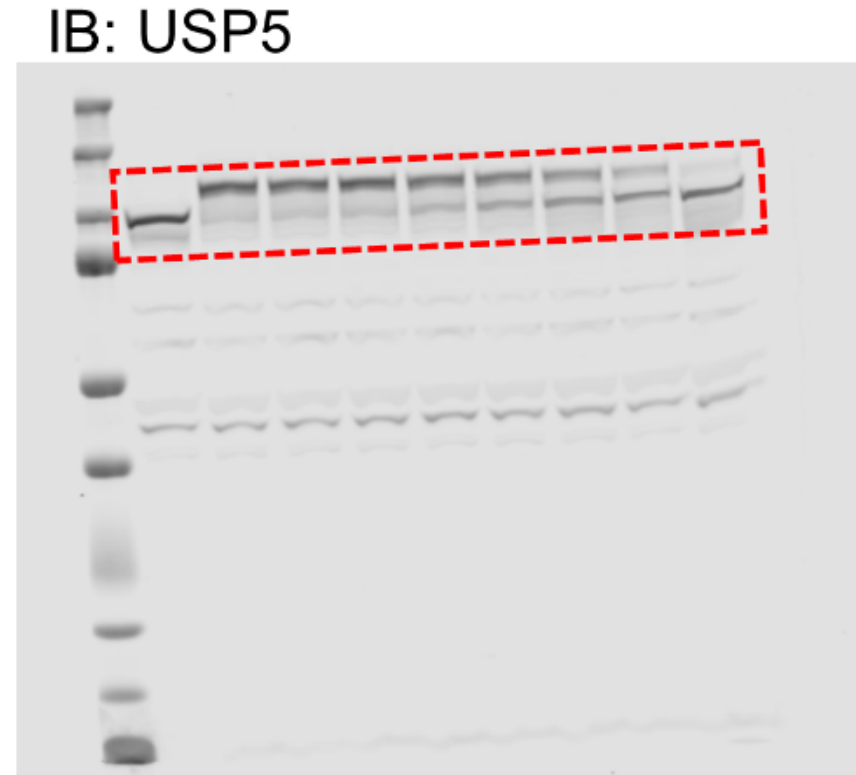

IB: β-actin

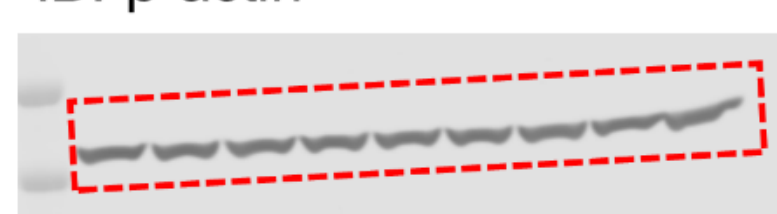

IB: immunoblot

Uncropped blots for Figure 4A

IB: USP47

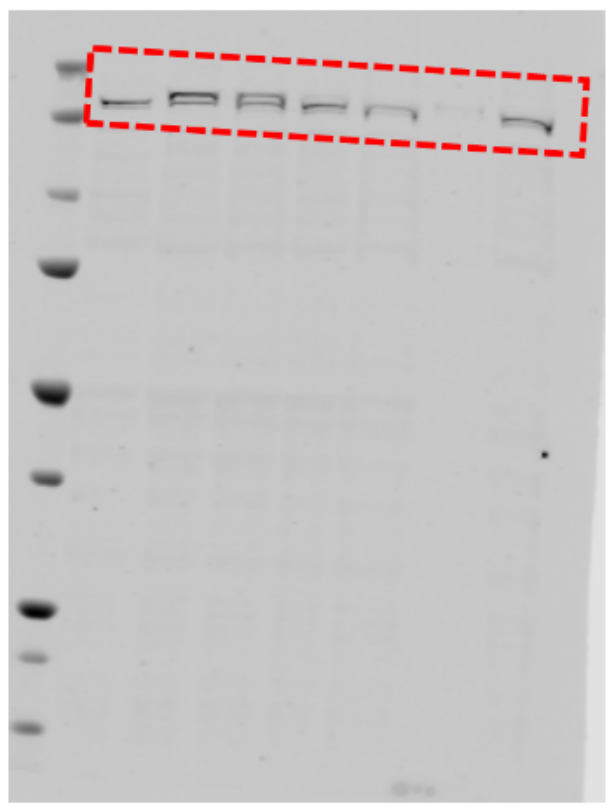

IB: USP7

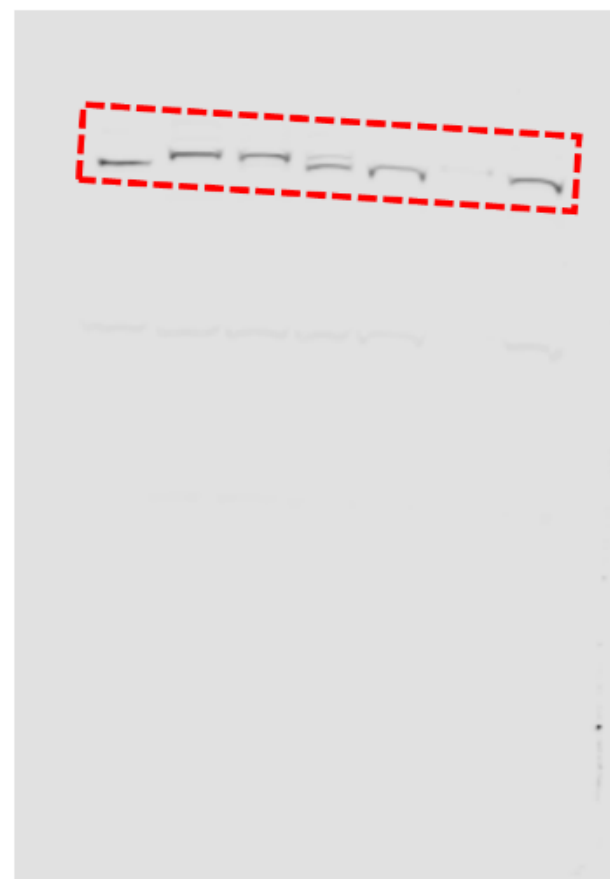

IB: CFL1

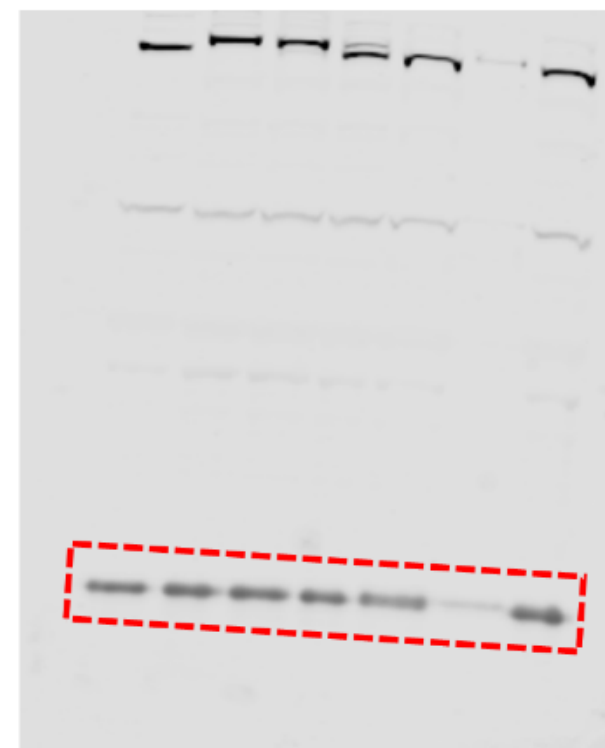

Uncropped blots for Figure 4C

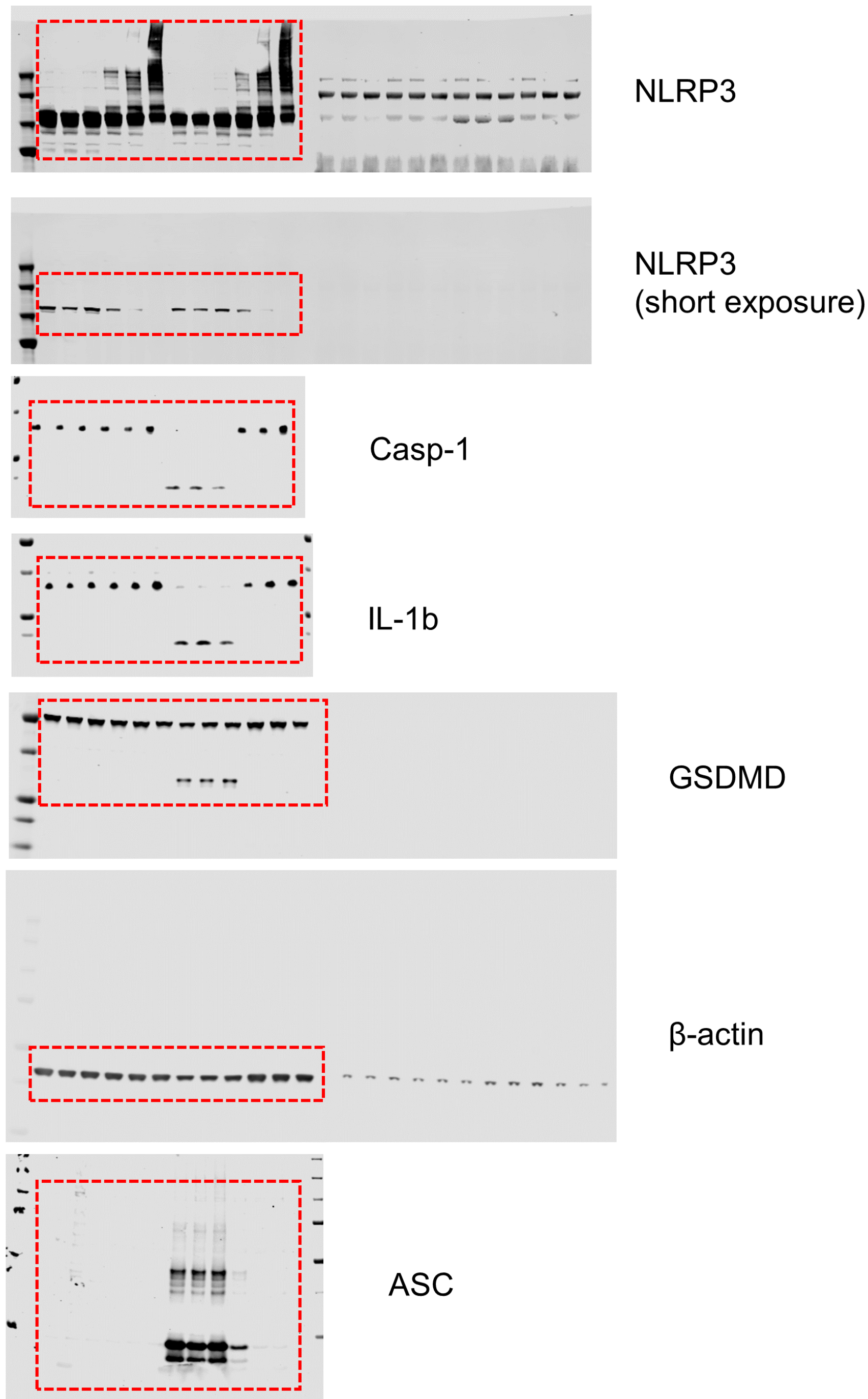

Uncropped blots for Figure 5D

IB: OTUD7B

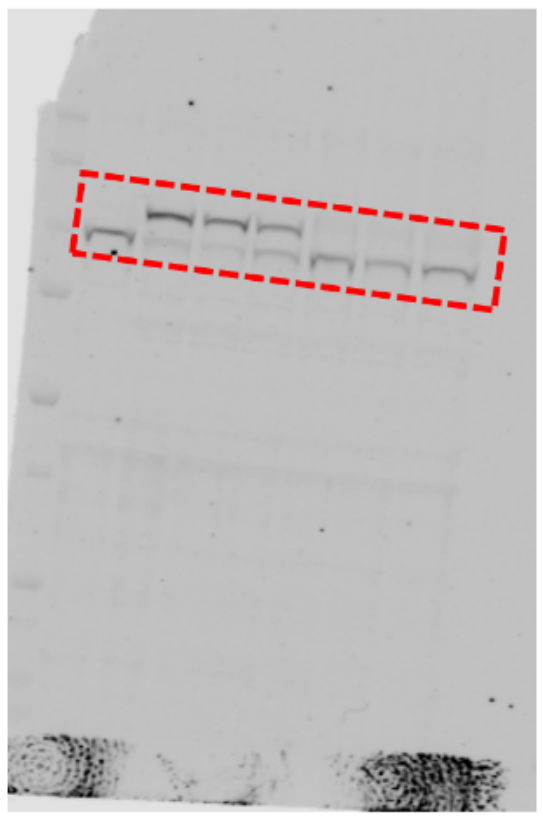

IB: CFL1

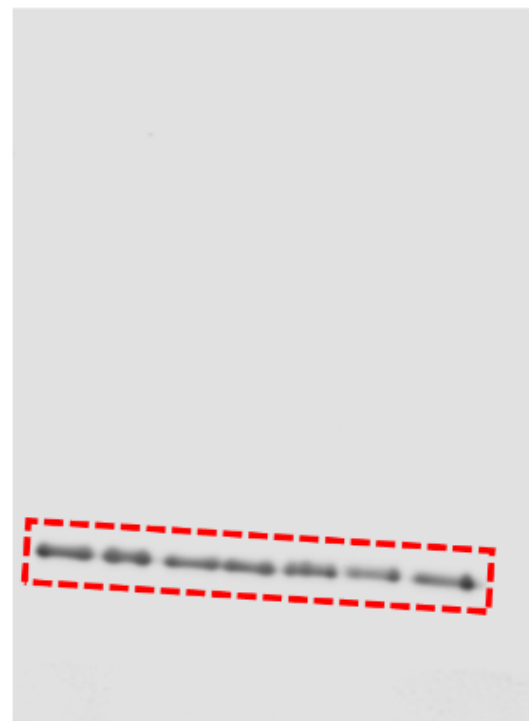

Uncropped blots for Figure 5E

IB: HIF-1α low

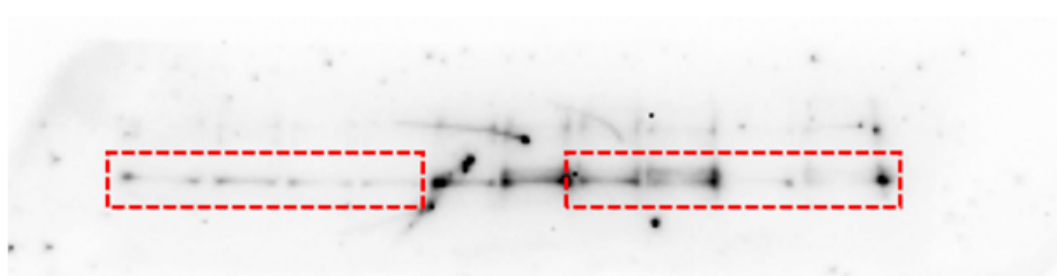

IB: β-actin

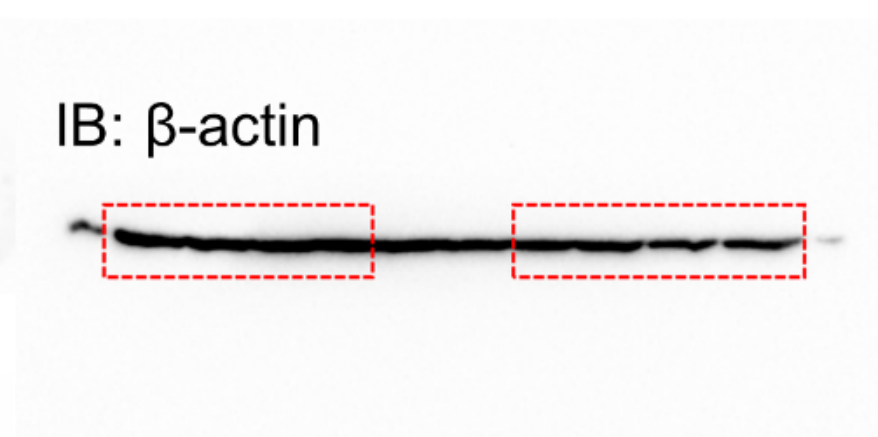

IB: HIF-1α High

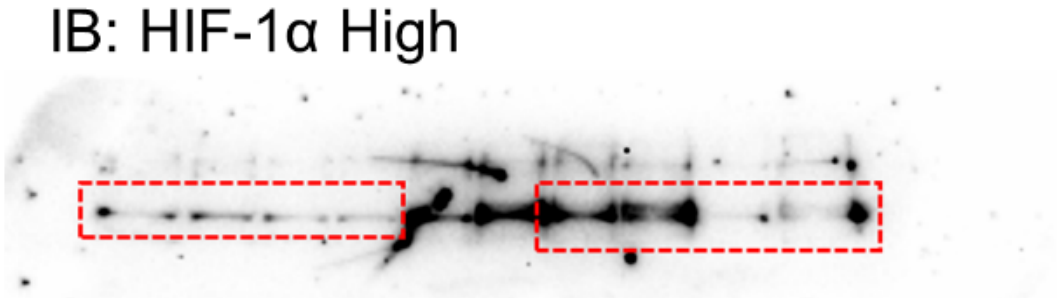

Uncropped blots for Figure 5F

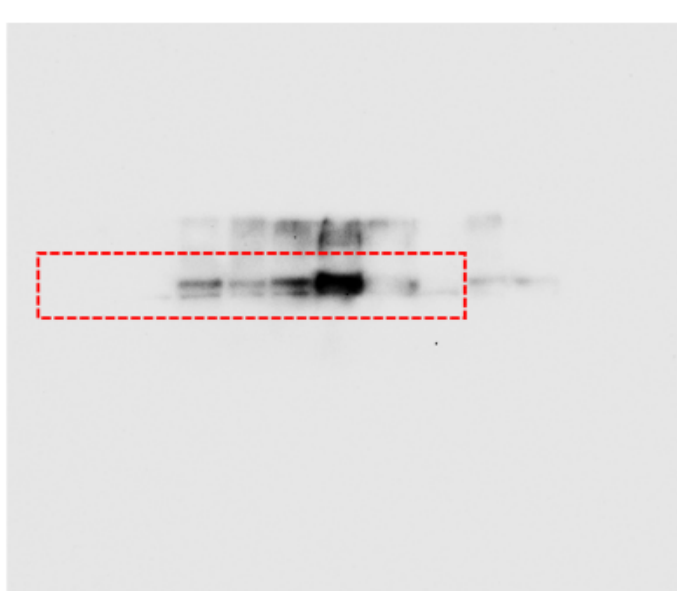

IB: HIF-1α

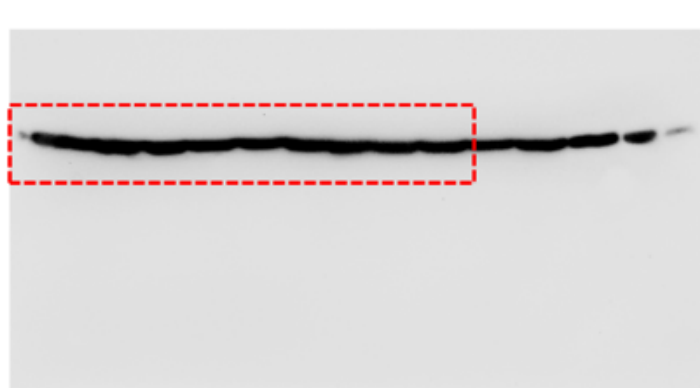

IB: β-actin

Uncropped blots for Figure 5G

IB: HIF-1α

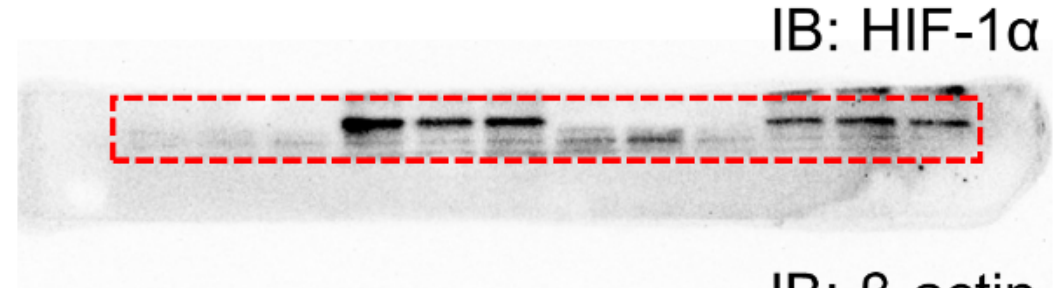

IB: β-actin

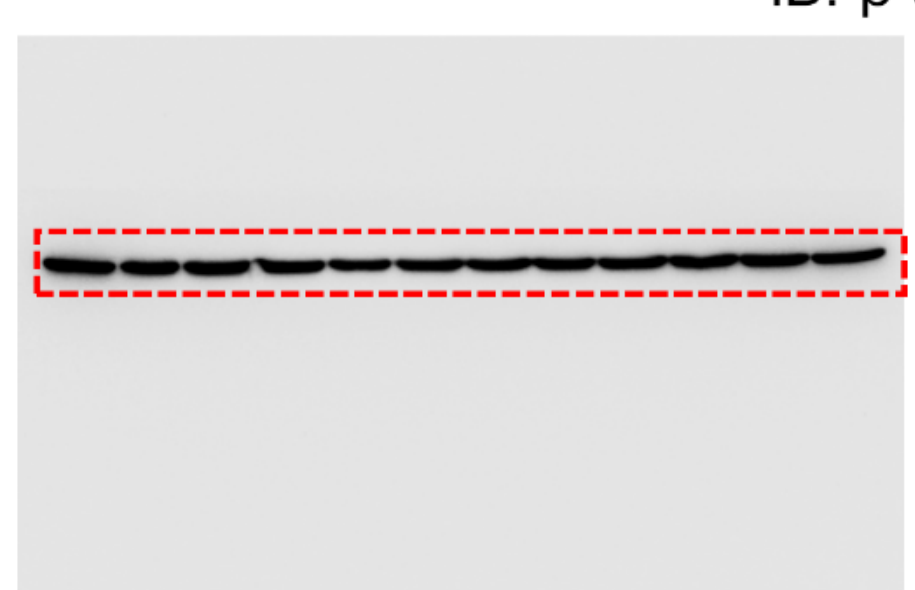

Uncropped blots for Figure 6A

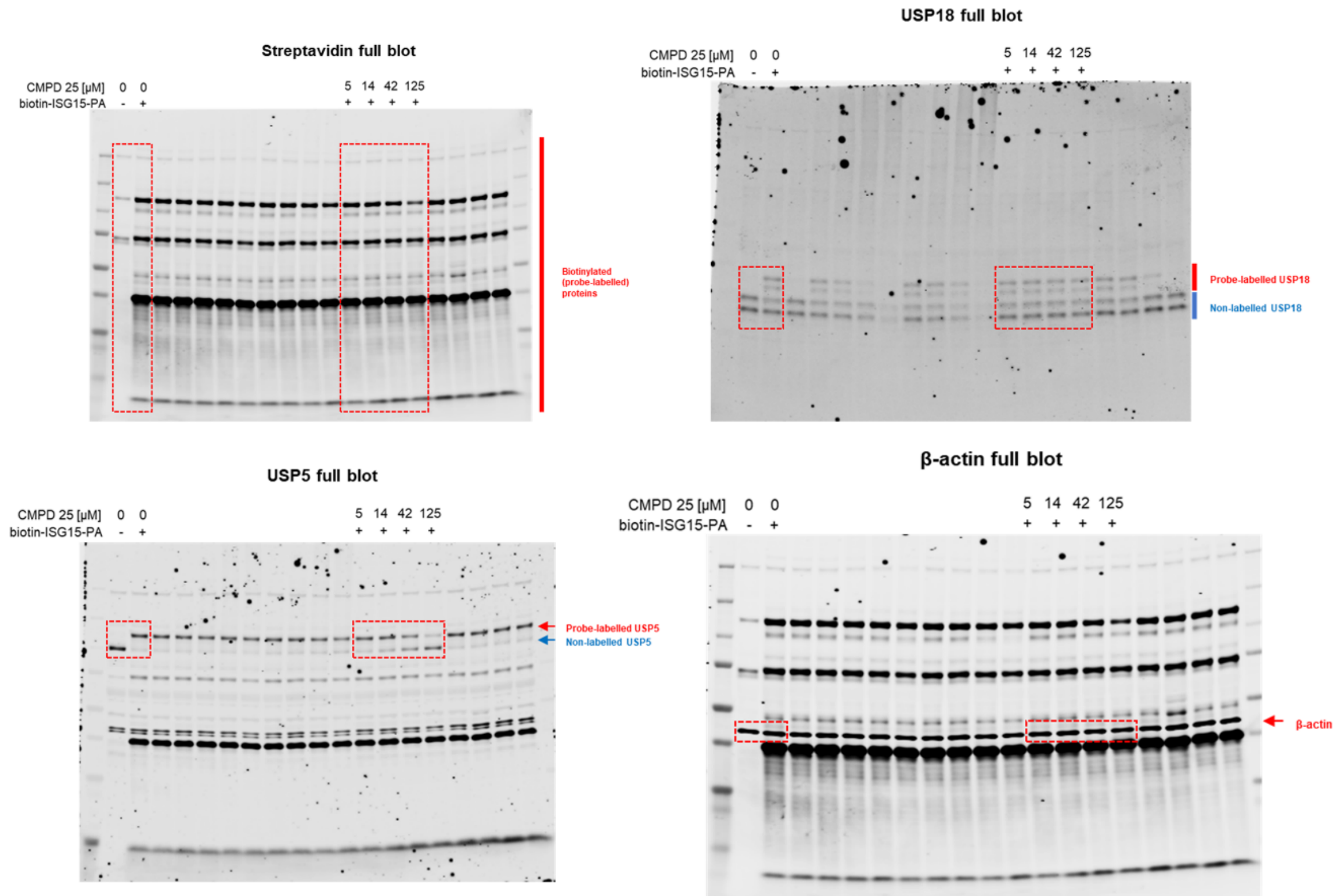

Uncropped blots for Figure 6B

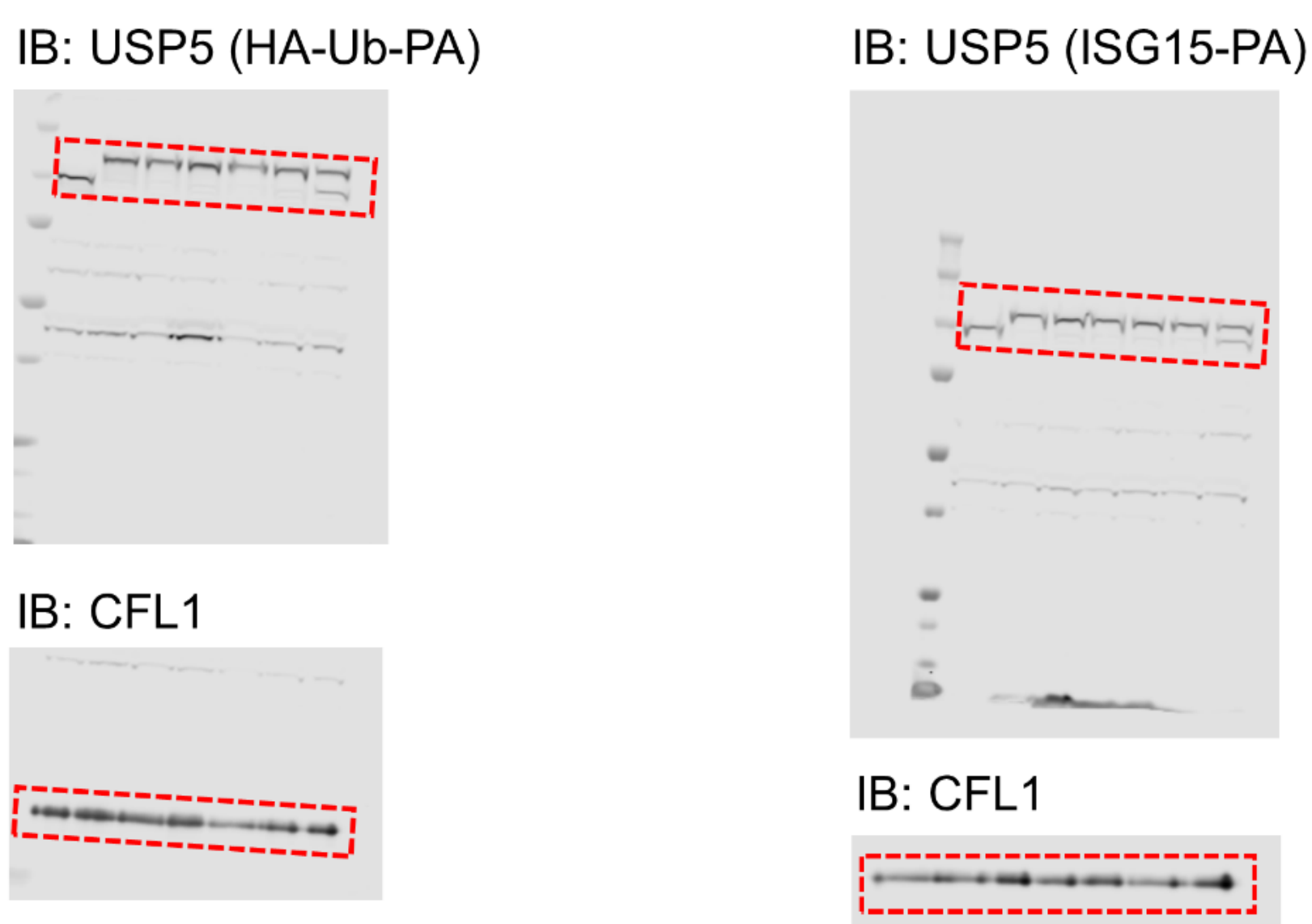

Uncropped blots for Figure 6D

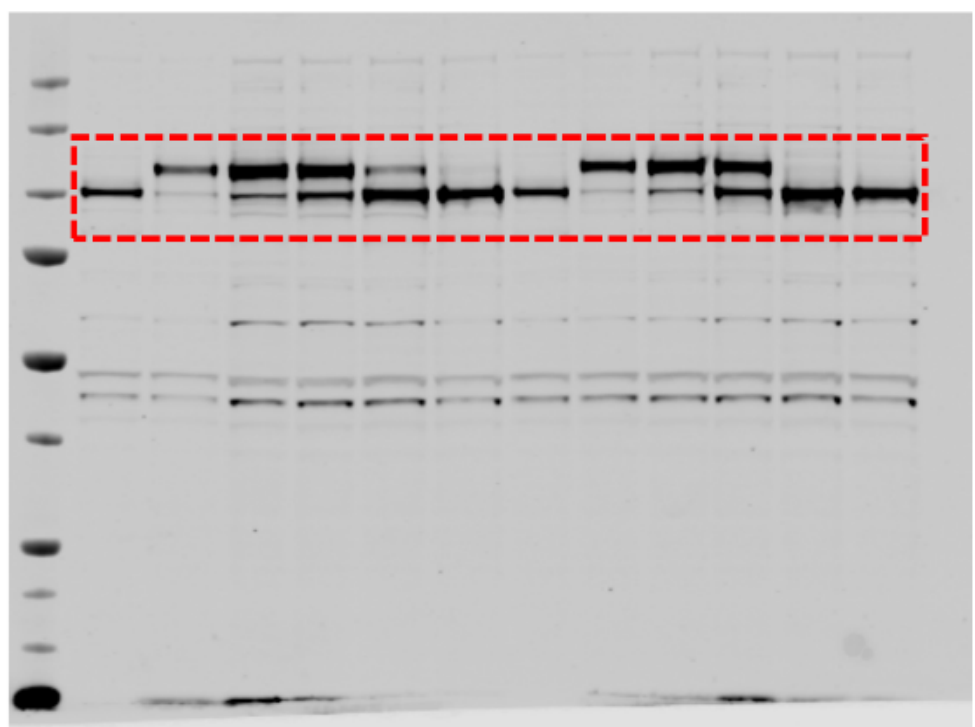

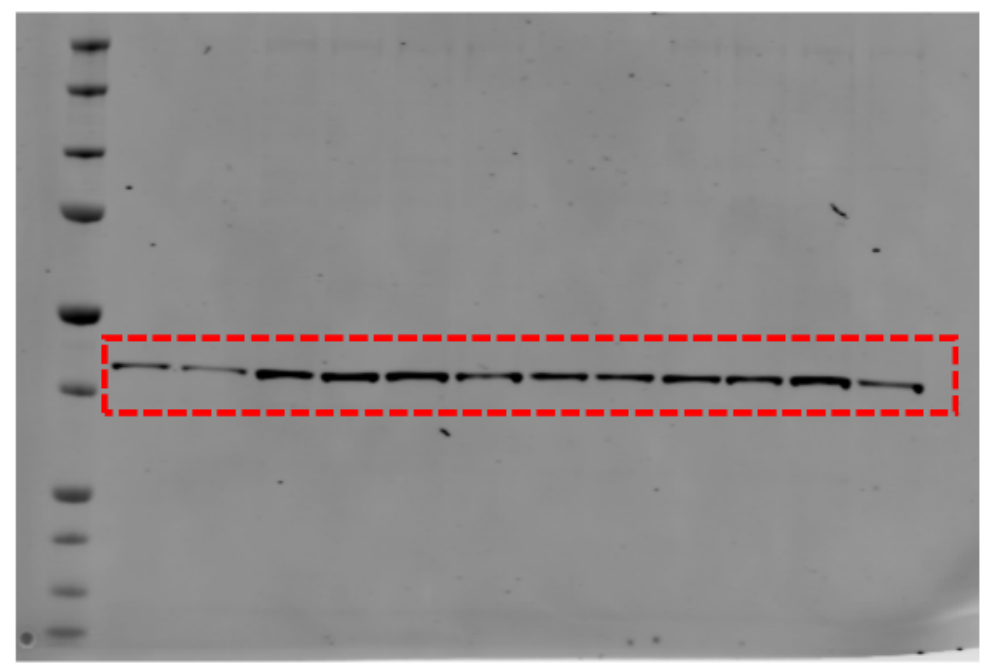

Uncropped blots for Figure 6E and S7D

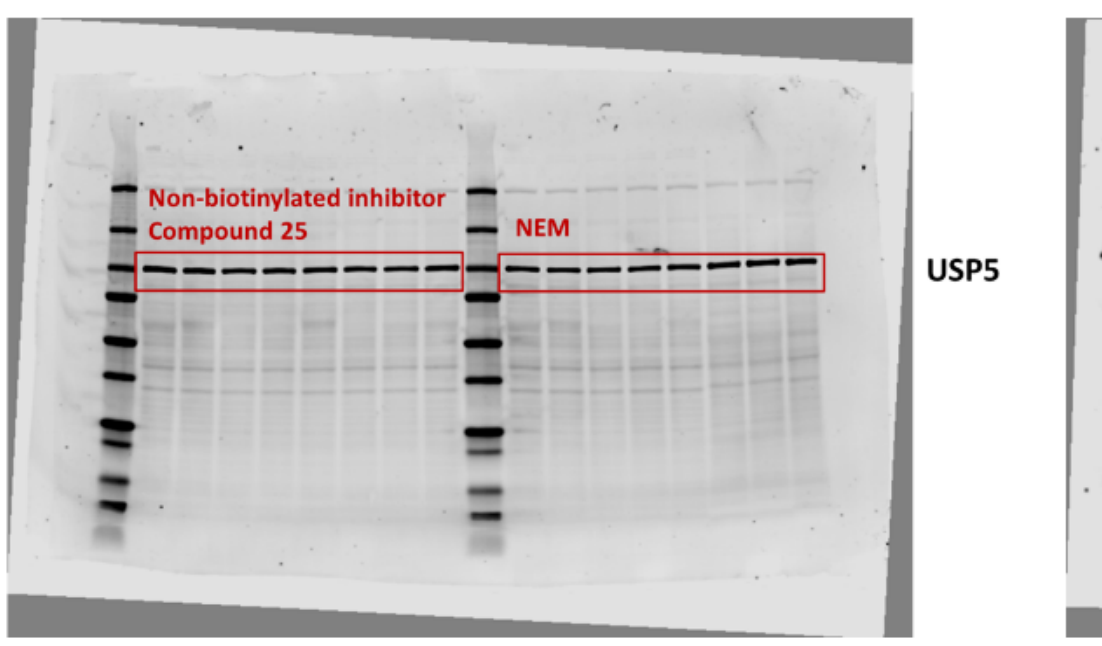

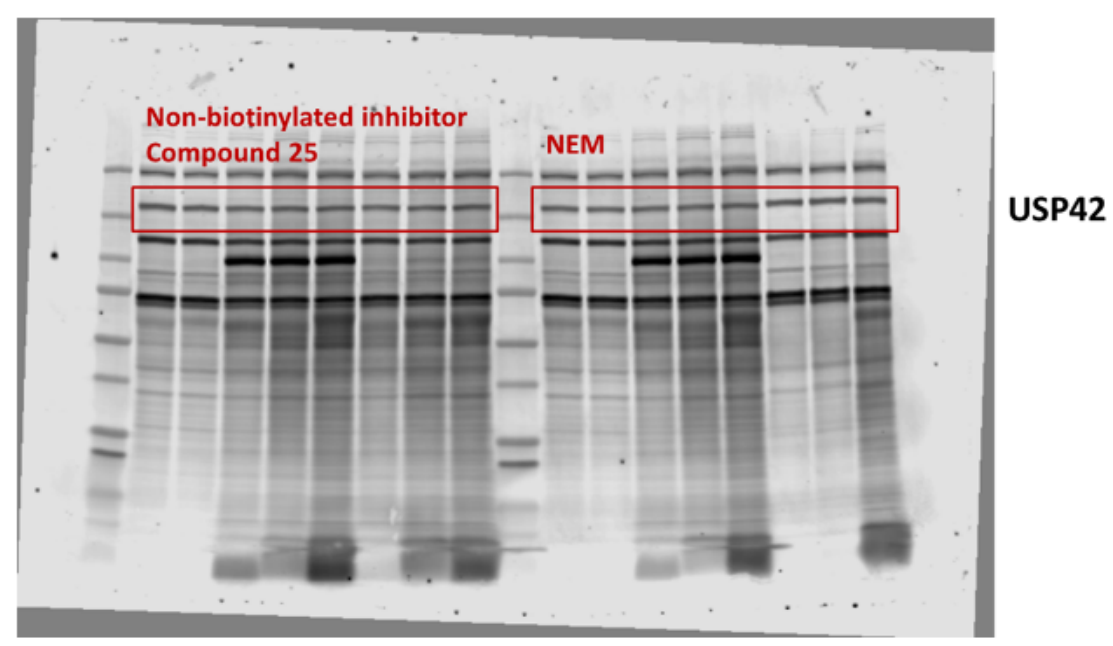

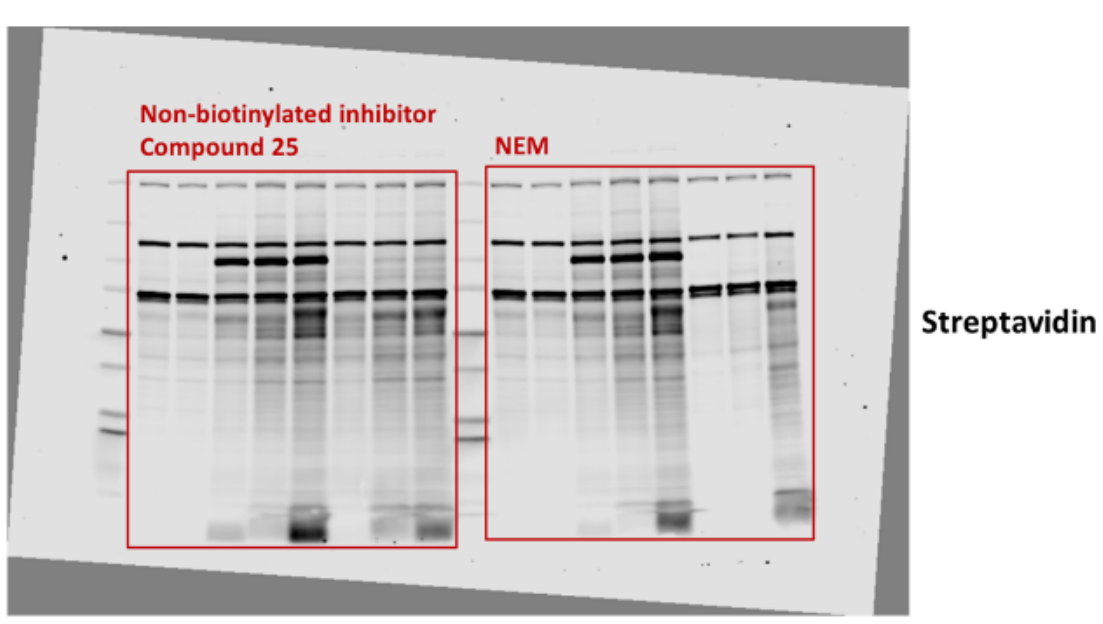

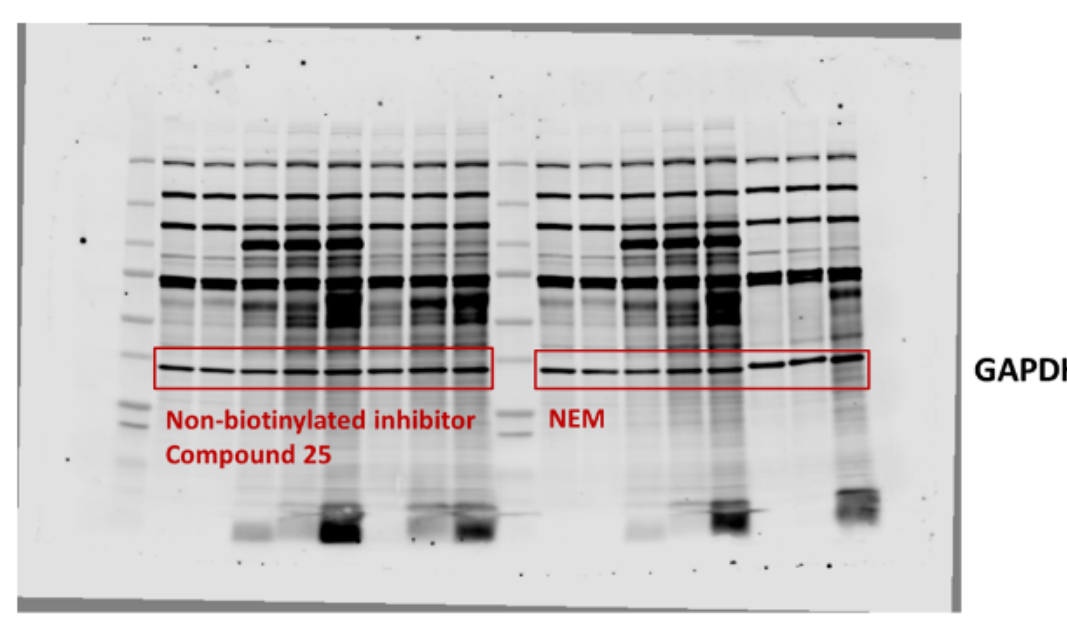

Uncropped blots for Figure S2D

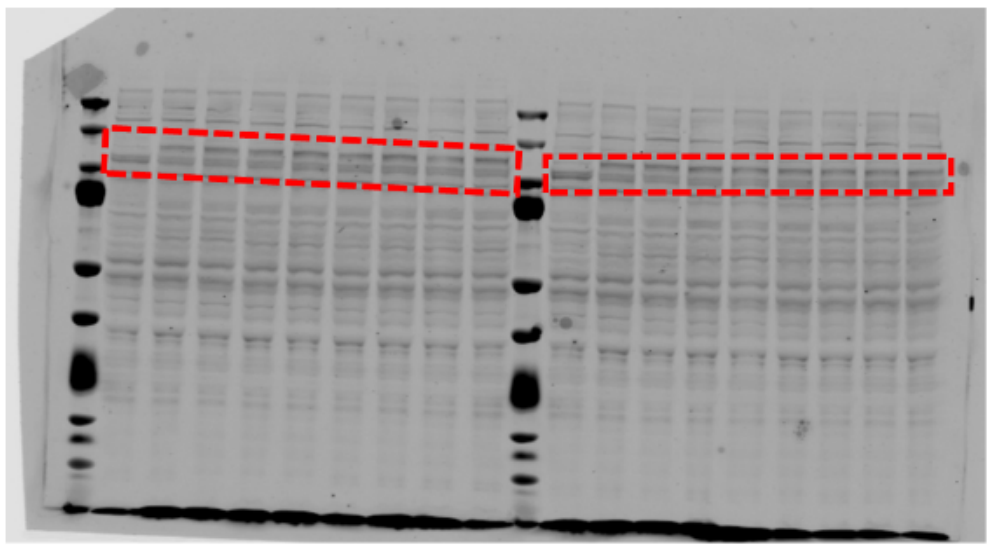

IB: USP33

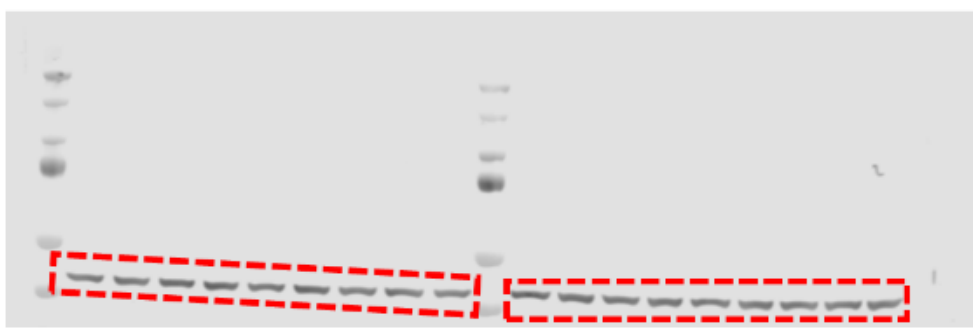

IB: CFL1

Uncropped blots for Figure S2F

IB: USP47

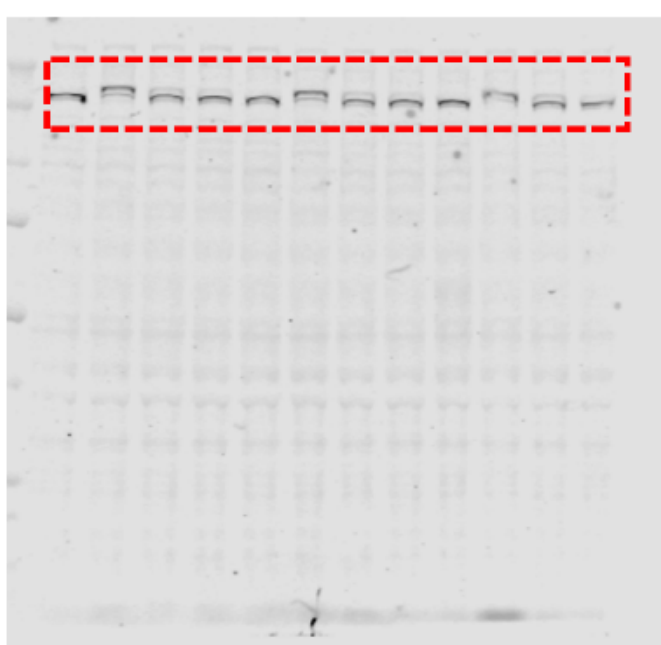

IB: USP7

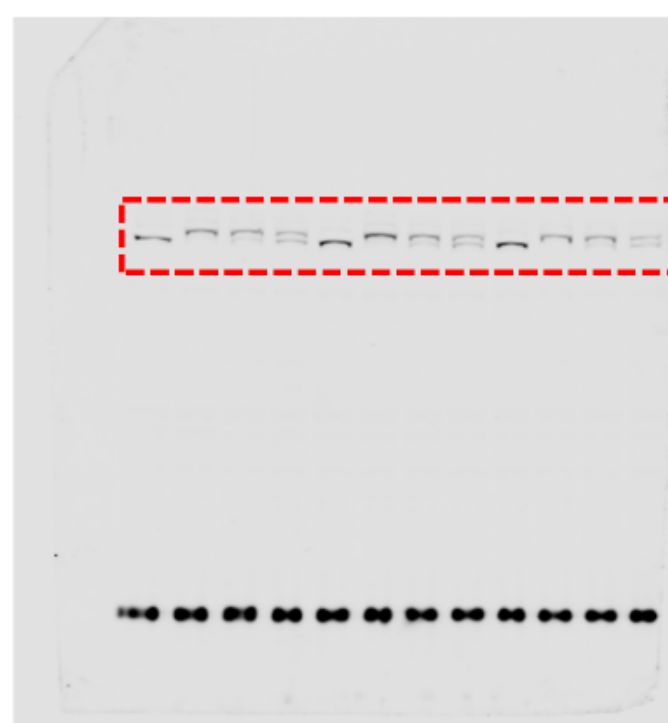

IB: CFL1

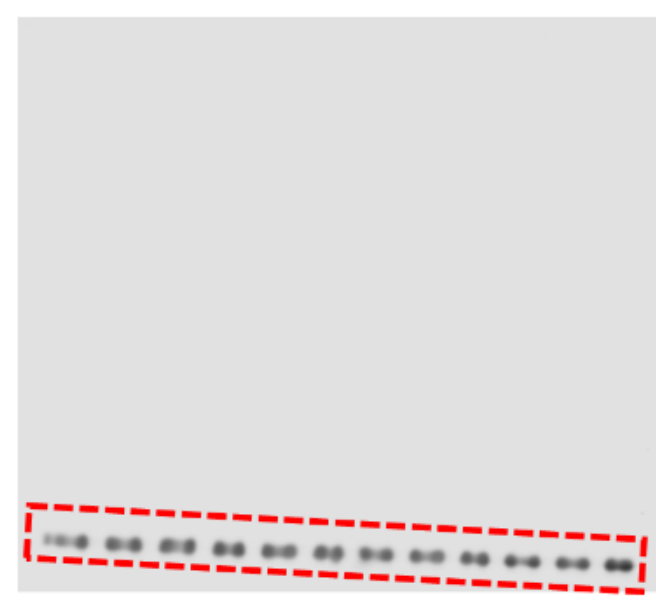

Uncropped blots for Figure S2G

IB: OTUD7B

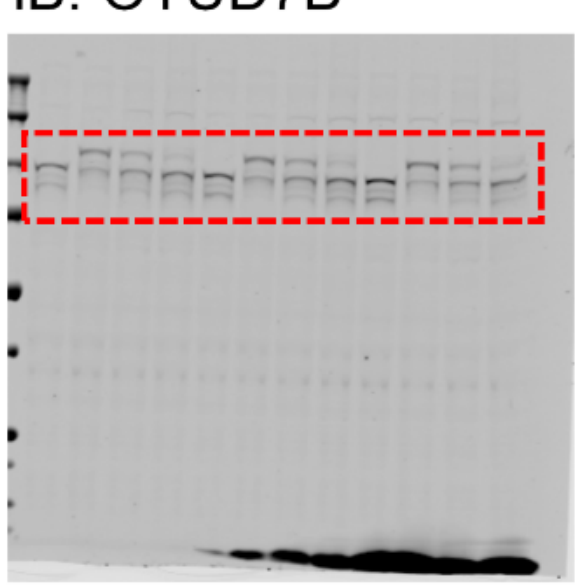

IB: CFL1

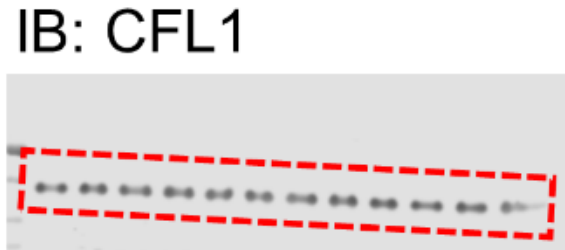

Uncropped blots for Figure S2H

IB: USP5

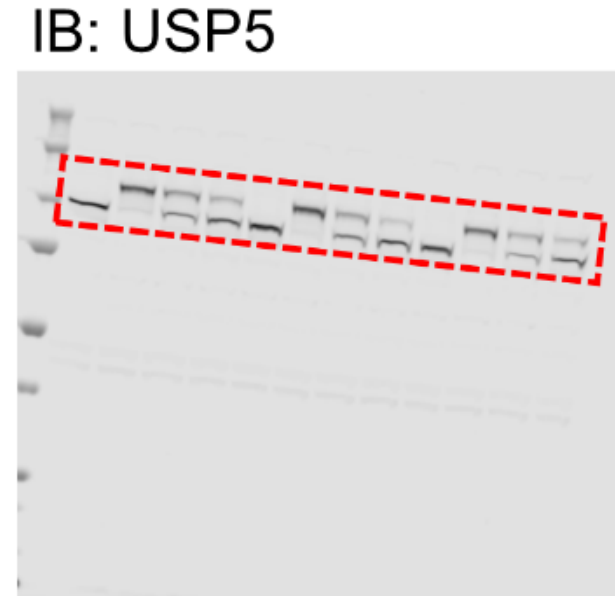

IB: CFL1

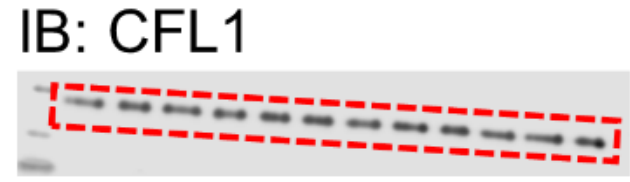

Uncropped blots for Figure S2E

IB: VCPIP1

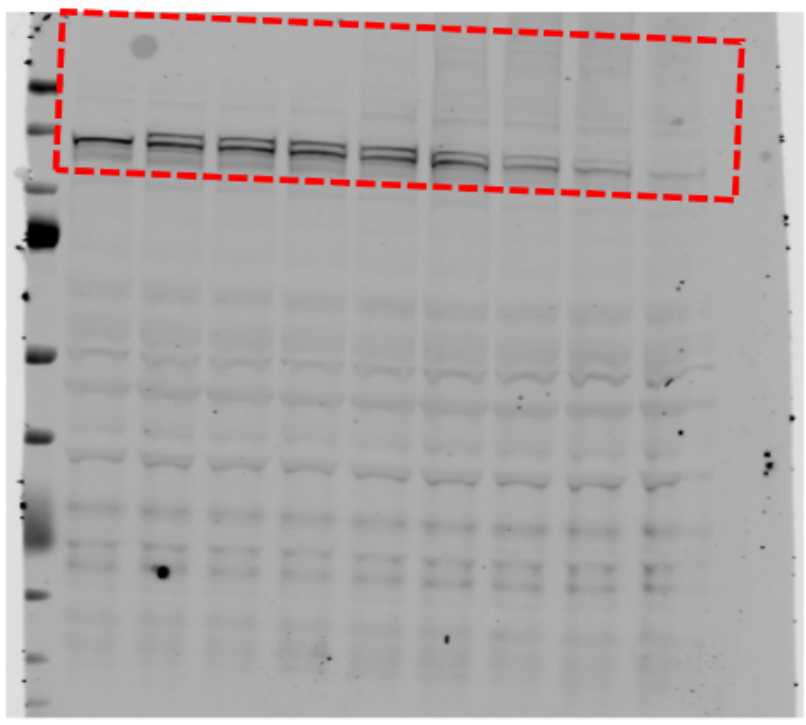

IB: β-actin

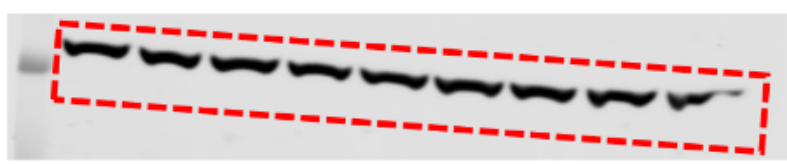

IB: USP30

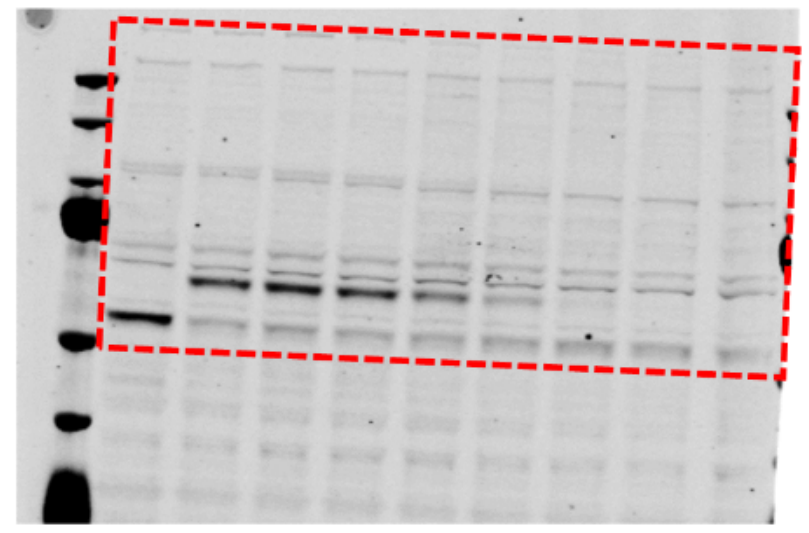

IB: β-actin

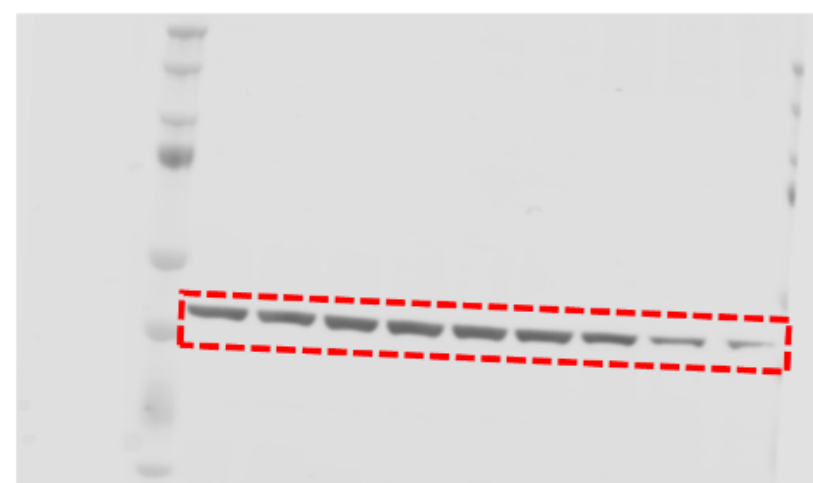

Uncropped blots for Figure S6

IB: USP5

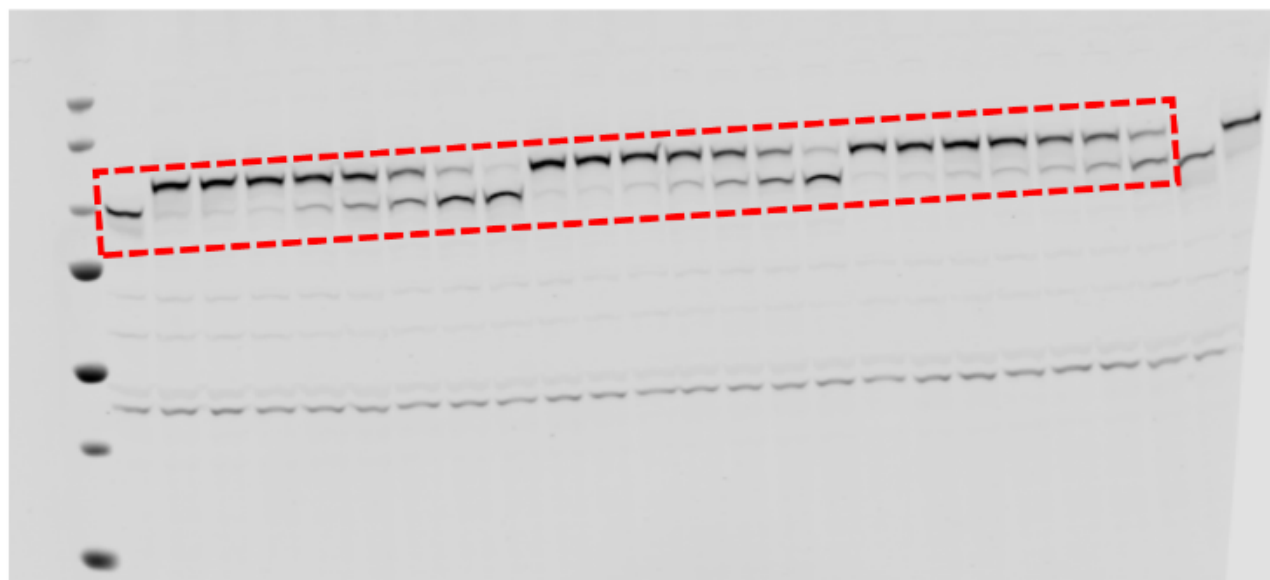

IB: Anti-HA

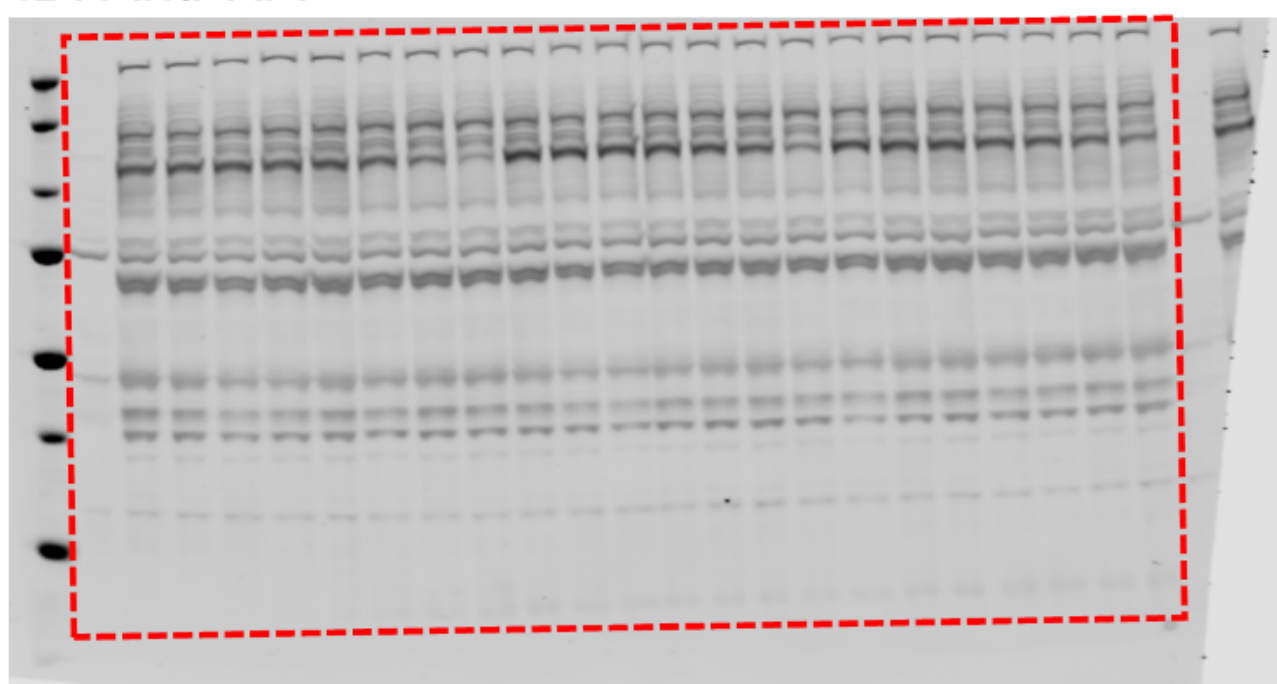

IB: β-actin

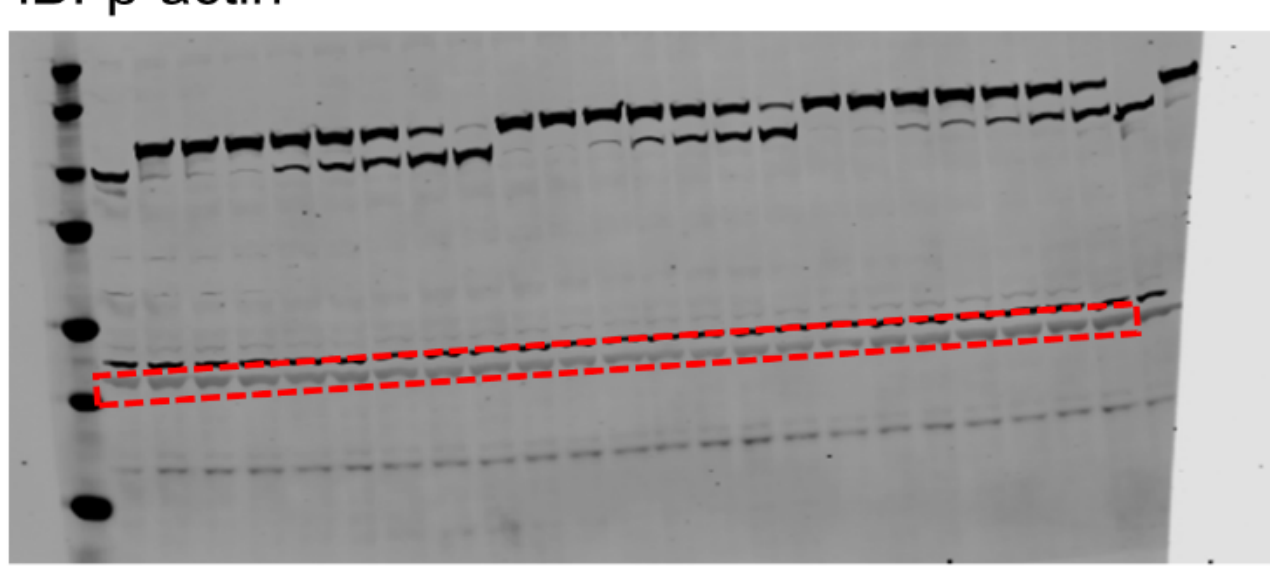

Uncropped blots for Figure S7B

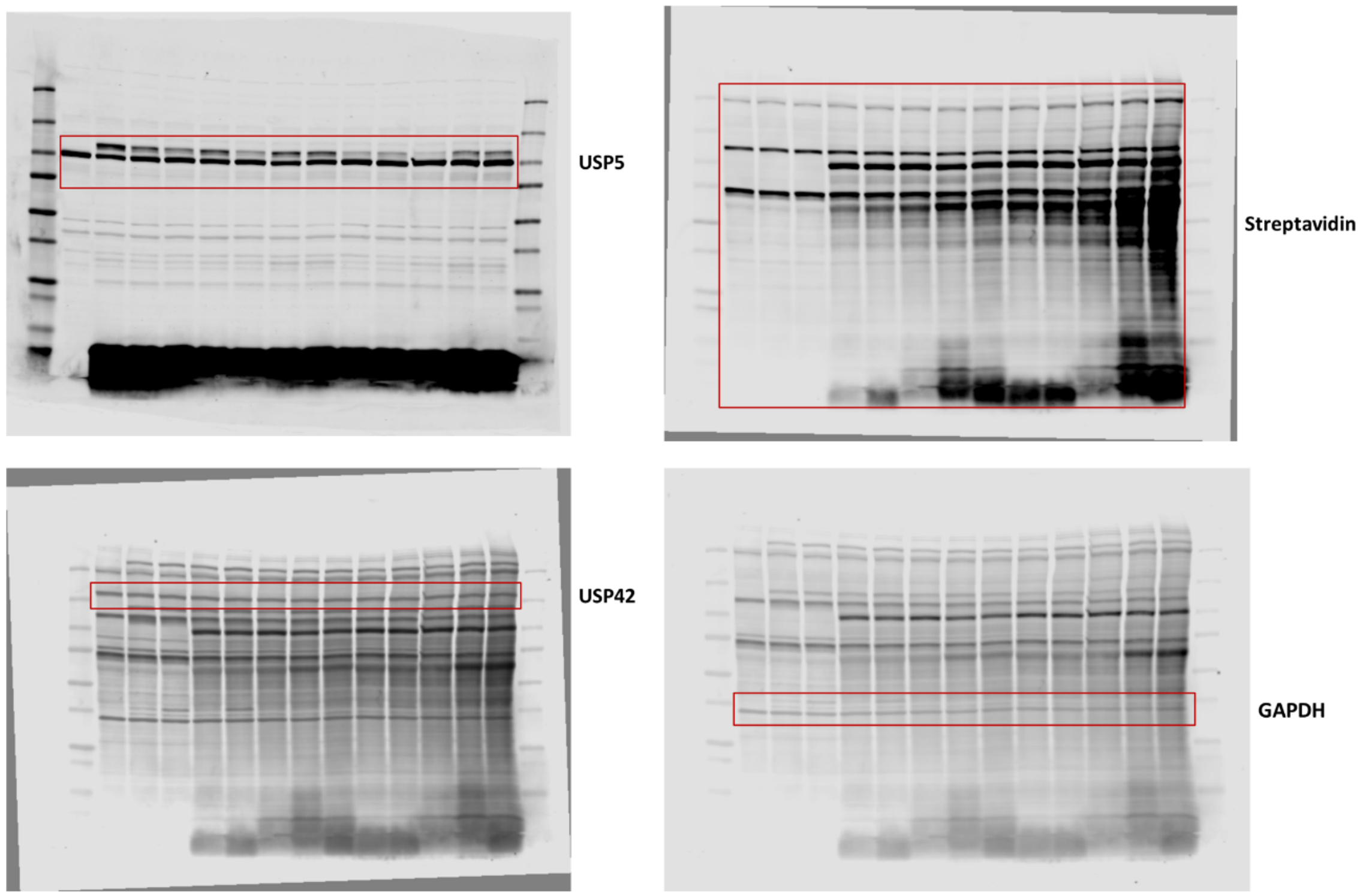

Fig S20. Uncropped and Unprocessed Gel Scans

Tables

Supplementary Data 1. Structures of reported αβ,α′β′-diepoxyketones (DEKs) tested using ABPP-HT*(9).

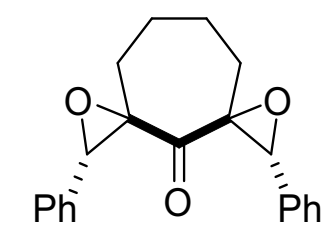<br>**1**	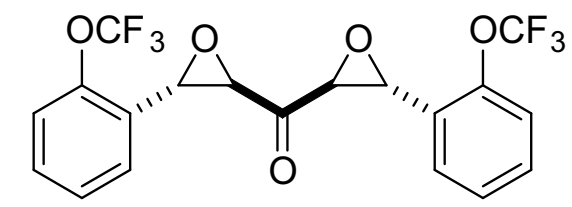<br>**5**
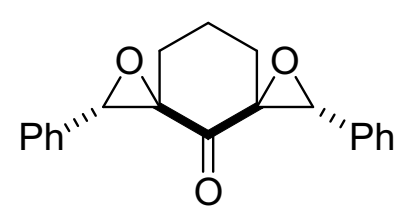<br>**2**	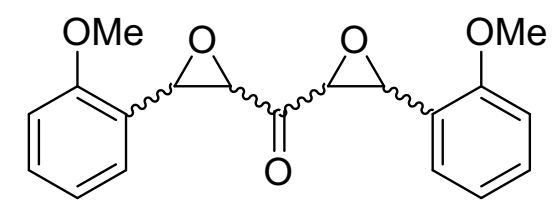<br>**6** (2:1 *trans,trans*:*cis,cis*)
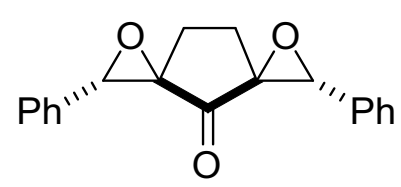<br>**3**	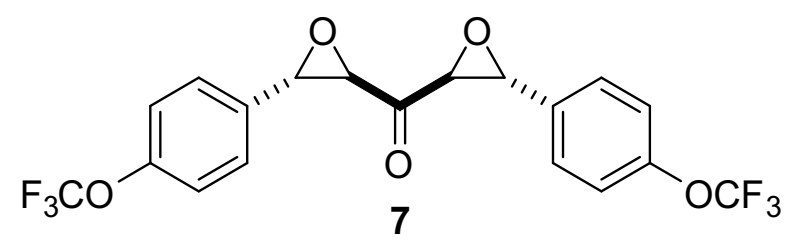<br>**7**
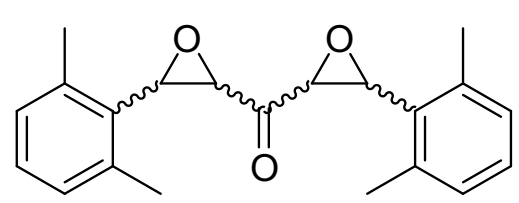<br>**4** (1.2:1 *trans,trans*:*cis,cis*)	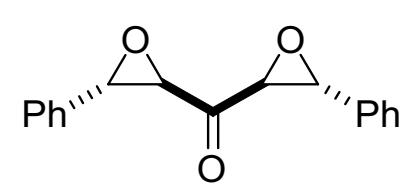<br>**8**

Supplementary Data S2. Structures of reported γ-lactams tested using ABPP-HT*(30).

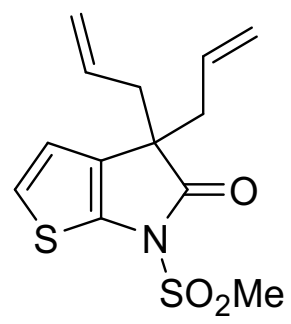 **9**	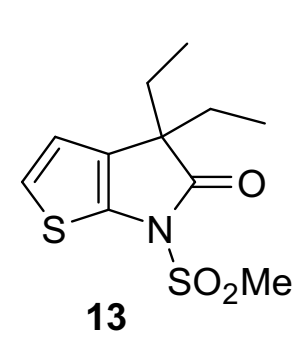 **13**	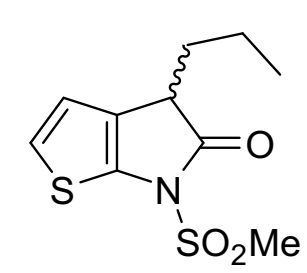 **17**	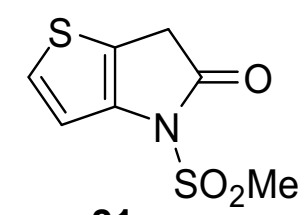 **21**
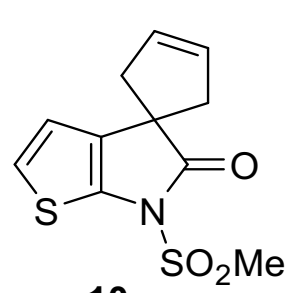 **10**	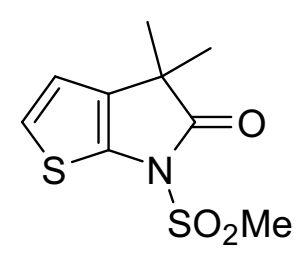 **14**	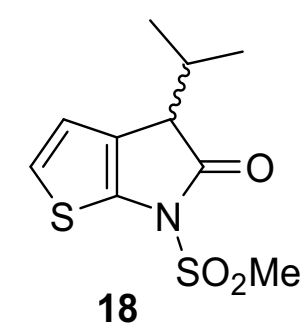 **18**	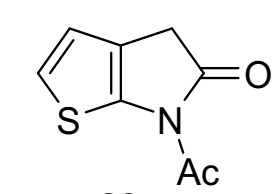 **22**
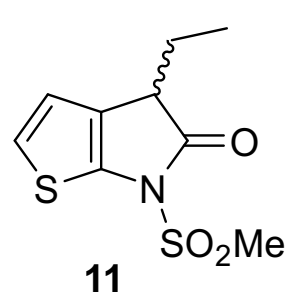 **11**	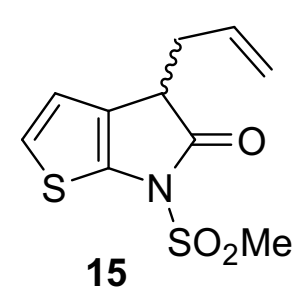 **15**	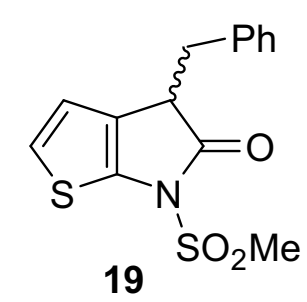 **19**	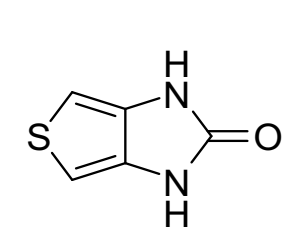 **23**
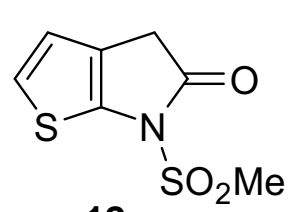 **12**	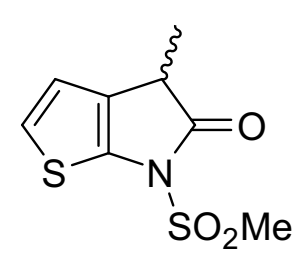 **16**	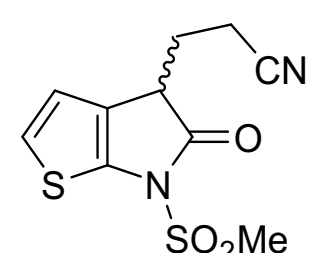 **20**	

Supplementary Data S3. Structures of Ub-derived peptides designed for this work that were tested using ABPP-HT*.

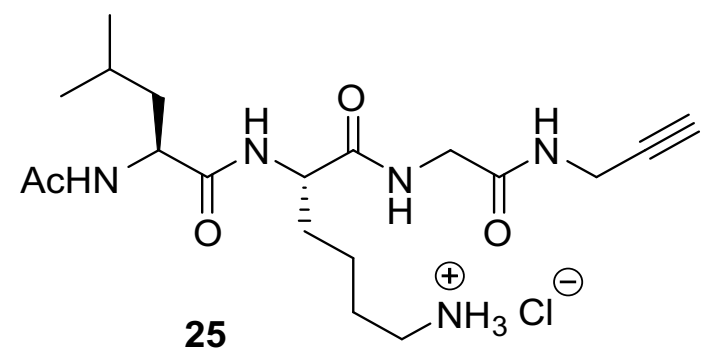 **25**	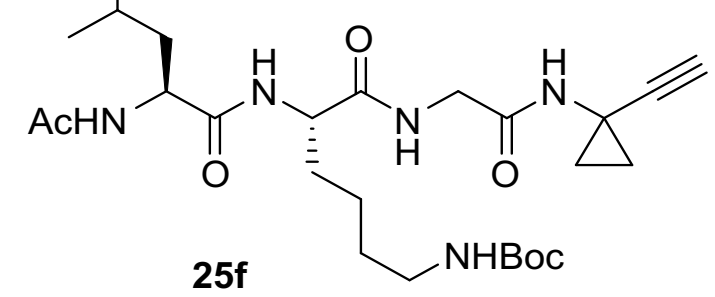 **25f**	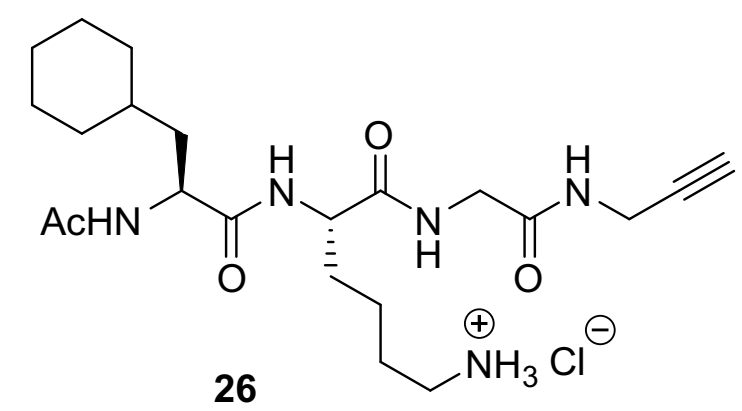 **26**
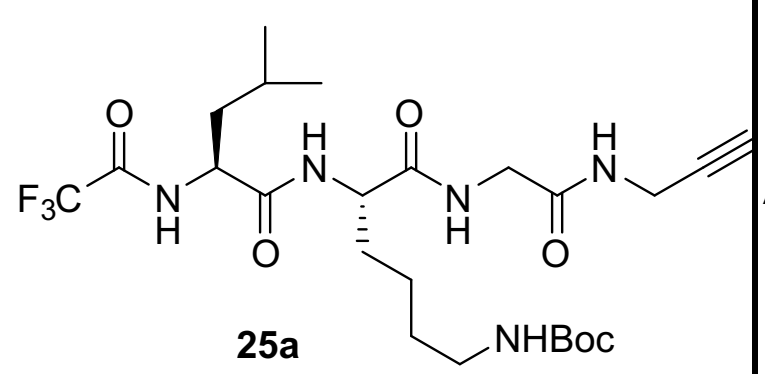 **25a**	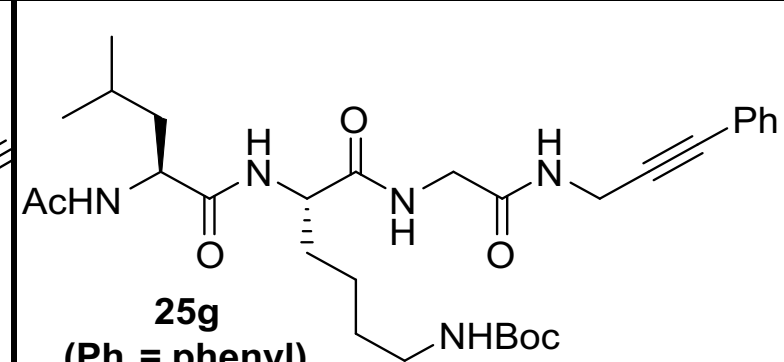 **25g** **(Ph = phenyl)**	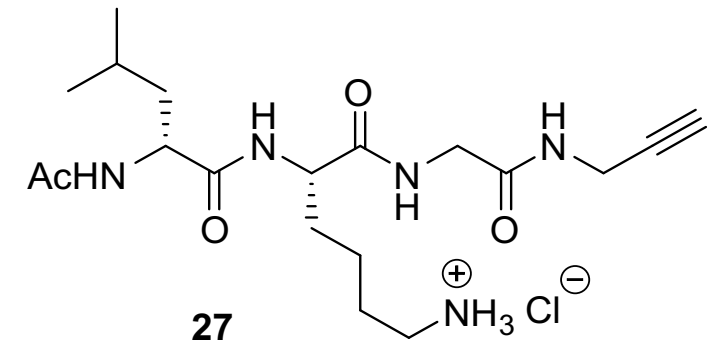 **27**
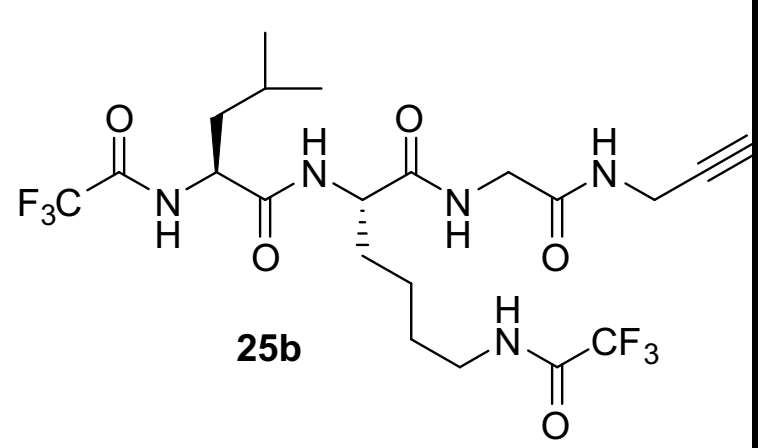 **25b**	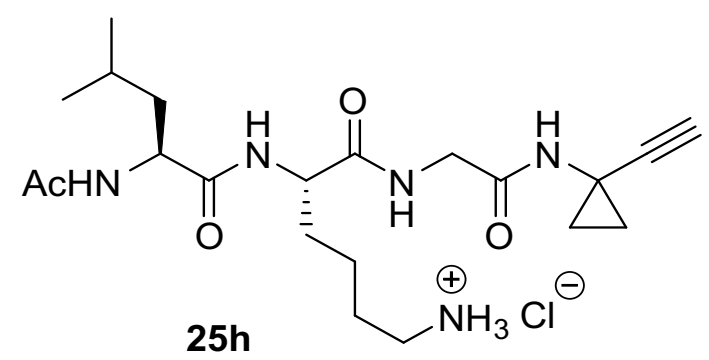 **25h**	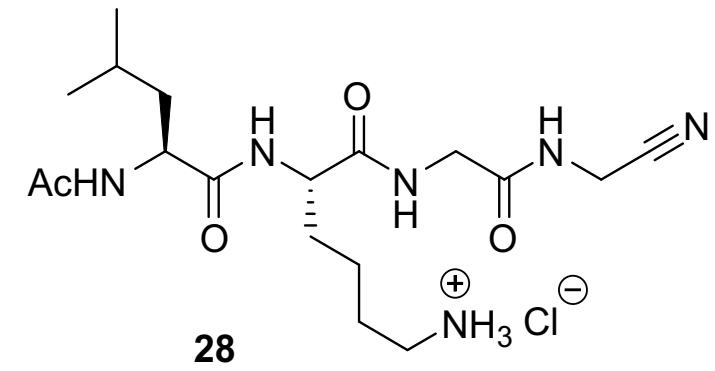 **28**
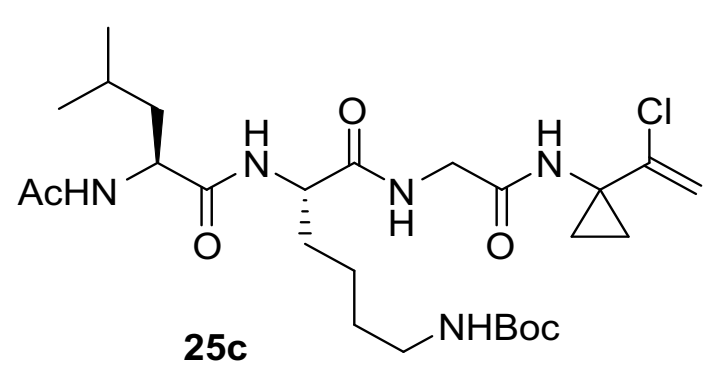 **25c**	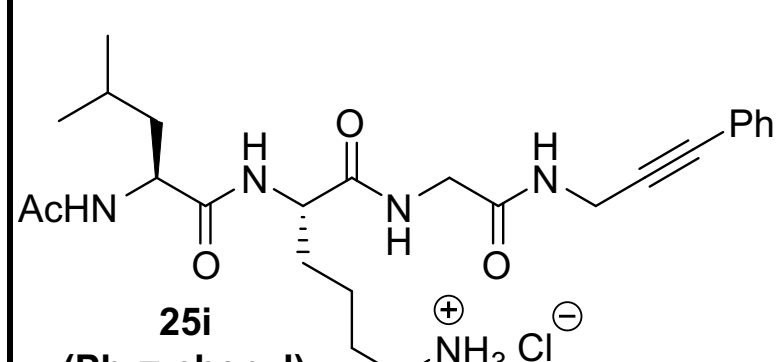 **25i** **(Ph = phenyl)**	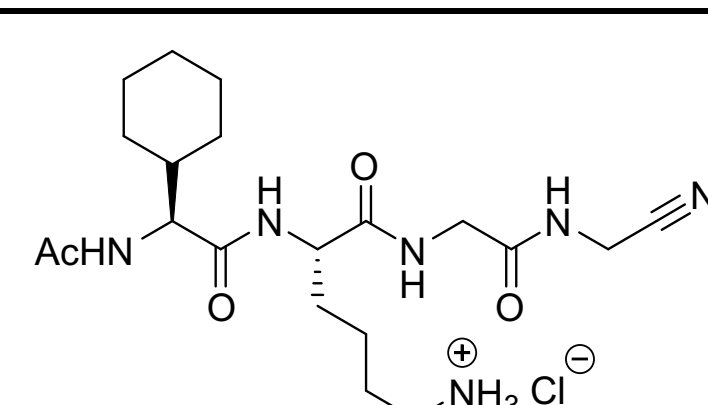 **29**
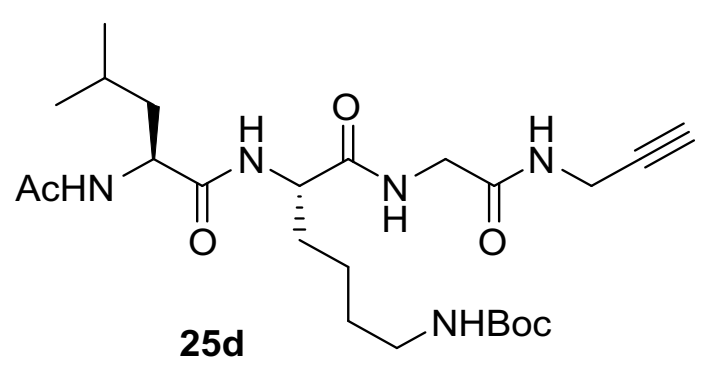 **25d**	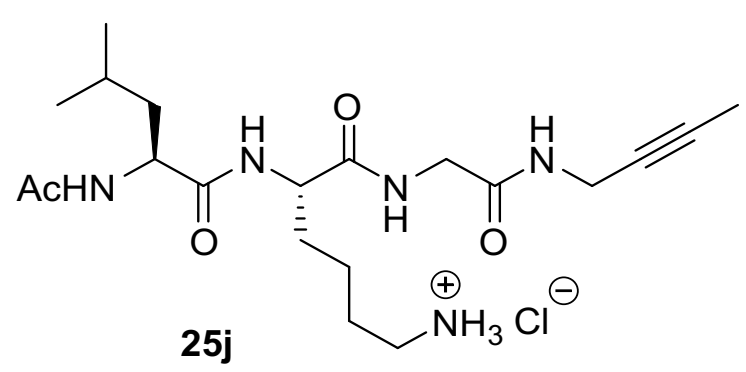 **25j**	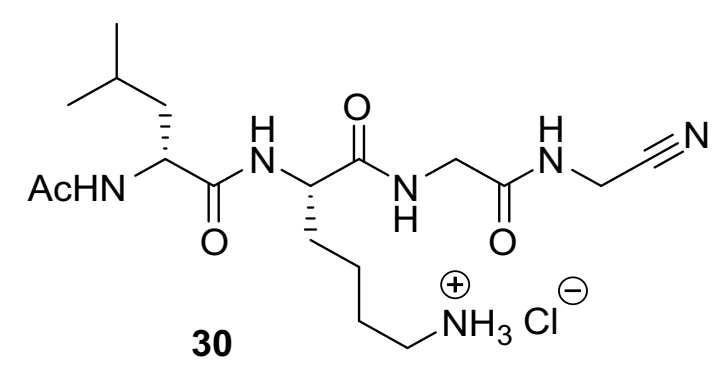 **30**
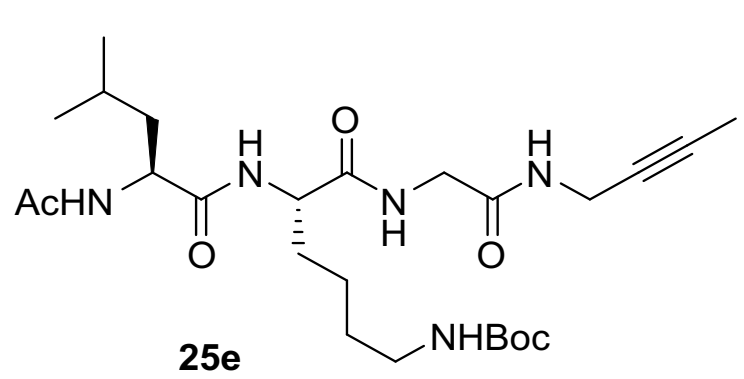 **25e**	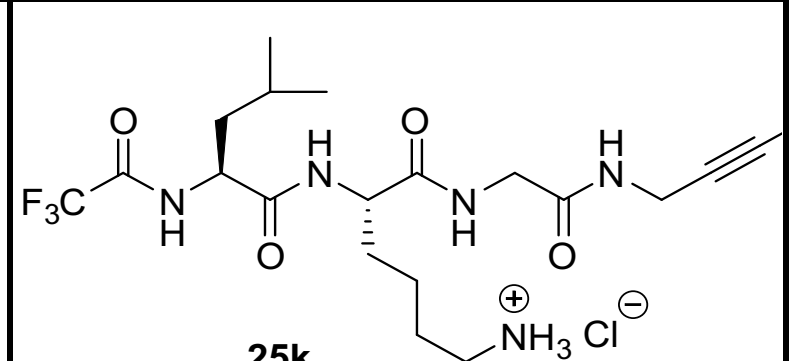 **25k**	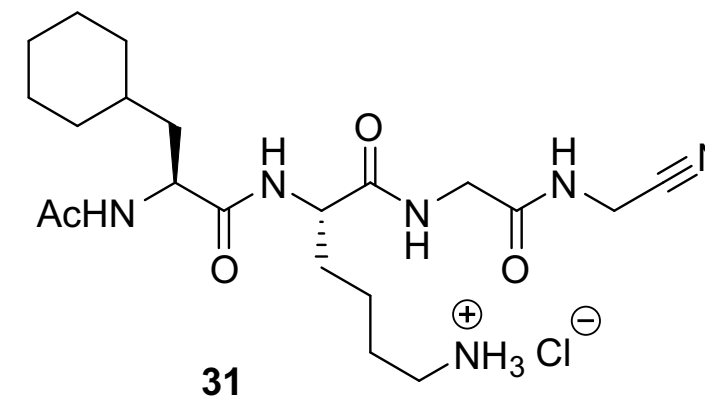 **31**

Supplementary Data S4. Effects of alterations of the R1, R2 and R3 groups of **Compound 25** on cell permeability and USP5 binding using HA-Ub-PA assays.

	R_1	R_2	R_3	Cell permeable	USP5 inhibition in lysates
25c	Me	NHBoc	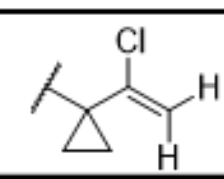	N	N
25d	Me	NHBoc	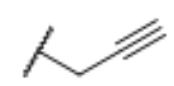	N	Y
25e	Me	NHBoc	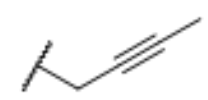	N	N
25f	Me	NHBoc	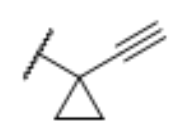	N	N
25g	Me	NHBoc	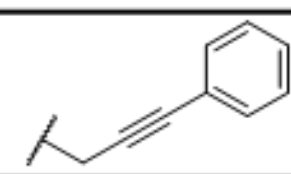	N	N
25h	Me	NH_3Cl	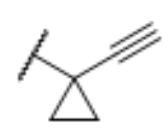	N	N
25i	Me	NH_3Cl	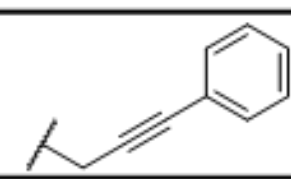	N	N
25j	Me	NH_3Cl	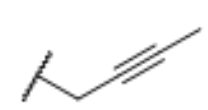	N	N
25a	CF_3	NHBoc	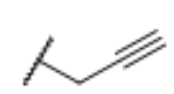	Y	Y
25k	CF_3	NH_3Cl	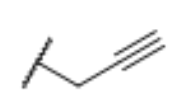	N	Y
25b	CF_3	NHTrifluoro acetyl	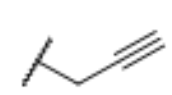	Y	Y

Supplementary Data S5. Structures of biotinylated derivatives of **Compound 25**.

Name	Structure
bio-a-25a (31)	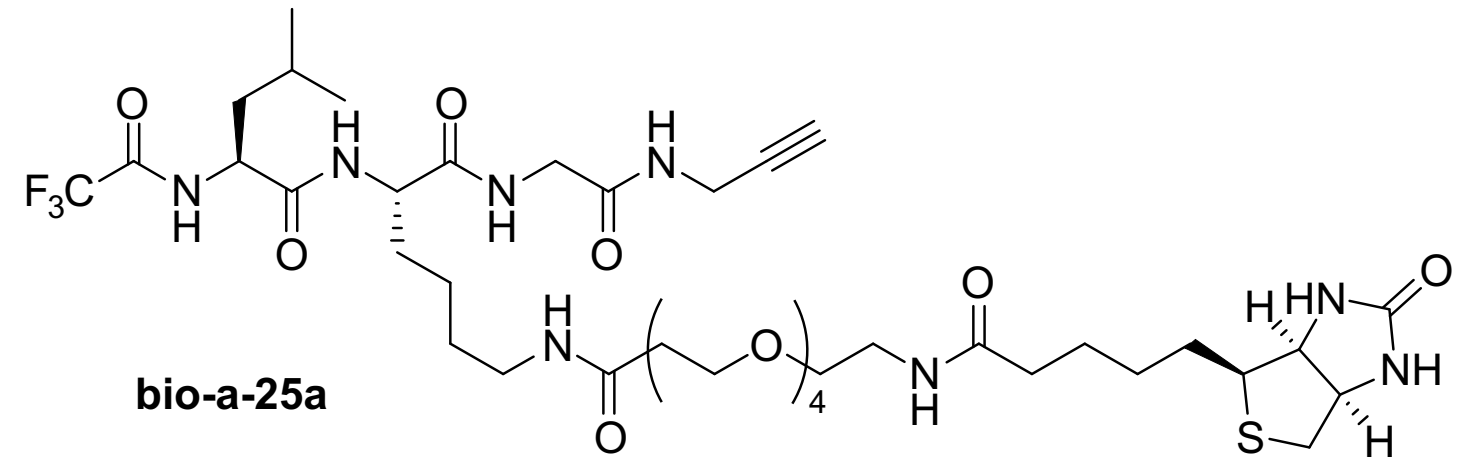 **bio-a-25a**
bio-b-25a (32)	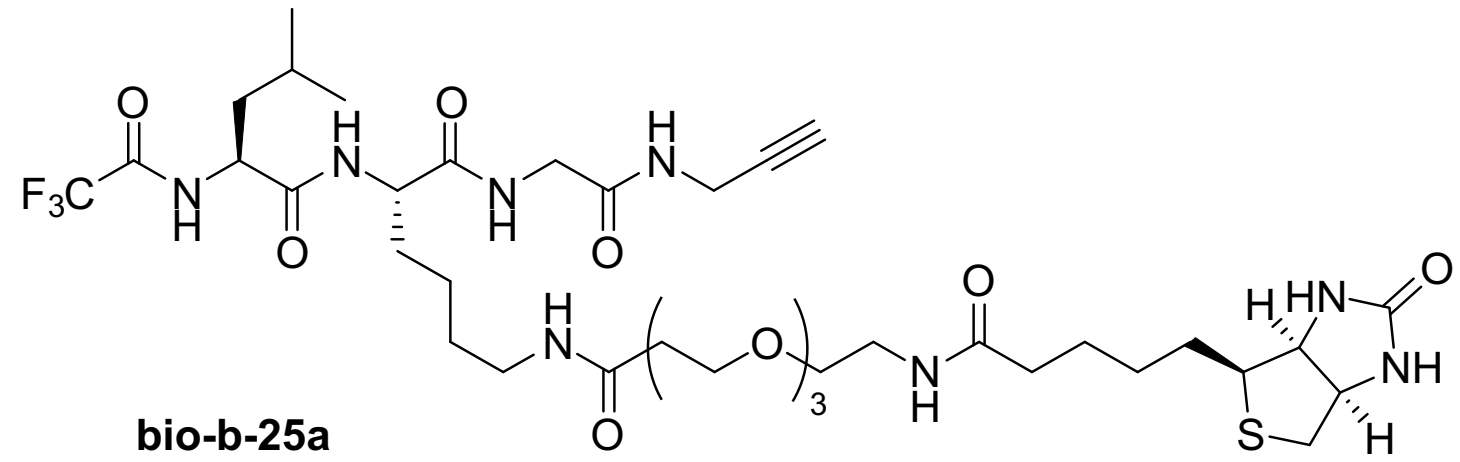 **bio-b-25a**
bio-a-I38 (34)	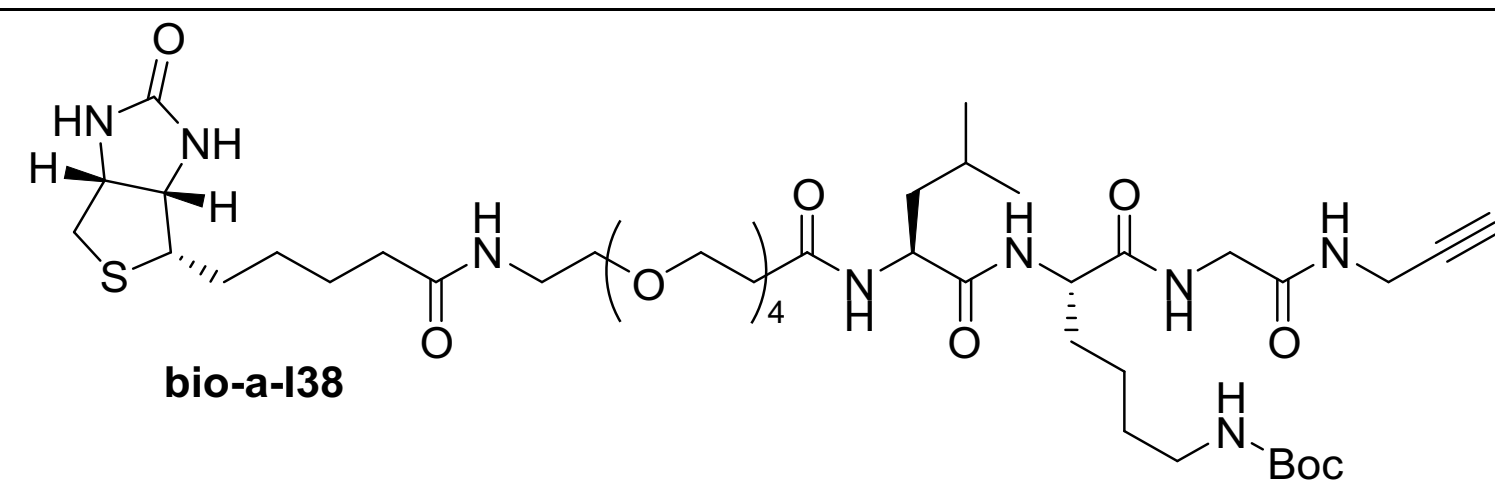 **bio-a-I38**
bio-b-I38 (33)	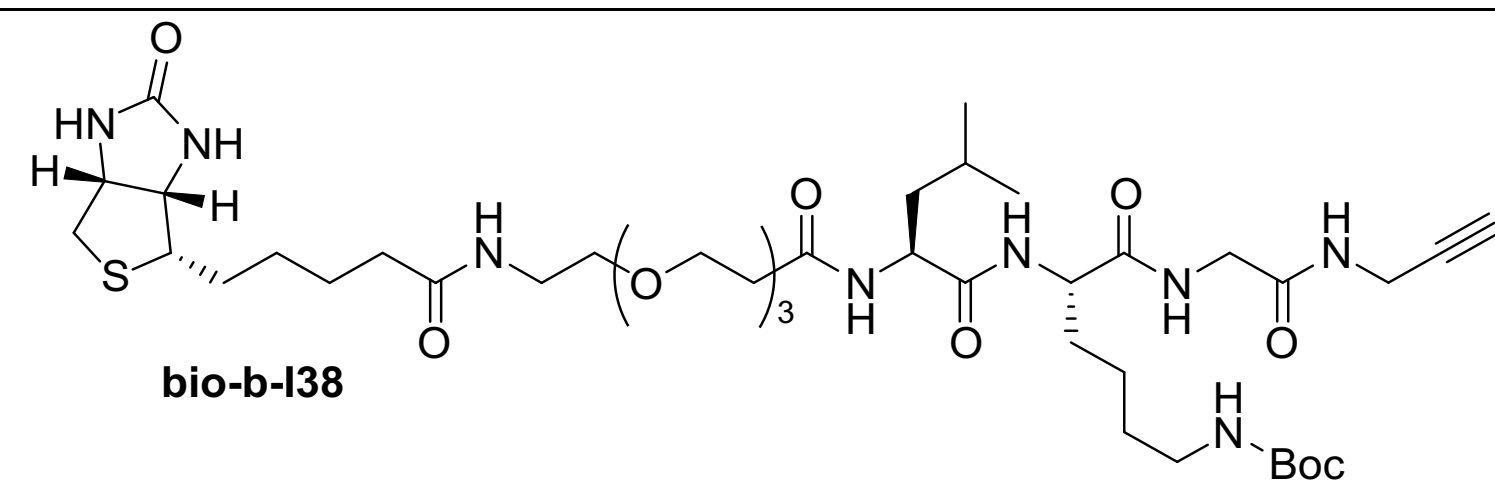 **bio-b-I38**
bio-a-25b (35)	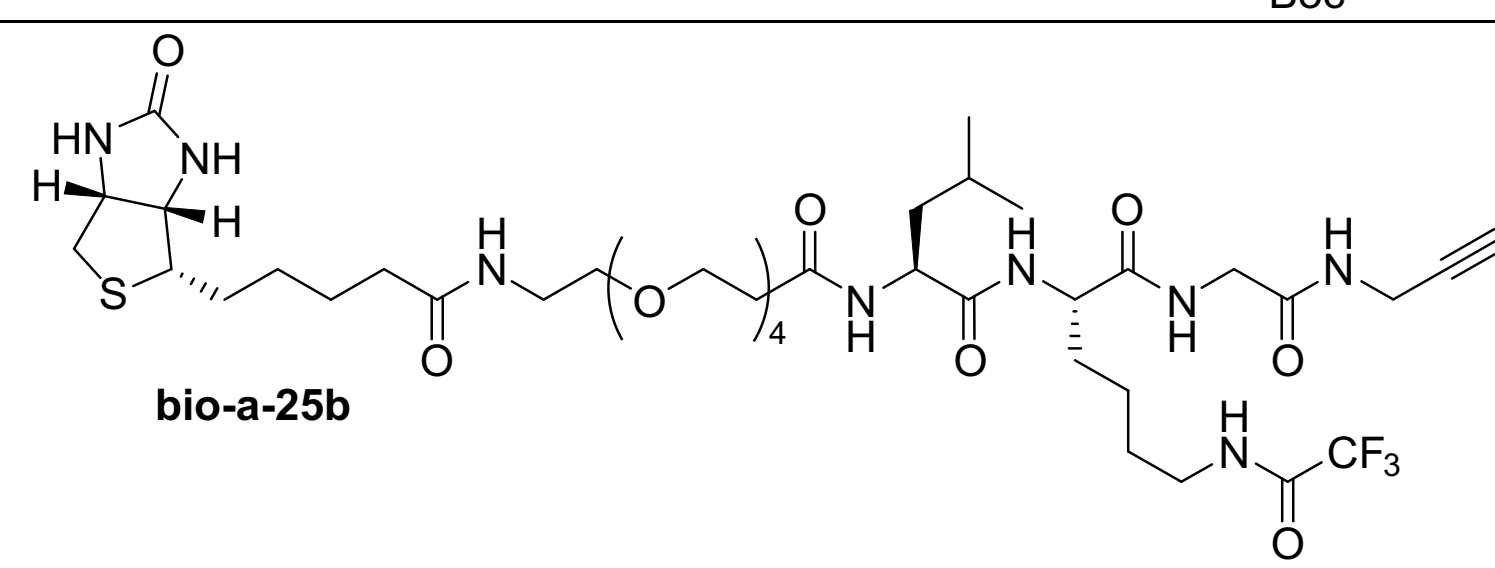 **bio-a-25b**

References

1. G. Cheng, V. Castelletto, C. M. Moulton, G. E. Newby, I. W. Hamley, Hydrogelation and Self-Assembly of Fmoc-Tripeptides: Unexpected Influence of Sequence on Self-Assembled Fibril Structure, and Hydrogel Modulus and Anisotropy. *Langmuir* **26**, 4990–4998 (2010).
2. M. Soethoudt, *et al.*, Selective Photoaffinity Probe That Enables Assessment of Cannabinoid CB2 Receptor Expression and Ligand Engagement in Human Cells. *J. Am. Chem. Soc.* **140**, 6067–6075 (2018).
3. M. Soethoudt, *et al.*, Selective Photoaffinity Probe That Enables Assessment of Cannabinoid CB_2 Receptor Expression and Ligand Engagement in Human Cells. *J. Am. Chem. Soc.* **140**, 6067–6075 (2018).
4. Y. Angell, K. Burgess, Ring Closure to β-Turn Mimics via Copper-Catalyzed Azide/Alkyne Cycloadditions. *J. Org. Chem.* **70**, 9595–9598 (2005).
5. M. Gredičak, A. Kolonić, I. Jerić, Novel chloroenyne-modified amino acid derivatives. *Amino Acids* **35**, 185–194 (2008).
6. A. Linhardt, *et al.*, Degradable, Dendritic Polyols on a Branched Polyphosphazene Backbone. *Ind. Eng. Chem. Res.* **57**, 3602–3609 (2018).
7. L. Qin, *et al.*, Visible-light-induced decarboxylative alkynylation of carboxylic acids in batch and continuous flow. *Green Synthesis and Catalysis* **5**, 20–24 (2024).
8. F. P. Bayer, M. Gander, B. Kuster, M. The, CurveCurator: a recalibrated F-statistic to assess, classify, and explore significance of dose–response curves. *Nat. Commun.* **14**, 7902 (2023).
9. M. de Munnik, *et al.*, αβ,α′β′-Diepoxyketones are mechanism-based inhibitors of nucleophilic cysteine enzymes. *Chemical Communications* **59**, 12859–12862 (2023).
10. S. C. Shin, *et al.*, Structural and functional characterization of USP47 reveals a hot spot for inhibitor design. *Commun. Biol.* **6**, 970 (2023).
11. A. P. Turnbull, *et al.*, Molecular basis of USP7 inhibition by selective small-molecule inhibitors. *Nature 2017 550:7677* **550**, 481–486 (2017).
12. M. Hu, *et al.*, Crystal Structure of a UBP-Family Deubiquitinating Enzyme in Isolation and in Complex with Ubiquitin Aldehyde. *Cell* **111**, 1041–1054 (2002).
13. L. Rougé, *et al.*, Molecular Understanding of USP7 Substrate Recognition and C-Terminal Activation. *Structure* **24**, 1335–1345 (2016).
14. Covalent Ubiquitin-Usp5 Complex. [Preprint] (2009). Available at: https://www.wwpdb.org/pdb?id=pdb_00003ihp [Accessed 19 March 2025].
15. S.-T. Gao, X. Xin, Z. Wang, Y. Hu, Q. Feng, USP5: Comprehensive insights into structure, function, biological and disease-related implications, and emerging therapeutic opportunities. *Mol. Cell. Probes* **73**, 101944 (2024).
16. V. Kapuria, *et al.*, Deubiquitinase Inhibition by Small-Molecule WP1130 Triggers Aggresome Formation and Tumor Cell Apoptosis. *Cancer Res.* **70**, 9265–9276 (2010).
17. M. K. Mann, *et al.*, Structure–Activity Relationship of USP5 Inhibitors. *J. Med. Chem.* **64**, 15017–15036 (2021).
18. L. F. Peterson, *et al.*, Targeting deubiquitinase activity with a novel small-molecule inhibitor as therapy for B-cell malignancies. *Blood* **125**, 3588–3597 (2015).
19. Y. Zheng, *et al.*, EOAI, a ubiquitin-specific peptidase 5 inhibitor, prevents non-small cell lung cancer progression by inducing DNA damage. *BMC Cancer* **23**, 28 (2023).
20. H. Potu, *et al.*, Usp5 links suppression of p53 and FAS levels in melanoma to the BRAF pathway. *Oncotarget* **5**, 5559-5569 (2014).
21. M. Altun, *et al.*, Activity-Based Chemical Proteomics Accelerates Inhibitor Development for Deubiquitylating Enzymes. *Chem. Biol.* **18**, 1401–1412 (2011).
22. O. A. Issaenko, A. Y. Amerik, Chalcone-based small-molecule inhibitors attenuate malignant phenotype via targeting deubiquitinating enzymes. *Cell Cycle* **11**, 1804–1817 (2012).
23. V. Kapuria, *et al.*, Protein cross-linking as a novel mechanism of action of a ubiquitin-activating enzyme inhibitor with anti-tumor activity. *Biochem. Pharmacol.* **82**, 341–349 (2011).

24. K. Okada, *et al.*, Vialinin A is a ubiquitin-specific peptidase inhibitor. *Bioorg. Med. Chem. Lett.* **23**, 4328–4331 (2013).
25. X. Chen, *et al.*, Mebendazole elicits potent antimyeloma activity by inhibiting the USP5/c-Maf axis. *Acta Pharmacol. Sin.* **40**, 1568–1577 (2019).
26. A. Aliabadi, et al., In vitro and in vivo anticancer activity of mebendazole in colon cancer: a promising drug repositioning. *Naunyn-Schmiedeberg's Arch. Pharmacol.* **397**, 2379-2388 (2023).
27. Z.-W. Zhou, *et al.*, Natural isoflavone formononetin inhibits IgE-mediated mast cell activation and allergic inflammation by increasing IgE receptor degradation. *Food Funct.* **14**, 2857–2869 (2023).
28. J. Meng, *et al.*, USP5 promotes epithelial-mesenchymal transition by stabilizing SLUG in hepatocellular carcinoma. *Theranostics* **9**, 573-587 (2019).
29. M. K. Mann, *et al.*, Discovery of Small Molecule Antagonists of the USP5 Zinc Finger Ubiquitin-Binding Domain. *J. Med. Chem.* **62**, 10144–10155 (2019).
30. Y. Shibata and M. Chiba, The Role of Extrahepatic Metabolism in the Pharmacokinetics of the Targeted Covalent Inhibitors Afatinib, Ibrutinib, and Neratinib, Drug Metab. Disp. 43, 375-384 (2015).
31. Gayatri, *et al.*, Thiophene-fused γ-lactams inhibit the SARS-CoV-2 main protease via reversible covalent acylation. *Chem. Sci.* **15**, 7667–7678 (2024).
